# The Morphometry of *Solenopsis* Fire Ants

**DOI:** 10.1371/journal.pone.0079559

**Published:** 2013-11-19

**Authors:** Walter R. Tschinkel

**Affiliations:** Department of Biological Science, Florida State University, Tallahassee, Florida, United States of America; VIB & Katholieke Universiteit Leuven, Belgium

## Abstract

Size-related changes of body shape were explored in 15 polymorphic species of *Solenopsis* fire ants by analyzing body weight along with linear measurements of 24 body parts. Log regression slopes were used to detect changes of shape with increasing size. Within species, the largest workers weighed from about 5 to 30-fold as much as the smallest. The range of within-species body lengths varied from 1.6 mm to 4 mm. As worker size increased, the gaster tended to make up a larger proportion of body length, usually at the cost of the petiole, and rarely at the cost of head length or mesosoma length. In most, the relative volume of the gaster increased and that of the head and mesosoma decreased. Most also showed an increasingly “humped” mesosoma. For all species, head shape changed from barrel-shaped to heart-shaped as worker size increased. Antennae became relatively shorter as the relative size of the club decreased. Shape changes of the legs were more variable. *S. geminata* was exceptional in the extreme nature of its head shape change, and was the only species in which relative head volume increased and gaster volume decreased with increasing body size. With the exception of *S. geminata*, the allometric rules governing shape are remarkably similar across species, suggesting a genus-level developmental scheme that is not easily modified by evolution. It also suggests that the evolution of shape is highly constrained by these conserved growth rules, and that it acts primarily (perhaps only) through allometric growth. The results are discussed in light of the growth of imaginal discs in a resource-limited body (the pupa). The substantial variation of allometries within species and across localities is also discussed in relation to using allometric patterns to identify species or to construct phylogenies.

## Introduction

In 1932 Huxley published *Problems of Relative Growth* [1], developing and expanding D’Arcy-Thompson’s methods for analyzing size and shape in terms of relative growth of dimensions. The analytic methods are applicable to any pair of dimensions undergoing power growth, that is, where the dimensions increase in a multiplicative rather than additive manner. More recently, the term “scaling” has been applied to such relative growth. Examples include the scaling of metabolic rate to body size [2]–[4] or to colony size [5], the increase of fire ant territory with colony size [6], the relationship of brain size [7], [8] or leg length [9] to body size, the scaling of sexual production to colony size [10], and even the scaling of foraging rate to trail dimensions in leaf cutter ants [11]. The principles of differential growth are applicable at all scales from the molecular and cellular, to organisms and even to superorganisms such as ant colonies [10], [12].

The principles of relative growth are most commonly applied to morphology. Related species are most commonly distinguished on the basis of their size and shape, and more specifically, the size and shape of their homologous body parts. These differences arise as a result of differential growth of the relevant dimensions during ontogeny, and evolve through changes in that differential growth. Because size and shape are related, shape can be expressed in a size-free manner as the ratio of dimensions [13]. Application of size-shape analyses to a series of related species can reveal to what extent size and shape are linked, and to what extent they are free to evolve independently.

For most animals, current size and shape represent a history of their relative growth, but for holometabolous insects the adult body is not the smooth continuation of the larval body, but is largely created in the pharate pupal stage through the growth of imaginal discs set aside in the embryo [14]. Allometries of adult holometabola such as ants are best understood as the products of the sizes and relative growth rates of these imaginal discs. Moreover, because the larva stops feeding before undergoing metamorphosis, all imaginal growth occurs under conditions of fixed resources in the form of storage protein and fat. Imaginal growth is thus a zero-sum game– material invested in one disc is not available for others.

Studies of the allometries of single species of ants are numerous, but will not be reviewed here. Comparative studies of the allometries of multiple species are less common. Pie and Traniello [15] carried out a principle components analysis of 231 species of *Pheidole* and found that most of the morphological differences among the species could be accounted for by allometric changes associated with body size differences. This suggested that morphological variation is highly constrained and under control of allometric growth rules, a point also made by Franks and Norris [16]. Less explicitly, Schöning et al. [17] also found regular allometric trends related to body size in 10 species of *Dorylus* army ants, with greater differentiation in larger species.

Within species, variation in differential growth commonly produces modest differences in shape and size, although it can lead to the great exaggeration of some structures in larger individuals [18]. Among about 15% of ant species, natural selection has acted on differential growth to produce workers of vastly different body size, and often shape, a phenomenon referred to as polymorphism [19]–[22]. Such within-colony variation in size and shape is usually assumed and/or shown to serve division of labor, with workers of different size and shape being associated with different tasks [17], [20], [23], [24]–[28]. The frequency distribution of body sizes (and their associated shapes) in polymorphic ants can be continuous, usually with a strong right skew (i.e. fewer larger workers), or it may be dimorphic with two distinct, discontinuous sizes present [19], or it may consist of multiple discontinuous forms [29]. These types of polymorphism result from a reprogramming of the size threshold for pupation but can also be accompanied by changes in the growth parameters [30]. Even in continuously polymorphic species such as the fire ant, *Solenopsis invicta*, there are only two classes based on this reprogramming– minor workers of small and modestly variable size, and major workers of large and much more variable size that overlap the size range of minor workers [31]–[32]. Great differences in worker size may or may not be accompanied by shape differences, but when they are, the differences result from variation in the relative growth of imaginal discs during pupal and adult development. Because these grow with fixed resources within a body of fixed size, discs compete with one another, and an increase in one is often accompanied by a decrease in another [14] resulting in non-linear log-log plots of the focal body parts. Such non-linearity has lead to the (sometimes arbitrary) designation of allometric relationships as “biphasic”, “triphasic” [19] or has been analyzed using polynomial regressions [33] or by subdividing into two separately analyzed castes [34].

Most studies of polymorphism need only to distinguish workers on the basis of their overall body size, and for this purpose only one or two measurements (e.g. head width, or pronotal length; [28]) are needed. More rarely, multiple measurements of diverse body parts are analyzed to produce a more complete picture of differential growth and size-related shape change [17], [33], [34]. Allometric estimates always compare at least two measures, that is, they estimate *relative* growth rates. Ideally one variable is a measure of the whole and the other a measure of a part, although many studies compare mostly parts, with the understanding that one serves as the estimate of the “whole.” In the present study, the sum of lengths served as the estimate of the whole. This seemed more appropriate than the body weight (or its cube root) because all allometric estimates were of linear body dimensions, which, although related to body weight, are not equivalent because air spaces and variation in tissue density affect weight in ways not reflected in length measurements.

The present study explores how size and shape vary within and between species of *Solenopsis* fire ants, a conspicuously polymorphic group of ant species. Because within-species shape differences seem often to be linked to size differences, the intent here is to determine what options are available for evolving different shapes, and to what extent these options are linked to size changes. In other words, how variable are the allometric growth rules within the genus?

## Materials and Methods

### Sample Collection and Preparation

#### Ethics statement

No permits were required to sample the ants, as most were collected on public rights of way. No protected species were sampled.

Samples of fire ant workers were collected from nests by the author, or were donated by other collectors (see Table 1). All samples were preserved in ethanol in glass vials. Many vials contained far more ants than needed for measurements. Forty workers (initially 50) were sampled from these vials such that the size extremes were somewhat over-represented in order to yield better estimates of the regression parameters. Reliable size-frequency distributions were thus sacrificed, but these were not the subject of this study anyway.

**Table 1:** The species analyzed, their collectors/donors, collection locality and vial ID number.

Species	vial	collection locality, date	collector/donor	ID by
*S. amblychila*	47	AZ, Cochise Co., 1 mi. NW Portal; VII.22.1997	RAJ	RAJ
*S. amblychila*	65	AZ, Santa Cruz, Yanks Canyon	RAJ	RAJ
*S. amblychila*	66	AZ, Santa Cruz, Sycamore Canyon, 17.iv.2006	RAJ	RAJ
*S. aurea*	5	6 m.WSW, Rodeo, NM	WRT	RAJ
*S. gayi*	69	Peru, Lima	RAJ	WRT
*S. geminata GUA&CR*	22	Guatemala, El Peten, Tikal Natl. Park; vic. of ruins. IV.25.1990	WRT	KLH
*S. geminata GUA&CR*	17	Guatemala, El Peten, Tikal Natl. Park; vic. of ruins. IV.25.1990	WRT	KLH
*S. geminata GUA&CR*	2	San Jose, Costa Rica; II.20.1985	WRT	JPP
*S. geminata GUA&CR*	6	Guatemala, Guatemala City, Zone 10	WRT	JPP
*S. geminata GUA&CR*	9	Guatemala, Panajachel, Lake Atitlan; IV.29.1990	WRT	JPP
*S. geminata GUA&CR*	10	Guatemala, Ixchimche	WRT	JPP
*S. geminata GUA&CR*	18	Guatemala, El Peten, Tikal Natl. Park; vic. of ruins. IV.25.1990	WRT	JPP
*S. geminata TX&FL*	49	Florida, Columbia Co.	WRT	KLH
*S. geminata TX&FL*	50	Florida, Columbia Co., US27 at Ichetucknee St. Pk.	WRT	KLH
*S. geminata TX&FL*	51	Florida, Columbia Co.	WRT	KLH
*S. geminata TX&FL*	53	Texas, Lampasas Co.; VI.6.1998	SDP	SDP
*S. geminata TX&FL*	54	Texas, Lampasas Co.; VI.6.1998	SDP	SDP
*S. geminata TX&FL*	55	Texas, Lampasas Co.; VI.6.1998	SDP	SDP
*S. interrupta*	42	Argentina, Santiago del Estero, Va San Martin, no date	JPP	JPP
*S. interrupta*	70	Argentina, Salta, RT 51	RAJ	RAJ
*S. invicta*	61	Argentina, Santa Fe Prov., San Justo; IV.2001	SDP	SDP
*S. invicta*	62	Argentina, Santa Fe Prov., San Justo; IV.2001	SDP	SDP
*S. invicta*	60	Argentina, Santa Fe Prov., San Justo; IV.2001	SDP	SDP
*S. macdonaghi*	71	Argentina, Entre Rios, Jct. Rts. 40 & 130; XII.18.2005	RAJ	JPP
*S. macdonaghi*	43	Brazil, Mato Grosso del Sul, Jct. Rt. 141 at Itaquirai;	RAJ	JPP
*S. megergates*	44	Brazil, Parana, Rt. 116, Rio Negro; no date	JPP	JPP
*S. pythia*	52	Argentina, Missiones Prov., S. of Posadas	SDP	SDP
*S. quinquecuspis*	45	Argentina, Santa Fe Roldan	JPP	JPP
*S. richteri*	72	Argentina, San Luis, Sierra San Luis; XII.26.2005	RAJ	JCT
*S. richteri*	41	Saltillon, MS, lab colony	SDP	JPP
*S. saevissima*	46	Brazil, Sergipe, Rt. 101, Propria; I.2001	JPP	JPP
*S. xyloni AZ*	68	AZ, Tempe	RAJ	RAJ
*S. xyloni OK*	56	OKLAHOMA, Caddo; XI.22.1998	SDP	SDP
*S. xyloni OK*	57	OKLAHOMA, Caddo; XI.22.1998	SDP	SDP
*S. xyloni OK*	58	OKLAHOMA, Caddo; XI.22.1998	SDP	SDP

Each vial contained workers from a single colony. The species are in alphabetical order. Abbreviations: WRT, the author; RAJ, Robert A. Johnson; JPP, James P. Pitts; JCT, James C. Trager; KLH, Kevin L. Haight; SDP, Sanford D. Porter.

The species, their sources, their localities and the identifying specialist are listed in Table 1. When a species was collected from two very distant localities, the localities were analyzed at the species level as though they were different species. Thus, colonies of *S. geminata* collected in the USA (Florida and Texas) were designated FL&TX, and those from Central America (Guatemala and Costa Rica) were designated GUA&CR. *S. xyloni* was collected from Arizona and Oklahoma (designated AZ and OK).

**Figure 1:**
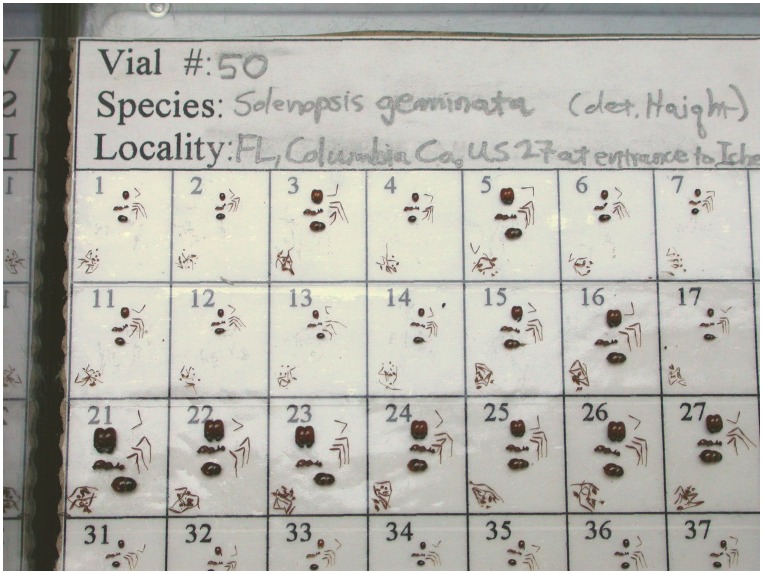
Disassembled ants were arranged on a gridded card covered with double stick tape in preparation for photography and measurement. A 2 mm scale was photographed at the same magnification to serve as the scale for measuring lengths.

Workers selected for measurement were dried for dry weight determination on a microbalance, and then rehydrated enough so that they could be broken into several pieces without shattering. The pieces (head, antennae, legs, mesosoma+petiole+postpetiole, gaster) were arranged on a gridded, numbered card that was covered with double-stick tape to hold the pieces in place (Fig. 1). The head was arranged in a face-on view, the mesosoma in lateral view and the gaster in dorsal view. The number associated with each grid unit, along with the vial ID identified each worker throughout the analysis.

**Figure 2:**
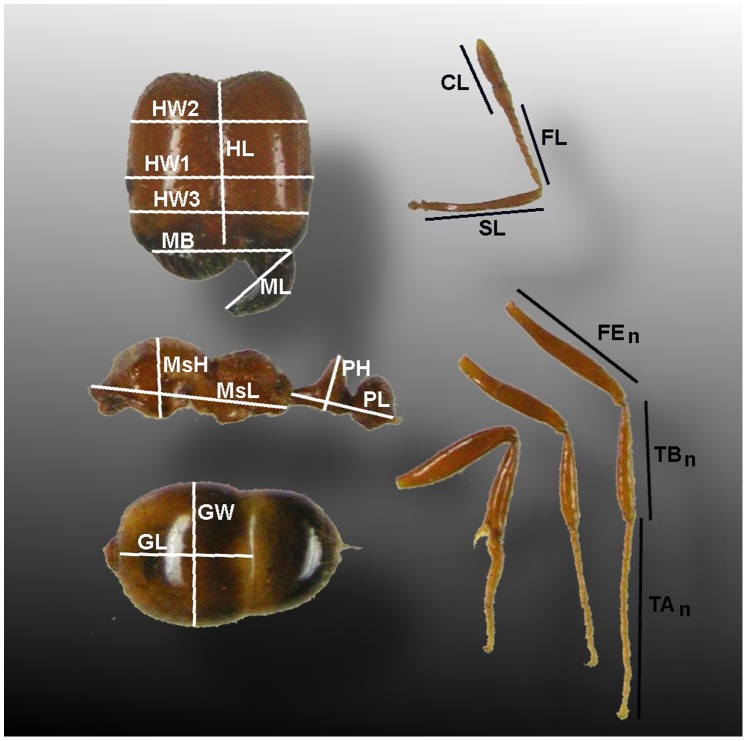
The measures taken, and the aspect measured. Abbreviations are explained in Table 2.

The worker pieces within each grid unit were photographed under a Leitz MZ12.5 dissecting microscope after first photographing a 2 mm scale at the same magnification. A standard set of measurements was taken from these photographs (Fig. 2) using ImageJ (http://rsbweb.nih.gov/ij/) set to the 2 mm scale. All data from ImageJ were thus in mm, and were the basic data upon which all subsequent analyses were based.

Some measurements were combined for analysis (Table 1). The length of the petiole and postpetiole are reported here simply as petiole length (PL). Similarly, leg lengths were the sums of the femur length, tibia length and tarsal length. Although “alinotum” is used in some reports, the more commonly accepted term for the mid-body of ants, “mesosoma” (abbreviated Ms) is used here.

#### Measures of the whole

In this study, the sums of lengths serve as such measurements of the whole. Body length (BL) is the sum of head length, mesosoma length, petiole length and the length of the first segment of the gaster. This sum is preferable to body weight or body volume because these are likely to be more variable because of changes in fatness and internal air spaces. Antennal length (ANT) is the sum of the club, flagellum and scape, and leg lengths are the sums of femur, tibia and tarsal lengths.

The basic data are available in Appendix S1 as a tab-delimited file. Two additional measures of the mesosoma (MsL-1 and MsL-2) reported in the Appendix were not used in this analysis.

### Data Analysis

The object of most of the analysis was to determine how the shape of the component parts changed with worker body size, colony of origin, collection locality and species. For localities from which multiple colonies of a species had been collected, a maximum of three colonies was prepared for analysis. Similarly, for species collected from multiple localities, a maximum of three localities were analyzed. As is appropriate for morphometric analysis of relative growth, all data were log transformed. Relative growth was determined for each body measurement in relation to a measure of the whole (body length, leg length, antenna length, etc.). Body length was the sum of head length (HL), mesosoma length (MsL), petiole length (PL) and the length of the first gastral tergite (GL). Because the gaster is capable of changing length through telescoping, only the length of the first segment was used. Note that this body length is not the “true” body length, although certainly proportional to it. The measures and their abbreviations are listed in Table 2.

**Table 2:** The measures, as shown in Fig. 2, and their abbreviations.

Abbreviation	Description of measure
HL	headlength at midline from posterior margin to clypeal margin
HW1	headwidth across eyes
HW2	headwidth at ¾ of HL
HW3	headwidth at ¼ of HL
MB	width of mandible base
ML	mandible length
SL	scape length
FL	flagellum length (without club)
CL	club length
MsL	mesosomal length
MsH	mesosomal height
FE#	femur length of leg #
TB#	tibial length of leg #
TA#	tarsal length of leg #
PL	length of petiole+postpetiole
PH	height of petiole
GL	length of first gaster tergite
GW	maximum width of first gaster segment
BL	body length = HL+AL+PL+GL
ANT	antennal length = SL+FL+CL

Changes of shape associated with changing size were detectable as deviations from isometry, i.e. a slope of the log-log regression significantly different from 1.0. However, deviation from isometry is more easily seen in a graph when the ratio of the measure to the whole is regressed against the measure of the whole (both logged). Deviations from isometry are then detectable as slopes that deviate significantly from zero [13]. For convenience, the ratios will be henceforth designated as R_x_, where R is the ratio of a measure (as in Table 2) to a measure of the whole, x. Specifically, R_BL_ is the ratio of any of the dimensions in Table 2 to body length (BL); R_ANT_ is the ratio of any dimension to total antennal length (ANT); R_HL_ is the ratio of a dimension to head length (HL), R_HW1_ is the ratio of dimensions to HW1; R_HW3_ is the ratio of dimensions to HW3. The logs of these ratios were usually regressed against the log of the same measure of the whole. For example, all log R_BL_ were regressed against log BL; log R_ANT_ against log ANT and log R_HL_ against log HL and so on. For convenience, the term “log” will usually be omitted below with the understanding that all regressions were made with logged data.

After the deletion of outliers (maximum of 3), the coefficients of these regressions of R_x_ vs. x for each worker sample formed the second level of analysis. Regressions were also tested for non-linearity of the log-log relationship, and for those that deviated significantly, the polynomial regression coefficients were also recorded.

### Body Volume

The volume of body components gives a more realistic estimate of allometry of these tagma because it integrates multiple dimensions such as the width and height. Gaster volume was estimated as an ovoid solid with major axis GL, and minor width axis GW/2 and minor height axis GW/2, i.e. the gaster volume was generated by rotating GW/2 around the GL axis. Head volume was considered to consist of three approximate sections, each 1/3^rd^ of HL times HW times half the headwidth HW, and was calculated as follows: HV = (HW1×HW1/2×HL/3)+(HW2×HW2/2×HL/3)+(HW3×HW3/2×HL/3).

Because both HWn and GW were positively allometric to BL in most of the species, relative head size and gaster size increased in most species. Body weight (WT) and volume are probably nearly isometric, that is, the slope of their log-log regression would be near 1.0. Thus, regressing the estimated volume of a body part vs. body weight should reveal changes in the proportion of body weight composed by the focal body part. The log of gaster volume (GV) and head volume (HV) were regressed against log WT for each species. Slopes greater than 1.0 indicated that relative gaster size increased with body weight, a slope less than 1.0 that it decreased and a slope of 1.0 that it did not change.

Mesosoma volume was roughly approximated by multiplying its lateral, approximately triangular aspect by its approximate side-to-side dimension (MsH/2). Thus, AV = (MsL x MsH/2) x MsH/2.

Petiole volume was approximated by assuming each of its segments to be approximately a triangular solid in which PV = PL/2×(PH/2)^2^.

The sum of these volumes formed the total body volume.

## Results

### Size Variation and Range

**Figure 3:**
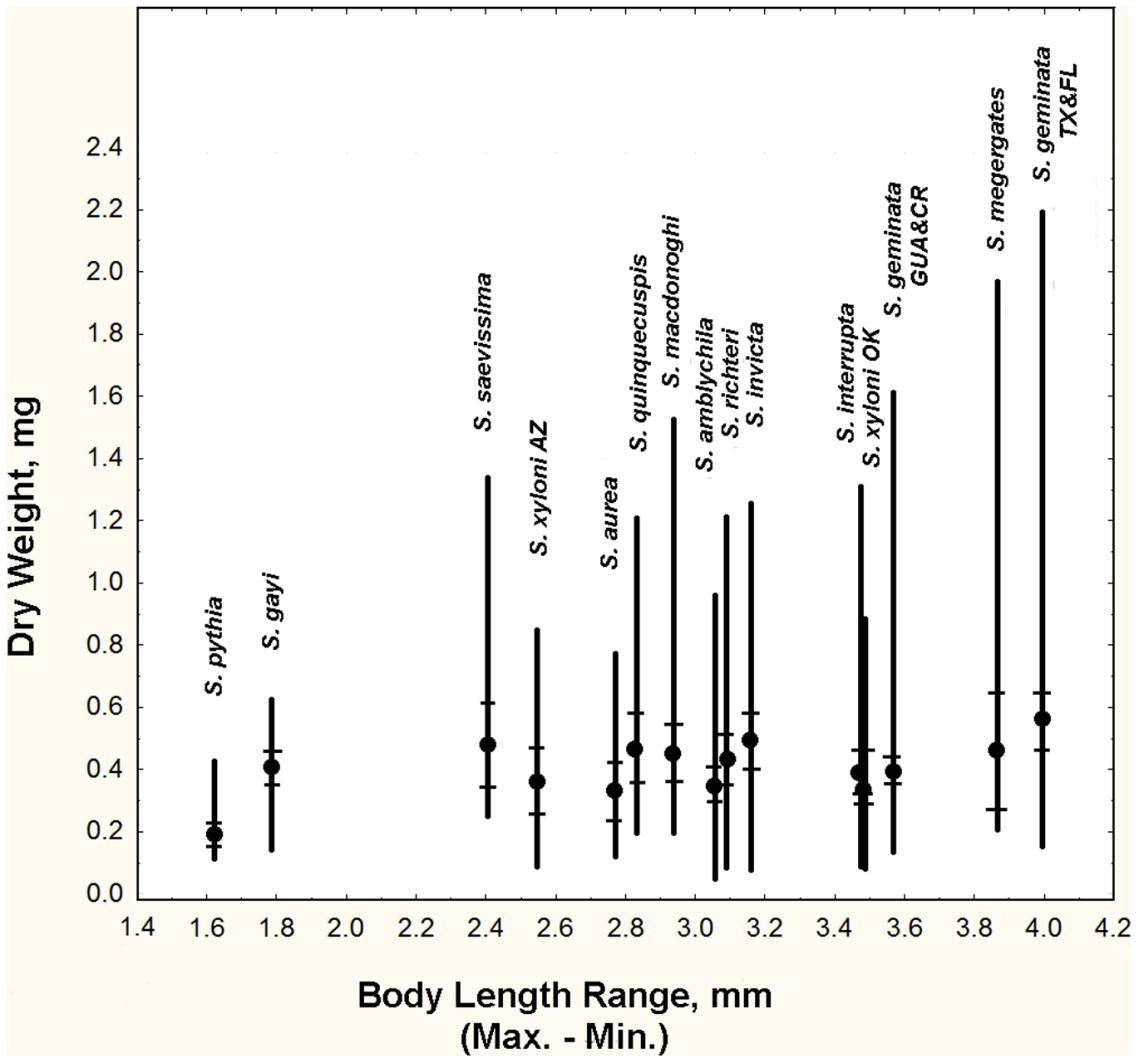
The mean, minimum and maximum dry weights of workers in relation to the range of body length (max. – min.). Body weight range increased more rapidly than body length range because the former is related to the cube of the latter, but changing allometry of body parts contributed to the irregularity of this trend. Maximum worker weight varied a great deal more than minimum. The hatches show 95% confidence intervals for the means.

Across species, the dry weight of *Solenopsis* workers ranged from as little as 0.04 mg to 2.20 mg, but this variation was not equally distributed across species (Fig. 3). The smallest minor workers were found in *S. amblychila* (0.04 mg) and the largest in *S. megergates* (0.16 mg, Fig. 3; Table 2). The smallest major workers occurred in *S. pythia* (0.42 mg), and the largest in the *S. geminata* from the USA at about 2.2 mg (a single exceptional worker weighed 5.5 mg, but was not used in the calculations below). The mean body dry weight among the species varied by only 2.5-fold, but within-species body weight (largest/smallest) varied greatly, describing the degree of worker polymorphism across species (Fig. 3; Table 3). Arranging the species in the order of their maximum size (dry weight) or size range (maximum minus minimum) was similar, but differed substantially from arranging them in order of fold-difference between the largest and smallest workers (Fig. 3; Table 4). *S. amblychila* and *S. megergates* were especially different in rank, the former because its smallest workers were very small, and the latter because its smallest workers were very large. The least polymorphic was *S. pythia* in which the largest workers weighed about 4.7 times as much as the smallest, but *S. gayi* was not far behind at 5.3 fold. The greatest polymorphism occurred in *S. geminata* (TX & FL) in which the largest workers weighed about 30 times as much as the smallest. The next most polymorphic were the Central American *S. geminata, S. invicta* and *S. amblychila,* all with a difference of about 24 to 28-fold between the smallest and largest worker. All other species had ratios between 8 and 18-fold. Overall, *S. pythia* and *S. gayi* were weakly polymorphic or perhaps not at all, while all other species showed varying degrees of polymorphism.

**Table 3:** Body dry weight (WT) and body length (BL) means and extremes of species of *Solenopsis*.

Species	Rank byWt. range(ascending)	Meandry wt.,mg	Minimumdry wt.,mg	Maximumdry wt.,mg	factordifference(min./max)	rangemax-min	Rank byBL range(ascending)	MeanBL(mm)	Min.BL(mm)	Max.BL(mm)	range(max-min)	factordifference(Max/min)
*S. pythia*	1	0.19	0.090	0.420	4.67	0.33	1	3.12	2.53	4.14	1.62	1.64
*S. gayi*	2	0.41	0.135	0.718	5.32	0.58	2	3.99	2.88	4.66	1.79	1.62
*S. aurea*	3	0.33	0.100	0.820	8.20	0.72	5	3.61	2.55	5.32	2.78	2.09
*S. amblychila*	4	0.35	0.040	0.960	24.00	0.92	8	3.63	2.28	5.34	3.07	2.35
*S. xyloni AZ*	5	0.36	0.070	1.170	16.71	1.10	4	3.47	2.37	4.92	2.55	2.08
*S. quinquecuspis*	6	0.47	0.120	1.230	10.25	1.11	6	4.11	2.97	5.82	2.85	1.96
*S. xyloni OK*	7	0.35	0.070	1.210	17.29	1.14	12	3.91	2.68	6.18	3.50	2.31
*S. richteri*	8	0.44	0.070	1.234	17.63	1.16	9	3.89	2.67	5.77	3.10	2.16
*S. interrupta*	9	0.39	0.088	1.310	14.89	1.22	11	4.01	2.57	6.05	3.49	2.36
*S. saevissima*	10	0.48	0.100	1.480	14.80	1.38	3	3.64	2.92	5.33	2.41	1.83
*S. macdonaghi*	11	0.46	0.120	1.541	12.84	1.42	7	3.85	2.91	5.85	2.95	2.01
*S. invicta*	12	0.49	0.070	1.960	28.00	1.89	10	3.84	2.50	5.67	3.17	2.27
*S. geminata GUA&CR*	13	0.40	0.070	2.020	28.86	1.95	13	3.82	2.64	6.23	3.58	2.36
*S. megergates*	14	0.46	0.160	2.410	15.06	2.25	14	3.95	3.01	6.89	3.88	2.29
*S. geminata TX&FL*	15	0.56	0.100	3.000	30.00	2.90	15	3.81	2.57	6.59	4.01	2.56

The means are not ideally estimated because workers of extreme sizes were represented at greater than random proportions. The ratio of maximum to minimum weights is an estimate of the degree of polymorphism. Ranked by weight range and body length range (max.-min.).

**Table 4:** Regression parameters for the log ratio of gaster volume: body weight (GV/BW) vs. body weight (BW) for all species.

GV/BW vs. BW							HV/BW vs. BW				
Species	code	slope	s.e. of slope	t-value	p-value	% change in relative GV for 10-x in BW	slope	s.e. of slope	t-value	p-value	
*S. xyloni AZ*	115	0.008	0.030	0.26	0.797	–				n.s.	
*S. amblychila*	101	−0.025	0.027	−0.932	0.353	–				n.s.	
*S. richteri*	107	0.034	0.024	1.44	0.154	–	−0.059	0.023	−2.56	0.013	
*S. invicta*	112	0.050	0.031	1.61	0.111	–	−0.049	0.024	−2.07	0.041	
*S. interrupta*	102	0.101	0.034	2.98	0.004	+26				n.s.	
*S. quinquecuspis*	106	0.129	0.034	3.75	0.001	+35				n.s.	
*S. xyloni* OK	111	0.133	0.029	4.64	0.00001	+36				n.s.	
*S. megergates*	104	0.145	0.048	3.01	0.005	+40				n.s.	
*S. macdonaghi*	103	0.146	0.039	3.77	0.0003	+40				n.s.	
*S. aurea*	110	0.184	0.031	5.95	0.000001	+53	0.138	0.023	6.09	0.000000	
*S. pythia*	105	0.225	0.088	2.560	0.015	+68				n.s.	
*S. gayi*	114	0.258	0.053	4.84	0.00002	+80	0.116	0.034	3.371	0.002	
*S. geminata* GUA&CR	113	−0.070	0.010	−7.04	0.000000	−15	0.153	0.011	13.31	0.000000	
*S. saevissima*	108	−0.211	0.054	−3.92	0.0004	−38	−0.295	0.049	−5.97	0.000001	
*S. geminata* TX&FL	116	−0.175	0.0207	−8.45	0.000	−37	0.117	0.025	4.722	0.000004	

Significantly positive slopes (red) indicate that the relative gaster size increases as body size increases, and negative slopes (blue) that it decreases. The species are arranged in order of increasing slope. The Factor Increase is the factor by which the ratio would change for a 10-fold increase in body weight (BW).

**Figure 4:**
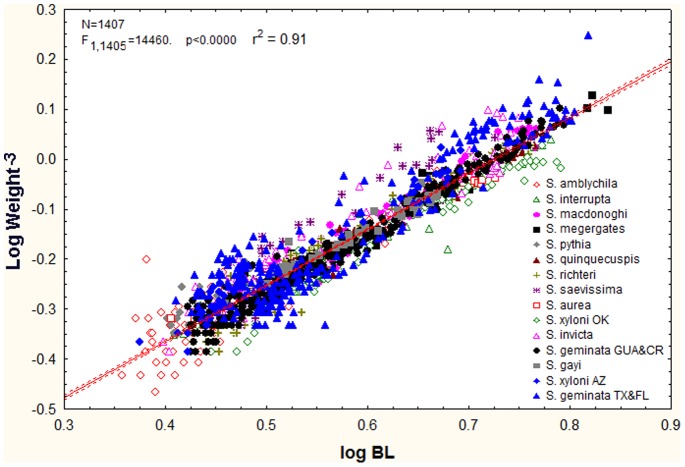
Body length is strongly correlated with body weight (here transformed to the same dimensional order as the cube root of dry weight). When all species were combined, weight^−3^ increased slightly faster than length (log log slope = 1.12). Thus, after adjusting for dimensions, larger workers were somewhat heavier than small ones, suggesting allometries or changes in density not apparent in BL. However, whereas the majority of the species were positively allometric, some were isometric (see text).

Total body length showed similar, though muted patterns because the weight is approximately related to the cube of the dimensions (Fig. 4). Minimum body length ranged from about 2.3 mm in *S. pythia* to 3.0 in *S. megergates*; maximum body length ranged from 4.1 mm in *S. pythia* to 6.9 mm in *S. megergates*. The range of body length was thus least in *S. pythia* (1.6 mm) and greatest in *S. geminata* TX & FL (4.0 mm) which just edged out *S. megergates* (3.9 mm). The corresponding factors of difference in body length between the smallest and largest workers were 1.6 for *S. pythia* and 2.6 in *S. geminata* (TX & FL). These dimensional changes are associated with weight differences of about 5 to 30-fold (see above).

As a first approximation and assuming no change of shape, weight should be proportional to the cube of the dimensions (i.e. BL). However, this condition of isometry held for only some of the species (log-log slope not significantly different than 1.0; *S. interrupta, S. aurea, S. xyloni, S. gayi*). The majority of species were positively allometric (slope>1.0), although *S. megergates* was barely so, and *S. pythia* was slightly negatively allometric (slope <1.0). A 10-fold increase in body length was associated with a 12 to 13-fold increase in dry weight in most positively allometric species, but only an 8-fold increase in the negatively allometric *S. pythia*. At the extreme of positive allometry was *S. saevissima* in which weight increased over 20-fold for a 10-fold increase in body length.

### Size-related Shape

Size-related difference of shape is the main subject of this paper. The range of body size within species, whether measured by weight or dimensions, defines the “space” in which allometric growth can occur, that is, in which individuals can evolve and develop differences in body shape. The question in the *Solenopsis* fire ants is to what degree are increases in worker size range associated with changes in shape, and to what degree is increase in size range necessary for a change of shape? Let us first consider how the components that make up body length change as body length increases. Then using body length (BL) as a measure of the whole, we will consider how the shapes of body parts change with body size, and finally, how shape of component parts relate to the size of those parts.

### Allometry of Body Length

**Figure 5:**
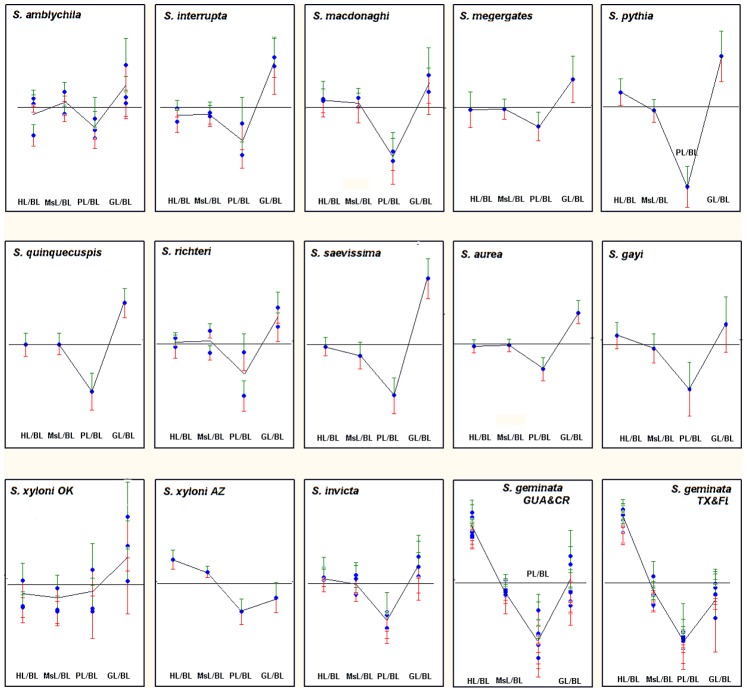
Comparison within species of the allometry of the body parts making up the body length (BL). Slopes for R_BL_ of each body part regressed against BL is shown. The mean and 95% CI (green = +, red = −) of each colony sample is shown. Isometry is indicated by the horizontal line at zero on the ordinate. In most species, GL was positively allometric, PL negatively and HL and AL isometric. S. geminata was exceptional in these allometries.

**Figure 6:**
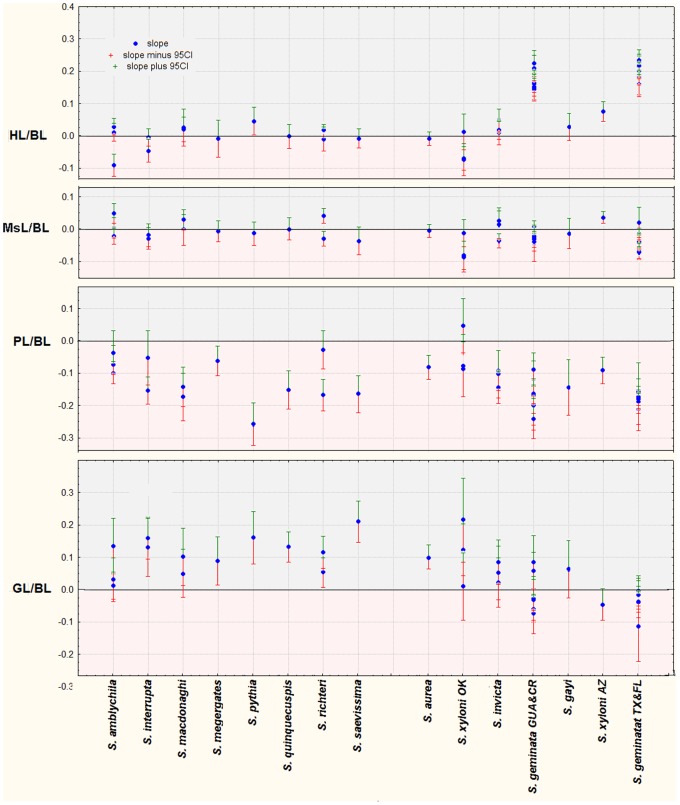
Comparison across species of slopes and 95% CI for the R_BL_ of each measure regressed against BL. Slopes significantly greater than zero are positively allometric (grey background; the measure become relatively larger) and less than zero are negatively allometric (pink background; the measure becomes relatively smaller). This figure allows comparison of the allometries among species.

Figs. 5 and 6 summarize how the components of body length (sum of head, mesosoma, petiole and gaster-1 length) change as body length increases. In Fig. 5, the log-log slope of the regression of tagma length R_BL_ vs. BL is presented by species, and in Fig. 6 it is presented by ratio. A slope of zero indicates isometry, and the 95% confidence intervals support decisions regarding allometry/isometry.

Figs. 5 and 6 show that in only three species (*S. geminata* GUA& CR; *S. geminata* TX & FL; *S. xyloni* AZ) do size-dependent changes in the relative size of the four tagma making up the body length involve changes in relative head length (HL). This is especially dramatic in the two samples of *S. geminata* in which the relative head length increased greatly with total worker size (BL). Doubling BL was associated with an approximately 6-fold increase in HL.

In only one species (*S. xyloni* AZ) was the mesosoma length allometric (positively) with body length, although one of the two samples of *S. richteri* was as well. In all others, MsL was isometric with BL.

In most species, an increase in the relative length of gaster-1 (significantly positive slope) was accompanied by a decrease in the relative length of the two petiole segments (significantly negative slope). In several cases where multiple samples were analyzed, one sample showed a significantly positive or negative slope, while the other did not (*S. amblychila, S. macdonoghi, S. richteri; S. xyloni OK; S. geminata*).

If all BL components were isometric, the slopes of their regressions would be near zero. The more allometric a component, the greater would be the slope’s deviation from zero, either positively or negatively, and the greater would be its absolute value. Therefore a rough estimate of the total degree of allometry of BL components was made by summing the absolute values of the regression slopes. Greater deviations from isometry would yield larger absolute sums. These sums varied by 3-fold across species. It also seemed reasonable to suppose that the degree of allometry might be related to the worker size range (either length or weight), but it was not. Shape changes seem not to be driven primarily by variation in the components of body length.

### Allometry of Body Parts and Appendages

**Figure 7:**
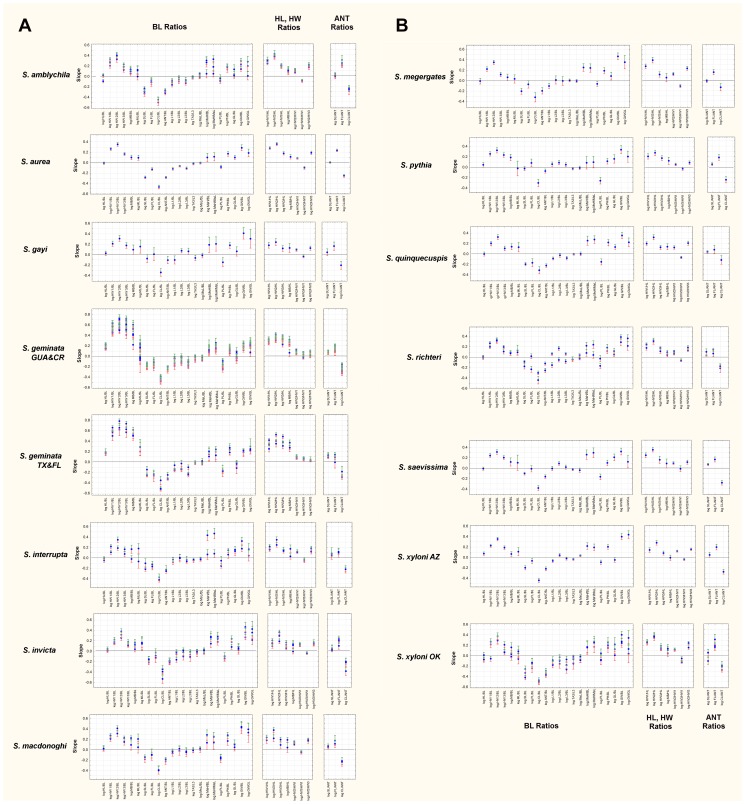
ab. All slopes arranged in species panels, allowing comparison of allometries within species. The left panel includes slopes of R_BL_ vs. BL, the central panel, R_HL_ and R_HW1_ and R_HW3_ slopes and the R_ANT_ slopes. Figs. 7a and 7b together present all the species.

**Figure 8:**
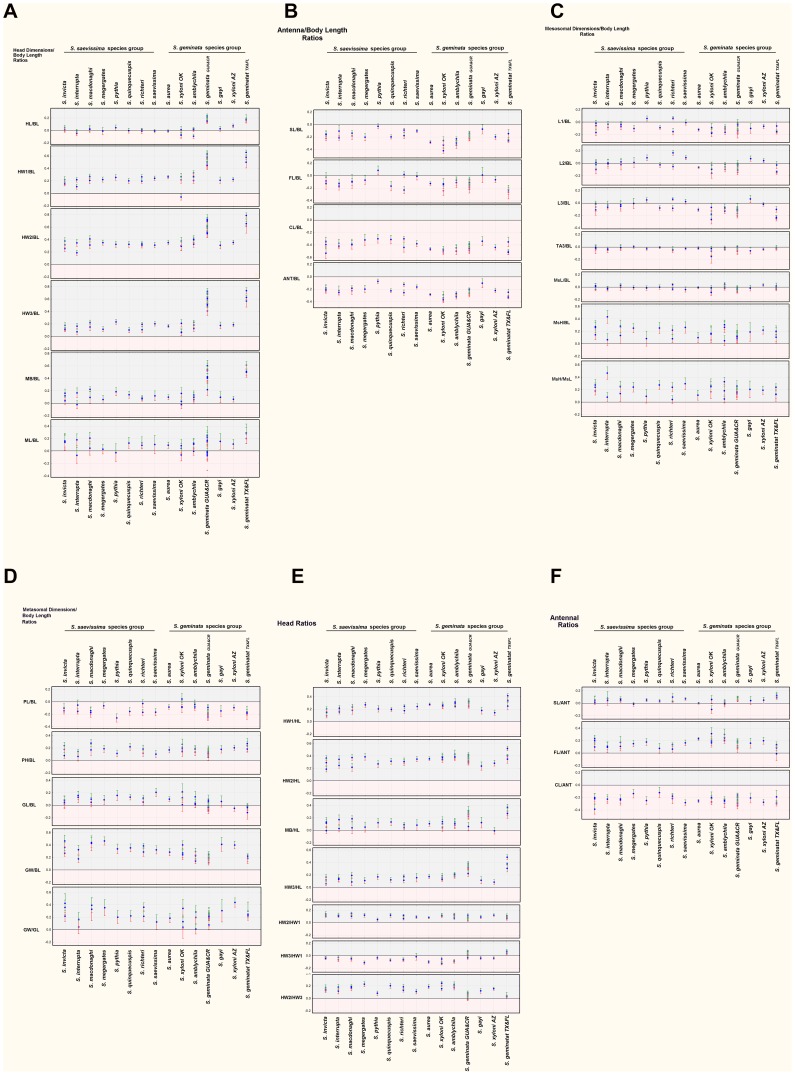
a–f. The slopes of the ratio regressions arranged for comparison across species. a. head-body length allometries; b. antennal allometries; c. mesosomal allometries; d. metasomal allometries; e. within-head allometries; f. within-antenna allometries. Positive allometries are shown on a grey background, negative on pink. Error bars are +95%CI (green) and −95%CI (red).

**Figure 9:**
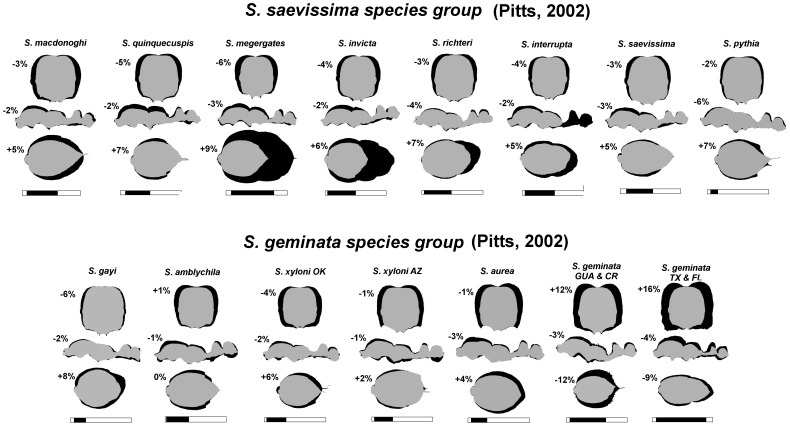
Sillhouettes of the smallest worker body parts (grey) superimposed on those of the largest workers (black) scaled to the same HL, MsL or GL, illuminating the changes of shape occurring during growth. Images of heads were resized until HL was equal for the smallest and largest worker heads. For images of the mesosoma, MsL was set to be equal (ignoring the petiole), and for images of the gaster, GL was set to be equal. The black zone in the bar under each species shows the size range (BL) of workers on a 5 mm scale. The percent values next to each silhouette are the changes in volume of the body part as a fraction of the whole body (i.e. if the head is 20% of the total body volume in small workers, and 25% in large, the change would be +5%).

Fig. 7 presents the slopes of the regression of each ratio for each species. The first panel of each figure presents the slopes of R_BL_ vs. log BL (although ratios with MsL and GL are included as well), the second panel presents head measure R_x_ in relation to HL, HW1 and HW3, and the third R_ANT_ in relation to ANT. Together, for each species, each panel describes size-related changes of shape with increasing body length, mesosomal length, gaster length, head length, head width and antenna length. In Fig. 8 each panel presents the slopes for each ratio for all 15 species, allowing easier assessment of how these ratios vary across species. The strength of shape changes can be seen in Fig. 7 and Fig. 8 as the positive or negative deviations of the slopes from zero. For ease of reading Figs. 7 and 8, positive slopes fall into the grey zone and indicate that a dimension increases faster than the measure of the whole. Negative slopes fall into the pink zone and indicate that a dimension increases more slowly than the dimension of the whole. Slopes not different from zero indicate the absence of shape change with increased size (isometry). The effect of these allometric changes can be seen in Fig. 9 in which a size-free silhouette of the body parts of the smallest workers of each species is superimposed over that of the largest, scaled to the same size. For example, for each species, the image of the largest head was resized until its HL was equal to that of the smallest head, and then the silhouettes were superimposed with the smallest on top. For images of the mesosoma, MsL was targeted (ignoring the petiole), and for images of the gaster, GL was used.

#### The head

There are consistent patterns of shape change that hold across many or even most species. As already noted, HL is isometric with BL for all species except *S. geminata* and (more weakly) *S. xyloni* AZ. In contrast, all measures of head width increase more rapidly than BL (Fig. 8a). Within the head, the strength of the positive allometry in all species decreased from the head vertex to the mandible base. The order of R_x_ slopes was thus HW2 (above the eyes, greatest), HW1, HW3 and MB (least, isometric for two species). The effect of these allometries was to change the head from barrel-shaped when small to heart-shaped when large (Fig. 9). With respect to head allometry, *S. geminata* was in a class by itself, as is apparent from the much higher value of the MB allometry and the strongly positive HL allometry (see also volume comparisons below). In most species, relative MB increased modestly with size, or was sometimes isometric as in *S. interrupta, S. megergates, S. xyloni* OK, *S. invicta* (Fig. 8a). However, in *S. geminata*, MB increased almost as rapidly as HW1, leading to a “square” or “blocky-shaped” head in the largest workers (Fig. 8a, Fig. 9).

Because in most species, HL is isometric with BL, and headwidth measurements are mostly allometric to BL, the allometry of headwidths to HL shows similar, although more consistent patterns (Fig. 8e). All species show a similar positive allometry between HL and HW1, HW2 and HW3. The high slope values for HW3/HL and MB/HL confirm that the heads of *S. geminata* become “square” as they become larger. For about half of the other species, MB/HL is isometric or weakly positively allometric with HL. In this circumstance, the higher the slopes of HW1 and HW2, the more heart-shaped the larger heads become (Fig. 8e, 9). Because the width above the eyes grows relatively more rapidly than that below the eyes, the ratio HW2/HW1 is positively allometric for all species, while HW3/HW1 is mostly negative, or isometric (*S. interrupta, S. saevissima*). Once again, *S. geminata* is exceptional in that HW3/HW1 is either isometric or weakly positively allometric in keeping with the “blockiness” of its largest heads.

The relative size of antennae and their parts also show consistent patterns across species (Fig. 8b). The antennae become relatively shorter as BL increases, with *S. amblychila* and *S. xyloni* OK being the extremes. The greatest part of this relative shortening resides in the club (CL/BL), with *S. amblychila* and *S. xyloni* again being the extremes. The scape (SL/BL) is more weakly negatively allometric with the most negative again being *S. amblychila* and *S. xyloni* OK, and *S. gayi* isometric. The flagellum (FL/BL) shows the weakest negative allometry, with 6 species being either isometric or almost so. The flagellum of *S. pythia* is even positively allometric. These patterns are clarified by the relationship of the ratio of each antennal part to the antennal whole (ANT, Fig. 8f). The CL/ANT is consistently negatively allometric to ANT, while FL/ANT is positively allometric and SL/ANT is weakly positive or isometric, depending on the species. In other words, most of the relative reduction of antennal length resides in the club. The flagellum becomes relatively longer while the scape contributes little to the change in relative length.

#### The mesosoma and legs

Mesosoma length (MsL) is isometric with BL (or nearly so for *S. xyloni* AZ) in all species. In contrast, mesosoma height (MsH) increases more rapidly than BL, but is rather variable even within species (e.g. *S. interrupta, S. amblychila, S. richteri*). This allometry can be seen by the larger “hump” of the mesosoma in the largest mesosomas (Fig. 9). This “hump” is not very pronounced in *S. pythia* and *S. gayi* because these species have the smallest size difference between the largest and smallest workers, and thus, not much scope for the expression of this allometry. The trait reaches its greatest expression in *S. geminata* TX & FL, which also has the largest size difference between the largest and smallest workers, and the greatest expression of head shape differences (see above). Fig. 9 suggests that mesosoma shape changes may also independently affect the metathoracic segment and propodeum, that is, the smaller “hump” posterior to the metanotal suture, but these were not measured.

Relative leg length showed variable patterns depending on the species and which leg (Fig. 8c). In 13 species, the first pair of legs (L1) grew more slowly than BL, but for 5 of these some samples were isometric. In *S. pythia*, the first pair of legs became relatively longer in larger workers, whereas in *S. richteri*, they became longer in one sample, and shorter in the other. Leg2 (L2) was isometric with BL in 4 species, positively allometric in 4, and weakly or inconsistently negatively allometric in 5 (Fig. 8c). Leg3 tracked L1 and L2 almost exactly, with the exception of *S. megergates, S. gayi* and *S. xyloni* AZ in which L1 becomes shorter relative to L2 and L3 in larger individuals.

#### The petiole and gaster

Here, we consider the petiole and postpetiole together as the “petiole.” In 11 species, its length (PL) increases more slowly than BL, is isometric or inconsistent in the remaining 4 species. In contrast, its height (PH) increases more rapidly than BL in all 15 species (*S. interrupta* was marginal), resulting in a more “robust” petiole in all (these patterns are not very visible in Fig. 9 because the silhouettes were matched for AL, not PL).

Gaster length (gaster tergite 1, GL) grew more rapidly than BL in 7 species, inconsistently in 3 (*S. amblychila, S. macdonoghi, S. xyloni* OK), isometrically in 4 (*S. invicta, S. geminata* GUA&CR, *S. geminata* TX&FL, *S. gayi*) and less rapidly in 1 (*S. xyloni* AZ). In contrast, gaster width (GW) grew more rapidly than BL in all 15 species, with the highest rates in *S. megergates* followed closely by *S. macdonoghi*, *S. invicta* and *S. gayi*. Using the ratio GW/GL, it is clear that in 12 species, GW increases more rapidly than GL causing gasters of larger workers to appear more “plump” (Fig. 8d, Fig. 9). In 3 species (*S. amblychila, S. interrupta, S. xyloni* AZ), one sample was isometric while the others were positively allometric.

### Allometry of Head, Mesosoma and Gaster Volume

**Figure 10:**
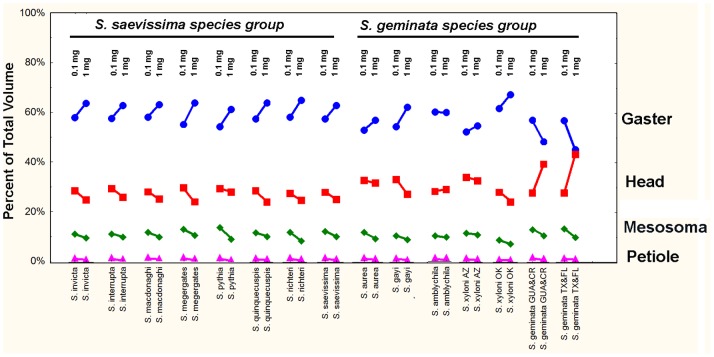
Percent of total body volume making up each body part for workers weighing 0.1 The differences represent the volume change for a 10-fold increase in weight, and were computed from the regressions of WT vs. BL solved for 0.1 and 1.0.

The volume of body components gives a more realistic estimate of allometry of these tagmata because it integrates multiple dimensions such as the width and height. The proportion of the total body volume made up by the head, mesosoma+petiole and gaster was computed from the volume vs. weight regressions. When a body part makes up a changing proportion of the total body, the slope of this regression will deviate significantly from 1.0. These regressions were solved for workers of 0.1 mg and 1.0 mg. As the weight of workers increased from 0.1 mg to 1.0 mg, the relative contribution of each tagma to the total body volume fell into two groups, those in which the gaster increased (13 species; including unchanged in *S. amblychila*), and *S. geminata* in which it decreased (Fig. 10). The increase in relative gaster volume was accompanied by an opposite change in the relative head volume– relative head size, decreased in 10 species, changed little in *S. amblychila, S. aurea* and *S. xyloni*, and increased greatly in *S. geminata*. Increased gaster size was compensated by a decrease in relative mesosoma size and petiole size in all species except *S. geminata* in which it was decreased gaster size that compensated for an increased head size. As dry weight increased from 0.1 mg to 1.0 mg, the gaster increased from 57% to 62% of the total in most species, but decreased from 57% to 47% in the samples of *S. geminata*. At the same time, head volume decreased from 30% to 27% in most species, but increased from 30% to 41% in *S. geminata*. Similarly mesosoma size decreased from 12% to 10% of the total in the first group, and 13% to 10% in *S. geminata*. Petiole size decreased in all species similarly, but the decrease was less than 1% of total volume. Overall then, the relative head size in *S. geminata* increased greatly at the expense of the gaster, mesosoma and petiole, whereas in all other species, relative gaster size increased (or remained unchanged) while the remaining tagmata decreased (but 3 species showed little change in relative head size). In Fig. 9, the difference in the percent value between workers of 1.0 mg and 0.1 mg is shown next to each tagma. Note that shape change is not consistently associated with these values.

### Regressions of Slopes vs. Intercepts

**Figure 11:**
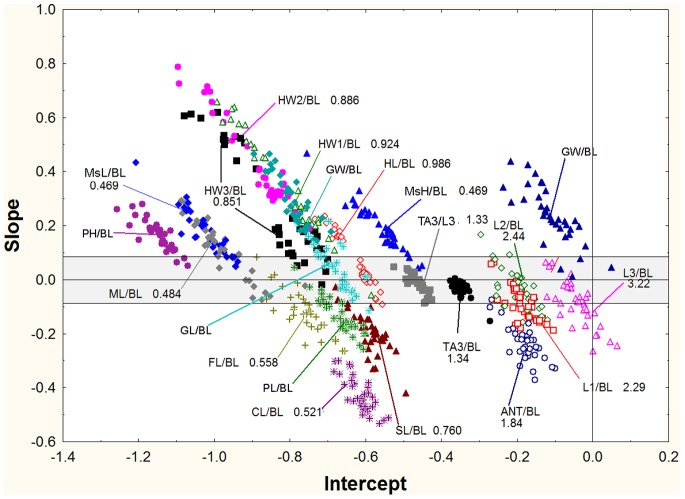
The slopes of the regressions of R_BL_ vs. BL in relationship to their intercepts, marked for the ratio’s identity. The grey zone indicates isometric relationships, positive allometries have larger slopes and negative smaller ones. For all regressions, the greater the slope, the lower the intercept. See text for a discussion of these patterns.

**Figure 12:**
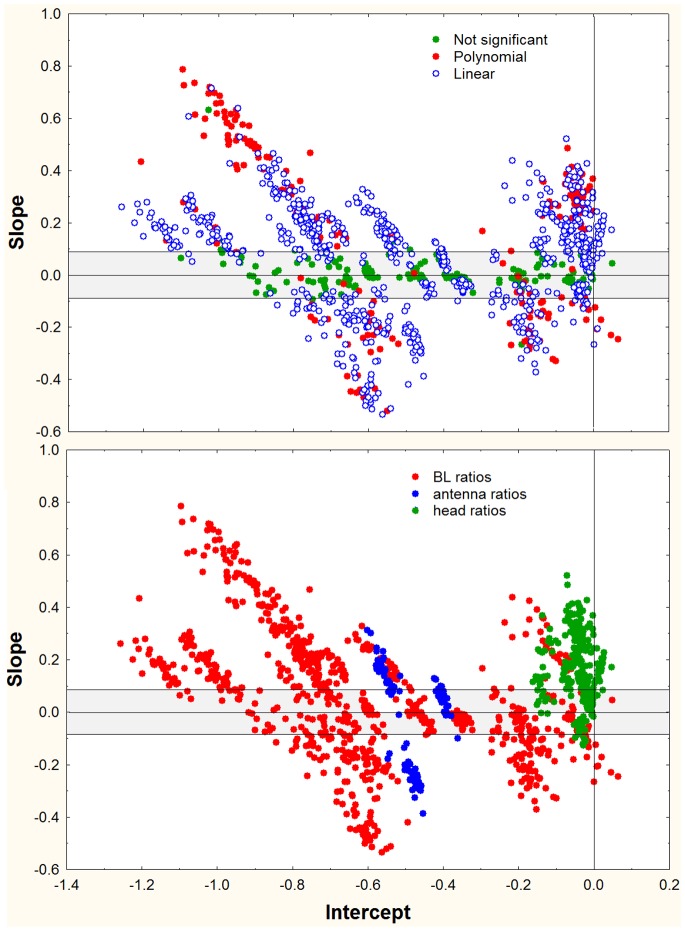
The same plots as in Fig. 11, but marked for whether the best fit was linear (upper panel, blue) or polynomial (upper panel, red). In the lower panel, the points are coded for ratios to BL, ratios to ANT or ratios to head measures.

**Figure 13:**
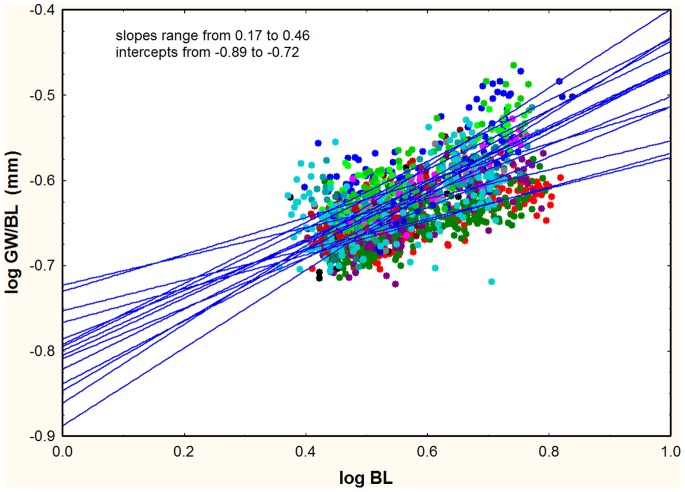
An example using GL/BL illustrating the rotation of plots around a zone within the data cloud. It is this rotation that leads to the negative relationship between the slopes and intercepts seen in Figs. 11 and 12. Each color and line represents one of the species.

For the log-log regressions of R_BL_ vs. BL (e.g. HW2/BL vs. BL), there was a negative relationship between the slope of the regression and its intercept (Fig. 11, Fig. 12). This relationship existed whether the slope was positive, negative or not significantly different from zero, and suggests that the regression lines for each ratio passed through a common point (or fairly narrow zone), in other words, that they appeared to rotate around this common point or zone. An increase in slope therefore necessitated a decrease in the intercept if the common point was a positive value of log BL. Mathematically, the intercept of each regression is the value of the log-ratio when BL is zero, or in linear terms, when the ant is 1 mm in body length. Because the various dimensions are all less than BL, the ratios are all less than 1.0 and their logs negative. The point or zone of rotation must be a positive value of log BL, that is, BL>1. Only then will the relationship between the intercept and slope be negative. If the rotation were around a negative value of log BL, then the relationship between the intercept and slopes would be positive. A multiple plot of all samples for each measure, or for the ratio to BL, showed that the rotation zone in the great majority of cases is within the cloud of data points. A typical example is shown in Fig. 13, using GW/BL vs. BL (but the pattern is similar for others as well). There is no single crossing point for any of these plots, but in view of normal variability, one would not expect such a point. In general, the rotation of plots within the data cloud can be interpreted as a trade-off between the rate of shape change with size (slope) and the “starting body (or imaginal disc) size”. The “scaling factor” of Tobler and Nijhout [35] is the intercept in this study, and their “allometric coefficient” is the slope. Body parts that change shape most rapidly with size tend to be in workers with relatively small body length when compared across species.

### Polynomial Regressions

**Figure 14:**
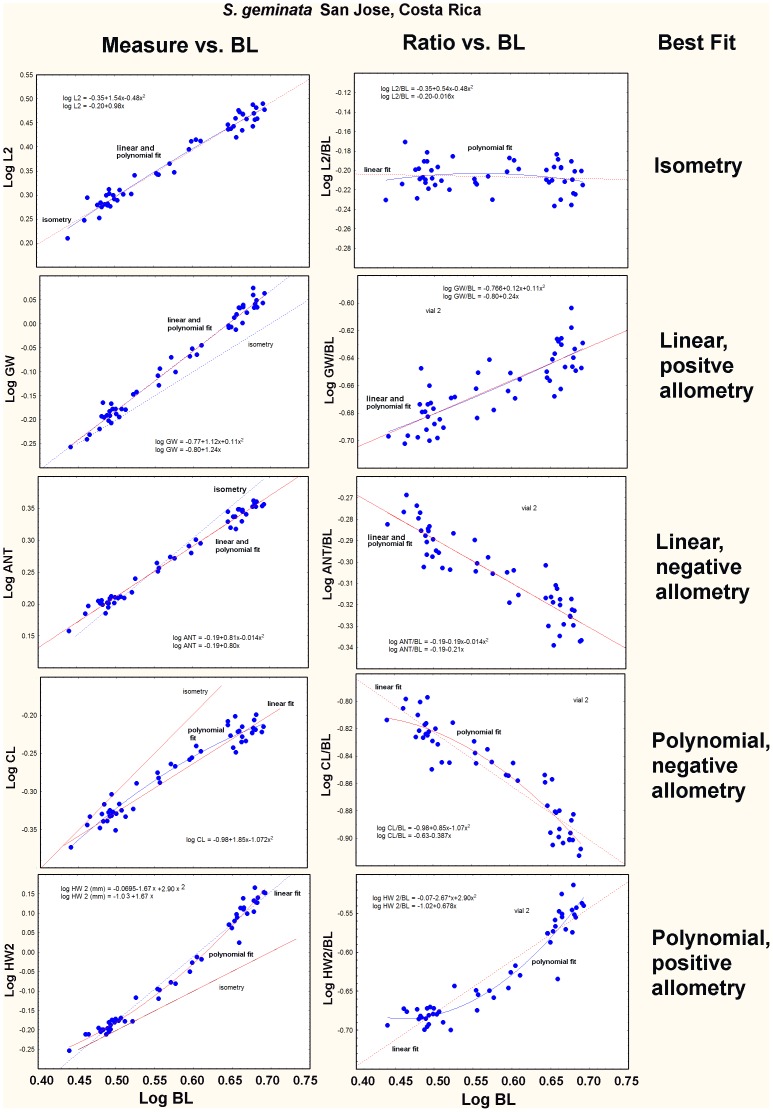
Examples of the range of regression fits plotted as Measure vs. BL (left column) and Measure/BL vs. BL (right column). As the allometry becomes stronger, the plots become curved such that a polynomial improves the fit. The plots illustrate isometry, positive and negative linear allometry and positive and negative polynomial allometry.

The coded symbols in Fig. 12a show that whereas the best fit for the majority (65%) of log-log regressions was linear, about 20% were fit better (based on analysis of residuals) by a polynomial function, whether in regressions of the measures themselves vs. BL, or their ratios vs. BL. Using examples from a single colony of *S. geminata,* the left column of Fig. 14 shows the measures plotted against BL, and the right column shows the R_BL_ of each measure plotted vs. BL. The line of isometry is shown in the left graphs. In the right ones, the line of isometry is horizontal, i.e. has a slope of zero. The possible relationships and best fits, range from isometry (panel A), to identical linear and polynomial fits (panels B, C), to examples in which a polynomial clearly fits the data better (panels D, E), as determined by analysis of residuals.

Linear fits were always a reasonable approximation of the allometry, but an improvement of fit with a polynomial has biological meaning because it reveals that the relative growth “constants” are not constant over the range of sizes. In morphological terms, this means that as BL increases, a focal dimension increases (or decreases, if the allometry is negative) ever more rapidly, exaggerating the allometry beyond the power function. This can be seen as an upward curvature in positively allometric relationships and a downward in the negative ones (Fig. 14D, E). This represents a deviation from standard allometry in which an increase in BL by a factor of x is associated with a fixed increase by a factor y in the focal dimension across the entire range of size. A similar proportion of each type of measurement– head, mesosoma and gaster – was fit better by a polynomial, suggesting that strong allometry can occur in any body part.

**Figure 15:**
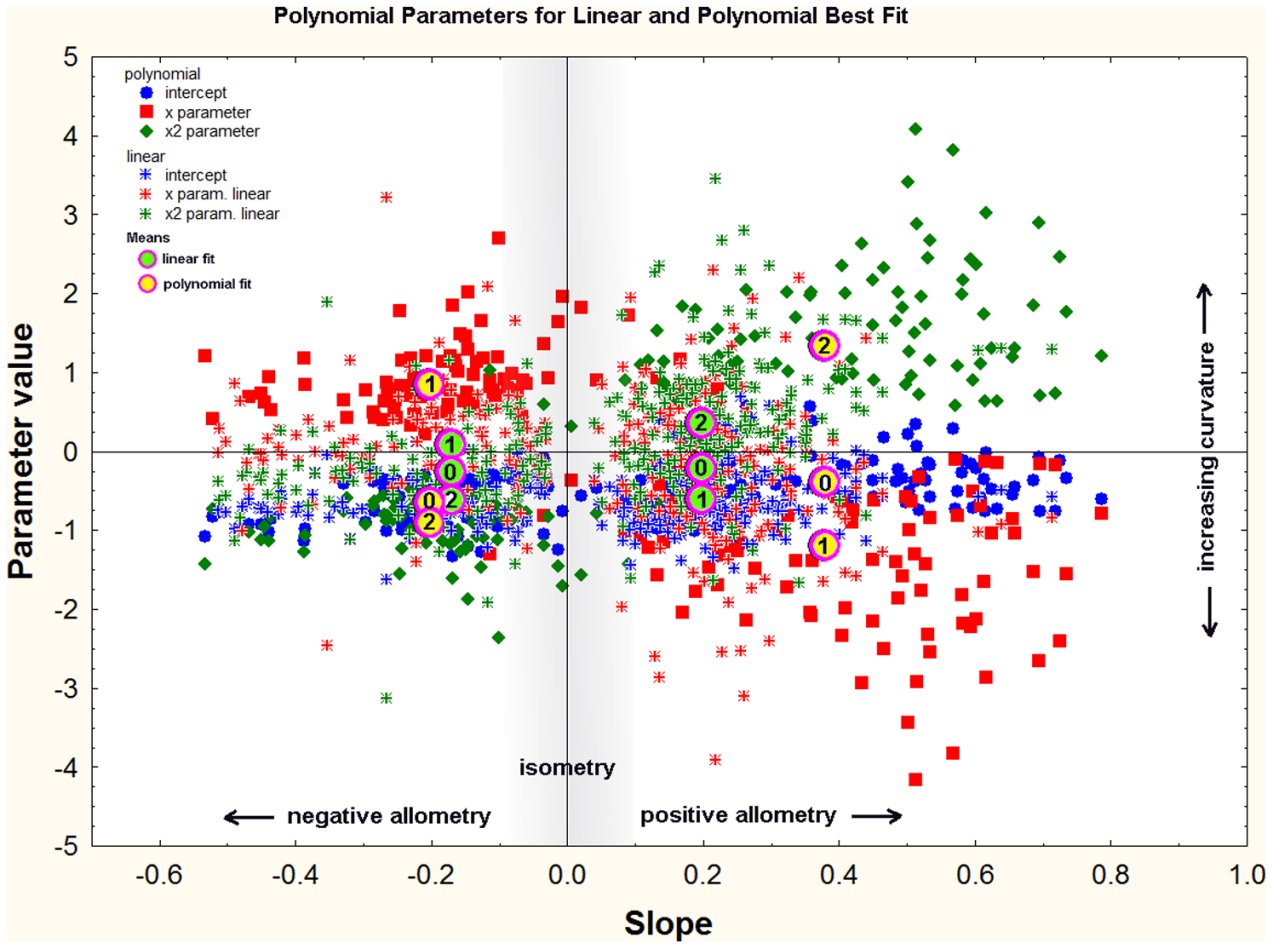
A comparison of the polynomial parameters for regressions best fit by linear or polynomial equations. The “+” symbols show the relationships with a linear best fit, and the solid symbols those with a polynomial best fit. Red symbols code for the x-parameter and green the x^2^ parameter. Polynomial best fits are strongly associated with extreme values of the linear slopes, that is, with strong positive or negative allometry, but the effect was stronger for positive allometry. The larger the x^2^-parameter and the smaller the x-parameter, the more curved was the allometric relationship.

For further analysis, all relationships that were not isometric were fit with both a linear and polynomial regression, and showed that a polynomial was more likely to improve the fit as the allometry became stronger, that is, as the slope of the linear equation became more positive or negative. In Fig. 15, all of the x and x^2^ parameters of the polynomial fit are plotted in relation to the slopes of a linear fit of the same data. Filled symbols show those in which a polynomial gave a better fit, and “plusses” show those in which a polynomial did not improve the fit of a linear regression. As the steepness of the linear slope increases, that is, as the allometry becomes stronger (either positively or negatively), it becomes more likely that the relationship becomes more curved and a polynomial will provide a better fit. An increase in curvature is indicated by an increase in the x^2^ parameter and a decrease in the x parameter. Clearly, fitting a polynomial to a linear relationship produces an equation in which the x^2^ parameter is zero or nearly zero, and the x parameter is identical (or nearly so) to the slope of a linear fit. Fig. 15 shows that most relationships with slopes greater than about 0.4 were fit better by a polynomial, i.e. were positively curved. The mean x^2^ parameter for positively allometric polynomial best-fits was 1.3, but for linear-best-fits it was only 0.25 (Fig. 15, means). The corresponding x parameters were −1.2 for polynomial best fits and −0.5 for linear. This large difference in parameter values was not seen in negative allometries (polynomial x^2^ = −0.85; linear x^2^ = −0.70; polynomial x = 0.85; linear x = 0.15).

**Figure 16:**
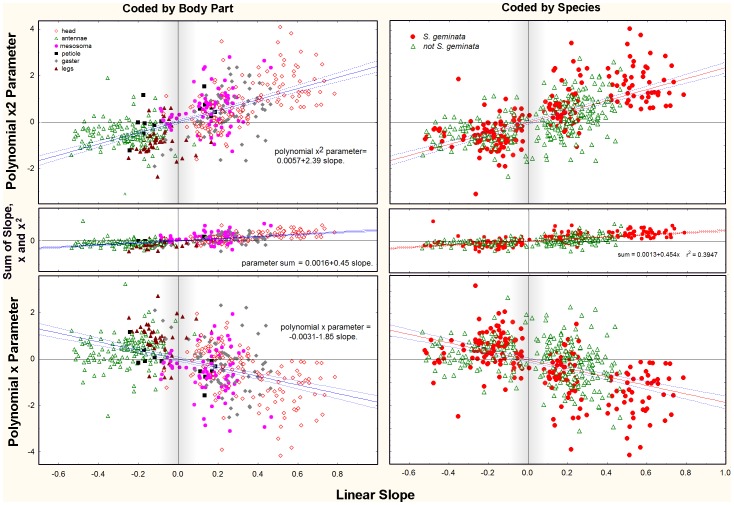
The same data as Fig. 15, but with parameters plotted in separate panels. The more extreme the linear slope, the more curved the allometric relationship is likely to be, and the more likely that it will be best fit by a polynomial relationship. This can be seen by comparing the top and bottom panels: as linear slope increases, the x^2^ parameter increases (positive allometry) or decreases (negative allometry) at the expense of the x-parameter. Their opposite trends define the curvature of the allometry. The center panels show the sums of the absolute values of the x^2^ and x parameters, illustrating that the compensation is not complete. In this figure, the symbols are coded by body part (left panels) or by *S. geminata* and other species (right panels). Curved, polynomial best fits are more common in *S. geminata*, and are more likely to affect the head, antennae and gaster.

On average, the quadratic parameter increased at the expense of the linear parameter (Fig. 16) (the intercept was not strongly affected, and is not shown in Fig. 16). As the slope increased, the x^2^ parameter (curvature) increased only slightly faster than the x parameter decreased. As a result, the sum of the linear slope, the x^2^ parameter and the x parameter was near zero, although it increased slowly as slope increased (middle panel, Fig. 15; sum = 0.0016+0.45(slope)).

Exaggerated allometry is not evenly distributed across body parts (Fig. 16 left; Table 5). Dimensions of the head and antennae were more strongly affected than other dimensions. Of the 175 polynomial best fits, 37% were of the head and 26% of the antennae. At the other extreme, the mesosoma, petiole and gaster were all less than 10%. This contrasts with linear best fits of which the head made up 24% while all others fell between 13 and 20%. Isometry was rarest for the antennae and petiole, and most common for the head and legs.

**Table 5:** Frequency of fit type for *S. geminata* and all other spp.

Species	linearbest fit		Polynomialbest fit		Not significant	
	N	Percent	N	Percent	N	Percent
*S. geminata*	144	50	116	40	29	10
other spp.	314	65	59	12	110	23
Total	458		175		139	

Total regressions = 769.

The exaggerated allometries were not evenly distributed across species (Fig. 16, right), but were especially characteristic of *S. geminata* (Table 5; Fig. 9). Significantly curved relationships were more frequent in *S. geminata* than other species (Table 5). A polynomial was the best fit in 40% of *S. geminata* regressions, but only 12% of other species. Linear fits were best for 50% of *S. geminata*, but 65% of other species. Finally, whereas only 10% of *S.geminata* regressions were isometric, 23% of other species were.

Combining species and body parts (Table 6) makes clear that the high proportion of polynomial best fits for *S. geminata* were more often of head dimensions. Of the 113 polynomial best fits for *S. geminata*, 47% were of the head, 20% of the antennae or legs, and fewer than 4% of any other body part. Of the 105 linear best fits for *S. geminata*, only 10% were of the head, 27% of the antennae and about 23% for each of the other parts. Finally, *S. geminata* body parts were rarely isometric, with legs and gaster showing the highest occurrence of isometry.

**Table 6:** Frequency of fit type by body part and species.

fit of log regression	body part	N	percent	N for other spp.	N for S. geminata	Totals
linear	head	110	24%	90	10	458
linear	antennae	87	19%	58	29	
linear	mesosoma	64	14%	40	24	
linear	petiole	61	13%	37	24	
linear	gaster	73	16%	48	25	
linear	legs	63	14%	41	22	
polynomial	head	64	37%	11	53	175
polynomial	antennae	46	26%	23	23	
polynomial	mesosoma	14	8%	5	9	
polynomial	petiole	3	2%	1	2	
polynomial	gaster	11	6%	7	4	
polynomial	legs	34	18%	12	22	
not significant	head	36	26%	31	5	139
not significant	antennae	7	5%	7	0	
not significant	mesosoma	27	19%	21	6	
not significant	petiole	6	4%	6	0	
not significant	gaster	21	15%	11	10	
not significant	legs	42	30%	34	8	
					Total	769

## Discussion

### Overview

**Figure 17:**
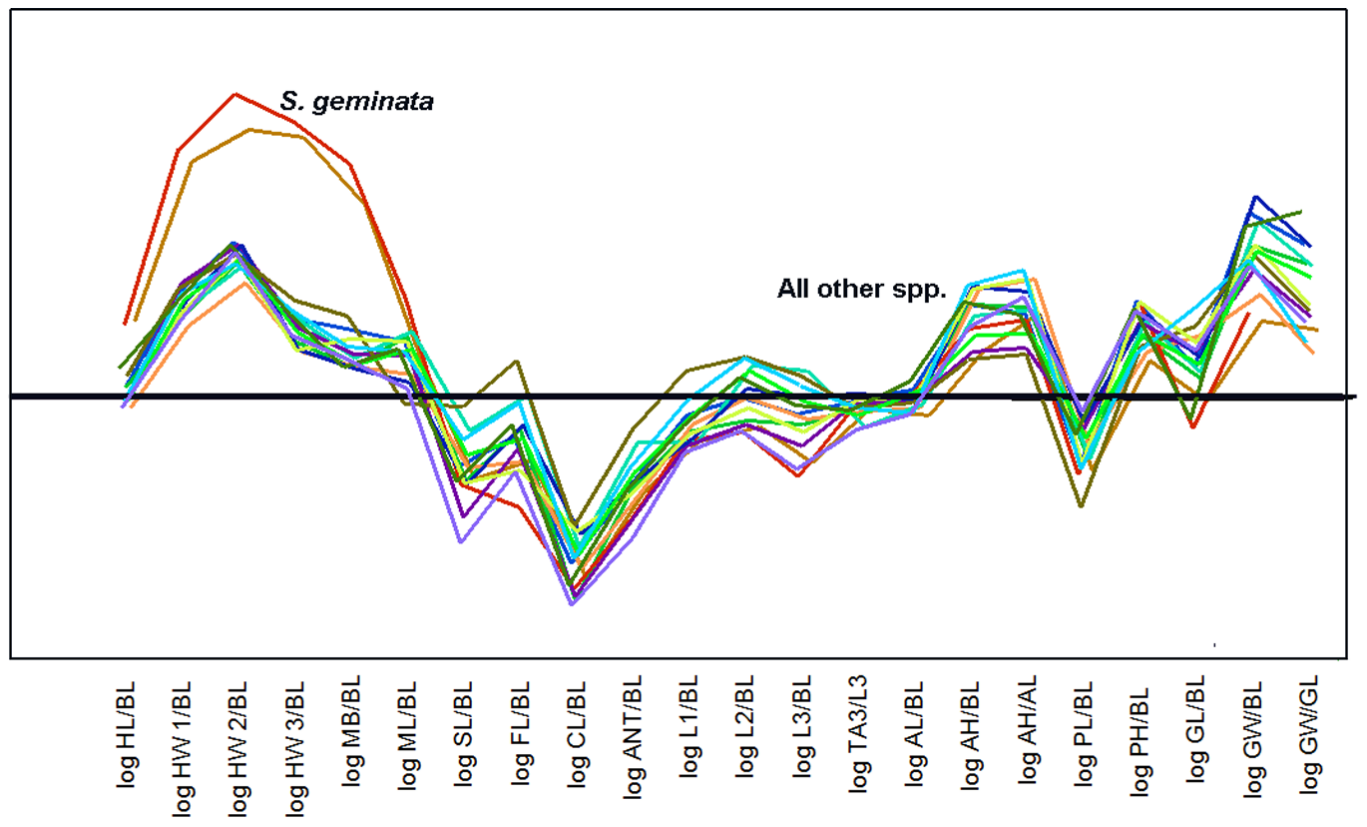
The mean allometry patterns for all *Solenopsis* species superimposed on the same axes. Size-related changes in shape follow similar patterns in all species except *S. geminata* in which the head allometries are much stronger. Other species are not identified in this plot. Antennal allometries (excluding the club), mesosomal height, and gaster width are more variable than other allometries.

The patterns of allometry across the 15 species of *Solenopsis* suggest that all but *S. geminata* follow very similar rules that generate shape as size increases (Fig. 17). Summarizing first all species except *S. geminata*, the head and gaster are subject to the strongest allometry, with relative head and mesosoma volumes decreasing with worker size and gaster increasing. Within the head, shape gradually changes from barrel shaped in small workers to heart shaped in large ones, as already reported in *S. invicta* [36]. The smoothness of this transition suggests that width growth rate increases on a gradient from the mandible insertions to the head vertex. At the same time, the antennae become relatively shorter, mostly on account of a decrease of the relative size of the club. Legs were variable with both weakly positive and negative allometries, even within species. Relative gaster volume increased with worker size primarily because gaster width increased faster than worker size. It is reasonable to suppose that relatively larger gasters are associated with increased alimentary functions, either crop retention of liquid food or storage of fat in fat bodies [32]. This is also consistent with the positive allometry between body length and weight^−3^ (Fig. 4).

### Evolution of Allometries

The patterns suggest that evolution cannot select on several of these traits independently of each other, that they are linked into character suites through allometric growth during ontogeny. The relative increase in one dimension or volume is accompanied by a decrease in another. The most striking feature of allometries in *Solenopsis* is how monotonous they are– they vary by degrees, so that some allometric constants are larger and some smaller, but their patterns across species are remarkably similar (Fig. 17; with the exception of *S. geminata*, see below). Variability is stronger for the antennal flagellum and scape, for mesosoma height and for gaster width. Head allometries are particularly similar among species.

The limited scope for evolving shape changes seems to be a common feature in polymorphic and dimorphic ants. In a sense, the major mechanism through which ants can change the shapes of their parts is through changing their size. These parts arise through the growth of imaginal discs whose complex dimensions grow at different relative rates from their inception as a small clump of cells to their final adult forms. The modest direct control over shape is probably responsible mostly for the variability of the allometric parameters (Fig. 17). In a study of 231 species of *Pheidole*, differences in shape were similarly constrained by size [15] in the sense that most of the shape differences were associated with size differences. However, this study is not strictly comparable to the current one on *Solenopsis*, because the authors measured only a single minor and major worker from each species. Thus, the described allometries apply across species, but not within species (for which allometric parameters are lacking).

The rotation of the regressions within the data clouds can be interpreted as growth constants acting on imaginal discs of a size that does not vary as much across species as do the growth constants, ie.e the size of the imaginal discs represents the “starting size” of the structure whose subsequent growth rate may be altered through evolution. However, because regressions often cross within the data cloud, an alternate interpretation could be that discs may evolve to a smaller “starting size,” in which case their dimensions grow more (or less) rapidly (i.e. are more strongly allometric). Of course, both could be operating simultaneously. These questions could be resolved by measuring imaginal discs across species.

### Ontogeny and Variability of Allometries

The attributes of ant colonies arise during sociogenesis [37]. For species with continuous polymorphisms, the proportion of large workers gradually increases, increasing the mean worker size [31], [37], and it has rarely been questioned whether the body size-shape relationships were constant. The substantial variability of allometric “constants” among localities within *Solenopsis* species, and even among colonies within the same locality suggests that growth rules are not completely constant within species, and that regression parameters may be of limited usefulness for distinguishing among species. At the very least, multiple colonies and localities of each species would have to be compared statistically. The variation among colonies within localities was not large, but suggests that the parameters are influenced by factors other than species identity. These factors could include colony size, season, nutritional state, growth rate, queen number and social form, but these were unknown for most of the samples in this study, though most probably they came from “mature” colonies. Reversing the point of view, the variation patterns emphasize that most of the species of *Solenopsis* follow similar growth rules at the species level, and these can be somewhat modified by social, environmental and life history conditions. The mechanism that links allometry with these factors is unknown, but could include nutrition, brood density and physical factors.

Variability of allometric “constants” has been documented several times. In the North American population of *S. invicta*, the allometry varies significantly between polygyne and monogyne social forms, and between small and large monogyne colonies [38]: head and antennae shape changed more rapidly with size in large monogyne colonies, while mesosoma height was actually negatively allometric in small monogyne colonies and isometric in polygyne and large monogyne. Porter and Tschinkel [39] and Tschinkel [31], [32] found that mean worker size of *S. invicta* increased as colony size increased, implying, according to Araujo and Tschinkel’s [38] findings, that the allometric constants may also have been affected. Allometry varied significantly among colonies of *Paraponera clavata* [40]. Kenne et al. [41] found that the headwidth-antenna allometry of *Myrmicaria opaciventris* was negative for workers from single-queen incipient colonies, isometric from multiple-queen incipient colonies and positive from larger colonies. Huang and Wheeler [29] showed that the allometry of workers from laboratory-reared colonies of *Pheidole obtusospinosa* differed significantly from field colonies, but workers from two field colonies did not differ from one another.

It was also striking that *S. geminata* follows dramatically different growth rules, or more specifically, that its allometries were much stronger. This was especially true for the head, in which HL and all HWs increased so strongly with worker size that it was the only species in which the relative head volume increased and gaster volume decreased. Even the allometries within the head are different. Whereas the mandible base is isometric or nearly so in all other species, in *S. geminata* it is strongly positively allometric to BL and HL (Fig. 7a), creating the huge, blocky head that makes *S. geminata* so easy to identify in the field.

*S. geminata’s* extreme divergence from the other species of *Solenopsis* begs for explanation. The extreme size and shape of the head is not characteristic of the other species in the clade to which *S. geminata* belongs, for example, *S. aurea* and *S. amblychila*, whose allometry is in line with the other *Solenopsis* species. It seems possible that *S. geminata’s* allometry is linked to the harvesting and processing of seeds. Several studies have reported that seeds, especially small seeds, are a substantial fraction of its diet [42]. *S. geminata* even collected the seeds of rice [43], sorghum [44] and cactus [45]. Tennant and Porter [46] found that *S. geminata* in Texas collected eight times as many seeds as did *S. invicta*. In laboratory colonies of *S. geminata*, Wilson [47] noted that the sole behavior of the large major workers was milling seeds, a function that would be aided by the large mandibles with associated muscles. If the frequency of seeds in the diets of most other *Solenopsis* species were low, the seed-milling origin of the extreme allometry of *S. geminata* would be supported. Unfortunately, there is little information on the frequency of seeds in the diets of other *Solenopsis* species. A more vexing question is, considering the uniformity of the allometric rules in all other *Solenopsis* species, through what trick did evolution change the developmental rules of *S. geminata* so greatly? It is unlikely that the large head is an ancestral trait, as it is shared with no other fire ants.

### Non-linear Allometries

A substantial fraction of the allometries were not well-fit by a simple log-log relationship, but were fit better by a polynomial log-log regression in which the magnitude of the x^2^ parameter described the deviation from linearity. Such non-linearity is common in allometric analyses and was discussed by both Feener et al. [33] and Knell [48]. Knell recommended testing the adequacy of a linear fit using standard statistical methods, including an analysis of residuals and goodness of fit tests, and recommended concluding non-linearity only if these tests justify it. Feener et al. [33] noted that the scaling exponents for a polynomial fit represent the instantaneous slope of the regression equation for a given body size, and provided methods for estimating the standard error. For *Solenopsis*, positive non-linear allometries were generally concave, whereas negative allometries were convex. This means that the allometric effects either lagged at small body sizes, or accelerated at large, but the present data do not distinguish between these states. Given that the allometries are continuous (as they appear to be), non-linearity could result from competition between imaginal discs undergoing rapid growth during the pupal period [14].

### Measures of the Whole

In this study, the sum of lengths served as the measure of the whole. Issues associated with the use of weight as the measure of the whole are discussed above. Other approaches to “the whole” include the estimation of overall body size as the first principle component in a PCA, and then estimating bivariate allometric parameters against the dimensions that are isometric with this first principle component ([34]; also references therein). Whether any of these methods for estimating size of the whole are superior is essentially a philosophical question, as it depends on exactly what the question is. An advantage of the method used in my study is that the comparisons are more easily imagined and understood, and that is surely of value.

### Possible Functions of Shape Changes

Most shape changes are probably driven by mechanical requirements. The shape changes in the head seem likely to be related to the necessity for containing relatively larger mandibular muscles so that larger workers have relatively greater strength. The increasingly humped mesosoma may result from the need to accommodate larger cervical and coxal muscles, and similar considerations probably apply to the more humped petiole. Leg length is probably related to running speed and perhaps strength via mechanical advantage.

### Allometry and Phylogeny

The range of maximum major worker sizes in *Solenopsis* was quite variable. According to the phylogenetic analysis of Pitts et al. [49], there is a trend within the *saevissima* group of fire ants to evolve larger major worker size. Such trends are not apparent in my data– major worker sizes are not congruent with Pitts et al.’s phylogeny. However, Pitts et al. [49] used mostly 2-state characters (0, 1) for their analyses, rather than size measurements. If one accepts the Pitts et al. [43] phylogeny along with my size estimates, and takes into account that maximum and minimum sizes both vary (often independently), then worker sizes do not map well on the phylogeny. Size appears to be a rather labile character.

## Supporting Information

Appendix S1 **The basic measurements, listed by species, collection locality, colony number (vial) and worker number.** See text for abbreviations. This is a tab-delimited file, each row ending in a carriage return.
(DOC)
