# Supplementary material for: The Morphometry of Solenopsis Fire Ants
Source: PLoS One. 2013 Nov 19;8(11):e79559. doi: 10.1371/journal.pone.0079559 (PMC3834273; doi:10.1371/journal.pone.0079559)
Supplement: Appendix S1 — The basic measurements, listed by species, collection locality, colony number (vial) and worker number. See text for abbreviations. This is a tab-delimited file, each row ending in a carriage return. (DOC) [file pone.0079559.s001.doc]

Appendix S1. The basic measurements, listed by species, collection locality, colony number (vial) and worker number. See text for abbreviations. This is a tab-delimited file, each row ending in a carriage return.

Species Vial ID locality Ant # WT (mg, dry) HL (mm) HW 1 (mm) HW 2 (mm) HW 3 (mm) MB (mm) ML (mm) SL (mm) FL (mm) CL (mm) FE 1 (mm) TB 1 (mm) TA 1 (mm) FE 2 (mm) TB 2 (mm) TA 2 (mm) FE 3 (mm) TB 3 (mm) TA 3 (mm) MsL (mm) MsH (mm) MsL 1 (mm) MsL 2 (mm) PL (mm) PH (1) (mm) GL (mm) GW (mm) BL

S. geminata 2 San Jose, Costa Rica 1 0.600 1.233 1.247 1.229 1.202 0.942 0.660 0.862 0.650 0.564 1.076 0.774 0.755 0.932 0.746 0.950 1.278 0.901 1.461 1.559 0.598 0.987 0.660 0.848 0.414 0.892 1.046 4.53

S. geminata 2 San Jose, Costa Rica 2 0.840 1.361 1.402 1.420 1.352 1.099 0.731 0.941 0.719 0.609 1.185 0.808 0.860 1.150 0.775 1.077 1.372 0.958 1.589 1.640 0.614 1.017 0.730 0.895 0.466 1.031 1.157 4.93

S. geminata 2 San Jose, Costa Rica 3 0.820 1.406 1.406 1.424 1.351 1.089 0.600 0.940 0.716 0.600 1.208 0.903 0.884 1.200 0.811 1.080 1.399 0.965 1.616 1.634 0.609 1.029 0.692 0.874 0.470 0.990 1.105 4.90

S. geminata 2 San Jose, Costa Rica 4 0.700 1.312 1.350 1.340 1.295 1.002 0.670 0.941 0.707 0.604 1.160 0.865 0.839 1.157 0.814 1.058 1.379 0.961 1.575 1.655 0.598 1.013 0.710 0.871 0.457 0.973 1.119 4.81

S. geminata 2 San Jose, Costa Rica 5 0.530 1.216 1.204 1.176 1.153 0.906 0.569 0.895 0.619 0.620 1.107 0.769 0.828 1.061 0.740 0.992 1.267 0.915 1.453 1.543 0.573 0.979 0.651 0.788 0.411 0.878 0.991 4.43

S. geminata 2 San Jose, Costa Rica 6 0.730 1.323 1.368 1.345 1.318 1.033 0.571 0.952 0.707 0.622 1.050 0.831 0.833 1.007 0.821 1.035 1.353 0.970 1.588 1.618 0.583 1.024 0.678 0.895 0.457 0.956 1.098 4.79

S. geminata 2 San Jose, Costa Rica 7 0.690 1.253 1.279 1.288 1.243 0.945 0.633 0.927 0.707 0.591 1.197 0.848 0.864 1.118 0.761 1.054 1.168 0.911 1.534 1.616 0.594 1.002 0.691 0.845 0.434 0.909 1.094 4.62

S. geminata 2 San Jose, Costa Rica 8 0.660 1.296 1.314 1.300 1.269 0.974 0.665 0.902 0.700 0.609 1.160 0.828 0.869 1.101 0.793 1.043 1.340 0.970 1.542 1.557 0.618 0.963 0.648 0.846 0.442 0.923 1.083 4.62

S. geminata 2 San Jose, Costa Rica 9 0.760 1.318 1.355 1.382 1.323 1.042 0.623 0.937 0.724 0.632 1.196 0.853 0.868 1.030 0.827 1.016 1.371 0.934 1.561 1.645 0.610 1.022 0.721 0.874 0.460 0.984 1.082 4.82

S. geminata 2 San Jose, Costa Rica 10 0.580 1.225 1.235 1.203 1.190 0.924 0.655 0.908 0.691 0.572 1.103 0.802 0.834 0.977 0.778 1.016 1.254 0.900 1.475 1.568 0.578 0.938 0.708 0.775 0.414 0.931 1.028 4.50

S. geminata 2 San Jose, Costa Rica 11 0.520 1.205 1.164 1.154 1.128 0.894 0.632 0.868 0.625 0.593 1.055 0.791 0.812 1.020 0.741 0.977 1.248 0.888 1.440 1.487 0.557 0.920 0.644 0.813 0.411 0.958 0.984 4.46

S. geminata 2 San Jose, Costa Rica 12 0.630 1.374 1.464 1.464 1.397 1.017 0.741 0.954 0.740 0.607 1.068 0.861 0.899 1.150 0.843 0.957 1.381 0.984 1.669 1.600 0.588 0.988 0.705 0.826 0.462 0.978 1.079 4.78

S. geminata 2 San Jose, Costa Rica 13 0.860 1.225 1.244 1.270 1.194 1.1 0.665 0.925 0.727 0.599 1.199 0.878 0.844 1.171 0.803 1.098 1.319 0.956 1.564 1.682 0.645 1.114 0.745 0.843 0.504 1.014 1.187 4.76

S. geminata 2 San Jose, Costa Rica 14 0.590 1.305 1.360 1.376 1.315 0.94 0.635 0.877 0.676 0.582 1.095 0.809 0.828 0.971 0.737 1.008 1.279 0.893 1.499 1.559 0.523 0.960 0.680 0.782 0.405 0.969 1.004 4.61

S. geminata 2 San Jose, Costa Rica 15 0.680 1.013 1.053 1.058 1.018 1.008 0.533 0.931 0.699 0.601 1.153 0.793 0.875 1.119 0.794 1.077 1.337 0.974 1.118 1.612 0.584 1.015 0.660 0.882 0.431 1.056 1.079 4.56

S. geminata 2 San Jose, Costa Rica 16 0.640 1.262 1.290 1.297 1.236 0.96 0.685 0.931 0.697 0.602 1.017 0.819 0.881 1.116 0.810 1.040 1.349 0.967 1.539 1.530 0.555 0.993 0.626 0.848 0.434 0.939 1.078 4.58

S. geminata 2 San Jose, Costa Rica 17 0.600 1.263 1.284 1.256 1.239 0.953 0.512 0.910 0.696 0.584 1.176 0.779 0.841 1.093 0.774 1.003 1.357 0.933 1.551 1.599 0.633 0.999 0.677 0.865 0.429 0.946 1.055 4.67

S. geminata 2 San Jose, Costa Rica 18 0.530 1.171 1.193 1.176 1.148 0.897 0.649 0.919 0.679 0.612 0.990 0.828 0.636 0.957 0.800 0.976 1.294 0.970 1.336 1.537 0.515 0.920 0.696 0.747 0.412 0.973 0.982 4.43

S. geminata 2 San Jose, Costa Rica 19 0.580 1.260 1.251 1.251 1.201 0.934 0.534 0.873 0.670 0.629 0.973 0.779 0.846 1.077 0.766 1.035 1.292 0.897 1.293 1.531 0.583 0.956 0.656 0.843 0.371 0.891 0.972 4.52

S. geminata 2 San Jose, Costa Rica 20 0.710 1.323 1.354 1.355 1.263 0.981 0.688 0.934 0.719 0.598 1.168 0.842 0.825 1.135 0.785 0.849 1.401 0.981 1.546 1.645 0.631 1.036 0.721 0.837 0.455 0.957 1.148 4.76

S. geminata 2 San Jose, Costa Rica 21 0.380 1.056 0.975 0.957 0.930 0.737 0.566 0.785 0.622 0.566 1.013 0.665 0.749 0.967 0.682 0.934 1.159 0.882 1.594 1.383 0.477 0.836 0.598 0.747 0.383 0.893 0.902 4.08

S. geminata 2 San Jose, Costa Rica 22 0.360 1.039 0.998 0.971 0.949 0.757 0.546 0.823 0.600 0.575 0.895 0.745 0.631 0.962 0.683 0.954 1.170 0.847 1.344 1.377 0.488 0.836 0.601 0.783 0.358 0.825 0.862 4.02

S. geminata 2 San Jose, Costa Rica 23 0.320 1.004 0.923 0.890 0.887 0.726 0.528 0.799 0.602 0.552 1.051 0.685 0.494 0.964 0.637 0.879 1.018 0.779 1.393 1.350 0.448 0.816 0.565 0.717 0.347 0.870 0.854 3.94

S. geminata 2 San Jose, Costa Rica 24 0.340 1.025 0.957 0.939 0.903 0.738 0.527 0.752 0.596 0.555 0.996 0.696 0.744 0.962 0.661 0.957 1.037 0.792 1.370 1.393 0.529 0.851 0.629 0.749 0.373 0.802 0.886 3.97

S. geminata 2 San Jose, Costa Rica 25 0.260 0.930 0.840 0.804 0.799 0.628 0.477 0.751 0.555 0.531 0.888 0.625 0.686 0.776 0.593 0.844 1.066 0.787 1.349 1.271 0.439 0.755 0.592 0.631 0.337 0.749 0.779 3.58

S. geminata 2 San Jose, Costa Rica 26 0.230 0.911 0.814 0.799 0.791 0.63 0.450 0.744 0.544 0.515 0.871 0.623 0.677 0.783 0.587 0.829 1.027 0.741 1.095 1.217 0.435 0.754 0.518 0.681 0.341 0.795 0.805 3.60

S. geminata 2 San Jose, Costa Rica 27 0.270 0.938 0.849 0.829 0.822 0.634 0.363 0.761 0.567 0.540 0.797 0.640 0.735 0.788 0.604 0.830 1.077 0.775 0.977 1.281 0.460 0.788 0.534 0.681 0.332 0.876 0.793 3.78

S. geminata 2 San Jose, Costa Rica 28 0.280 0.962 0.867 0.835 0.826 0.653 0.487 0.769 0.563 0.544 0.912 0.669 0.724 0.871 0.573 0.873 1.083 0.757 1.259 1.266 0.436 0.768 0.546 0.724 0.356 0.773 0.851 3.73

S. geminata 2 San Jose, Costa Rica 29 0.220 0.895 0.786 0.759 0.755 0.598 0.394 0.725 0.536 0.522 0.830 0.606 0.638 0.810 0.634 0.757 0.998 0.706 1.184 1.179 0.405 0.734 0.511 0.769 0.321 0.749 0.744 3.59

S. geminata 2 San Jose, Costa Rica 30 0.210 0.859 0.791 0.764 0.755 0.603 0.442 0.702 0.521 0.513 0.848 0.596 0.670 0.798 0.570 0.823 0.987 0.714 1.186 1.191 0.450 0.724 0.531 0.637 0.306 0.672 0.721 3.36

S. geminata 2 San Jose, Costa Rica 31 0.190 0.804 0.704 0.664 0.664 0.56 0.430 0.658 0.519 0.475 0.797 0.568 0.640 0.739 0.541 0.723 0.837 0.666 1.081 1.107 0.368 0.677 0.484 0.677 0.298 0.737 0.712 3.32

S. geminata 2 San Jose, Costa Rica 32 0.170 0.831 0.691 0.677 0.655 0.556 0.418 0.658 0.495 0.474 0.763 0.546 0.636 0.783 0.549 0.712 0.963 0.640 1.123 1.108 0.385 0.670 0.467 0.634 0.249 0.640 0.664 3.21

S. geminata 2 San Jose, Costa Rica 33 0.160 0.799 0.682 0.668 0.655 0.524 0.393 0.650 0.508 0.471 0.812 0.523 0.625 0.640 0.524 0.725 0.910 0.667 1.081 1.064 0.391 0.640 0.462 0.577 0.286 0.686 0.664 3.13

S. geminata 2 San Jose, Costa Rica 34 0.180 0.799 0.695 0.673 0.664 0.551 0.401 0.664 0.490 0.469 0.754 0.542 0.591 0.731 0.524 0.702 0.899 0.624 1.053 1.104 0.388 0.679 0.475 0.582 0.270 0.676 0.649 3.16

S. geminata 2 San Jose, Costa Rica 35 0.160 0.786 0.655 0.632 0.628 0.524 0.377 0.647 0.470 0.497 0.759 0.540 0.577 0.775 0.534 0.698 0.891 0.626 1.069 1.070 0.399 0.640 0.481 0.620 0.278 0.639 0.621 3.11

S. geminata 2 San Jose, Costa Rica 36 0.130 0.769 0.660 0.625 0.625 0.505 0.368 0.635 0.466 0.465 0.713 0.514 0.596 0.725 0.489 0.685 0.782 0.622 0.997 1.051 0.353 0.638 0.448 0.665 0.279 0.614 0.657 3.10

S. geminata 2 San Jose, Costa Rica 37 0.150 0.790 0.692 0.660 0.659 0.523 0.394 0.632 0.482 0.473 0.783 0.528 0.627 0.657 0.520 0.736 0.909 0.644 1.141 1.076 0.384 0.648 0.469 0.631 0.274 0.604 0.644 3.10

S. geminata 2 San Jose, Costa Rica 38 0.140 0.804 0.692 0.660 0.656 0.538 0.388 0.651 0.493 0.446 0.781 0.590 0.568 0.738 0.576 0.677 0.925 0.650 1.081 1.078 0.351 0.639 0.475 0.611 0.285 0.660 0.664 3.15

S. geminata 2 San Jose, Costa Rica 39 0.140 0.759 0.677 0.632 0.637 0.517 0.390 0.645 0.502 0.458 0.817 0.533 0.607 0.738 0.513 0.673 0.902 0.630 1.021 1.098 0.357 0.642 0.505 0.556 0.259 0.625 0.684 3.04

S. geminata 2 San Jose, Costa Rica 40 0.130 0.750 0.637 0.614 0.592 0.487 0.365 0.619 0.455 0.458 0.726 0.512 0.588 0.689 0.480 0.739 0.740 0.571 1.049 0.990 0.350 0.627 0.412 0.628 0.266 0.707 0.644 3.07

S. geminata 2 San Jose, Costa Rica 41 0.170 0.776 0.697 0.667 0.666 0.528 0.384 0.660 0.475 0.483 0.754 0.532 0.593 0.723 0.518 0.704 0.875 0.675 1.068 1.109 0.384 0.659 0.502 0.621 0.295 0.684 0.639 3.19

S. geminata 2 San Jose, Costa Rica 42 0.150 0.825 0.687 0.663 0.655 0.549 0.394 0.675 0.478 0.465 0.776 0.526 0.567 0.745 0.579 0.676 0.922 0.641 1.093 1.098 0.394 0.656 0.479 0.637 0.265 0.689 0.662 3.25

S. geminata 2 San Jose, Costa Rica 43 0.160 0.771 0.690 0.649 0.658 0.533 0.382 0.648 0.504 0.466 0.680 0.563 0.611 0.725 0.528 0.796 0.917 0.619 1.142 1.058 0.364 0.665 0.476 0.601 0.283 0.684 0.681 3.11

S. geminata 2 San Jose, Costa Rica 44 0.140 0.759 0.664 0.624 0.610 0.497 0.369 0.648 0.473 0.468 0.726 0.561 0.633 0.695 0.514 0.577 0.867 0.658 1.185 1.043 0.384 0.630 0.443 0.599 0.264 0.625 0.641 3.02

S. geminata 2 San Jose, Costa Rica 45 0.140 0.746 0.660 0.637 0.651 0.52 0.390 0.646 0.476 0.470 0.765 0.538 0.525 0.699 0.518 0.774 0.899 0.642 0.967 1.052 0.425 0.642 0.472 0.583 0.275 0.706 0.627 3.09

S. geminata 2 San Jose, Costa Rica 46 0.120 0.770 0.679 0.632 0.656 0.525 0.386 0.629 0.469 0.482 0.719 0.502 0.610 0.689 0.508 0.687 0.884 0.624 1.059 1.064 0.398 0.659 0.457 0.588 0.247 0.625 0.638 3.05

S. geminata 2 San Jose, Costa Rica 47 0.130 0.750 0.629 0.615 0.579 0.474 0.363 0.601 0.476 0.453 0.703 0.495 0.430 0.597 0.488 0.682 0.766 0.583 0.909 0.991 0.305 0.606 0.416 0.583 0.240 0.570 0.574 2.89

S. geminata 2 San Jose, Costa Rica 48 0.150 0.732 0.643 0.615 0.625 0.524 0.386 0.624 0.484 0.464 0.668 0.524 0.554 0.718 0.520 0.732 0.874 0.589 1.166 1.045 0.365 0.627 0.444 0.564 0.273 0.578 0.587 2.92

S. geminata 2 San Jose, Costa Rica 49 0.140 0.755 0.678 0.638 0.651 0.51 0.377 0.652 0.501 0.449 0.663 0.517 0.584 0.735 0.487 0.679 0.888 0.627 1.034 1.051 0.353 0.638 0.447 0.605 0.267 0.597 0.603 3.01

S. geminata 2 San Jose, Costa Rica 50 0.090 0.709 0.576 0.557 0.550 0.455 0.331 0.571 0.444 0.423 0.606 0.490 0.427 0.658 0.453 0.510 0.779 0.572 0.972 0.961 0.308 0.574 0.429 0.516 0.238 0.570 0.554 2.76

S. aurea 5 6 m.WSW, Rodeo, NM 1 0.720 1.311 1.294 1.293 1.190 0.977 0.659 0.846 0.683 0.562 1.107 0.781 0.784 1.079 0.731 0.923 1.262 0.892 1.428 1.633 0.675 1.052 0.684 0.951 0.475 1.064 1.329 4.96

S. aurea 5 6 m.WSW, Rodeo, NM 2 0.820 1.321 1.395 1.419 1.256 1.048 0.708 0.849 0.717 0.578 1.177 0.819 0.836 1.113 0.767 1.038 1.283 0.934 1.558 1.721 0.774 1.147 0.730 0.935 0.491 1.196 1.346 5.17

S. aurea 5 6 m.WSW, Rodeo, NM 3 0.750 1.354 1.354 1.391 1.256 1.002 0.696 0.863 0.688 0.560 1.153 0.772 0.901 1.125 0.733 0.996 1.260 0.935 1.531 1.702 0.639 1.071 0.714 0.984 0.467 1.137 1.317 5.18

S. aurea 5 6 m.WSW, Rodeo, NM 4 0.770 1.358 1.367 1.395 1.242 1.048 0.697 0.866 0.718 0.588 1.111 0.777 0.867 1.105 0.793 1.059 1.289 0.935 1.604 1.671 0.623 1.089 0.695 1.081 0.510 1.212 1.383 5.32

S. aurea 5 6 m.WSW, Rodeo, NM 5 0.710 1.336 1.349 1.353 1.279 0.974 0.723 0.880 0.713 0.563 1.152 0.767 0.818 1.082 0.784 0.993 1.163 0.926 1.566 1.678 0.624 1.076 0.684 1.000 0.491 1.140 1.416 5.15

S. aurea 5 6 m.WSW, Rodeo, NM 6 0.710 1.298 1.312 1.340 1.191 0.951 0.680 0.858 0.710 0.578 1.085 0.793 0.837 1.071 0.710 1.012 1.247 0.889 1.461 1.670 0.676 1.085 0.686 0.933 0.476 1.129 1.271 5.03

S. aurea 5 6 m.WSW, Rodeo, NM 7 0.720 1.340 1.340 1.354 1.224 0.956 0.676 0.857 0.702 0.572 1.115 0.806 0.881 1.063 0.780 1.011 1.225 0.929 1.512 1.694 0.543 1.071 0.721 0.987 0.492 1.107 1.403 5.13

S. aurea 5 6 m.WSW, Rodeo, NM 8 0.750 1.293 1.312 1.345 1.206 0.934 0.662 0.832 0.697 0.564 1.112 0.774 0.815 1.073 0.726 0.978 1.232 0.905 1.498 1.624 0.611 1.053 0.676 0.988 0.484 1.044 1.218 4.95

S. aurea 5 6 m.WSW, Rodeo, NM 9 0.740 1.307 1.339 1.321 1.214 0.938 0.643 0.852 0.707 0.577 1.113 0.770 0.856 1.088 0.762 0.970 1.239 0.925 1.514 1.709 0.652 1.109 0.706 0.939 0.494 1.124 1.288 5.08

S. aurea 5 6 m.WSW, Rodeo, NM 10 0.690 1.281 1.315 1.339 1.186 0.947 0.662 0.856 0.698 0.576 0.968 0.765 0.846 1.045 0.731 1.027 1.204 0.886 1.506 1.630 0.663 1.044 0.664 0.885 0.469 1.028 1.231 4.82

S. aurea 5 6 m.WSW, Rodeo, NM 11 0.770 1.340 1.372 1.386 1.223 1.007 0.707 0.880 0.732 0.584 1.134 0.806 0.888 0.966 0.760 1.061 1.257 0.945 1.604 1.694 0.660 1.082 0.716 0.877 0.490 1.105 1.340 5.02

S. aurea 5 6 m.WSW, Rodeo, NM 12 0.600 1.259 1.255 1.245 1.135 0.886 0.625 0.845 0.648 0.565 1.077 0.742 0.797 1.027 0.725 0.939 1.188 0.882 1.423 1.525 0.549 0.990 0.626 0.868 0.435 0.992 1.133 4.64

S. aurea 5 6 m.WSW, Rodeo, NM 13 0.500 1.174 1.161 1.161 1.078 0.87 0.612 0.797 0.661 0.546 1.009 0.720 0.779 0.983 0.690 0.908 1.156 0.848 1.455 1.468 0.562 0.929 0.625 0.863 0.422 0.936 1.106 4.44

S. aurea 5 6 m.WSW, Rodeo, NM 14 0.350 1.070 1.019 0.981 0.935 0.772 0.540 0.734 0.573 0.534 0.903 0.633 0.732 0.855 0.614 0.834 1.035 0.744 1.300 1.294 0.447 0.820 0.532 0.777 0.354 0.860 0.907 4.00

S. aurea 5 6 m.WSW, Rodeo, NM 15 0.360 1.075 1.010 0.991 0.964 0.768 0.527 0.730 0.574 0.519 0.938 0.622 0.730 0.868 0.642 0.813 1.036 0.716 1.304 1.304 0.580 0.807 0.544 0.759 0.352 0.845 0.996 3.98

S. aurea 5 6 m.WSW, Rodeo, NM 16 0.300 0.991 0.936 0.926 0.907 0.722 0.514 0.716 0.556 0.509 0.886 0.628 0.692 0.852 0.579 0.800 1.003 0.722 1.223 1.239 0.439 0.793 0.499 0.695 0.338 0.766 0.950 3.69

S. aurea 5 6 m.WSW, Rodeo, NM 17 0.230 0.866 0.782 0.758 0.749 0.626 0.542 0.670 0.516 0.463 0.810 0.530 0.623 0.754 0.533 0.710 0.898 0.635 1.124 1.108 0.412 0.714 0.451 0.693 0.300 0.698 0.785 3.36

S. aurea 5 6 m.WSW, Rodeo, NM 18 0.140 0.814 0.712 0.675 0.689 0.565 0.385 0.605 0.460 0.457 0.730 0.488 0.566 0.677 0.447 0.668 0.809 0.576 1.005 1.019 0.361 0.647 0.415 0.616 0.277 0.617 0.696 3.07

S. aurea 5 6 m.WSW, Rodeo, NM 19 0.160 0.786 0.684 0.665 0.665 0.579 0.418 0.594 0.448 0.462 0.637 0.462 0.526 0.653 0.474 0.638 0.799 0.575 0.933 0.989 0.360 0.630 0.406 0.607 0.258 0.660 0.618 3.04

S. aurea 5 6 m.WSW, Rodeo, NM 20 0.100 0.750 0.648 0.620 0.621 0.543 0.386 0.578 0.440 0.413 0.674 0.487 0.501 0.608 0.453 0.578 0.738 0.541 0.908 0.924 0.340 0.580 0.381 0.579 0.246 0.580 0.636 2.83

S. aurea 5 6 m.WSW, Rodeo, NM 21 0.160 0.741 0.658 0.630 0.659 0.521 0.381 0.573 0.434 0.422 0.662 0.468 0.527 0.640 0.439 0.627 0.775 0.556 0.930 0.948 0.331 0.612 0.379 0.567 0.246 0.623 0.606 2.88

S. aurea 5 6 m.WSW, Rodeo, NM 22 0.120 0.735 0.628 0.623 0.609 0.529 0.365 0.582 0.426 0.431 0.654 0.483 0.507 0.609 0.460 0.578 0.754 0.541 0.917 0.924 0.333 0.589 0.377 0.540 0.226 0.612 0.631 2.81

S. aurea 5 6 m.WSW, Rodeo, NM 23 0.120 0.750 0.652 0.619 0.629 0.567 0.368 0.570 0.422 0.432 0.658 0.444 0.527 0.615 0.435 0.624 0.757 0.546 0.929 0.924 0.330 0.582 0.384 0.577 0.238 0.603 0.596 2.85

S. aurea 5 6 m.WSW, Rodeo, NM 24 0.120 0.740 0.629 0.620 0.625 0.533 0.340 0.560 0.427 0.415 0.656 0.478 0.513 0.606 0.446 0.600 0.738 0.528 0.909 0.924 0.336 0.575 0.379 0.574 0.221 0.587 0.647 2.82

S. aurea 5 6 m.WSW, Rodeo, NM 25 0.150 0.805 0.689 0.647 0.684 0.556 0.382 0.600 0.450 0.450 0.728 0.505 0.573 0.671 0.477 0.645 0.813 0.562 0.999 1.009 0.337 0.628 0.423 0.571 0.252 0.645 0.648 3.03

S. aurea 5 6 m.WSW, Rodeo, NM 26 0.120 0.712 0.609 0.586 0.605 0.478 0.358 0.541 0.392 0.419 0.628 0.458 0.484 0.593 0.451 0.539 0.710 0.561 0.828 0.903 0.360 0.558 0.367 0.587 0.246 0.589 0.566 2.79

S. aurea 5 6 m.WSW, Rodeo, NM 27 0.150 0.728 0.625 0.592 0.601 0.493 0.312 0.566 0.421 0.420 0.641 0.433 0.539 0.598 0.409 0.598 0.755 0.531 0.921 0.925 0.325 0.558 0.388 0.551 0.245 0.548 0.639 2.75

S. aurea 5 6 m.WSW, Rodeo, NM 28 0.110 0.707 0.614 0.595 0.563 0.469 0.351 0.543 0.392 0.407 0.651 0.446 0.520 0.594 0.426 0.562 0.710 0.495 0.880 0.901 0.322 0.553 0.375 0.518 0.221 0.557 0.557 2.68

S. aurea 5 6 m.WSW, Rodeo, NM 29 0.110 0.741 0.640 0.618 0.608 0.532 0.375 0.554 0.423 0.416 0.649 0.447 0.537 0.603 0.437 0.595 0.742 0.565 0.895 0.906 0.322 0.563 0.378 0.553 0.250 0.569 0.637 2.77

S. aurea 5 6 m.WSW, Rodeo, NM 30 0.100 0.744 0.624 0.563 0.563 0.469 0.356 0.549 0.412 0.418 0.655 0.444 0.501 0.606 0.442 0.557 0.745 0.511 0.910 0.878 0.358 0.559 0.360 0.584 0.248 0.540 0.567 2.75

S. aurea 5 6 m.WSW, Rodeo, NM 31 0.110 0.721 0.642 0.609 0.623 0.501 0.375 0.550 0.407 0.426 0.623 0.450 0.509 0.603 0.450 0.585 0.723 0.530 0.909 0.936 0.350 0.583 0.381 0.566 0.256 0.632 0.599 2.86

S. aurea 5 6 m.WSW, Rodeo, NM 32 0.110 0.736 0.629 0.595 0.616 0.489 0.330 0.563 0.400 0.419 0.654 0.462 0.534 0.605 0.440 0.563 0.711 0.536 0.932 0.923 0.289 0.570 0.385 0.546 0.229 0.534 0.612 2.74

S. aurea 5 6 m.WSW, Rodeo, NM 33 0.120 0.745 0.629 0.591 0.633 0.501 0.295 0.560 0.407 0.412 0.672 0.461 0.522 0.626 0.414 0.627 0.742 0.522 0.941 0.941 0.333 0.591 0.400 0.561 0.247 0.609 0.585 2.86

S. aurea 5 6 m.WSW, Rodeo, NM 34 0.120 0.717 0.591 0.568 0.582 0.511 0.350 0.559 0.415 0.418 0.632 0.450 0.533 0.528 0.423 0.545 0.722 0.509 0.932 0.907 0.301 0.567 0.374 0.539 0.225 0.520 0.607 2.68

S. aurea 5 6 m.WSW, Rodeo, NM 35 0.120 0.721 0.623 0.605 0.609 0.496 0.348 0.541 0.406 0.407 0.635 0.449 0.523 0.573 0.429 0.565 0.721 0.524 0.875 0.903 0.307 0.582 0.358 0.541 0.243 0.557 0.580 2.72

S. aurea 5 6 m.WSW, Rodeo, NM 36 0.120 0.721 0.629 0.605 0.619 0.519 0.366 0.581 0.432 0.410 0.646 0.468 0.504 0.626 0.413 0.598 0.734 0.523 0.931 0.923 0.360 0.580 0.380 0.550 0.239 0.615 0.675 2.81

S. aurea 5 6 m.WSW, Rodeo, NM 37 0.120 0.758 0.652 0.647 0.619 0.506 0.377 0.584 0.428 0.437 0.673 0.445 0.537 0.625 0.431 0.614 0.772 0.537 0.942 0.947 0.322 0.588 0.398 0.567 0.227 0.614 0.668 2.89

S. aurea 5 6 m.WSW, Rodeo, NM 38 0.110 0.680 0.579 0.570 0.558 0.47 0.289 0.520 0.396 0.386 0.597 0.383 0.487 0.538 0.378 0.549 0.659 0.474 0.852 0.867 0.276 0.541 0.351 0.500 0.235 0.499 0.531 2.55

S. aurea 5 6 m.WSW, Rodeo, NM 39 0.100 0.759 0.657 0.628 0.629 0.516 0.362 0.552 0.421 0.417 0.645 0.476 0.532 0.593 0.406 0.618 0.729 0.526 0.927 0.945 0.400 0.590 0.384 0.529 0.250 0.567 0.650 2.80

S. aurea 5 6 m.WSW, Rodeo, NM 40 0.120 0.698 0.619 0.591 0.605 0.487 0.354 0.562 0.407 0.412 0.651 0.421 0.505 0.599 0.413 0.575 0.664 0.510 0.901 0.903 0.299 0.568 0.378 0.516 0.239 0.564 0.613 2.68

S. geminata 6 Guatemala City, Zone 10 1 0.850 1.314 1.332 1.288 1.277 1.016 0.987 0.951 0.755 0.618 1.127 0.862 0.820 1.205 0.785 1.137 1.412 0.979 1.584 1.688 0.625 1.057 0.719 0.880 0.470 1.014 1.132 4.90

S. geminata 6 Guatemala City, Zone 10 2 0.850 1.317 1.347 1.328 1.283 0.987 0.860 0.969 0.760 0.614 1.128 0.900 0.911 1.080 0.810 1.179 1.450 1.010 1.699 1.730 0.640 1.068 0.754 0.895 0.485 1.084 1.251 5.03

S. geminata 6 Guatemala City, Zone 10 3 0.690 1.222 1.217 1.190 1.171 0.953 0.858 0.929 0.723 0.619 1.070 0.826 0.880 1.168 0.796 1.065 1.252 0.964 1.659 1.571 0.569 0.978 0.695 0.846 0.444 1.087 1.127 4.73

S. geminata 6 Guatemala City, Zone 10 4 0.660 1.239 1.237 1.219 1.182 0.953 0.766 0.912 0.700 0.618 1.094 0.819 0.906 1.129 0.799 1.105 1.370 0.949 1.565 1.608 0.582 0.985 0.692 0.832 0.433 0.982 1.084 4.66

S. geminata 6 Guatemala City, Zone 10 5 0.670 1.206 1.245 1.193 1.168 0.898 0.778 0.930 0.682 0.634 1.054 0.760 0.816 1.170 0.747 1.040 1.397 0.933 1.304 1.593 0.614 1.002 0.714 0.883 0.456 1.026 1.152 4.71

S. geminata 6 Guatemala City, Zone 10 6 0.610 1.173 1.160 1.101 1.105 0.872 0.669 0.897 0.668 0.610 0.993 0.780 0.897 1.077 0.768 1.010 1.299 0.949 1.508 1.434 0.547 0.938 0.599 0.821 0.403 1.037 1.016 4.46

S. geminata 6 Guatemala City, Zone 10 7 0.560 1.131 1.092 1.046 1.030 0.816 0.593 0.879 0.676 0.582 1.117 0.794 0.788 1.078 0.738 1.013 1.298 0.899 1.508 1.503 0.522 0.916 0.673 0.826 0.410 0.986 1.032 4.45

S. geminata 6 Guatemala City, Zone 10 8 0.650 1.208 1.237 1.200 1.156 0.916 0.798 0.924 0.688 0.632 1.054 0.798 0.856 1.117 0.788 1.064 1.357 0.959 1.573 1.578 0.587 0.958 0.701 0.922 0.444 0.981 1.102 4.69

S. geminata 6 Guatemala City, Zone 10 9 0.710 1.199 1.190 1.139 1.111 0.889 0.732 0.902 0.692 0.620 0.998 0.736 0.862 1.109 0.765 1.064 1.323 0.928 1.589 1.565 0.567 0.961 0.703 0.867 0.428 1.005 1.070 4.64

S. geminata 6 Guatemala City, Zone 10 10 0.400 1.110 1.044 0.999 0.987 0.803 0.724 0.874 0.644 0.588 0.973 0.721 0.795 0.986 0.691 0.992 1.264 0.857 1.507 1.451 0.529 0.883 0.620 0.786 0.386 0.958 0.973 4.31

S. geminata 6 Guatemala City, Zone 10 11 0.550 1.153 1.110 1.065 1.051 0.872 0.976 0.884 0.630 0.574 1.076 0.736 0.814 0.980 0.708 0.959 1.275 0.897 1.479 1.485 0.551 0.917 0.647 0.831 0.405 0.881 0.982 4.35

S. geminata 6 Guatemala City, Zone 10 12 0.360 0.976 0.929 0.883 0.883 0.668 0.477 0.791 0.582 0.538 0.912 0.675 0.681 0.918 0.609 0.896 1.130 0.807 1.348 1.295 0.454 0.796 0.536 0.753 0.342 0.784 0.861 3.81

S. geminata 6 Guatemala City, Zone 10 13 0.500 1.085 1.021 0.965 0.954 0.803 0.752 0.855 0.644 0.609 1.065 0.724 0.806 1.031 0.678 0.984 1.260 0.859 1.474 1.463 0.523 0.883 0.658 0.771 0.398 0.948 1.005 4.27

S. geminata 6 Guatemala City, Zone 10 14 0.370 0.994 0.937 0.882 0.900 0.719 0.724 0.819 0.625 0.562 1.001 0.717 0.818 0.969 0.630 0.924 1.203 0.802 1.436 1.368 0.486 0.823 0.601 0.717 0.375 0.830 0.923 3.91

S. geminata 6 Guatemala City, Zone 10 15 0.380 1.051 0.967 0.924 0.909 0.718 0.599 0.827 0.623 0.556 1.026 0.686 0.807 0.925 0.654 0.938 1.217 0.827 1.394 1.410 0.541 0.865 0.615 0.737 0.383 0.877 0.909 4.08

S. geminata 6 Guatemala City, Zone 10 16 0.440 1.063 0.990 0.944 0.948 0.767 0.676 0.862 0.635 0.583 1.079 0.751 0.784 1.018 0.698 0.953 1.230 0.907 1.464 1.392 0.522 0.865 0.581 0.802 0.378 0.864 0.960 4.12

S. geminata 6 Guatemala City, Zone 10 17 0.370 1.045 1.008 0.953 0.953 0.745 0.689 0.849 0.651 0.565 1.063 0.742 0.781 0.948 0.700 0.966 1.096 0.828 1.500 1.420 0.546 0.876 0.631 0.737 0.400 0.927 1.144 4.13

S. geminata 6 Guatemala City, Zone 10 18 0.420 1.036 0.973 0.929 0.932 0.789 0.954 0.842 0.631 0.584 1.054 0.693 0.821 1.021 0.706 0.977 1.232 0.842 1.486 1.420 0.519 0.875 0.614 0.768 0.377 0.908 0.959 4.13

S. geminata 6 Guatemala City, Zone 10 19 0.440 1.031 0.963 0.927 0.926 0.726 0.640 0.849 0.607 0.592 0.938 0.687 0.804 1.011 0.688 0.965 1.203 0.864 1.435 1.419 0.490 0.854 0.637 0.771 0.374 0.893 0.960 4.11

S. geminata 6 Guatemala City, Zone 10 20 0.360 0.993 0.937 0.898 0.884 0.695 0.707 0.797 0.612 0.548 1.009 0.736 0.737 0.967 0.669 0.885 1.172 0.824 1.416 1.341 0.478 0.822 0.568 0.767 0.355 0.865 0.870 3.97

S. geminata 6 Guatemala City, Zone 10 21 0.290 0.959 0.877 0.823 0.833 0.649 0.602 0.796 0.575 0.555 1.044 0.655 0.765 0.831 0.654 0.934 1.151 0.828 1.388 1.331 0.468 0.819 0.568 0.733 0.362 0.806 0.891 3.83

S. geminata 6 Guatemala City, Zone 10 22 0.270 0.925 0.860 0.823 0.823 0.665 0.559 0.769 0.578 0.541 0.881 0.663 0.724 0.918 0.632 0.898 0.980 0.800 1.345 1.287 0.430 0.786 0.541 0.730 0.336 0.760 0.826 3.70

S. geminata 6 Guatemala City, Zone 10 23 0.280 0.963 0.868 0.827 0.834 0.671 0.555 0.784 0.598 0.566 0.957 0.638 0.753 0.922 0.616 0.916 1.115 0.820 1.371 1.307 0.463 0.797 0.567 0.792 0.346 0.918 0.858 3.98

S. geminata 6 Guatemala City, Zone 10 24 0.370 1.012 0.915 0.865 0.878 0.73 0.697 0.814 0.611 0.570 0.910 0.708 0.780 0.947 0.665 0.931 1.186 0.830 1.410 1.355 0.492 0.832 0.603 0.748 0.372 0.813 0.887 3.93

S. geminata 6 Guatemala City, Zone 10 25 0.330 0.940 0.875 0.820 0.842 0.64 0.647 0.788 0.564 0.548 0.970 0.704 0.734 0.914 0.634 0.900 1.118 0.832 1.353 1.317 0.511 0.800 0.568 0.699 0.331 0.798 0.843 3.75

S. geminata 6 Guatemala City, Zone 10 26 0.430 1.072 1.011 0.969 0.967 0.78 0.727 0.848 0.630 0.569 0.968 0.758 0.769 0.926 0.696 0.972 1.236 0.912 1.459 1.437 0.541 0.878 0.632 0.792 0.381 0.938 0.965 4.24

S. geminata 6 Guatemala City, Zone 10 27 0.330 0.970 0.897 0.853 0.880 0.717 0.734 0.769 0.605 0.542 0.961 0.685 0.732 0.853 0.690 0.859 1.118 0.815 1.347 1.372 0.488 0.819 0.619 0.732 0.347 0.794 0.847 3.87

S. geminata 6 Guatemala City, Zone 10 28 0.320 0.985 0.869 0.814 0.832 0.703 0.663 0.780 0.576 0.574 0.882 0.702 0.775 0.922 0.653 0.826 1.146 0.824 1.380 1.369 0.478 0.822 0.607 0.726 0.365 0.860 0.855 3.94

S. geminata 6 Guatemala City, Zone 10 29 0.280 0.908 0.822 0.779 0.800 0.644 0.612 0.764 0.551 0.541 0.955 0.680 0.731 0.889 0.660 0.897 1.117 0.779 1.355 1.270 0.416 0.773 0.539 0.714 0.339 0.905 0.865 3.80

S. geminata 6 Guatemala City, Zone 10 30 0.330 0.944 0.864 0.819 0.832 0.659 0.588 0.773 0.605 0.536 0.970 0.692 0.757 0.898 0.649 0.916 1.131 0.807 1.341 1.290 0.501 0.801 0.553 0.734 0.340 0.812 0.867 3.78

S. geminata 6 Guatemala City, Zone 10 31 0.300 0.922 0.896 0.840 0.845 0.651 0.553 0.782 0.605 0.517 0.871 0.666 0.722 0.852 0.659 0.890 1.131 0.814 1.368 1.335 0.489 0.809 0.579 0.701 0.349 0.824 0.857 3.78

S. geminata 6 Guatemala City, Zone 10 32 0.300 0.936 0.856 0.805 0.826 0.681 0.568 0.770 0.590 0.541 0.842 0.668 0.700 0.912 0.603 0.901 1.095 0.797 1.295 1.272 0.439 0.774 0.546 0.691 0.348 0.769 0.826 3.67

S. geminata 6 Guatemala City, Zone 10 33 0.290 0.899 0.832 0.797 0.797 0.639 0.545 0.755 0.614 0.517 0.835 0.674 0.702 0.889 0.599 0.882 1.096 0.780 1.297 1.264 0.430 0.782 0.522 0.730 0.323 0.763 0.804 3.66

S. geminata 6 Guatemala City, Zone 10 34 0.260 0.875 0.825 0.794 0.811 0.686 0.824 0.750 0.567 0.527 0.895 0.654 0.694 0.882 0.609 0.871 1.074 0.726 1.312 1.232 0.419 0.756 0.533 0.685 0.328 0.754 0.794 3.55

S. geminata 6 Guatemala City, Zone 10 35 0.290 0.905 0.822 0.771 0.784 0.668 0.637 0.760 0.572 0.539 0.836 0.646 0.714 0.883 0.633 0.882 1.070 0.792 1.332 1.264 0.435 0.770 0.528 0.721 0.338 0.767 0.806 3.66

S. geminata 6 Guatemala City, Zone 10 36 0.250 0.850 0.777 0.739 0.750 0.617 0.567 0.732 0.548 0.561 0.883 0.591 0.654 0.840 0.599 0.788 1.021 0.757 1.210 1.179 0.399 0.719 0.505 0.641 0.321 0.772 0.752 3.44

S. geminata 6 Guatemala City, Zone 10 37 0.260 0.895 0.808 0.767 0.766 0.614 0.573 0.750 0.563 0.520 0.808 0.641 0.682 0.775 0.623 0.897 1.077 0.783 1.247 1.223 0.416 0.751 0.522 0.689 0.326 0.698 0.751 3.51

S. geminata 6 Guatemala City, Zone 10 38 0.240 0.900 0.812 0.770 0.776 0.643 0.537 0.747 0.619 0.535 0.799 0.667 0.578 0.771 0.569 0.784 1.069 0.712 1.267 1.179 0.394 0.732 0.511 0.775 0.311 0.724 0.745 3.58

S. geminata 6 Guatemala City, Zone 10 39 0.240 0.857 0.807 0.756 0.771 0.636 0.508 0.746 0.564 0.538 0.797 0.654 0.711 0.793 0.614 0.877 1.091 0.766 1.281 1.229 0.425 0.746 0.546 0.640 0.329 0.725 0.773 3.45

S. geminata 6 Guatemala City, Zone 10 40 0.240 0.891 0.800 0.760 0.778 0.653 0.604 0.747 0.577 0.541 0.915 0.642 0.680 0.857 0.628 0.686 1.091 0.775 1.151 1.222 0.423 0.743 0.536 0.673 0.317 0.773 0.780 3.56

S. geminata 6 Guatemala City, Zone 10 41 0.260 0.868 0.772 0.727 0.741 0.638 0.608 0.730 0.567 0.540 0.901 0.643 0.558 0.829 0.575 0.842 1.017 0.728 1.244 1.221 0.416 0.730 0.530 0.640 0.307 0.763 0.778 3.49

S. geminata 6 Guatemala City, Zone 10 42 0.250 0.836 0.762 0.724 0.731 0.637 0.650 0.732 0.542 0.534 0.810 0.585 0.685 0.834 0.572 0.827 1.010 0.735 1.280 1.166 0.405 0.709 0.512 0.671 0.308 0.750 0.739 3.42

S. geminata 6 Guatemala City, Zone 10 43 0.240 0.860 0.794 0.728 0.756 0.641 0.652 0.717 0.560 0.506 0.794 0.637 0.681 0.733 0.592 0.836 1.048 0.773 1.240 1.221 0.387 0.750 0.511 0.669 0.310 0.750 0.770 3.50

S. geminata 6 Guatemala City, Zone 10 44 0.220 0.833 0.752 0.715 0.740 0.626 0.550 0.722 0.568 0.500 0.871 0.591 0.667 0.841 0.546 0.832 1.032 0.723 1.223 1.176 0.384 0.727 0.496 0.673 0.319 0.732 0.778 3.41

S. geminata 6 Guatemala City, Zone 10 45 0.200 0.831 0.738 0.701 0.696 0.594 0.592 0.712 0.533 0.504 0.831 0.623 0.669 0.800 0.582 0.772 0.878 0.720 1.152 1.111 0.383 0.693 0.493 0.637 0.291 0.683 0.677 3.26

S. geminata 6 Guatemala City, Zone 10 46 0.210 0.835 0.733 0.687 0.715 0.635 0.654 0.731 0.546 0.513 0.849 0.574 0.674 0.816 0.543 0.820 0.987 0.680 1.247 1.141 0.374 0.699 0.473 0.688 0.290 0.742 0.728 3.41

S. geminata 6 Guatemala City, Zone 10 47 0.190 0.790 0.711 0.679 0.684 0.585 0.534 0.686 0.513 0.486 0.800 0.575 0.642 0.792 0.531 0.750 0.845 0.660 1.151 1.112 0.376 0.679 0.479 0.621 0.289 0.654 0.709 3.18

S. geminata 6 Guatemala City, Zone 10 48 0.170 0.787 0.710 0.676 0.685 0.575 0.541 0.684 0.509 0.482 0.744 0.555 0.661 0.781 0.548 0.768 0.972 0.689 1.159 1.130 0.411 0.681 0.493 0.630 0.299 0.634 0.701 3.18

S. geminata 6 Guatemala City, Zone 10 49 0.180 0.790 0.692 0.655 0.655 0.562 0.543 0.672 0.517 0.506 0.711 0.521 0.631 0.680 0.571 0.760 0.964 0.681 1.146 1.086 0.368 0.670 0.485 0.654 0.271 0.744 0.672 3.27

S. geminata 6 Guatemala City, Zone 10 50 0.170 0.799 0.705 0.660 0.669 0.59 0.633 0.675 0.519 0.479 0.830 0.577 0.625 0.769 0.543 0.769 0.970 0.673 1.160 1.098 0.374 0.681 0.474 0.632 0.283 0.675 0.709 3.20

S. geminata 9 Guatemala, Lake Atitlan 1 1.560 1.682 1.804 1.719 1.810 1.426 0.637 1.069 0.782 0.713 1.235 0.925 0.998 1.360 0.950 1.240 1.413 1.176 1.950 2.030 0.893 1.304 0.898 1.013 0.624 1.367 1.590 6.09

S. geminata 9 Guatemala, Lake Atitlan 2 1.650 1.785 1.940 1.918 1.888 1.471 0.689 1.041 0.734 0.637 1.249 0.969 0.991 1.420 0.902 1.235 1.586 1.091 1.841 2.095 0.854 1.322 1.076 0.982 0.591 1.301 1.559 6.16

S. geminata 9 Guatemala, Lake Atitlan 3 1.610 1.699 1.847 1.868 1.874 1.359 0.489 1.033 0.790 0.668 1.334 0.913 0.994 1.320 0.930 1.017 1.520 1.045 1.795 2.047 0.836 1.365 0.863 1.089 0.578 1.391 1.521 6.23

S. geminata 9 Guatemala, Lake Atitlan 4 0.990 1.559 1.596 1.578 1.545 1.253 0.751 0.965 0.748 0.634 1.117 0.897 0.960 1.077 0.899 1.197 1.446 1.042 1.790 1.793 0.671 1.136 0.906 0.930 0.520 1.201 1.220 5.48

S. geminata 9 Guatemala, Lake Atitlan 5 1.180 1.659 1.722 1.722 1.687 1.369 0.754 0.983 0.793 0.570 1.176 0.870 0.994 1.094 0.887 1.201 1.457 1.070 1.747 1.852 0.727 1.213 0.805 0.918 0.556 1.180 1.325 5.61

S. geminata 9 Guatemala, Lake Atitlan 6 1.370 1.588 1.660 1.620 1.678 1.432 0.849 0.946 0.737 0.637 1.215 0.981 0.955 1.351 0.945 1.177 1.502 1.072 1.769 2.115 0.847 1.424 0.847 0.995 0.586 1.244 1.582 5.94

S. geminata 9 Guatemala, Lake Atitlan 7 1.240 1.653 1.739 1.691 1.700 1.316 0.606 1.006 0.714 0.667 1.330 0.872 1.104 1.288 0.862 1.182 1.465 0.998 1.714 1.861 0.731 1.240 0.792 0.925 0.566 1.220 1.392 5.66

S. geminata 9 Guatemala, Lake Atitlan 8 1.530 1.677 1.800 1.759 1.765 1.402 0.590 1.015 0.779 0.648 1.208 0.959 1.026 1.186 0.923 1.215 1.546 1.091 1.832 2.031 0.820 1.311 0.878 1.029 0.594 1.179 1.376 5.92

S. geminata 9 Guatemala, Lake Atitlan 9 1.410 1.614 1.728 1.715 1.681 1.353 0.615 0.991 0.741 0.670 1.215 0.979 1.042 1.330 0.930 1.267 1.581 1.080 1.080 2.065 0.862 1.326 0.903 0.963 0.608 1.189 1.409 5.83

S. geminata 9 Guatemala, Lake Atitlan 10 1.030 1.513 1.598 1.549 1.550 1.255 0.652 0.980 0.742 0.654 1.164 0.959 0.992 1.254 0.854 1.163 1.481 1.105 1.753 1.828 0.705 1.135 0.825 0.929 0.525 1.143 1.213 5.41

S. geminata 9 Guatemala, Lake Atitlan 11 0.900 1.489 1.502 1.409 1.421 1.202 0.700 0.914 0.732 0.635 1.315 0.951 0.887 1.237 0.833 1.152 1.468 0.953 1.692 1.796 0.709 1.148 0.758 0.904 0.523 1.044 1.177 5.23

S. geminata 9 Guatemala, Lake Atitlan 12 1.230 1.604 1.708 1.682 1.636 1.276 0.543 0.945 0.761 0.649 1.192 0.952 0.919 1.289 0.915 1.201 1.516 1.116 1.766 1.861 0.763 1.215 0.825 0.923 0.532 1.253 1.317 5.64

S. geminata 9 Guatemala, Lake Atitlan 13 1.420 1.659 1.746 1.706 1.692 1.394 0.651 1.004 0.745 0.659 1.394 0.999 1.021 1.376 0.933 1.236 1.610 1.082 1.858 1.977 0.744 1.243 0.904 0.962 0.563 1.056 1.353 5.65

S. geminata 9 Guatemala, Lake Atitlan 14 0.810 1.470 1.422 1.381 1.401 1.127 0.727 0.898 0.665 0.613 1.105 0.830 0.915 1.037 0.820 1.108 1.390 1.007 1.652 1.743 0.632 1.096 0.739 0.892 0.494 1.025 1.170 5.13

S. geminata 9 Guatemala, Lake Atitlan 15 0.920 1.415 1.443 1.388 1.424 1.093 0.580 0.938 0.755 0.605 1.148 0.903 0.943 1.109 0.896 1.190 1.482 1.043 1.814 1.669 0.640 1.136 0.686 0.905 0.499 1.089 1.234 5.08

S. geminata 9 Guatemala, Lake Atitlan 16 0.470 1.238 1.144 1.089 1.130 0.869 0.616 0.893 0.641 0.605 1.015 0.745 0.790 1.095 0.733 1.028 1.236 0.923 1.557 1.493 0.564 0.932 0.647 0.773 0.410 0.866 0.982 4.37

S. geminata 9 Guatemala, Lake Atitlan 17 1.080 1.479 1.594 1.553 1.573 1.277 0.819 0.965 0.726 0.587 1.107 0.870 0.975 1.279 0.929 1.224 1.441 1.053 1.782 1.854 0.740 1.240 0.745 1.063 0.544 1.075 1.254 5.47

S. geminata 9 Guatemala, Lake Atitlan 18 1.680 1.598 1.856 1.843 1.857 1.533 0.903 0.972 0.745 0.662 1.197 0.901 1.012 1.318 0.908 1.199 1.476 1.078 1.756 1.939 0.893 1.327 0.840 1.021 0.597 1.192 1.463 5.75

S. geminata 9 Guatemala, Lake Atitlan 19 0.930 1.408 1.493 1.431 1.472 1.279 0.604 0.909 0.699 0.634 1.099 0.841 0.802 1.197 0.838 1.138 1.430 0.988 1.709 1.694 0.684 1.055 0.718 0.890 0.478 1.009 1.183 5.00

S. geminata 9 Guatemala, Lake Atitlan 20 0.900 1.428 1.469 1.428 1.442 1.181 0.770 0.935 0.695 0.624 1.098 0.945 0.937 1.233 0.863 1.142 1.437 1.003 1.708 1.694 0.646 1.103 0.715 0.902 0.499 1.105 1.190 5.13

S. geminata 9 Guatemala, Lake Atitlan 21 1.010 1.522 1.612 1.548 1.578 1.27 0.689 0.960 0.721 0.626 1.130 0.905 0.907 1.240 0.885 0.963 1.459 1.063 1.712 1.683 0.678 1.158 0.691 0.914 0.505 1.090 1.216 5.21

S. geminata 9 Guatemala, Lake Atitlan 22 0.470 1.136 1.061 0.987 1.042 0.868 0.540 0.797 0.615 0.563 0.531 0.429 0.401 0.588 0.400 0.417 0.713 0.445 0.850 1.435 0.559 0.895 0.593 0.740 0.403 1.047 1.049 4.36

S. geminata 9 Guatemala, Lake Atitlan 23 0.350 1.024 0.970 0.930 0.920 0.756 0.549 0.777 0.601 0.528 0.877 0.696 0.701 0.839 0.632 0.898 1.141 0.792 1.339 1.322 0.495 0.816 0.586 0.726 0.362 0.962 0.946 4.03

S. geminata 9 Guatemala, Lake Atitlan 24 0.380 1.075 0.977 0.927 0.951 0.791 0.389 0.769 0.552 0.563 0.866 0.705 0.751 0.948 0.695 0.916 1.160 0.784 1.388 1.333 0.550 0.850 0.557 0.759 0.357 0.897 1.008 4.06

S. geminata 9 Guatemala, Lake Atitlan 25 0.280 0.951 0.871 0.824 0.841 0.685 0.458 0.743 0.564 0.537 0.937 0.645 0.752 0.905 0.619 0.848 1.100 0.773 1.325 1.243 0.448 0.757 0.520 0.684 0.336 0.939 0.895 3.82

S. geminata 9 Guatemala, Lake Atitlan 26 0.340 1.057 0.955 0.887 0.928 0.747 0.477 0.803 0.618 0.537 0.834 0.717 0.554 0.953 0.675 0.938 1.163 0.812 1.439 1.346 0.493 0.821 0.592 0.735 0.363 0.960 0.987 4.10

S. geminata 9 Guatemala, Lake Atitlan 27 0.370 1.029 0.927 0.855 0.898 0.725 0.479 0.737 0.570 0.531 1.005 0.688 0.729 0.918 0.633 0.910 1.097 0.783 1.324 1.334 0.494 0.847 0.557 0.720 0.359 0.930 0.968 4.01

S. geminata 9 Guatemala, Lake Atitlan 28 0.290 0.927 0.839 0.785 0.779 0.657 0.395 0.716 0.550 0.523 0.914 0.676 0.733 0.889 0.614 0.832 1.091 0.749 1.282 1.196 0.445 0.748 0.502 0.681 0.318 0.849 0.883 3.65

S. geminata 9 Guatemala, Lake Atitlan 29 0.210 0.911 0.809 0.755 0.779 0.627 0.456 0.707 0.579 0.439 0.796 0.621 0.668 0.852 0.599 0.813 1.073 0.776 1.276 1.200 0.429 0.739 0.508 0.643 0.309 0.821 0.861 3.58

S. geminata 9 Guatemala, Lake Atitlan 30 0.410 1.033 0.936 0.884 0.885 0.728 0.521 0.779 0.586 0.536 0.876 0.694 0.782 0.962 0.671 0.959 1.198 0.836 1.428 1.333 0.486 0.825 0.559 0.717 0.362 0.922 0.950 4.00

S. geminata 9 Guatemala, Lake Atitlan 31 0.170 0.719 0.614 0.577 0.560 0.5 0.317 0.574 0.397 0.456 0.722 0.577 0.496 0.682 0.435 0.655 0.813 0.554 0.983 0.955 0.333 0.600 0.396 0.550 0.232 0.663 0.612 2.89

S. geminata 9 Guatemala, Lake Atitlan 32 0.110 0.676 0.573 0.530 0.532 0.444 0.345 0.536 0.398 0.422 0.602 0.512 0.516 0.626 0.424 0.596 0.751 0.532 0.953 0.892 0.322 0.558 0.387 0.526 0.231 0.657 0.602 2.75

S. geminata 9 Guatemala, Lake Atitlan 33 0.130 0.681 0.583 0.549 0.529 0.426 0.291 0.559 0.381 0.408 0.675 0.482 0.540 0.612 0.424 0.603 0.767 0.500 0.909 0.917 0.327 0.582 0.369 0.504 0.227 0.604 0.609 2.71

S. geminata 9 Guatemala, Lake Atitlan 34 0.110 0.717 0.624 0.576 0.601 0.52 0.403 0.571 0.436 0.469 0.701 0.536 0.560 0.674 0.435 0.700 0.810 0.604 1.021 0.943 0.363 0.589 0.395 0.536 0.239 0.652 0.647 2.85

S. geminata 9 Guatemala, Lake Atitlan 35 0.130 0.705 0.586 0.552 0.552 0.459 0.354 0.561 0.402 0.432 0.675 0.487 0.522 0.569 0.427 0.641 0.703 0.545 0.970 0.917 0.337 0.572 0.383 0.508 0.239 0.610 0.605 2.74

S. geminata 9 Guatemala, Lake Atitlan 36 0.130 0.683 0.612 0.572 0.589 0.494 0.312 0.592 0.432 0.449 0.628 0.524 0.552 0.604 0.453 0.706 0.814 0.568 0.995 0.964 0.334 0.592 0.409 0.590 0.243 0.663 0.649 2.90

S. geminata 9 Guatemala, Lake Atitlan 37 0.110 0.700 0.596 0.566 0.570 0.452 0.344 0.558 0.408 0.430 0.611 0.497 0.551 0.646 0.455 0.664 0.798 0.576 0.988 0.945 0.324 0.589 0.395 0.510 0.234 0.572 0.584 2.73

S. geminata 9 Guatemala, Lake Atitlan 38 0.100 0.687 0.573 0.539 0.546 0.46 0.278 0.545 0.402 0.421 0.587 0.424 0.433 0.611 0.438 0.613 0.751 0.532 0.929 0.918 0.313 0.575 0.385 0.512 0.253 0.622 0.617 2.74

S. geminata 9 Guatemala, Lake Atitlan 39 0.110 0.706 0.595 0.554 0.560 0.461 0.360 0.569 0.408 0.436 0.629 0.482 0.557 0.676 0.460 0.646 0.822 0.570 0.964 0.900 0.332 0.556 0.379 0.492 0.228 0.600 0.677 2.70

S. geminata 9 Guatemala, Lake Atitlan 40 0.120 0.722 0.594 0.554 0.577 0.487 0.353 0.582 0.408 0.454 0.620 0.523 0.541 0.649 0.462 0.641 0.798 0.578 0.965 0.950 0.320 0.576 0.391 0.519 0.227 0.587 0.592 2.78

S. geminata 9 Guatemala, Lake Atitlan 41 0.100 0.664 0.594 0.563 0.570 0.481 0.348 0.563 0.398 0.425 0.713 0.451 0.538 0.640 0.443 0.642 0.770 0.525 0.960 0.918 0.322 0.577 0.365 0.519 0.237 0.562 0.610 2.66

S. geminata 9 Guatemala, Lake Atitlan 42 0.140 0.708 0.604 0.560 0.573 0.495 0.392 0.578 0.410 0.423 0.680 0.449 0.650 0.674 0.479 0.656 0.836 0.586 0.985 0.916 0.317 0.581 0.388 0.534 0.241 0.611 0.637 2.77

S. geminata 9 Guatemala, Lake Atitlan 43 0.100 0.684 0.580 0.546 0.556 0.512 0.355 0.580 0.419 0.414 0.727 0.472 0.464 0.649 0.444 0.642 0.809 0.567 0.987 0.911 0.322 0.548 0.410 0.491 0.227 0.584 0.583 2.67

S. geminata 9 Guatemala, Lake Atitlan 44 0.070 0.668 0.573 0.543 0.533 0.457 0.341 0.564 0.404 0.426 0.682 0.453 0.575 0.646 0.411 0.633 0.769 0.524 0.939 0.892 0.300 0.552 0.375 0.528 0.224 0.577 0.567 2.66

S. geminata 9 Guatemala, Lake Atitlan 45 0.120 0.658 0.579 0.549 0.556 0.501 0.307 0.554 0.392 0.430 0.661 0.462 0.510 0.631 0.433 0.600 0.770 0.540 0.943 0.899 0.320 0.551 0.392 0.560 0.236 0.580 0.585 2.70

S. geminata 9 Guatemala, Lake Atitlan 46 0.130 0.681 0.563 0.523 0.533 0.47 0.327 0.523 0.404 0.427 0.690 0.434 0.496 0.610 0.415 0.623 0.734 0.512 0.930 0.879 0.317 0.555 0.362 0.493 0.229 0.590 0.586 2.64

S. geminata 9 Guatemala, Lake Atitlan 47 0.140 0.688 0.563 0.536 0.533 0.463 0.335 0.553 0.394 0.426 0.684 0.469 0.521 0.570 0.411 0.633 0.770 0.549 0.947 0.892 0.303 0.548 0.382 0.508 0.229 0.585 0.570 2.67

S. geminata 9 Guatemala, Lake Atitlan 48 0.100 0.698 0.585 0.567 0.544 0.47 0.339 0.562 0.415 0.421 0.608 0.471 0.518 0.631 0.442 0.657 0.785 0.566 0.997 0.919 0.338 0.565 0.387 0.536 0.240 0.591 0.618 2.74

S. geminata 9 Guatemala, Lake Atitlan 49 0.090 0.690 0.572 0.542 0.536 0.446 0.328 0.528 0.389 0.410 0.724 0.509 0.495 0.538 0.445 0.612 0.698 0.508 0.947 0.904 0.323 0.559 0.386 0.572 0.246 0.517 0.614 2.68

S. geminata 9 Guatemala, Lake Atitlan 50 0.120 0.731 0.611 0.577 0.577 0.495 0.332 0.582 0.424 0.454 0.656 0.456 0.507 0.661 0.458 0.655 0.826 0.582 0.989 0.948 0.341 0.581 0.410 0.514 0.241 0.600 0.627 2.79

S. geminata 10 Guatemala, Ixchimche 1 1.210 1.595 1.692 1.682 1.655 1.339 0.851 1.024 0.790 0.653 1.345 0.899 1.007 1.334 0.864 1.189 1.359 1.102 1.704 1.962 0.776 1.254 0.856 0.961 0.510 1.229 1.370 5.75

S. geminata 10 Guatemala, Ixchimche 2 0.940 1.454 1.517 1.516 1.448 1.195 0.776 0.959 0.735 0.639 1.250 0.914 0.919 1.126 0.874 1.117 1.456 1.026 1.661 1.759 0.635 1.127 0.777 0.901 0.482 1.021 1.297 5.14

S. geminata 10 Guatemala, Ixchimche 3 1.240 1.595 1.691 1.673 1.641 1.238 0.585 0.986 0.749 0.654 1.157 0.854 0.985 1.145 0.913 1.129 1.509 1.043 1.745 1.920 0.702 1.230 0.834 0.979 0.535 1.074 1.402 5.57

S. geminata 10 Guatemala, Ixchimche 4 0.980 1.503 1.590 1.572 1.544 1.247 0.709 0.994 0.735 0.638 1.133 0.902 0.946 1.271 0.902 1.137 1.463 1.091 1.693 1.816 0.725 1.162 0.760 1.006 0.493 1.067 1.264 5.39

S. geminata 10 Guatemala, Ixchimche 5 1.050 1.539 1.547 1.541 1.510 1.204 0.572 1.000 0.744 0.648 1.329 0.917 0.966 1.274 0.864 1.164 1.467 1.072 1.757 1.878 0.774 1.229 0.841 1.026 0.513 1.083 1.260 5.53

S. geminata 10 Guatemala, Ixchimche 6 1.120 1.568 1.671 1.629 1.603 1.248 0.621 0.993 0.757 0.626 1.293 0.908 0.947 1.141 0.858 1.169 1.394 1.007 1.693 1.979 0.834 1.355 0.815 0.956 0.547 1.071 1.324 5.57

S. geminata 10 Guatemala, Ixchimche 7 1.030 1.471 1.605 1.582 1.560 1.229 0.663 0.988 0.724 0.645 1.255 0.896 0.933 1.264 0.877 1.169 1.481 1.082 1.688 1.731 0.747 1.124 0.765 1.081 0.499 1.067 1.282 5.35

S. geminata 10 Guatemala, Ixchimche 8 0.960 1.477 1.558 1.511 1.498 1.189 0.594 0.973 0.745 0.631 1.294 0.908 0.936 1.272 0.847 1.167 1.503 1.025 1.687 1.779 0.683 1.110 0.779 0.973 0.507 1.040 1.326 5.27

S. geminata 10 Guatemala, Ixchimche 9 0.850 1.458 1.455 1.432 1.423 1.104 0.713 0.959 0.705 0.619 1.222 0.891 0.867 1.207 0.841 1.063 1.434 1.003 1.589 1.751 0.738 1.152 0.727 0.950 0.478 1.064 1.183 5.22

S. geminata 10 Guatemala, Ixchimche 10 0.870 1.441 1.504 1.475 1.431 1.136 0.620 0.969 0.737 0.629 1.276 0.852 0.916 1.250 0.867 1.102 1.469 1.025 1.689 1.788 0.739 1.127 0.780 0.896 0.472 1.053 1.171 5.18

S. geminata 10 Guatemala, Ixchimche 11 0.700 1.338 1.302 1.270 1.256 0.97 0.663 0.915 0.708 0.612 1.184 0.843 0.889 1.179 0.839 1.034 1.416 0.956 1.623 1.615 0.631 1.038 0.676 0.869 0.467 1.049 1.120 4.87

S. geminata 10 Guatemala, Ixchimche 12 0.620 1.301 1.274 1.215 1.229 1.007 0.578 0.889 0.661 0.579 1.189 0.814 0.775 0.996 0.785 1.072 1.360 0.932 1.526 1.576 0.649 0.995 0.711 0.898 0.471 1.060 1.102 4.83

S. geminata 10 Guatemala, Ixchimche 13 0.620 1.288 1.274 1.237 1.204 0.959 0.619 0.891 0.695 0.602 1.141 0.830 0.890 1.118 0.797 1.118 1.353 0.977 1.551 1.589 0.617 0.997 0.676 0.903 0.427 1.036 1.078 4.82

S. geminata 10 Guatemala, Ixchimche 14 0.610 1.338 1.283 1.238 1.237 0.967 0.667 0.906 0.680 0.599 1.170 0.830 0.870 1.155 0.780 1.058 1.360 0.978 1.560 1.609 0.589 1.006 0.691 0.842 0.451 0.968 1.088 4.76

S. geminata 10 Guatemala, Ixchimche 15 0.340 1.103 1.011 0.970 0.947 0.792 0.560 0.801 0.607 0.561 0.996 0.708 0.794 0.867 0.706 0.938 1.195 0.831 1.393 1.338 0.459 0.821 0.586 0.711 0.362 0.849 0.867 4.00

S. geminata 10 Guatemala, Ixchimche 16 0.470 1.172 1.147 1.108 1.116 0.902 0.582 0.865 0.629 0.575 1.102 0.755 0.867 1.081 0.744 1.019 1.304 0.910 1.514 1.491 0.561 0.925 0.648 0.810 0.392 0.943 0.984 4.42

S. geminata 10 Guatemala, Ixchimche 17 0.180 0.838 0.729 0.667 0.716 0.589 0.425 0.669 0.521 0.495 0.838 0.594 0.670 0.792 0.558 0.798 0.977 0.690 1.187 1.116 0.397 0.698 0.453 0.653 0.286 0.744 0.718 3.35

S. geminata 10 Guatemala, Ixchimche 18 0.200 0.892 0.773 0.722 0.732 0.625 0.458 0.707 0.519 0.503 0.860 0.658 0.670 0.834 0.610 0.769 1.022 0.726 1.209 1.152 0.417 0.715 0.486 0.694 0.270 0.693 0.763 3.43

S. geminata 10 Guatemala, Ixchimche 19 0.270 0.990 0.895 0.858 0.844 0.664 0.504 0.788 0.577 0.537 0.954 0.669 0.743 0.945 0.638 0.895 1.171 0.790 1.362 1.307 0.461 0.786 0.574 0.749 0.333 0.849 0.869 3.90

S. geminata 10 Guatemala, Ixchimche 20 0.150 0.819 0.656 0.633 0.647 0.541 0.416 0.631 0.484 0.471 0.764 0.576 0.616 0.739 0.500 0.717 0.922 0.634 1.058 1.053 0.396 0.622 0.466 0.605 0.268 0.733 0.651 3.21

S. geminata 10 Guatemala, Ixchimche 21 0.170 0.828 0.718 0.676 0.695 0.539 0.374 0.653 0.495 0.481 0.786 0.525 0.654 0.772 0.528 0.788 0.961 0.663 1.142 1.123 0.391 0.675 0.496 0.628 0.307 0.660 0.722 3.24

S. geminata 10 Guatemala, Ixchimche 22 0.220 0.882 0.804 0.758 0.763 0.63 0.462 0.705 0.572 0.520 0.812 0.635 0.685 0.879 0.630 0.851 1.045 0.760 1.318 1.204 0.403 0.711 0.503 0.714 0.315 0.800 0.719 3.60

S. geminata 10 Guatemala, Ixchimche 23 0.140 0.808 0.672 0.622 0.631 0.529 0.422 0.631 0.455 0.473 0.747 0.522 0.611 0.744 0.494 0.718 0.902 0.649 1.073 1.028 0.365 0.637 0.442 0.620 0.269 0.694 0.688 3.15

S. geminata 10 Guatemala, Ixchimche 24 0.130 0.782 0.657 0.616 0.620 0.517 0.382 0.632 0.469 0.481 0.764 0.505 0.614 0.729 0.513 0.684 0.902 0.623 1.090 1.057 0.325 0.650 0.437 0.590 0.261 0.627 0.652 3.06

S. geminata 10 Guatemala, Ixchimche 25 0.230 0.911 0.800 0.732 0.773 0.634 0.422 0.727 0.564 0.513 0.915 0.635 0.678 0.840 0.595 0.795 1.058 0.749 1.221 1.151 0.399 0.728 0.473 0.688 0.308 0.602 0.785 3.35

S. geminata 10 Guatemala, Ixchimche 26 0.210 0.860 0.776 0.720 0.754 0.627 0.448 0.701 0.487 0.518 0.904 0.591 0.652 0.823 0.580 0.801 0.953 0.697 1.180 1.211 0.408 0.723 0.532 0.599 0.298 0.785 0.739 3.46

S. geminata 10 Guatemala, Ixchimche 27 0.110 0.732 0.618 0.610 0.600 0.494 0.381 0.596 0.450 0.439 0.708 0.483 0.551 0.690 0.469 0.694 0.832 0.603 1.039 0.998 0.375 0.636 0.411 0.570 0.266 0.585 0.626 2.89

S. geminata 10 Guatemala, Ixchimche 28 0.180 0.850 0.726 0.694 0.708 0.612 0.442 0.688 0.469 0.509 0.838 0.593 0.664 0.808 0.567 0.777 1.009 0.711 1.193 1.198 0.423 0.715 0.527 0.628 0.287 0.645 0.768 3.32

S. geminata 10 Guatemala, Ixchimche 29 0.120 0.758 0.648 0.611 0.588 0.487 0.358 0.604 0.432 0.464 0.749 0.525 0.570 0.711 0.468 0.721 0.860 0.584 1.078 1.012 0.369 0.645 0.417 0.559 0.241 0.702 0.595 3.03

S. geminata 10 Guatemala, Ixchimche 30 0.170 0.790 0.694 0.657 0.666 0.57 0.409 0.654 0.475 0.484 0.799 0.559 0.602 0.747 0.542 0.746 0.934 0.673 1.128 1.120 0.364 0.663 0.490 0.556 0.272 0.688 0.659 3.15

S. geminata 10 Guatemala, Ixchimche 31 0.200 0.896 0.778 0.732 0.745 0.585 0.459 0.704 0.532 0.524 0.922 0.623 0.689 0.891 0.592 0.821 1.060 0.751 1.263 1.202 0.402 0.722 0.496 0.615 0.302 0.815 0.716 3.53

S. geminata 10 Guatemala, Ixchimche 32 0.190 0.828 0.713 0.689 0.677 0.57 0.407 0.666 0.503 0.483 0.848 0.611 0.662 0.707 0.581 0.807 0.977 0.686 1.197 1.104 0.396 0.691 0.485 0.689 0.289 0.659 0.758 3.28

S. geminata 10 Guatemala, Ixchimche 33 0.150 0.729 0.686 0.629 0.657 0.541 0.384 0.634 0.463 0.477 0.743 0.503 0.607 0.708 0.487 0.716 0.881 0.600 1.076 1.049 0.413 0.659 0.429 0.590 0.268 0.609 0.626 2.98

S. geminata 10 Guatemala, Ixchimche 34 0.190 0.816 0.683 0.637 0.670 0.568 0.401 0.670 0.487 0.503 0.845 0.589 0.635 0.766 0.527 0.755 0.967 0.641 1.159 1.101 0.375 0.694 0.446 0.630 0.292 0.681 0.695 3.23

S. geminata 10 Guatemala, Ixchimche 35 0.220 0.905 0.772 0.726 0.740 0.658 0.465 0.696 0.538 0.498 0.855 0.626 0.677 0.847 0.607 0.802 1.044 0.701 1.248 1.174 0.410 0.719 0.503 0.667 0.305 0.747 0.768 3.49

S. geminata 10 Guatemala, Ixchimche 36 0.140 0.777 0.657 0.620 0.625 0.533 0.381 0.642 0.470 0.468 0.768 0.509 0.595 0.715 0.484 0.722 0.901 0.631 1.042 1.047 0.390 0.621 0.484 0.569 0.254 0.726 0.642 3.12

S. geminata 10 Guatemala, Ixchimche 37 0.130 0.768 0.650 0.622 0.613 0.527 0.380 0.613 0.447 0.494 0.768 0.512 0.583 0.705 0.484 0.689 0.880 0.605 1.034 0.984 0.364 0.594 0.408 0.594 0.267 0.595 0.623 2.94

S. geminata 10 Guatemala, Ixchimche 38 0.160 0.797 0.691 0.645 0.650 0.548 0.391 0.656 0.475 0.492 0.806 0.555 0.597 0.758 0.518 0.741 0.953 0.685 1.093 1.076 0.385 0.664 0.466 0.604 0.275 0.721 0.637 3.20

S. geminata 10 Guatemala, Ixchimche 39 0.120 0.772 0.662 0.625 0.616 0.533 0.401 0.645 0.465 0.473 0.815 0.567 0.568 0.780 0.553 0.714 0.931 0.666 1.068 1.033 0.355 0.628 0.431 0.602 0.254 0.602 0.658 3.01

S. geminata 10 Guatemala, Ixchimche 40 0.140 0.787 0.677 0.635 0.653 0.539 0.393 0.641 0.467 0.474 0.797 0.523 0.608 0.794 0.502 0.718 0.946 0.649 1.088 1.095 0.354 0.653 0.495 0.564 0.273 0.620 0.659 3.07

S. geminata 10 Guatemala, Ixchimche 41 0.140 0.768 0.644 0.611 0.616 0.524 0.366 0.636 0.462 0.449 0.749 0.564 0.597 0.737 0.551 0.743 0.892 0.637 1.100 1.002 0.360 0.609 0.415 0.578 0.259 0.636 0.620 2.98

S. geminata 10 Guatemala, Ixchimche 42 0.130 0.761 0.663 0.621 0.636 0.565 0.395 0.590 0.446 0.460 0.768 0.513 0.570 0.703 0.459 0.699 0.874 0.634 1.081 1.005 0.396 0.626 0.415 0.542 0.265 0.593 0.653 2.90

S. geminata 10 Guatemala, Ixchimche 43 0.170 0.795 0.689 0.648 0.653 0.529 0.424 0.653 0.485 0.493 0.786 0.550 0.616 0.751 0.522 0.750 0.937 0.651 1.118 1.105 0.400 0.662 0.490 0.567 0.279 0.706 0.743 3.17

S. geminata 10 Guatemala, Ixchimche 44 0.170 0.804 0.699 0.657 0.653 0.552 0.401 0.652 0.488 0.463 0.759 0.596 0.549 0.736 0.508 0.731 0.924 0.622 1.061 1.063 0.377 0.664 0.417 0.563 0.271 0.794 0.655 3.22

S. geminata 10 Guatemala, Ixchimche 45 0.220 0.910 0.791 0.753 0.757 0.674 0.462 0.701 0.519 0.508 0.858 0.596 0.670 0.811 0.571 0.800 1.023 0.728 1.184 1.212 0.467 0.741 0.514 0.675 0.284 0.745 0.725 3.54

S. geminata 10 Guatemala, Ixchimche 46 0.160 0.803 0.680 0.638 0.671 0.549 0.392 0.630 0.477 0.464 0.767 0.514 0.579 0.743 0.501 0.708 0.924 0.626 1.046 1.043 0.424 0.660 0.430 0.583 0.255 0.699 0.627 3.13

S. geminata 10 Guatemala, Ixchimche 47 0.150 0.759 0.649 0.626 0.630 0.538 0.389 0.610 0.463 0.472 0.744 0.534 0.570 0.723 0.471 0.691 0.919 0.609 1.066 1.008 0.350 0.621 0.429 0.554 0.262 0.624 0.683 2.95

S. geminata 10 Guatemala, Ixchimche 48 0.150 0.781 0.662 0.625 0.634 0.547 0.397 0.633 0.482 0.461 0.788 0.527 0.599 0.743 0.493 0.710 0.905 0.642 1.084 1.039 0.385 0.621 0.467 0.554 0.256 0.606 0.652 2.98

S. geminata 10 Guatemala, Ixchimche 49 0.150 0.810 0.691 0.664 0.668 0.552 0.410 0.658 0.489 0.481 0.814 0.560 0.671 0.750 0.512 0.743 0.934 0.668 1.173 1.104 0.400 0.647 0.481 0.565 0.286 0.680 0.659 3.16

S. geminata 10 Guatemala, Ixchimche 50 0.130 0.777 0.639 0.607 0.612 0.524 0.387 0.629 0.473 0.456 0.773 0.561 0.580 0.629 0.542 0.656 0.907 0.635 1.060 0.997 0.392 0.627 0.422 0.573 0.245 0.581 0.613 2.93

S. geminata 17 Guatemala, El Peten 1 0.300 0.950 0.850 0.789 0.807 0.667 0.401 0.800 0.580 0.551 0.796 0.565 0.902 0.806 0.647 0.933 1.004 0.787 1.370 1.311 0.414 0.801 0.552 0.795 0.327 0.856 0.917 3.91

S. geminata 17 Guatemala, El Peten 2 0.330 0.940 0.896 0.823 0.861 0.761 0.479 0.836 0.644 0.547 0.824 0.629 0.863 0.830 0.640 0.969 1.014 0.807 1.450 1.303 0.452 0.816 0.567 0.744 0.343 0.817 0.934 3.80

S. geminata 17 Guatemala, El Peten 3 0.320 1.001 0.929 0.840 0.875 0.756 0.462 0.817 0.594 0.582 0.859 0.686 0.797 0.811 0.671 0.978 1.042 0.836 1.417 1.344 0.434 0.858 0.569 0.835 0.359 0.880 0.986 4.06

S. geminata 17 Guatemala, El Peten 4 0.340 0.939 0.878 0.789 0.833 0.672 0.388 0.796 0.584 0.540 0.803 0.634 0.802 0.768 0.628 0.906 0.983 0.806 1.344 1.288 0.381 0.783 0.539 0.747 0.342 0.811 0.922 3.78

S. geminata 17 Guatemala, El Peten 5 0.280 0.902 0.819 0.757 0.784 0.639 0.405 0.788 0.572 0.552 0.798 0.602 0.755 0.740 0.588 0.893 1.001 0.740 1.323 1.244 0.445 0.801 0.527 0.713 0.329 0.774 0.813 3.63

S. geminata 17 Guatemala, El Peten 6 0.250 0.845 0.745 0.689 0.713 0.617 0.347 0.704 0.518 0.551 0.726 0.549 0.751 0.694 0.551 0.893 0.798 0.704 1.281 1.162 0.389 0.712 0.477 0.659 0.280 0.768 0.758 3.43

S. geminata 17 Guatemala, El Peten 7 0.740 1.289 1.339 1.283 1.261 1.106 0.654 0.979 0.749 0.642 1.093 0.830 1.002 1.051 0.806 1.152 1.240 0.995 1.711 1.661 0.536 0.995 0.729 0.873 0.432 1.026 1.129 4.85

S. geminata 17 Guatemala, El Peten 8 0.240 0.934 0.811 0.751 0.757 0.656 0.474 0.767 0.556 0.536 0.738 0.584 0.750 0.755 0.598 0.867 0.953 0.749 1.297 1.212 0.406 0.736 0.543 0.652 0.306 0.730 0.822 3.53

S. geminata 17 Guatemala, El Peten 9 0.260 0.922 0.823 0.756 0.788 0.634 0.459 0.795 0.564 0.542 0.788 0.606 0.721 0.759 0.623 0.851 0.939 0.767 1.326 1.304 0.417 0.812 0.567 0.674 0.330 0.845 0.840 3.75

S. geminata 17 Guatemala, El Peten 10 0.240 0.850 0.772 0.717 0.721 0.617 0.385 0.741 0.559 0.509 0.811 0.591 0.710 0.668 0.568 0.841 0.917 0.723 1.263 1.182 0.367 0.745 0.533 0.639 0.322 0.786 0.771 3.46

S. geminata 17 Guatemala, El Peten 11 0.330 1.001 0.901 0.835 0.855 0.746 0.430 0.837 0.605 0.573 0.830 0.627 0.884 0.822 0.662 0.949 1.024 0.778 1.420 1.373 0.456 0.840 0.620 0.715 0.340 0.876 0.980 3.96

S. geminata 17 Guatemala, El Peten 12 0.240 0.900 0.828 0.756 0.775 0.661 0.396 0.768 0.559 0.538 0.837 0.615 0.785 0.748 0.621 0.912 0.985 0.761 1.335 1.267 0.450 0.803 0.517 0.766 0.341 0.812 0.873 3.75

S. geminata 17 Guatemala, El Peten 13 0.290 0.945 0.850 0.806 0.81 0.756 0.456 0.795 0.618 0.559 0.793 0.608 0.785 0.782 0.605 0.921 1.022 0.765 1.356 1.293 0.428 0.800 0.603 0.673 0.334 0.833 0.872 3.74

S. geminata 17 Guatemala, El Peten 14 0.680 1.253 1.217 1.197 1.185 1.022 0.551 0.986 0.685 0.653 1.022 0.774 0.990 1.001 0.807 1.137 1.235 0.959 1.683 1.572 0.544 0.972 0.709 0.829 0.449 1.056 1.157 4.71

S. geminata 17 Guatemala, El Peten 15 0.200 0.812 0.729 0.667 0.686 0.584 0.396 0.716 0.540 0.499 0.705 0.544 0.694 0.707 0.536 0.839 0.905 0.679 1.228 1.141 0.368 0.698 0.504 0.674 0.283 0.738 0.761 3.37

S. geminata 17 Guatemala, El Peten 16 0.240 0.912 0.813 0.773 0.768 0.668 0.485 0.782 0.568 0.542 0.703 0.580 0.803 0.773 0.601 0.892 0.979 0.748 1.353 1.256 0.434 0.777 0.518 0.785 0.324 0.787 0.801 3.74

S. geminata 17 Guatemala, El Peten 17 0.300 0.953 0.857 0.795 0.829 0.684 0.405 0.802 0.593 0.560 0.856 0.598 0.798 0.800 0.649 0.896 1.023 0.799 1.350 1.291 0.467 0.808 0.562 0.735 0.326 0.868 0.916 3.85

S. geminata 17 Guatemala, El Peten 18 0.680 1.250 1.221 1.182 1.189 1.036 0.624 0.956 0.730 0.622 1.017 0.746 1.005 0.979 0.778 1.107 1.256 0.973 1.694 1.589 0.546 1.008 0.651 0.907 0.443 1.085 1.113 4.83

S. geminata 17 Guatemala, El Peten 19 0.230 0.862 0.772 0.700 0.735 0.617 0.378 0.742 0.572 0.519 0.747 0.600 0.715 0.753 0.582 0.835 0.895 0.721 1.264 1.188 0.421 0.739 0.520 0.711 0.316 0.753 0.794 3.51

S. geminata 17 Guatemala, El Peten 20 0.280 0.923 0.846 0.777 0.811 0.704 0.454 0.805 0.596 0.561 0.813 0.666 0.806 0.802 0.621 0.931 1.021 0.765 1.384 1.278 0.394 0.784 0.539 0.741 0.323 0.829 0.879 3.77

S. geminata 17 Guatemala, El Peten 21 1.660 1.673 1.850 1.817 1.813 1.439 0.712 1.126 0.849 0.704 1.261 0.956 1.117 1.226 0.945 1.333 1.380 1.139 1.984 1.966 0.733 1.261 0.857 1.000 0.557 1.293 1.577 5.93

S. geminata 17 Guatemala, El Peten 22 1.350 1.514 1.642 1.614 1.611 1.338 0.646 1.056 0.820 0.677 1.191 0.896 1.068 1.137 0.873 1.293 1.358 1.117 1.832 1.855 0.687 1.164 0.822 0.936 0.523 1.159 1.361 5.46

S. geminata 17 Guatemala, El Peten 23 1.060 1.376 1.435 1.385 1.4 1.165 0.624 1.063 0.691 0.718 1.151 0.851 1.068 1.061 0.897 1.251 1.317 1.063 1.904 1.717 0.647 1.098 0.738 0.925 0.457 1.102 1.274 5.12

S. geminata 17 Guatemala, El Peten 24 1.340 1.569 1.658 1.637 1.617 1.366 0.676 1.064 0.820 0.665 1.211 0.914 1.091 1.149 0.922 1.303 1.426 1.126 1.953 1.868 0.725 1.199 0.792 1.026 0.511 1.220 1.452 5.68

S. geminata 17 Guatemala, El Peten 25 0.750 1.242 1.242 1.208 1.211 1.048 0.594 0.957 0.729 0.635 1.049 0.800 0.960 1.000 0.770 1.154 1.164 0.988 1.747 1.668 0.567 1.028 0.728 0.883 0.453 1.075 1.196 4.87

S. geminata 17 Guatemala, El Peten 26 0.750 1.289 1.317 1.278 1.269 1.078 0.629 0.996 0.741 0.633 1.039 0.813 1.006 1.007 0.825 1.149 1.268 0.998 1.734 1.625 0.547 1.005 0.686 0.940 0.462 1.122 1.117 4.98

S. geminata 17 Guatemala, El Peten 27 0.540 1.172 1.122 1.067 1.087 0.950 0.602 0.916 0.699 0.638 0.995 0.754 0.912 0.966 0.752 1.100 1.203 0.924 1.662 1.536 0.562 0.956 0.658 0.894 0.414 0.964 1.038 4.57

S. geminata 17 Guatemala, El Peten 28 0.740 1.239 1.256 1.212 1.192 1.034 0.554 0.911 0.729 0.611 1.042 0.812 0.945 1.033 0.804 1.163 1.215 0.976 1.715 1.634 0.539 1.012 0.689 0.877 0.447 1.077 1.142 4.83

S. geminata 17 Guatemala, El Peten 29 0.680 1.267 1.250 1.217 1.192 1.050 0.638 0.968 0.713 0.633 1.045 0.794 0.964 1.044 0.784 1.162 1.232 0.977 1.703 1.661 0.556 1.004 0.724 0.854 0.449 1.012 1.107 4.79

S. geminata 17 Guatemala, El Peten 30 0.760 1.255 1.280 1.232 1.267 1.093 0.644 0.972 0.730 0.647 1.072 0.817 0.997 1.042 0.842 1.176 1.274 1.012 1.742 1.650 0.583 1.030 0.681 0.900 0.440 1.062 1.179 4.87

S. geminata 17 Guatemala, El Peten 31 0.230 0.867 0.800 0.739 0.765 0.656 0.393 0.735 0.568 0.513 0.767 0.597 0.720 0.771 0.584 0.867 0.942 0.721 1.292 1.191 0.424 0.759 0.533 0.641 0.303 0.734 0.850 3.43

S. geminata 17 Guatemala, El Peten 32 0.220 0.839 0.744 0.683 0.706 0.583 0.347 0.711 0.536 0.509 0.693 0.589 0.680 0.680 0.554 0.825 0.883 0.730 1.267 1.152 0.396 0.688 0.507 0.639 0.303 0.757 0.797 3.39

S. geminata 17 Guatemala, El Peten 33 0.200 0.862 0.756 0.695 0.722 0.601 0.420 0.748 0.527 0.536 0.756 0.574 0.717 0.695 0.582 0.840 0.895 0.744 1.243 1.160 0.387 0.712 0.508 0.688 0.306 0.782 0.786 3.49

S. geminata 17 Guatemala, El Peten 34 0.190 0.832 0.754 0.709 0.722 0.634 0.350 0.743 0.519 0.539 0.730 0.562 0.706 0.728 0.545 0.823 0.906 0.722 1.246 1.163 0.445 0.741 0.507 0.645 0.289 0.746 0.769 3.39

S. geminata 17 Guatemala, El Peten 35 0.220 0.830 0.763 0.698 0.736 0.627 0.359 0.754 0.547 0.525 0.734 0.572 0.750 0.695 0.557 0.863 0.936 0.722 1.256 1.172 0.400 0.734 0.492 0.719 0.316 0.773 0.778 3.49

S. geminata 17 Guatemala, El Peten 36 0.290 0.929 0.857 0.796 0.821 0.701 0.401 0.801 0.577 0.547 0.830 0.653 0.793 0.793 0.633 0.901 0.975 0.803 1.367 1.317 0.467 0.809 0.569 0.747 0.328 0.888 0.893 3.88

S. geminata 17 Guatemala, El Peten 37 0.210 0.878 0.800 0.723 0.764 0.707 0.393 0.745 0.564 0.534 0.766 0.585 0.770 0.691 0.580 0.872 0.880 0.745 1.316 1.267 0.436 0.769 0.542 0.680 0.312 0.806 0.800 3.63

S. geminata 17 Guatemala, El Peten 38 0.190 0.817 0.734 0.678 0.705 0.606 0.425 0.701 0.508 0.543 0.691 0.571 0.684 0.700 0.537 0.817 0.880 0.689 1.189 1.135 0.393 0.673 0.498 0.674 0.295 0.743 0.766 3.37

S. geminata 17 Guatemala, El Peten 39 0.260 0.892 0.814 0.746 0.783 0.687 0.452 0.776 0.540 0.545 0.769 0.638 0.735 0.770 0.601 0.928 0.955 0.762 1.336 1.248 0.433 0.765 0.550 0.697 0.323 0.802 0.835 3.64

S. geminata 17 Guatemala, El Peten 40 0.230 0.900 0.767 0.711 0.743 0.634 0.425 0.744 0.574 0.517 0.753 0.582 0.739 0.742 0.564 0.879 0.922 0.733 1.274 1.206 0.406 0.733 0.514 0.672 0.312 0.728 0.772 3.51

S. geminata 18 Guatemala, El Peten 1 2.020 1.849 2.181 2.114 2.147 1.669 0.724 1.166 0.905 0.673 1.524 1.033 1.110 1.416 0.934 1.268 1.635 1.125 1.890 2.085 0.834 1.389 0.852 1.108 0.593 1.128 1.670 6.17

S. geminata 18 Guatemala, El Peten 2 1.700 1.752 2.029 1.997 1.965 1.603 0.908 1.102 0.870 0.691 1.425 0.988 1.082 1.191 0.930 1.276 1.571 1.096 1.805 2.015 0.789 1.276 0.858 0.967 0.566 1.178 1.483 5.91

S. geminata 18 Guatemala, El Peten 3 0.500 1.238 1.201 1.159 1.155 0.914 0.698 0.919 0.688 0.617 1.122 0.812 0.891 1.076 0.802 1.025 1.323 0.931 1.576 1.516 0.528 0.975 0.610 0.929 0.411 0.983 1.046 4.67

S. geminata 18 Guatemala, El Peten 4 0.630 1.290 1.259 1.199 1.222 0.989 0.676 0.978 0.741 0.665 1.200 0.854 0.868 1.147 0.807 1.133 1.371 0.962 1.670 1.561 0.582 0.996 0.628 0.900 0.441 0.958 1.149 4.71

S. geminata 18 Guatemala, El Peten 5 0.570 1.268 1.239 1.184 1.221 1.012 0.632 0.889 0.706 0.626 1.134 0.824 0.854 1.125 0.781 1.071 1.353 0.950 1.555 1.502 0.518 0.978 0.586 0.861 0.419 0.945 1.006 4.58

S. geminata 18 Guatemala, El Peten 6 0.520 1.202 1.165 1.068 1.114 0.897 0.606 0.879 0.694 0.610 1.122 0.752 0.839 1.060 0.785 1.022 1.258 0.938 1.505 1.470 0.501 0.921 0.586 0.828 0.392 0.878 0.978 4.38

S. geminata 18 Guatemala, El Peten 7 0.450 1.133 1.101 1.028 1.065 0.842 0.576 0.894 0.679 0.599 1.142 0.781 0.797 0.993 0.792 1.008 1.322 0.907 1.462 1.409 0.474 0.910 0.568 0.794 0.386 0.965 0.960 4.30

S. geminata 18 Guatemala, El Peten 8 0.320 1.054 0.975 0.892 0.957 0.73 0.500 0.836 0.640 0.594 1.008 0.740 0.802 0.956 0.715 0.961 1.176 0.911 1.424 1.382 0.469 0.850 0.545 0.775 0.363 0.861 0.915 4.07

S. geminata 18 Guatemala, El Peten 9 0.380 1.074 1.020 0.954 0.969 0.755 0.567 0.850 0.647 0.591 1.025 0.754 0.804 1.014 0.694 0.946 1.226 0.877 1.423 1.400 0.473 0.862 0.577 0.767 0.373 0.831 0.924 4.07

S. geminata 18 Guatemala, El Peten 10 0.360 1.082 1.013 0.934 0.953 0.763 0.542 0.864 0.633 0.571 1.005 0.726 0.779 0.966 0.734 0.929 1.214 0.862 1.483 1.369 0.501 0.855 0.571 0.842 0.363 0.906 0.933 4.20

S. geminata 18 Guatemala, El Peten 11 0.430 1.140 1.066 1.015 1.038 0.837 0.577 0.876 0.650 0.614 1.076 0.732 0.826 0.998 0.790 1.006 1.220 0.904 1.537 1.426 0.476 0.884 0.582 0.850 0.380 0.843 0.990 4.26

S. geminata 18 Guatemala, El Peten 12 0.390 1.078 1.014 0.935 0.990 0.782 0.581 0.833 0.642 0.599 1.027 0.773 0.777 1.014 0.732 0.949 1.216 0.862 1.422 1.409 0.483 0.876 0.579 0.808 0.387 0.901 0.956 4.20

S. geminata 18 Guatemala, El Peten 13 0.340 1.049 0.961 0.878 0.911 0.735 0.515 0.817 0.633 0.587 0.879 0.682 0.774 0.870 0.704 0.920 1.183 0.873 1.386 1.345 0.455 0.842 0.569 0.763 0.359 0.851 0.883 4.01

S. geminata 18 Guatemala, El Peten 14 0.330 1.031 0.944 0.870 0.921 0.749 0.513 0.836 0.616 0.592 0.969 0.714 0.765 0.920 0.673 0.863 1.127 0.849 1.382 1.334 0.478 0.849 0.508 0.778 0.353 0.826 0.904 3.97

S. geminata 18 Guatemala, El Peten 15 0.210 0.932 0.807 0.733 0.780 0.625 0.395 0.735 0.570 0.530 0.879 0.639 0.649 0.831 0.619 0.824 1.047 0.720 1.275 1.158 0.397 0.742 0.487 0.615 0.312 0.845 0.767 3.55

S. geminata 18 Guatemala, El Peten 16 0.140 0.791 0.676 0.615 0.630 0.541 0.352 0.653 0.461 0.504 0.761 0.555 0.606 0.645 0.519 0.696 0.887 0.670 1.062 1.027 0.335 0.641 0.432 0.617 0.284 0.606 0.657 3.04

S. geminata 18 Guatemala, El Peten 17 0.140 0.796 0.672 0.626 0.631 0.546 0.364 0.660 0.482 0.498 0.748 0.529 0.609 0.730 0.520 0.736 0.917 0.654 1.124 1.039 0.379 0.660 0.429 0.594 0.257 0.629 0.661 3.06

S. geminata 18 Guatemala, El Peten 18 0.140 0.777 0.673 0.645 0.667 0.563 0.400 0.656 0.475 0.518 0.811 0.511 0.589 0.744 0.497 0.703 0.920 0.660 1.127 1.053 0.350 0.665 0.442 0.632 0.273 0.617 0.631 3.08

S. geminata 18 Guatemala, El Peten 19 0.100 0.746 0.644 0.593 0.620 0.513 0.389 0.649 0.452 0.474 0.716 0.538 0.584 0.698 0.493 0.688 0.877 0.615 1.060 0.998 0.346 0.627 0.421 0.591 0.261 0.638 0.634 2.97

S. geminata 18 Guatemala, El Peten 20 0.130 0.802 0.654 0.612 0.635 0.535 0.366 0.665 0.491 0.486 0.804 0.582 0.572 0.717 0.533 0.714 0.901 0.699 1.045 1.021 0.358 0.643 0.421 0.642 0.261 0.626 0.653 3.09

S. geminata 18 Guatemala, El Peten 21 0.130 0.795 0.679 0.633 0.638 0.541 0.414 0.644 0.494 0.484 0.757 0.555 0.555 0.728 0.493 0.805 0.812 0.636 1.080 1.034 0.384 0.655 0.432 0.582 0.256 0.681 0.654 3.09

S. geminata 18 Guatemala, El Peten 22 0.120 0.740 0.648 0.587 0.625 0.532 0.360 0.632 0.475 0.491 0.731 0.548 0.658 0.674 0.526 0.686 0.900 0.637 1.083 1.048 0.377 0.642 0.402 0.648 0.262 0.722 0.680 3.16

S. geminata 18 Guatemala, El Peten 23 0.110 0.768 0.635 0.588 0.603 0.5 0.380 0.636 0.463 0.490 0.716 0.518 0.555 0.692 0.526 0.672 0.868 0.636 1.058 0.976 0.309 0.596 0.411 0.576 0.238 0.535 0.681 2.86

S. geminata 18 Guatemala, El Peten 24 0.130 0.749 0.638 0.592 0.606 0.523 0.377 0.619 0.431 0.474 0.715 0.496 0.559 0.702 0.489 0.726 0.874 0.592 1.075 0.978 0.329 0.635 0.416 0.603 0.237 0.616 0.643 2.95

S. geminata 18 Guatemala, El Peten 25 0.140 0.819 0.704 0.634 0.680 0.555 0.415 0.695 0.483 0.513 0.809 0.545 0.644 0.759 0.577 0.727 0.933 0.705 1.119 1.078 0.342 0.672 0.448 0.615 0.275 0.662 0.643 3.17

S. geminata 18 Guatemala, El Peten 26 0.130 0.767 0.651 0.605 0.623 0.555 0.364 0.641 0.453 0.498 0.702 0.563 0.555 0.668 0.486 0.641 0.862 0.584 1.047 0.986 0.315 0.623 0.398 0.606 0.263 0.585 0.594 2.94

S. geminata 18 Guatemala, El Peten 27 0.120 0.763 0.624 0.582 0.601 0.522 0.402 0.652 0.426 0.474 0.771 0.549 0.589 0.668 0.470 0.711 0.867 0.611 1.035 0.962 0.312 0.588 0.414 0.604 0.233 0.647 0.641 2.98

S. geminata 18 Guatemala, El Peten 28 0.140 0.815 0.681 0.635 0.657 0.555 0.391 0.674 0.492 0.509 0.741 0.584 0.597 0.637 0.534 0.755 0.802 0.650 1.159 1.067 0.351 0.654 0.441 0.593 0.277 0.695 0.655 3.17

S. geminata 18 Guatemala, El Peten 29 0.100 0.715 0.594 0.547 0.572 0.495 0.354 0.620 0.433 0.465 0.656 0.490 0.544 0.630 0.455 0.653 0.806 0.583 0.992 0.942 0.303 0.558 0.411 0.528 0.245 0.641 0.684 2.83

S. geminata 18 Guatemala, El Peten 30 0.140 0.772 0.670 0.610 0.647 0.536 0.386 0.658 0.488 0.490 0.773 0.550 0.605 0.738 0.522 0.697 0.903 0.643 1.096 1.053 0.332 0.665 0.417 0.597 0.249 0.694 0.657 3.12

S. geminata 18 Guatemala, El Peten 31 0.120 0.731 0.620 0.569 0.578 0.485 0.366 0.603 0.440 0.471 0.674 0.496 0.608 0.654 0.452 0.645 0.818 0.638 0.946 0.957 0.317 0.586 0.420 0.583 0.233 0.542 0.597 2.81

S. geminata 18 Guatemala, El Peten 32 0.180 0.818 0.726 0.661 0.684 0.568 0.417 0.710 0.495 0.520 0.829 0.615 0.637 0.778 0.584 0.747 0.998 0.724 1.163 1.095 0.350 0.684 0.450 0.682 0.291 0.634 0.729 3.23

S. geminata 18 Guatemala, El Peten 33 0.110 0.750 0.634 0.597 0.587 0.513 0.351 0.634 0.464 0.471 0.725 0.469 0.618 0.681 0.514 0.678 0.879 0.595 1.041 0.977 0.315 0.609 0.393 0.586 0.245 0.606 0.592 2.92

S. geminata 18 Guatemala, El Peten 34 0.110 0.745 0.653 0.593 0.611 0.518 0.366 0.626 0.467 0.479 0.729 0.509 0.588 0.714 0.495 0.693 0.865 0.612 1.063 0.987 0.335 0.609 0.423 0.570 0.243 0.587 0.628 2.89

S. geminata 18 Guatemala, El Peten 35 0.110 0.705 0.612 0.580 0.590 0.492 0.354 0.625 0.450 0.472 0.735 0.521 0.588 0.692 0.493 0.663 0.860 0.582 1.012 0.964 0.318 0.590 0.426 0.529 0.253 0.588 0.606 2.79

S. geminata 18 Guatemala, El Peten 36 0.150 0.786 0.652 0.629 0.656 0.541 0.382 0.665 0.482 0.512 0.813 0.544 0.609 0.738 0.524 0.747 0.907 0.642 1.150 1.074 0.360 0.664 0.448 0.597 0.275 0.653 0.666 3.11

S. geminata 18 Guatemala, El Peten 37 0.120 0.753 0.656 0.615 0.624 0.541 0.389 0.634 0.477 0.471 0.775 0.555 0.583 0.693 0.494 0.700 0.886 0.594 1.089 1.014 0.356 0.626 0.450 0.548 0.258 0.603 0.606 2.92

S. geminata 18 Guatemala, El Peten 38 0.100 0.763 0.642 0.587 0.596 0.49 0.363 0.612 0.456 0.473 0.759 0.509 0.548 0.680 0.483 0.678 0.846 0.618 1.023 0.986 0.342 0.597 0.409 0.564 0.247 0.556 0.625 2.87

S. geminata 18 Guatemala, El Peten 39 0.130 0.728 0.639 0.597 0.607 0.522 0.376 0.612 0.451 0.473 0.751 0.497 0.553 0.588 0.467 0.697 0.844 0.596 1.025 0.994 0.350 0.649 0.393 0.564 0.230 0.572 0.616 2.86

S. geminata 18 Guatemala, El Peten 40 0.120 0.736 0.640 0.579 0.612 0.514 0.389 0.640 0.451 0.483 0.719 0.482 0.579 0.696 0.520 0.678 0.849 0.728 0.988 1.016 0.337 0.609 0.435 0.610 0.260 0.602 0.625 2.96

S. geminata 18 Guatemala, El Peten 41 0.130 0.765 0.650 0.586 0.614 0.513 0.376 0.641 0.452 0.489 0.750 0.510 0.578 0.717 0.494 0.675 0.856 0.607 1.025 1.022 0.361 0.631 0.421 0.600 0.260 0.606 0.646 2.99

S. geminata 18 Guatemala, El Peten 42 0.110 0.724 0.626 0.589 0.616 0.513 0.374 0.622 0.477 0.469 0.706 0.503 0.575 0.600 0.510 0.659 0.853 0.585 1.018 0.984 0.295 0.629 0.392 0.565 0.239 0.604 0.593 2.88

S. geminata 18 Guatemala, El Peten 43 0.110 0.777 0.629 0.583 0.606 0.508 0.332 0.623 0.474 0.461 0.711 0.498 0.571 0.693 0.482 0.667 0.852 0.576 1.013 0.973 0.334 0.569 0.412 0.572 0.221 0.596 0.594 2.92

S. geminata 18 Guatemala, El Peten 44 0.110 0.721 0.638 0.587 0.620 0.518 0.376 0.611 0.449 0.472 0.697 0.515 0.547 0.666 0.473 0.677 0.834 0.592 1.002 0.974 0.337 0.595 0.403 0.562 0.252 0.619 0.594 2.88

S. geminata 18 Guatemala, El Peten 45 0.110 0.754 0.620 0.592 0.592 0.49 0.340 0.617 0.431 0.464 0.690 0.488 0.576 0.672 0.482 0.661 0.822 0.587 0.996 0.938 0.322 0.564 0.389 0.570 0.245 0.594 0.552 2.85

S. geminata 18 Guatemala, El Peten 46 0.080 0.702 0.621 0.562 0.585 0.492 0.333 0.615 0.440 0.472 0.700 0.489 0.554 0.655 0.465 0.650 0.827 0.546 0.996 0.961 0.330 0.593 0.407 0.573 0.228 0.564 0.590 2.80

S. geminata 18 Guatemala, El Peten 47 0.100 0.714 0.630 0.584 0.593 0.501 0.360 0.609 0.448 0.446 0.712 0.499 0.514 0.665 0.487 0.625 0.849 0.598 0.982 0.967 0.327 0.597 0.399 0.542 0.247 0.611 0.574 2.83

S. geminata 18 Guatemala, El Peten 48 0.100 0.736 0.641 0.590 0.603 0.509 0.376 0.622 0.444 0.471 0.755 0.522 0.558 0.595 0.503 0.678 0.836 0.622 1.002 0.961 0.322 0.607 0.377 0.613 0.249 0.612 0.570 2.92

S. geminata 18 Guatemala, El Peten 49 0.100 0.763 0.629 0.606 0.597 0.508 0.348 0.641 0.462 0.457 0.722 0.529 0.561 0.619 0.518 0.665 0.869 0.615 1.037 1.022 0.330 0.621 0.450 0.512 0.262 0.594 0.627 2.89

S. geminata 18 Guatemala, El Peten 50 0.110 0.745 0.652 0.610 0.624 0.522 0.380 0.641 0.468 0.492 0.746 0.583 0.556 0.633 0.534 0.717 0.873 0.659 1.047 1.042 0.339 0.628 0.465 0.566 0.261 0.638 0.638 2.99

S. geminata 22 Guatemala, El Peten 1 0.260 0.757 0.723 0.680 0.701 0.621 0.372 0.689 0.511 0.469 0.668 0.547 0.683 0.691 0.520 0.793 0.865 0.704 1.161 1.089 0.380 0.672 0.474 0.681 0.287 0.711 0.682 3.24

S. geminata 22 Guatemala, El Peten 2 0.230 0.791 0.661 0.624 0.642 0.531 0.374 0.678 0.474 0.475 0.664 0.526 0.612 0.631 0.492 0.765 0.808 0.619 1.160 1.053 0.348 0.640 0.456 0.587 0.262 0.631 0.651 3.06

S. geminata 22 Guatemala, El Peten 3 0.130 0.770 0.667 0.629 0.632 0.516 0.359 0.640 0.453 0.481 0.641 0.527 0.638 0.594 0.505 0.711 0.786 0.628 1.090 1.029 0.330 0.651 0.456 0.584 0.264 0.613 0.640 3.00

S. geminata 22 Guatemala, El Peten 4 0.350 1.070 1.054 1.026 1.042 0.862 0.557 0.878 0.647 0.611 0.938 0.699 0.840 0.911 0.678 1.037 1.074 0.853 1.550 1.410 0.495 0.886 0.616 0.773 0.396 0.898 0.950 4.15

S. geminata 22 Guatemala, El Peten 5 0.180 0.797 0.694 0.634 0.669 0.612 0.364 0.681 0.548 0.471 0.688 0.541 0.668 0.636 0.513 0.795 0.835 0.660 1.182 1.110 0.385 0.668 0.497 0.586 0.278 0.652 0.699 3.15

S. geminata 22 Guatemala, El Peten 6 0.100 0.688 0.645 0.608 0.602 0.515 0.333 0.629 0.426 0.475 0.624 0.467 0.629 0.623 0.480 0.700 0.769 0.602 1.073 0.993 0.365 0.606 0.454 0.507 0.250 0.609 0.578 2.80

S. geminata 22 Guatemala, El Peten 7 0.140 0.750 0.653 0.619 0.613 0.564 0.303 0.602 0.432 0.461 0.604 0.475 0.623 0.560 0.448 0.711 0.761 0.565 1.029 0.976 0.347 0.602 0.427 0.561 0.235 0.605 0.664 2.89

S. geminata 22 Guatemala, El Peten 8 0.140 0.743 0.645 0.618 0.618 0.521 0.348 0.648 0.480 0.451 0.640 0.517 0.603 0.598 0.493 0.702 0.764 0.645 1.065 1.041 0.370 0.705 0.448 0.528 0.266 0.575 0.645 2.89

S. geminata 22 Guatemala, El Peten 9 0.150 0.672 0.602 0.559 0.569 0.493 0.357 0.591 0.403 0.460 0.568 0.481 0.559 0.584 0.450 0.649 0.700 0.573 0.996 0.956 0.327 0.591 0.421 0.512 0.250 0.597 0.618 2.74

S. geminata 22 Guatemala, El Peten 10 0.160 0.787 0.717 0.673 0.683 0.587 0.396 0.679 0.505 0.490 0.633 0.544 0.659 0.643 0.505 0.773 0.822 0.657 1.159 1.092 0.402 0.669 0.493 0.553 0.282 0.688 0.650 3.12

S. geminata 22 Guatemala, El Peten 11 0.150 0.803 0.707 0.669 0.667 0.594 0.357 0.691 0.492 0.502 0.664 0.531 0.698 0.656 0.535 0.795 0.826 0.658 1.182 1.113 0.412 0.665 0.497 0.650 0.286 0.724 0.684 3.29

S. geminata 22 Guatemala, El Peten 12 0.110 0.754 0.662 0.629 0.615 0.570 0.336 0.623 0.495 0.464 0.634 0.549 0.623 0.623 0.477 0.759 0.788 0.633 1.103 0.990 0.322 0.619 0.434 0.510 0.260 0.661 0.629 2.92

S. geminata 22 Guatemala, El Peten 13 0.160 0.836 0.728 0.690 0.705 0.587 0.419 0.711 0.537 0.472 0.705 0.581 0.664 0.714 0.564 0.783 0.871 0.705 1.200 1.133 0.390 0.675 0.534 0.599 0.309 0.694 0.721 3.26

S. geminata 22 Guatemala, El Peten 14 0.220 0.949 0.841 0.791 0.803 0.721 0.473 0.765 0.564 0.530 0.725 0.614 0.729 0.718 0.606 0.876 0.948 0.752 1.293 1.220 0.412 0.747 0.544 0.619 0.304 0.779 0.757 3.57

S. geminata 22 Guatemala, El Peten 15 0.170 0.829 0.737 0.683 0.683 0.618 0.371 0.724 0.535 0.473 0.710 0.573 0.659 0.714 0.555 0.793 0.863 0.699 1.178 1.162 0.375 0.680 0.530 0.606 0.291 0.685 0.691 3.28

S. geminata 22 Guatemala, El Peten 16 0.390 1.106 1.068 1.030 1.014 0.895 0.559 0.870 0.563 0.597 0.910 0.699 0.801 0.793 0.680 0.953 1.090 0.851 1.415 1.436 0.483 0.854 0.612 0.779 0.376 0.915 0.942 4.24

S. geminata 22 Guatemala, El Peten 17 0.080 0.699 0.618 0.569 0.575 0.510 0.358 0.603 0.446 0.420 0.607 0.491 0.571 0.603 0.454 0.687 0.728 0.576 1.024 0.949 0.320 0.577 0.423 0.527 0.239 0.594 0.577 2.77

S. geminata 22 Guatemala, El Peten 18 0.190 0.889 0.792 0.732 0.775 0.656 0.396 0.754 0.569 0.529 0.756 0.584 0.723 0.744 0.590 0.848 0.939 0.695 1.276 1.188 0.393 0.683 0.546 0.629 0.314 0.722 0.754 3.43

S. geminata 22 Guatemala, El Peten 19 0.100 0.695 0.591 0.569 0.58 0.504 0.337 0.586 0.444 0.439 0.596 0.456 0.561 0.561 0.441 0.681 0.704 0.575 0.990 0.937 0.311 0.562 0.410 0.503 0.240 0.542 0.580 2.68

S. geminata 22 Guatemala, El Peten 20 0.200 0.728 0.567 0.498 0.764 0.609 0.701 0.698 0.580 0.830 0.884 0.738 1.249 1.225 0.456 0.751 0.529 0.645 0.299 0.786 0.772

S. geminata 22 Guatemala, El Peten 21 1.180 1.575 1.678 1.600 1.676 1.349 0.579 1.025 0.753 0.610 1.106 0.843 0.971 1.067 0.863 1.161 1.294 1.031 1.694 1.802 0.645 1.142 0.789 0.807 0.512 1.176 1.270 5.36

S. geminata 22 Guatemala, El Peten 22 1.470 1.670 1.800 1.781 1.778 1.462 0.606 1.083 0.834 0.664 1.166 0.930 1.042 1.158 0.911 1.258 1.348 1.057 1.821 1.864 0.733 1.206 0.820 0.834 0.527 1.318 1.298 5.69

S. geminata 22 Guatemala, El Peten 23 0.880 1.285 1.328 1.279 1.264 1.062 0.593 0.970 0.730 0.655 1.106 0.806 0.950 1.066 0.831 1.146 1.258 1.006 1.719 1.655 0.636 1.051 0.710 0.809 0.440 1.161 1.166 4.91

S. geminata 22 Guatemala, El Peten 24 0.810 1.332 1.370 1.293 1.318 1.069 0.528 0.971 0.708 0.588 1.051 0.823 0.945 1.028 0.781 1.134 1.234 0.959 1.639 1.616 0.640 1.019 0.710 0.747 0.439 0.987 1.122 4.68

S. geminata 22 Guatemala, El Peten 25 0.580 1.221 1.177 1.111 1.135 0.971 0.539 0.890 0.701 0.585 0.997 0.759 0.867 0.964 0.755 1.057 1.145 0.949 1.541 1.518 0.564 0.951 0.665 0.688 0.399 0.908 1.004 4.34

S. geminata 22 Guatemala, El Peten 26 0.600 1.157 1.130 1.076 1.086 0.936 0.543 0.929 0.640 0.636 1.006 0.788 0.898 0.957 0.741 1.099 1.143 0.946 1.613 1.544 0.566 0.965 0.675 0.841 0.422 1.137 1.089 4.68

S. geminata 22 Guatemala, El Peten 27 0.470 1.184 1.129 1.053 1.111 0.922 0.556 0.905 0.671 0.568 0.966 0.751 0.857 0.955 0.732 1.050 1.138 0.880 1.516 1.504 0.538 0.910 0.645 0.764 0.412 0.954 0.954 4.41

S. geminata 22 Guatemala, El Peten 28 0.700 1.324 1.329 1.281 1.296 1.135 0.600 0.946 0.710 0.606 1.051 0.785 0.908 1.009 0.769 1.095 1.161 0.943 1.628 1.602 0.600 0.972 0.675 0.810 0.461 0.981 1.107 4.72

S. geminata 22 Guatemala, El Peten 29 0.590 1.258 1.263 1.209 1.241 1.073 0.584 0.916 0.674 0.599 0.996 0.738 0.862 0.959 0.732 1.069 1.129 0.881 1.500 1.554 0.565 0.956 0.662 0.809 0.412 1.005 1.058 4.63

S. geminata 22 Guatemala, El Peten 30 0.540 1.151 1.079 1.036 1.041 0.916 0.525 0.909 0.663 0.586 0.986 0.758 0.874 0.945 0.742 1.043 1.127 0.886 1.544 1.480 0.515 0.919 0.657 0.735 0.392 0.966 0.993 4.33

S. geminata 22 Guatemala, El Peten 31 0.110 0.717 0.610 0.572 0.586 0.506 0.368 0.621 0.416 0.472 0.601 0.459 0.571 0.559 0.458 0.672 0.599 0.567 0.999 0.959 0.317 0.581 0.412 0.571 0.240 0.645 0.607 2.89

S. geminata 22 Guatemala, El Peten 32 0.070 0.689 0.581 0.555 0.547 0.468 0.328 0.573 0.420 0.442 0.583 0.432 0.551 0.581 0.437 0.660 0.646 0.548 0.959 0.902 0.331 0.538 0.412 0.511 0.235 0.591 0.559 2.69

S. geminata 22 Guatemala, El Peten 33 0.080 0.662 0.585 0.547 0.548 0.472 0.326 0.574 0.409 0.435 0.565 0.415 0.580 0.537 0.456 0.638 0.671 0.569 0.951 0.907 0.315 0.571 0.411 0.497 0.243 0.617 0.550 2.68

S. geminata 22 Guatemala, El Peten 34 0.070 0.716 0.598 0.555 0.559 0.506 0.350 0.582 0.444 0.478 0.591 0.461 0.560 0.512 0.468 0.675 0.722 0.557 1.019 0.921 0.320 0.549 0.410 0.514 0.239 0.608 0.587 2.76

S. geminata 22 Guatemala, El Peten 35 0.080 0.716 0.634 0.583 0.591 0.535 0.332 0.619 0.453 0.449 0.603 0.489 0.587 0.604 0.481 0.708 0.738 0.578 1.046 0.961 0.337 0.596 0.405 0.539 0.245 0.603 0.624 2.82

S. geminata 22 Guatemala, El Peten 36 0.100 0.721 0.607 0.580 0.569 0.504 0.369 0.599 0.451 0.433 0.603 0.457 0.574 0.589 0.494 0.693 0.725 0.590 1.016 0.993 0.337 0.587 0.424 0.512 0.256 0.618 0.597 2.84

S. geminata 22 Guatemala, El Peten 37 0.100 0.721 0.591 0.542 0.569 0.489 0.345 0.583 0.444 0.439 0.587 0.430 0.628 0.550 0.445 0.683 0.720 0.564 0.959 0.953 0.308 0.576 0.407 0.532 0.260 0.558 0.568 2.76

S. geminata 22 Guatemala, El Peten 38 0.100 0.718 0.626 0.577 0.588 0.502 0.391 0.620 0.473 0.446 0.612 0.514 0.569 0.599 0.505 0.678 0.763 0.637 1.049 0.991 0.336 0.599 0.447 0.510 0.266 0.640 0.640 2.86

S. geminata 22 Guatemala, El Peten 39 0.090 0.715 0.632 0.589 0.601 0.523 0.357 0.611 0.441 0.462 0.582 0.480 0.614 0.647 0.440 0.731 0.717 0.597 1.046 0.969 0.338 0.612 0.401 0.550 0.265 0.558 0.577 2.79

S. geminata 22 Guatemala, El Peten 40 0.090 0.706 0.613 0.577 0.586 0.502 0.323 0.597 0.442 0.439 0.622 0.528 0.528 0.605 0.454 0.763 0.736 0.578 1.020 0.938 0.320 0.595 0.417 0.514 0.254 0.544 0.578 2.70

S. richteri 41 Saltillom, MS, lab colony 1 1.060 1.419 1.414 1.414 1.299 1.063 0.791 1.050 0.825 0.671 1.358 0.968 0.940 1.351 0.934 1.164 1.547 1.137 1.768 1.999 0.765 1.291 0.838 1.071 0.539 1.255 1.721 5.74

S. richteri 41 Saltillom, MS, lab colony 2 1.140 1.332 1.326 1.322 1.244 1.031 0.750 1.035 0.798 0.661 1.329 0.955 0.932 1.308 0.927 1.251 1.538 1.122 1.839 1.856 0.683 1.231 0.743 1.042 0.506 1.126 1.564 5.36

S. richteri 41 Saltillom, MS, lab colony 3 0.730 1.285 1.257 1.229 1.179 0.963 0.641 0.985 0.744 0.665 1.251 0.885 0.973 1.244 0.901 1.216 1.490 1.109 1.830 1.732 0.625 1.117 0.707 0.965 0.480 1.084 1.421 5.07

S. richteri 41 Saltillom, MS, lab colony 4 0.850 1.346 1.305 1.314 1.227 1.024 0.728 1.039 0.813 0.657 1.314 0.942 0.969 1.312 0.923 1.260 1.557 1.126 1.909 1.873 0.674 1.188 0.798 0.975 0.476 1.162 1.517 5.36

S. richteri 41 Saltillom, MS, lab colony 5 0.840 1.327 1.300 1.309 1.199 1.008 0.701 1.008 0.770 0.661 1.291 1.007 0.979 1.304 0.975 1.245 1.555 1.113 1.867 1.859 0.724 1.187 0.764 0.986 0.485 1.203 1.481 5.37

S. richteri 41 Saltillom, MS, lab colony 6 0.890 1.326 1.326 1.326 1.243 1.022 0.704 1.030 0.805 0.667 1.161 0.899 1.017 1.306 0.928 1.270 1.558 1.134 1.917 1.885 0.666 1.224 0.787 0.975 0.469 1.080 1.513 5.27

S. richteri 41 Saltillom, MS, lab colony 7 0.870 1.263 1.239 1.239 1.151 0.981 0.691 0.956 0.713 0.636 1.226 0.912 0.908 1.199 0.856 1.161 1.449 1.072 1.749 1.716 0.620 1.092 0.703 0.930 0.464 1.004 1.353 4.91

S. richteri 41 Saltillom, MS, lab colony 8 0.830 1.337 1.333 1.300 1.241 1.005 0.690 1.029 0.769 0.686 1.294 0.963 1.023 1.291 0.987 1.313 1.559 1.161 1.943 1.816 0.688 1.184 0.753 0.941 0.489 1.106 1.498 5.20

S. richteri 41 Saltillom, MS, lab colony 9 0.660 1.273 1.223 1.223 1.136 0.922 0.642 0.959 0.734 0.638 1.092 0.855 0.954 1.231 0.871 1.231 1.470 1.052 1.791 1.704 0.642 1.075 0.693 0.881 0.456 0.995 1.377 4.85

S. richteri 41 Saltillom, MS, lab colony 10 0.690 1.252 1.212 1.175 1.117 0.953 0.693 0.954 0.707 0.651 1.192 0.826 0.966 1.196 0.877 1.190 1.426 1.088 1.788 1.655 0.658 1.085 0.668 0.995 0.467 1.098 1.344 5.00

S. richteri 41 Saltillom, MS, lab colony 11 0.570 1.217 1.150 1.140 1.067 0.892 0.640 0.914 0.691 0.618 1.140 0.865 0.867 1.155 0.846 1.088 1.374 0.970 1.679 1.687 0.612 1.076 0.728 0.917 0.446 1.007 1.360 4.83

S. richteri 41 Saltillom, MS, lab colony 12 0.780 1.227 1.205 1.195 1.108 0.923 0.659 0.960 0.746 0.626 1.171 0.775 0.923 1.174 0.867 1.138 1.408 1.023 1.703 1.615 0.614 1.058 0.660 0.968 0.454 0.961 1.337 4.77

S. richteri 41 Saltillom, MS, lab colony 13 0.740 1.227 1.195 1.181 1.113 0.916 0.650 0.955 0.745 0.626 1.177 0.827 0.916 1.198 0.828 1.168 1.436 1.021 1.694 1.664 0.516 1.071 0.665 0.863 0.440 1.086 1.290 4.84

S. richteri 41 Saltillom, MS, lab colony 14 0.460 1.110 0.995 0.986 0.954 0.797 0.565 0.868 0.638 0.579 0.920 0.680 0.824 1.038 0.726 1.021 1.276 0.907 1.514 1.443 0.537 0.945 0.581 0.837 0.377 0.920 1.094 4.31

S. richteri 41 Saltillom, MS, lab colony 15 0.280 0.977 0.885 0.835 0.848 0.715 0.501 0.784 0.576 0.547 0.953 0.665 0.794 0.827 0.654 0.919 1.138 0.791 1.408 1.308 0.447 0.831 0.550 0.774 0.341 0.814 0.984 3.87

S. richteri 41 Saltillom, MS, lab colony 16 0.210 0.890 0.776 0.736 0.741 0.666 0.470 0.698 0.520 0.513 0.832 0.584 0.686 0.821 0.558 0.818 1.009 0.717 1.261 1.184 0.401 0.752 0.495 0.703 0.284 0.744 0.825 3.52

S. richteri 41 Saltillom, MS, lab colony 17 0.170 0.895 0.779 0.728 0.730 0.656 0.471 0.716 0.539 0.508 0.839 0.575 0.676 0.819 0.599 0.819 0.917 0.710 1.283 1.141 0.315 0.725 0.482 0.698 0.290 0.701 0.824 3.43

S. richteri 41 Saltillom, MS, lab colony 18 0.220 0.902 0.792 0.737 0.774 0.648 0.525 0.673 0.496 0.486 0.881 0.607 0.713 0.860 0.603 0.917 1.063 0.740 1.308 1.254 0.447 0.787 0.507 0.672 0.323 0.820 0.911 3.65

S. richteri 41 Saltillom, MS, lab colony 19 0.180 0.827 0.716 0.665 0.672 0.597 0.431 0.673 0.496 0.486 0.784 0.511 0.630 0.762 0.520 0.758 0.950 0.657 1.154 1.145 0.385 0.708 0.479 0.641 0.287 0.684 0.736 3.30

S. richteri 41 Saltillom, MS, lab colony 20 0.120 0.862 0.770 0.723 0.719 0.61 0.411 0.697 0.532 0.498 0.834 0.591 0.674 0.801 0.577 0.823 0.992 0.712 1.189 1.134 0.440 0.742 0.453 0.711 0.312 0.718 0.799 3.43

S. richteri 41 Saltillom, MS, lab colony 21 0.240 0.913 0.803 0.770 0.765 0.641 0.454 0.736 0.560 0.505 0.862 0.626 0.686 0.754 0.587 0.803 1.060 0.736 1.243 1.216 0.443 0.786 0.484 0.717 0.318 0.767 0.888 3.61

S. richteri 41 Saltillom, MS, lab colony 22 0.220 0.881 0.756 0.733 0.737 0.688 0.470 0.729 0.536 0.503 0.853 0.572 0.708 0.830 0.603 0.843 1.023 0.742 1.268 1.200 0.451 0.763 0.493 0.727 0.307 0.675 0.884 3.48

S. richteri 41 Saltillom, MS, lab colony 23 0.120 0.806 0.681 0.668 0.691 0.583 0.426 0.666 0.479 0.478 0.769 0.510 0.630 0.760 0.507 0.794 0.932 0.640 1.170 1.068 0.406 0.697 0.421 0.671 0.271 0.679 0.720 3.22

S. richteri 41 Saltillom, MS, lab colony 24 0.130 0.712 0.602 0.563 0.593 0.543 0.348 0.565 0.420 0.408 0.627 0.414 0.523 0.555 0.432 0.599 0.762 0.529 0.956 0.972 0.317 0.625 0.387 0.584 0.250 0.632 0.644 2.90

S. richteri 41 Saltillom, MS, lab colony 25 0.180 0.751 0.644 0.608 0.617 0.55 0.381 0.605 0.463 0.425 0.704 0.500 0.569 0.610 0.460 0.714 0.864 0.600 1.050 1.002 0.360 0.631 0.402 0.670 0.266 0.641 0.708 3.06

S. richteri 41 Saltillom, MS, lab colony 26 0.160 0.792 0.665 0.638 0.649 0.581 0.410 0.636 0.469 0.456 0.740 0.507 0.580 0.708 0.478 0.747 0.889 0.635 1.068 1.073 0.360 0.680 0.444 0.611 0.262 0.707 0.730 3.18

S. richteri 41 Saltillom, MS, lab colony 27 0.130 0.741 0.642 0.614 0.614 0.515 0.390 0.605 0.436 0.463 0.686 0.477 0.558 0.671 0.460 0.707 0.861 0.620 0.998 1.007 0.383 0.640 0.406 0.599 0.258 0.633 0.656 2.98

S. richteri 41 Saltillom, MS, lab colony 28 0.150 0.723 0.626 0.612 0.603 0.546 0.389 0.604 0.461 0.442 0.689 0.490 0.523 0.595 0.449 0.691 0.843 0.589 1.062 1.005 0.335 0.663 0.399 0.615 0.261 0.609 0.647 2.95

S. richteri 41 Saltillom, MS, lab colony 29 0.100 0.744 0.632 0.599 0.603 0.526 0.370 0.597 0.447 0.432 0.601 0.445 0.585 0.580 0.464 0.665 0.809 0.592 1.021 0.986 0.339 0.633 0.381 0.597 0.243 0.602 0.638 2.93

S. richteri 41 Saltillom, MS, lab colony 30 0.110 0.740 0.624 0.602 0.611 0.539 0.401 0.609 0.421 0.449 0.698 0.477 0.542 0.665 0.480 0.731 0.855 0.599 1.049 0.999 0.361 0.654 0.390 0.630 0.250 0.628 0.646 3.00

S. richteri 41 Saltillom, MS, lab colony 31 0.100 0.772 0.653 0.625 0.634 0.578 0.402 0.629 0.452 0.453 0.709 0.497 0.595 0.695 0.503 0.686 0.838 0.631 1.038 1.040 0.364 0.662 0.410 0.600 0.252 0.599 0.709 3.01

S. richteri 41 Saltillom, MS, lab colony 32 0.100 0.700 0.590 0.571 0.550 0.523 0.358 0.555 0.401 0.412 0.653 0.481 0.525 0.541 0.432 0.649 0.773 0.532 0.956 0.954 0.357 0.613 0.358 0.595 0.234 0.631 0.656 2.88

S. richteri 41 Saltillom, MS, lab colony 33 0.090 0.745 0.621 0.589 0.607 0.542 0.380 0.599 0.429 0.437 0.654 0.487 0.578 0.649 0.433 0.687 0.790 0.595 1.031 1.004 0.372 0.648 0.384 0.596 0.255 0.612 0.695 2.96

S. richteri 41 Saltillom, MS, lab colony 34 0.090 0.742 0.603 0.585 0.594 0.482 0.340 0.635 0.425 0.427 0.667 0.484 0.523 0.656 0.419 0.692 0.803 0.557 1.006 1.001 0.381 0.623 0.409 0.536 0.224 0.553 0.632 2.83

S. richteri 41 Saltillom, MS, lab colony 35 0.090 0.722 0.613 0.576 0.600 0.541 0.371 0.592 0.408 0.441 0.676 0.486 0.533 0.590 0.455 0.637 0.808 0.589 1.023 0.996 0.371 0.623 0.409 0.588 0.255 0.629 0.679 2.94

S. richteri 41 Saltillom, MS, lab colony 36 0.090 0.701 0.603 0.575 0.584 0.509 0.358 0.577 0.417 0.434 0.647 0.436 0.539 0.562 0.416 0.650 0.786 0.531 0.994 0.952 0.342 0.626 0.390 0.593 0.258 0.515 0.652 2.76

S. richteri 41 Saltillom, MS, lab colony 37 0.080 0.687 0.582 0.573 0.552 0.519 0.340 0.564 0.384 0.425 0.635 0.454 0.509 0.618 0.428 0.627 0.757 0.515 0.974 0.942 0.340 0.600 0.387 0.577 0.252 0.557 0.689 2.76

S. richteri 41 Saltillom, MS, lab colony 38 0.080 0.681 0.571 0.547 0.553 0.482 0.333 0.530 0.391 0.429 0.617 0.458 0.507 0.526 0.396 0.642 0.758 0.521 0.986 0.896 0.348 0.586 0.361 0.612 0.247 0.484 0.620 2.67

S. richteri 41 Saltillom, MS, lab colony 39 0.070 0.715 0.642 0.613 0.610 0.552 0.403 0.604 0.410 0.439 0.661 0.453 0.560 0.678 0.449 0.685 0.829 0.590 1.037 0.967 0.379 0.623 0.402 0.602 0.263 0.557 0.663 2.84

S. richteri 41 Saltillom, MS, lab colony 40 0.090 0.699 0.576 0.556 0.580 0.516 0.380 0.582 0.396 0.447 0.653 0.440 0.522 0.575 0.413 0.666 0.777 0.553 0.990 0.936 0.348 0.595 0.368 0.587 0.236 0.563 0.612 2.79

S. interrupta 42 Argentina, Santiago del Estero, Va San Martin 1 1.310 1.523 1.500 1.517 1.366 1.273 0.747 1.125 0.848 0.728 1.452 1.002 1.099 1.413 1.065 1.354 1.726 1.262 2.095 2.087 0.890 1.352 0.926 1.050 0.596 1.393 1.731 6.05

S. interrupta 42 Argentina, Santiago del Estero, Va San Martin 2 1.200 1.527 1.527 1.571 1.326 1.204 0.678 1.129 0.815 0.781 1.409 0.996 1.084 1.445 1.005 1.386 1.685 1.222 2.121 1.969 0.846 1.335 0.909 1.030 0.552 1.396 1.812 5.92

S. interrupta 42 Argentina, Santiago del Estero, Va San Martin 3 1.180 1.463 1.432 1.441 1.27 1.191 0.553 1.103 0.793 0.719 1.390 1.003 1.025 1.448 0.924 1.351 1.689 1.194 2.078 1.961 0.815 1.275 0.859 1.005 0.550 1.387 1.603 5.82

S. interrupta 42 Argentina, Santiago del Estero, Va San Martin 4 0.850 1.340 1.339 1.313 1.205 1.013 0.596 1.046 0.814 0.662 1.305 0.914 1.026 1.339 0.910 1.284 1.623 1.169 1.975 1.764 0.642 1.126 0.734 1.079 0.507 1.218 1.430 5.40

S. interrupta 42 Argentina, Santiago del Estero, Va San Martin 5 0.690 1.288 1.364 1.283 1.173 1.009 0.568 1.045 0.775 0.638 1.231 0.892 0.967 1.287 0.874 1.233 1.534 1.102 1.790 1.706 0.635 1.078 0.720 0.927 0.467 1.132 1.303 5.05

S. interrupta 42 Argentina, Santiago del Estero, Va San Martin 6 0.630 1.260 1.202 1.177 1.09 0.927 0.532 1.018 0.781 0.647 1.234 0.856 0.935 1.255 0.840 1.240 1.489 1.095 1.799 1.630 0.610 1.027 0.709 0.902 0.459 1.142 1.299 4.93

S. interrupta 42 Argentina, Santiago del Estero, Va San Martin 7 0.930 1.442 1.383 1.382 1.238 1.046 0.732 1.077 0.813 0.684 1.311 0.924 0.987 1.363 0.932 1.305 1.594 1.167 1.955 1.879 0.763 1.262 0.843 0.957 0.530 1.386 1.447 5.66

S. interrupta 42 Argentina, Santiago del Estero, Va San Martin 8 0.560 1.187 1.131 1.132 1.047 0.906 0.560 0.969 0.741 0.635 1.182 0.823 0.930 1.213 0.833 1.184 1.454 1.006 1.840 1.564 0.589 0.958 0.683 0.876 0.427 1.066 1.178 4.69

S. interrupta 42 Argentina, Santiago del Estero, Va San Martin 9 0.710 1.329 1.267 1.258 1.147 1.044 0.562 1.044 0.776 0.668 1.256 0.830 0.997 1.276 0.863 1.236 1.340 1.096 1.812 1.735 0.647 1.149 0.738 0.944 0.454 1.143 1.300 5.15

S. interrupta 42 Argentina, Santiago del Estero, Va San Martin 10 0.640 1.309 1.242 1.242 1.113 0.961 0.538 1.024 0.741 0.639 1.220 0.843 0.994 1.263 0.846 1.232 1.461 1.069 1.808 1.671 0.543 1.087 0.724 0.865 0.472 1.040 1.288 4.88

S. interrupta 42 Argentina, Santiago del Estero, Va San Martin 11 0.690 1.299 1.243 1.234 1.109 0.948 0.575 0.975 0.727 0.627 1.197 0.803 0.973 1.236 0.863 1.145 1.473 1.081 1.746 1.656 0.638 1.085 0.703 0.976 0.474 1.249 1.262 5.18

S. interrupta 42 Argentina, Santiago del Estero, Va San Martin 12 0.390 1.123 1.020 0.994 0.924 0.804 0.433 0.884 0.704 0.536 0.937 0.749 0.862 1.020 0.727 1.107 1.124 0.906 1.551 1.443 0.485 0.904 0.645 0.741 0.384 1.051 1.091 4.36

S. interrupta 42 Argentina, Santiago del Estero, Va San Martin 13 0.380 1.172 1.084 1.049 0.941 0.881 0.552 0.938 0.683 0.639 1.097 0.859 0.863 1.130 0.806 1.191 1.362 0.982 1.710 1.464 0.424 0.914 0.606 0.841 0.404 1.105 1.140 4.58

S. interrupta 42 Argentina, Santiago del Estero, Va San Martin 14 0.340 1.119 1.021 0.966 0.938 0.881 0.461 0.901 0.674 0.601 0.934 0.720 0.933 1.069 0.734 1.103 1.271 0.946 1.556 1.437 0.460 0.887 0.634 0.737 0.381 0.878 1.015 4.17

S. interrupta 42 Argentina, Santiago del Estero, Va San Martin 15 0.220 0.941 0.847 0.784 0.776 0.750 0.429 0.783 0.604 0.573 0.942 0.655 0.775 0.827 0.643 0.960 1.121 0.800 1.415 1.268 0.380 0.794 0.549 0.689 0.326 0.831 0.878 3.73

S. interrupta 42 Argentina, Santiago del Estero, Va San Martin 16 0.290 0.998 0.911 0.855 0.818 0.748 0.479 0.842 0.606 0.578 0.971 0.702 0.787 0.840 0.676 1.043 1.183 0.865 1.457 1.329 0.452 0.846 0.554 0.734 0.349 0.916 1.041 3.98

S. interrupta 42 Argentina, Santiago del Estero, Va San Martin 17 0.280 0.862 0.970 0.807 0.792 0.735 0.377 0.789 0.590 0.545 0.934 0.614 0.789 0.934 0.646 0.926 1.015 0.797 1.387 1.313 0.426 0.798 0.543 0.701 0.333 0.807 0.895 3.68

S. interrupta 42 Argentina, Santiago del Estero, Va San Martin 18 0.240 0.953 0.836 0.780 0.774 0.709 0.458 0.811 0.600 0.555 0.920 0.679 0.780 0.904 0.614 0.967 1.157 0.823 1.383 1.248 0.414 0.782 0.543 0.693 0.320 0.818 0.919 3.71

S. interrupta 42 Argentina, Santiago del Estero, Va San Martin 19 0.160 0.886 0.774 0.723 0.722 0.641 0.398 0.746 0.558 0.523 0.876 0.597 0.660 1.036 0.740 1.278 1.048 0.716 1.294 1.170 0.354 0.736 0.488 0.654 0.306 0.707 0.802 3.42

S. interrupta 42 Argentina, Santiago del Estero, Va San Martin 20 0.170 0.837 0.711 0.670 0.652 0.565 0.388 0.705 0.538 0.505 0.774 0.551 0.709 0.772 0.543 0.677 0.951 0.708 1.194 1.125 0.307 0.695 0.468 0.616 0.267 0.719 0.727 3.30

S. interrupta 42 Argentina, Santiago del Estero, Va San Martin 21 0.190 0.906 0.770 0.720 0.709 0.622 0.438 0.736 0.561 0.520 0.834 0.584 0.699 0.843 0.602 0.869 1.027 0.741 1.306 1.168 0.373 0.735 0.461 0.650 0.313 0.792 0.772 3.52

S. interrupta 42 Argentina, Santiago del Estero, Va San Martin 22 0.160 0.858 0.733 0.711 0.686 0.596 0.395 0.732 0.529 0.517 0.813 0.621 0.679 0.823 0.569 0.830 1.002 0.723 1.239 1.137 0.337 0.732 0.443 0.653 0.291 0.752 0.754 3.40

S. interrupta 42 Argentina, Santiago del Estero, Va San Martin 23 0.170 0.880 0.753 0.682 0.694 0.601 0.467 0.724 0.535 0.491 0.827 0.609 0.680 0.843 0.553 0.885 1.033 0.739 1.268 1.137 0.426 0.728 0.488 0.640 0.302 0.713 0.762 3.37

S. interrupta 42 Argentina, Santiago del Estero, Va San Martin 24 0.160 0.809 0.703 0.653 0.654 0.569 0.399 0.707 0.493 0.509 0.782 0.518 0.684 0.767 0.541 0.809 0.939 0.685 1.257 1.093 0.363 0.691 0.466 0.628 0.281 0.701 0.741 3.23

S. interrupta 42 Argentina, Santiago del Estero, Va San Martin 25 0.160 0.806 0.699 0.688 0.641 0.595 0.332 0.690 0.489 0.488 0.750 0.553 0.604 0.635 0.526 0.761 0.910 0.657 1.149 1.077 0.336 0.655 0.456 0.598 0.266 0.665 0.744 3.15

S. interrupta 42 Argentina, Santiago del Estero, Va San Martin 26 0.130 0.813 0.689 0.648 0.644 0.555 0.391 0.665 0.502 0.480 0.748 0.591 0.609 0.750 0.539 0.802 0.929 0.674 1.161 1.082 0.327 0.657 0.446 0.604 0.272 0.653 0.754 3.15

S. interrupta 42 Argentina, Santiago del Estero, Va San Martin 27 0.140 0.798 0.738 0.674 0.656 0.602 0.415 0.723 0.501 0.515 0.777 0.559 0.684 0.793 0.557 0.845 0.983 0.703 1.231 1.072 0.351 0.716 0.439 0.655 0.278 0.747 0.699 3.27

S. interrupta 42 Argentina, Santiago del Estero, Va San Martin 28 0.120 0.781 0.685 0.655 0.636 0.532 0.332 0.691 0.479 0.492 0.774 0.467 0.650 0.645 0.508 0.791 0.914 0.667 1.156 1.058 0.341 0.681 0.433 0.608 0.281 0.616 0.673 3.06

S. macdonaghi 43 Brazil, Mato Grosso del Sul, Rt. 141 at Itaquirai 1 0.220 0.806 0.732 0.675 0.668 0.576 0.381 0.704 0.544 0.508 0.819 0.617 0.630 0.805 0.516 0.860 0.995 0.704 1.216 1.054 0.402 0.681 0.453 0.632 0.287 0.638 0.756 3.13

S. macdonaghi 43 Brazil, Mato Grosso del Sul, Rt. 141 at Itaquirai 2 0.200 0.859 0.761 0.726 0.693 0.570 0.315 0.710 0.564 0.483 0.760 0.611 0.740 0.817 0.586 0.871 0.860 0.721 1.327 1.115 0.362 0.702 0.446 0.621 0.291 0.699 0.813 3.29

S. macdonaghi 43 Brazil, Mato Grosso del Sul, Rt. 141 at Itaquirai 3 0.280 0.816 0.778 0.717 0.697 0.603 0.351 0.802 0.626 0.462 0.869 0.720 0.599 0.765 0.582 0.903 1.091 0.824 1.261 1.153 0.385 0.690 0.511 0.632 0.304 0.723 0.732 3.32

S. macdonaghi 43 Brazil, Mato Grosso del Sul, Rt. 141 at Itaquirai 4 0.300 0.926 0.846 0.768 0.743 0.645 0.439 0.767 0.594 0.559 0.935 0.706 0.748 0.928 0.616 0.971 1.111 0.809 1.388 1.240 0.408 0.792 0.512 0.698 0.330 0.769 0.856 3.63

S. macdonaghi 43 Brazil, Mato Grosso del Sul, Rt. 141 at Itaquirai 5 0.370 0.959 0.871 0.804 0.779 0.686 0.463 0.772 0.579 0.578 0.948 0.720 0.765 0.911 0.689 0.954 1.123 0.816 1.447 1.293 0.419 0.809 0.532 0.736 0.354 0.804 0.926 3.79

S. macdonaghi 43 Brazil, Mato Grosso del Sul, Rt. 141 at Itaquirai 6 0.400 0.966 0.892 0.836 0.838 0.744 0.469 0.794 0.622 0.583 0.952 0.693 0.763 0.985 0.652 1.024 1.202 0.833 1.476 1.304 0.425 0.794 0.597 0.711 0.358 0.677 0.883 3.66

S. macdonaghi 43 Brazil, Mato Grosso del Sul, Rt. 141 at Itaquirai 7 0.620 1.223 1.177 1.177 1.031 0.888 0.614 0.966 0.728 0.656 1.192 0.859 0.951 1.206 0.856 1.216 1.447 1.058 1.828 1.638 0.555 0.998 0.727 0.857 0.442 1.081 1.221 4.80

S. macdonaghi 43 Brazil, Mato Grosso del Sul, Rt. 141 at Itaquirai 8 0.450 1.124 1.026 1.011 0.945 0.753 0.537 0.890 0.681 0.616 1.085 0.782 0.881 1.112 0.770 1.101 1.333 0.939 1.652 1.511 0.512 0.913 0.657 0.714 0.393 0.960 1.071 4.31

S. macdonaghi 43 Brazil, Mato Grosso del Sul, Rt. 141 at Itaquirai 9 0.360 0.944 0.870 0.833 0.787 0.691 0.426 0.774 0.586 0.574 0.925 0.641 0.754 0.928 0.627 0.949 1.117 0.852 1.410 1.273 0.434 0.788 0.566 0.692 0.343 0.801 0.941 3.71

S. macdonaghi 43 Brazil, Mato Grosso del Sul, Rt. 141 at Itaquirai 10 0.260 0.784 0.742 0.698 0.683 0.585 0.393 0.689 0.521 0.492 0.812 0.577 0.665 0.808 0.563 0.814 0.982 0.687 1.237 1.096 0.433 0.688 0.459 0.706 0.307 0.747 0.776 3.33

S. macdonaghi 43 Brazil, Mato Grosso del Sul, Rt. 141 at Itaquirai 11 0.250 0.779 0.710 0.674 0.659 0.545 0.371 0.678 0.512 0.529 0.802 0.595 0.687 0.796 0.558 0.859 0.987 0.700 1.309 1.106 0.353 0.696 0.469 0.621 0.311 0.718 0.695 3.22

S. macdonaghi 43 Brazil, Mato Grosso del Sul, Rt. 141 at Itaquirai 12 1.180 1.419 1.483 1.514 1.315 1.104 0.683 1.046 0.832 0.715 1.371 0.964 1.123 1.221 0.966 1.353 1.482 1.144 2.089 1.981 0.793 1.332 0.941 1.136 0.641 1.302 1.902 5.84

S. macdonaghi 43 Brazil, Mato Grosso del Sul, Rt. 141 at Itaquirai 13 0.220 0.792 0.699 0.657 0.663 0.535 0.357 0.684 0.530 0.490 0.790 0.545 0.672 0.758 0.571 0.807 0.965 0.701 1.226 1.075 0.383 0.679 0.445 0.620 0.284 0.649 0.774 3.14

S. macdonaghi 43 Brazil, Mato Grosso del Sul, Rt. 141 at Itaquirai 14 0.180 0.864 0.777 0.724 0.719 0.596 0.415 0.731 0.553 0.530 0.850 0.581 0.716 0.732 0.590 0.892 0.932 0.747 1.327 1.162 0.407 0.745 0.466 0.667 0.321 0.760 0.842 3.45

S. macdonaghi 43 Brazil, Mato Grosso del Sul, Rt. 141 at Itaquirai 15 0.120 0.799 0.691 0.660 0.637 0.562 0.378 0.654 0.492 0.494 0.773 0.538 0.646 0.760 0.542 0.785 0.843 0.639 1.179 1.048 0.381 0.645 0.439 0.585 0.274 0.654 0.683 3.09

S. macdonaghi 43 Brazil, Mato Grosso del Sul, Rt. 141 at Itaquirai 16 0.190 0.825 0.691 0.655 0.642 0.562 0.370 0.667 0.496 0.474 0.966 0.622 0.786 0.972 0.684 0.945 1.153 0.819 1.455 1.052 0.349 0.640 0.462 0.544 0.248 0.590 0.729 3.01

S. macdonaghi 43 Brazil, Mato Grosso del Sul, Rt. 141 at Itaquirai 17 0.320 0.974 0.872 0.825 0.794 0.696 0.455 0.805 0.612 0.572 0.879 0.588 0.761 0.865 0.589 0.908 1.062 0.745 1.377 1.345 0.524 0.807 0.633 0.697 0.347 0.798 0.934 3.81

S. macdonaghi 43 Brazil, Mato Grosso del Sul, Rt. 141 at Itaquirai 18 0.230 0.872 0.770 0.744 0.722 0.580 0.435 0.711 0.555 0.525 0.858 0.579 0.766 0.860 0.594 0.917 1.058 0.735 1.377 1.184 0.407 0.746 0.489 0.635 0.326 0.734 0.781 3.42

S. macdonaghi 43 Brazil, Mato Grosso del Sul, Rt. 141 at Itaquirai 19 0.250 0.826 0.732 0.691 0.683 0.541 0.401 0.711 0.481 0.554 0.808 0.548 0.726 0.801 0.557 0.838 0.995 0.710 1.235 1.107 0.401 0.703 0.463 0.641 0.296 0.665 0.810 3.24

S. macdonaghi 43 Brazil, Mato Grosso del Sul, Rt. 141 at Itaquirai 20 0.400 1.160 1.082 1.036 0.975 0.784 0.590 0.913 0.647 0.620 1.100 0.814 0.902 1.094 0.787 1.113 1.312 0.967 1.652 1.468 0.517 0.943 0.644 0.785 0.400 0.905 1.184 4.32

S. macdonaghi 43 Brazil, Mato Grosso del Sul, Rt. 141 at Itaquirai 21 0.250 0.960 0.815 0.800 0.768 0.665 0.434 0.790 0.552 0.576 0.949 0.627 0.761 0.803 0.637 0.924 1.124 0.770 1.403 1.227 0.394 0.755 0.494 0.701 0.308 0.782 0.865 3.67

S. macdonaghi 43 Brazil, Mato Grosso del Sul, Rt. 141 at Itaquirai 22 0.610 1.124 1.103 1.134 0.99 0.768 0.559 0.925 0.725 0.591 1.160 0.766 0.932 1.144 0.838 1.189 1.415 0.962 1.754 1.584 0.568 0.976 0.677 0.812 0.423 0.920 1.164 4.44

S. macdonaghi 43 Brazil, Mato Grosso del Sul, Rt. 141 at Itaquirai 23 0.170 0.733 0.635 0.604 0.586 0.512 0.375 0.628 0.432 0.499 0.741 0.462 0.618 0.719 0.488 0.773 0.865 0.599 1.127 1.010 0.337 0.647 0.402 0.580 0.271 0.596 0.623 2.92

S. macdonaghi 43 Brazil, Mato Grosso del Sul, Rt. 141 at Itaquirai 24 0.220 0.801 0.716 0.676 0.675 0.571 0.418 0.683 0.476 0.544 0.820 0.553 0.679 0.693 0.557 0.824 1.001 0.686 1.210 1.123 0.388 0.724 0.487 0.614 0.310 0.668 0.726 3.21

S. macdonaghi 43 Brazil, Mato Grosso del Sul, Rt. 141 at Itaquirai 25 0.340 0.830 0.732 0.686 0.675 0.552 0.364 0.694 0.480 0.534 0.828 0.592 0.681 0.761 0.557 0.870 1.015 0.712 1.282 1.169 0.419 0.742 0.477 0.676 0.298 0.751 0.814 3.43

S. macdonaghi 43 Brazil, Mato Grosso del Sul, Rt. 141 at Itaquirai 26 0.720 1.258 1.217 1.201 1.107 0.969 0.587 0.991 0.746 0.672 1.096 0.862 1.018 1.246 0.848 1.266 1.524 1.075 1.903 1.746 0.675 1.050 0.795 0.865 0.480 1.031 1.433 4.90

S. macdonaghi 43 Brazil, Mato Grosso del Sul, Rt. 141 at Itaquirai 27 0.450 1.145 1.052 1.005 0.97 0.799 0.535 0.903 0.665 0.625 1.080 0.740 0.912 1.076 0.743 1.113 1.305 0.947 1.625 1.477 0.542 0.884 0.652 0.811 0.411 0.947 1.078 4.38

S. macdonaghi 43 Brazil, Mato Grosso del Sul, Rt. 141 at Itaquirai 28 0.320 0.918 0.815 0.779 0.776 0.656 0.436 0.779 0.600 0.555 0.943 0.668 0.776 0.947 0.628 0.947 1.156 0.824 1.472 1.268 0.410 0.784 0.564 0.662 0.337 0.729 0.947 3.58

S. macdonaghi 43 Brazil, Mato Grosso del Sul, Rt. 141 at Itaquirai 29 0.220 0.954 0.881 0.840 0.818 0.706 0.482 0.792 0.644 0.565 0.969 0.689 0.811 0.857 0.680 0.977 1.174 0.827 1.410 1.277 0.432 0.793 0.556 0.715 0.350 0.758 1.002 3.70

S. macdonaghi 43 Brazil, Mato Grosso del Sul, Rt. 141 at Itaquirai 30 0.180 0.785 0.688 0.647 0.64 0.518 0.374 0.693 0.466 0.518 0.781 0.585 0.659 0.676 0.549 0.819 0.971 0.701 1.183 1.056 0.339 0.672 0.442 0.597 0.285 0.685 0.678 3.12

S. macdonaghi 43 Brazil, Mato Grosso del Sul, Rt. 141 at Itaquirai 31 0.270 0.959 0.887 0.830 0.82 0.702 0.466 0.790 0.623 0.547 0.928 0.680 0.807 0.973 0.663 0.987 1.166 0.822 1.453 1.289 0.433 0.814 0.543 0.725 0.339 0.837 0.894 3.81

S. macdonaghi 43 Brazil, Mato Grosso del Sul, Rt. 141 at Itaquirai 32 0.400 1.051 0.985 0.938 0.907 0.744 0.522 0.865 0.636 0.613 1.054 0.778 0.887 1.053 0.761 1.079 1.309 0.932 1.604 1.428 0.455 0.860 0.632 0.778 0.402 0.998 0.991 4.25

S. macdonaghi 43 Brazil, Mato Grosso del Sul, Rt. 141 at Itaquirai 33 0.160 0.763 0.681 0.633 0.627 0.561 0.376 0.640 0.460 0.484 0.765 0.513 0.625 0.745 0.499 0.785 0.892 0.656 1.133 1.051 0.349 0.664 0.405 0.577 0.270 0.620 0.672 3.01

S. macdonaghi 43 Brazil, Mato Grosso del Sul, Rt. 141 at Itaquirai 34 0.190 0.782 0.703 0.652 0.64 0.563 0.352 0.676 0.473 0.491 0.620 0.538 0.663 0.789 0.525 0.779 0.923 0.644 1.179 1.102 0.392 0.673 0.478 0.601 0.277 0.720 0.736 3.21

S. macdonaghi 43 Brazil, Mato Grosso del Sul, Rt. 141 at Itaquirai 35 0.160 0.676 0.670 0.619 0.611 0.521 0.362 0.622 0.494 0.468 0.756 0.537 0.622 0.729 0.503 0.788 0.900 0.647 1.169 1.031 0.337 0.664 0.403 0.619 0.271 0.669 0.630 2.99

S. macdonaghi 43 Brazil, Mato Grosso del Sul, Rt. 141 at Itaquirai 36 0.150 0.800 0.715 0.674 0.673 0.601 0.413 0.689 0.506 0.519 0.807 0.576 0.675 0.804 0.558 0.834 0.971 0.717 1.267 1.120 0.348 0.723 0.445 0.635 0.289 0.605 0.811 3.16

S. macdonaghi 43 Brazil, Mato Grosso del Sul, Rt. 141 at Itaquirai 37 0.180 0.832 0.754 0.696 0.692 0.599 0.383 0.714 0.528 0.513 0.813 0.587 0.700 0.839 0.538 0.860 1.016 0.700 1.299 1.178 0.404 0.716 0.523 0.590 0.290 0.672 0.746 3.27

S. macdonaghi 43 Brazil, Mato Grosso del Sul, Rt. 141 at Itaquirai 38 0.330 0.928 0.872 0.844 0.81 0.673 0.453 0.830 0.581 0.578 0.946 0.692 0.797 0.990 0.686 0.977 1.180 0.857 1.413 1.216 0.453 0.760 0.518 0.638 0.301 0.801 0.889 3.58

S. macdonaghi 43 Brazil, Mato Grosso del Sul, Rt. 141 at Itaquirai 39 0.250 0.865 0.787 0.742 0.731 0.667 0.400 0.753 0.557 0.549 0.858 0.585 0.770 0.761 0.608 0.910 1.062 0.743 1.375 1.230 0.443 0.753 0.518 0.632 0.320 0.635 0.763 3.36

S. macdonaghi 43 Brazil, Mato Grosso del Sul, Rt. 141 at Itaquirai 40 0.280 0.964 0.907 0.851 0.835 0.718 0.481 0.820 0.620 0.552 0.988 0.710 0.783 0.992 0.708 0.993 1.210 0.882 1.502 1.319 0.444 0.825 0.567 0.676 0.347 0.797 1.026 3.76

S. megergates 44 Brazil, Parana, Rt. 116, Rio Negro 1 2.010 1.637 1.674 1.689 1.498 1.316 0.936 1.262 0.965 0.796 1.399 1.095 1.285 1.431 1.146 1.632 1.727 1.364 2.377 2.204 0.951 1.498 0.971 1.204 0.650 1.518 2.156 6.56

S. megergates 44 Brazil, Parana, Rt. 116, Rio Negro 2 1.970 1.621 1.757 1.753 1.522 1.297 0.895 1.308 1.040 0.804 1.368 1.174 1.334 1.523 1.161 1.699 1.765 1.404 2.433 2.436 1.039 1.612 1.104 1.270 0.706 1.565 2.170 6.89

S. megergates 44 Brazil, Parana, Rt. 116, Rio Negro 3 2.410 1.582 1.670 1.659 1.503 1.239 0.835 1.239 0.926 0.828 1.334 1.180 1.212 1.406 1.124 1.678 1.639 1.411 2.462 2.275 0.922 1.449 0.993 1.172 0.656 1.609 2.090 6.64

S. megergates 44 Brazil, Parana, Rt. 116, Rio Negro 4 0.860 1.348 1.286 1.264 1.19 1.023 0.691 1.080 0.844 0.693 1.138 0.962 1.041 1.158 0.952 1.402 1.466 1.172 2.096 1.783 0.649 1.104 0.738 0.956 0.480 1.093 1.363 5.18

S. megergates 44 Brazil, Parana, Rt. 116, Rio Negro 5 0.650 1.253 1.188 1.143 1.115 0.997 0.684 1.029 0.760 0.663 1.057 0.896 1.038 1.083 0.859 1.204 1.292 1.051 1.710 1.741 0.657 1.080 0.752 0.868 0.445 1.162 1.223 5.02

S. megergates 44 Brazil, Parana, Rt. 116, Rio Negro 6 0.790 1.252 1.171 1.086 1.073 0.907 0.629 1.034 0.767 0.728 1.136 0.866 1.033 1.165 0.907 1.309 1.303 1.143 1.931 1.627 0.561 1.013 0.682 0.856 0.440 0.861 1.223 4.60

S. megergates 44 Brazil, Parana, Rt. 116, Rio Negro 7 0.820 1.174 1.111 1.031 1.037 0.868 0.570 0.971 0.747 0.715 1.073 0.833 1.111 1.044 0.861 1.324 1.291 1.130 1.873 1.601 0.525 0.988 0.675 0.802 0.428 0.935 1.166 4.51

S. megergates 44 Brazil, Parana, Rt. 116, Rio Negro 8 0.520 1.142 1.043 0.992 1.001 0.858 0.622 0.932 0.698 0.632 0.927 0.787 0.894 0.944 0.748 1.078 1.141 0.924 1.573 1.523 0.542 0.960 0.631 0.834 0.396 1.145 1.152 4.64

S. megergates 44 Brazil, Parana, Rt. 116, Rio Negro 9 0.300 1.031 0.928 0.865 0.888 0.731 0.501 0.881 0.627 0.587 0.855 0.710 0.826 0.878 0.673 1.012 1.066 0.878 1.415 1.378 0.485 0.872 0.598 0.723 0.355 0.929 0.955 4.06

S. megergates 44 Brazil, Parana, Rt. 116, Rio Negro 10 0.260 0.922 0.845 0.777 0.809 0.689 0.487 0.861 0.664 0.590 0.842 0.712 0.854 0.866 0.690 1.048 1.088 0.913 1.530 1.318 0.461 0.821 0.533 0.726 0.347 0.722 0.886 3.69

S. megergates 44 Brazil, Parana, Rt. 116, Rio Negro 11 0.260 0.895 0.774 0.715 0.74 0.602 0.473 0.788 0.560 0.542 0.730 0.647 0.703 0.753 0.616 0.878 0.920 0.763 1.269 1.229 0.392 0.767 0.519 0.633 0.299 0.742 0.923 3.50

S. megergates 44 Brazil, Parana, Rt. 116, Rio Negro 12 0.290 0.881 0.782 0.710 0.763 0.669 0.503 0.771 0.549 0.539 0.731 0.645 0.732 0.758 0.587 0.915 0.923 0.775 1.281 1.239 0.405 0.762 0.495 0.593 0.294 0.824 0.777 3.54

S. megergates 44 Brazil, Parana, Rt. 116, Rio Negro 13 0.260 0.965 0.877 0.778 0.834 0.769 0.535 0.845 0.616 0.608 0.839 0.668 0.814 0.802 0.679 0.944 0.986 0.821 1.419 1.270 0.446 0.803 0.543 0.684 0.341 0.783 0.896 3.70

S. megergates 44 Brazil, Parana, Rt. 116, Rio Negro 14 0.250 0.882 0.762 0.710 0.754 0.644 0.425 0.770 0.529 0.526 0.750 0.629 0.703 0.746 0.587 0.870 0.923 0.705 1.268 1.222 0.409 0.754 0.500 0.644 0.306 0.746 0.825 3.49

S. megergates 44 Brazil, Parana, Rt. 116, Rio Negro 15 0.230 0.912 0.834 0.762 0.812 0.736 0.518 0.777 0.553 0.517 0.785 0.636 0.759 0.747 0.611 0.936 0.937 0.768 1.333 1.273 0.446 0.782 0.536 0.690 0.321 0.797 0.862 3.67

S. megergates 44 Brazil, Parana, Rt. 116, Rio Negro 16 0.200 0.907 0.793 0.731 0.774 0.669 0.457 0.802 0.595 0.524 0.821 0.645 0.759 0.768 0.637 0.910 0.944 0.760 1.326 1.272 0.441 0.780 0.538 0.673 0.314 0.772 0.850 3.62

S. megergates 44 Brazil, Parana, Rt. 116, Rio Negro 17 0.300 0.892 0.966 0.846 0.871 0.741 0.518 0.868 0.597 0.587 0.865 0.686 0.820 0.806 0.642 0.978 0.993 0.812 1.403 1.361 0.501 0.850 0.549 0.741 0.345 0.883 0.954 3.88

S. megergates 44 Brazil, Parana, Rt. 116, Rio Negro 18 0.310 0.954 0.860 0.788 0.826 0.705 0.474 0.799 0.560 0.596 0.783 0.678 0.779 0.819 0.635 0.941 0.984 0.779 1.351 1.301 0.487 0.827 0.562 0.731 0.325 0.845 0.886 3.83

S. megergates 44 Brazil, Parana, Rt. 116, Rio Negro 19 0.170 0.815 0.689 0.627 0.66 0.596 0.395 0.744 0.503 0.478 0.689 0.583 0.660 0.643 0.538 0.825 0.855 0.692 1.213 1.102 0.384 0.683 0.431 0.631 0.272 0.693 0.709 3.24

S. megergates 44 Brazil, Parana, Rt. 116, Rio Negro 20 0.270 0.881 0.762 0.705 0.732 0.606 0.452 0.773 0.524 0.549 0.728 0.608 0.733 0.715 0.588 0.886 0.891 0.740 1.223 1.191 0.424 0.769 0.482 0.675 0.311 0.764 0.769 3.51

S. megergates 44 Brazil, Parana, Rt. 116, Rio Negro 21 0.180 0.806 0.696 0.640 0.688 0.601 0.425 0.686 0.508 0.512 0.698 0.551 0.679 0.705 0.528 0.857 0.807 0.678 1.174 1.115 0.379 0.706 0.476 0.625 0.273 0.780 0.690 3.33

S. megergates 44 Brazil, Parana, Rt. 116, Rio Negro 22 0.250 0.855 0.736 0.682 0.715 0.626 0.443 0.756 0.542 0.514 0.774 0.559 0.746 0.745 0.582 0.880 0.897 0.731 1.240 1.150 0.425 0.721 0.476 0.643 0.290 0.768 0.774 3.42

S. megergates 44 Brazil, Parana, Rt. 116, Rio Negro 23 0.310 0.924 0.815 0.747 0.792 0.701 0.489 0.802 0.547 0.591 0.789 0.608 0.770 0.739 0.624 0.915 0.947 0.807 1.280 1.258 0.445 0.788 0.517 0.670 0.323 0.771 0.861 3.62

S. megergates 44 Brazil, Parana, Rt. 116, Rio Negro 24 0.260 0.882 0.764 0.704 0.743 0.677 0.482 0.750 0.542 0.510 0.722 0.639 0.722 0.779 0.583 0.915 0.944 0.757 1.292 1.196 0.447 0.772 0.508 0.663 0.308 0.793 0.790 3.53

S. megergates 44 Brazil, Parana, Rt. 116, Rio Negro 25 0.180 0.850 0.732 0.695 0.715 0.658 0.426 0.723 0.498 0.529 0.837 0.589 0.716 0.725 0.542 0.916 0.852 0.708 1.267 1.177 0.364 0.716 0.493 0.651 0.288 0.674 0.746 3.35

S. megergates 44 Brazil, Parana, Rt. 116, Rio Negro 26 0.220 0.850 0.720 0.668 0.694 0.622 0.431 0.699 0.499 0.495 0.790 0.583 0.686 0.707 0.563 0.839 0.877 0.675 1.204 1.146 0.375 0.714 0.458 0.644 0.280 0.660 0.755 3.30

S. megergates 44 Brazil, Parana, Rt. 116, Rio Negro 27 0.270 0.767 0.741 0.668 0.698 0.653 0.466 0.794 0.550 0.577 0.753 0.655 0.708 0.764 0.639 0.993 0.973 0.815 1.344 1.211 0.425 0.733 0.529 0.623 0.305 0.753 0.787 3.35

S. megergates 44 Brazil, Parana, Rt. 116, Rio Negro 28 0.190 0.835 0.751 0.705 0.729 0.617 0.440 0.762 0.519 0.540 0.825 0.612 0.661 0.727 0.591 0.831 0.883 0.728 1.207 1.164 0.379 0.728 0.493 0.627 0.302 0.718 0.789 3.34

S. megergates 44 Brazil, Parana, Rt. 116, Rio Negro 29 0.220 0.944 0.821 0.734 0.784 0.700 0.485 0.803 0.600 0.624 0.829 0.691 0.760 0.865 0.739 0.993 1.043 0.853 1.501 1.251 0.431 0.795 0.545 0.665 0.312 0.780 0.875 3.64

S. megergates 44 Brazil, Parana, Rt. 116, Rio Negro 30 0.230 0.886 0.736 0.700 0.731 0.632 0.430 0.754 0.550 0.500 0.745 0.576 0.704 0.731 0.582 0.849 0.878 0.722 1.223 1.178 0.397 0.739 0.472 0.647 0.296 0.793 0.772 3.50

S. megergates 44 Brazil, Parana, Rt. 116, Rio Negro 31 0.200 0.862 0.752 0.663 0.73 0.591 0.415 0.757 0.514 0.514 0.793 0.596 0.719 0.735 0.588 0.829 0.892 0.737 1.224 1.170 0.424 0.749 0.461 0.653 0.313 0.725 0.757 3.41

S. megergates 44 Brazil, Parana, Rt. 116, Rio Negro 32 0.240 0.830 0.726 0.674 0.681 0.627 0.411 0.739 0.573 0.498 0.739 0.588 0.696 0.744 0.565 0.876 0.899 0.707 1.306 1.162 0.403 0.729 0.503 0.608 0.299 0.720 0.752 3.32

S. megergates 44 Brazil, Parana, Rt. 116, Rio Negro 33 0.270 0.883 0.811 0.758 0.778 0.706 0.507 0.744 0.556 0.532 0.782 0.647 0.704 0.767 0.594 0.901 0.935 0.783 1.259 1.259 0.434 0.767 0.534 0.641 0.332 0.859 0.820 3.64

S. megergates 44 Brazil, Parana, Rt. 116, Rio Negro 34 0.200 0.824 0.746 0.674 0.731 0.606 0.466 0.766 0.550 0.511 0.771 0.633 0.719 0.721 0.622 0.851 0.920 0.715 1.240 1.165 0.404 0.705 0.514 0.596 0.286 0.720 0.691 3.30

S. megergates 44 Brazil, Parana, Rt. 116, Rio Negro 35 0.160 0.814 0.690 0.633 0.663 0.607 0.422 0.725 0.515 0.480 0.729 0.563 0.717 0.658 0.550 0.860 0.829 0.693 1.176 1.107 0.379 0.674 0.453 0.594 0.266 0.731 0.715 3.25

S. megergates 44 Brazil, Parana, Rt. 116, Rio Negro 36 0.200 0.706 0.665 0.600 0.631 0.567 0.392 0.664 0.480 0.470 0.673 0.529 0.627 0.607 0.493 0.780 0.772 0.632 1.131 1.049 0.385 0.668 0.410 0.608 0.266 0.648 0.689 3.01

S. quinquecuspis 45 Argentina, Santa Fe Roldan 1 0.380 0.944 0.855 0.769 0.772 0.698 0.455 0.767 0.593 0.562 0.777 0.582 0.740 0.715 0.602 0.921 0.929 0.761 1.364 1.220 0.414 0.801 0.515 0.756 0.306 0.823 0.935 3.74

S. quinquecuspis 45 Argentina, Santa Fe Roldan 2 0.950 1.317 1.339 1.339 1.193 0.968 0.645 1.055 0.817 0.689 1.087 0.885 1.106 1.075 0.912 1.242 1.394 1.069 1.887 1.886 0.718 1.281 0.883 0.944 0.558 1.345 1.629 5.49

S. quinquecuspis 45 Argentina, Santa Fe Roldan 3 0.260 0.824 0.737 0.704 0.672 0.559 0.357 0.690 0.470 0.570 0.710 0.525 0.701 0.667 0.547 0.837 0.832 0.693 1.224 1.215 0.406 0.726 0.527 0.582 0.300 0.744 0.754 3.37

S. quinquecuspis 45 Argentina, Santa Fe Roldan 4 0.680 1.191 1.160 1.155 1.044 0.895 0.549 0.942 0.645 0.705 1.031 0.798 0.893 1.012 0.786 1.178 1.215 0.960 1.744 1.742 0.603 1.017 0.824 0.776 0.452 1.050 1.221 4.76

S. quinquecuspis 45 Argentina, Santa Fe Roldan 5 0.370 0.973 0.909 0.849 0.828 0.704 0.472 0.786 0.598 0.564 0.775 0.650 0.776 0.806 0.640 0.931 1.000 0.785 1.387 1.322 0.447 0.836 0.558 0.741 0.346 0.834 0.973 3.87

S. quinquecuspis 45 Argentina, Santa Fe Roldan 6 0.220 0.860 0.742 0.710 0.687 0.581 0.375 0.699 0.512 0.540 0.688 0.549 0.676 0.673 0.535 0.816 0.864 0.720 1.204 1.159 0.355 0.717 0.478 0.641 0.272 0.753 0.753 3.41

S. quinquecuspis 45 Argentina, Santa Fe Roldan 7 0.310 0.936 0.855 0.807 0.778 0.635 0.445 0.804 0.582 0.560 0.809 0.645 0.755 0.748 0.616 0.898 0.957 0.768 1.366 1.311 0.461 0.820 0.531 0.726 0.331 0.839 0.928 3.81

S. quinquecuspis 45 Argentina, Santa Fe Roldan 8 0.190 0.743 0.651 0.613 0.605 0.505 0.328 0.635 0.489 0.445 0.638 0.485 0.605 0.604 0.483 0.733 0.762 0.576 1.083 1.024 0.330 0.672 0.422 0.590 0.246 0.614 0.678 2.97

S. quinquecuspis 45 Argentina, Santa Fe Roldan 9 0.350 1.059 0.969 0.931 0.859 0.688 0.450 0.861 0.659 0.570 0.901 0.735 0.810 0.874 0.696 1.001 1.066 0.869 1.517 1.398 0.486 0.905 0.638 0.636 0.376 0.960 0.969 4.05

S. quinquecuspis 45 Argentina, Santa Fe Roldan 10 0.190 0.845 0.747 0.721 0.693 0.603 0.412 0.724 0.556 0.527 0.714 0.568 0.700 0.694 0.549 0.821 0.856 0.684 1.249 1.183 0.371 0.755 0.500 0.672 0.299 0.738 0.807 3.44

S. quinquecuspis 45 Argentina, Santa Fe Roldan 11 1.090 1.367 1.345 1.351 1.195 1.002 0.673 1.025 0.726 0.799 1.165 0.868 1.017 1.126 0.894 1.281 1.336 1.046 1.933 2.013 0.738 1.257 0.888 0.989 0.541 1.297 1.568 5.67

S. quinquecuspis 45 Argentina, Santa Fe Roldan 12 1.020 1.353 1.340 1.388 1.197 1.022 0.627 1.056 0.815 0.665 1.161 0.888 1.038 1.111 0.900 1.289 1.343 1.092 1.933 1.952 0.651 1.242 0.830 1.005 0.536 1.238 1.539 5.55

S. quinquecuspis 45 Argentina, Santa Fe Roldan 13 0.260 0.876 0.812 0.763 0.773 0.635 0.352 0.742 0.565 0.554 0.753 0.566 0.722 0.739 0.576 0.877 0.945 0.722 1.277 1.243 0.398 0.777 0.520 0.652 0.303 0.747 0.822 3.52

S. quinquecuspis 45 Argentina, Santa Fe Roldan 14 1.210 1.442 1.473 1.462 1.286 1.097 0.648 1.073 0.810 0.738 1.161 0.898 1.054 1.217 0.934 1.325 1.389 1.152 1.964 2.026 0.836 1.347 0.897 0.981 0.575 1.367 1.647 5.82

S. quinquecuspis 45 Argentina, Santa Fe Roldan 15 0.310 0.922 0.845 0.797 0.756 0.571 0.366 0.760 0.607 0.556 0.756 0.620 0.741 0.765 0.620 0.955 0.954 0.784 1.437 1.296 0.430 0.818 0.550 0.706 0.307 0.872 0.900 3.80

S. quinquecuspis 45 Argentina, Santa Fe Roldan 16 0.200 0.914 0.812 0.753 0.738 0.629 0.425 0.770 0.576 0.550 0.760 0.576 0.758 0.740 0.627 0.894 0.951 0.776 1.357 1.237 0.398 0.764 0.539 0.704 0.328 0.829 0.798 3.68

S. quinquecuspis 45 Argentina, Santa Fe Roldan 17 0.370 1.006 0.901 0.868 0.846 0.732 0.469 0.826 0.616 0.601 0.812 0.667 0.774 0.811 0.670 0.985 1.004 0.849 1.423 1.322 0.502 0.872 0.578 0.661 0.376 0.879 0.988 3.87

S. quinquecuspis 45 Argentina, Santa Fe Roldan 18 0.830 1.285 1.254 1.248 1.164 0.979 0.595 1.005 0.711 0.723 1.061 0.749 1.045 1.087 0.827 1.232 1.279 1.002 1.829 1.670 0.635 1.125 0.710 0.834 0.475 1.129 1.355 4.92

S. quinquecuspis 45 Argentina, Santa Fe Roldan 19 0.590 1.220 1.161 1.140 1.074 0.925 0.586 0.961 0.692 0.672 0.984 0.770 0.949 0.968 0.780 1.147 1.217 0.977 1.759 1.675 0.609 1.065 0.726 0.870 0.451 1.118 1.204 4.88

S. quinquecuspis 45 Argentina, Santa Fe Roldan 20 0.200 0.873 0.770 0.710 0.711 0.636 0.415 0.726 0.493 0.541 0.712 0.574 0.674 0.684 0.545 0.850 0.862 0.699 1.226 1.230 0.386 0.753 0.544 0.609 0.312 0.802 0.788 3.51

S. quinquecuspis 45 Argentina, Santa Fe Roldan 21 0.750 1.287 1.237 1.227 1.099 0.948 0.575 0.982 0.754 0.650 0.993 0.837 0.959 1.069 0.827 1.194 1.256 1.022 1.775 1.746 0.637 1.151 0.771 0.831 0.463 1.167 1.319 5.03

S. quinquecuspis 45 Argentina, Santa Fe Roldan 22 1.230 1.366 1.404 1.426 1.236 1.088 0.698 1.059 0.814 0.760 1.156 0.880 1.071 1.180 0.925 1.258 1.331 1.078 1.941 2.043 0.845 1.339 0.904 0.926 0.539 1.307 1.628 5.64

S. quinquecuspis 45 Argentina, Santa Fe Roldan 23 1.080 1.425 1.419 1.414 1.23 1.005 0.663 1.058 0.834 0.744 1.103 0.920 1.014 1.169 0.878 1.314 1.327 1.083 1.688 1.945 0.676 1.301 0.831 0.940 0.519 1.258 1.568 5.57

S. quinquecuspis 45 Argentina, Santa Fe Roldan 24 1.020 1.331 1.308 1.298 1.143 0.962 0.681 1.061 0.713 0.795 1.090 0.859 1.059 1.130 0.860 1.274 1.318 1.043 1.873 1.938 0.765 1.260 0.839 0.977 0.536 1.217 1.517 5.46

S. quinquecuspis 45 Argentina, Santa Fe Roldan 25 0.730 1.250 1.172 1.154 1.058 0.932 0.581 0.980 0.776 0.673 1.024 0.852 0.925 1.053 0.864 1.187 1.262 1.031 1.787 1.731 0.614 1.075 0.709 0.917 0.456 1.140 1.250 5.04

S. quinquecuspis 45 Argentina, Santa Fe Roldan 26 0.700 1.217 1.146 1.098 1.055 0.900 0.568 0.974 0.735 0.671 0.983 0.747 0.938 0.972 0.795 1.126 1.193 0.980 1.712 1.579 0.575 1.068 0.662 0.830 0.463 1.149 1.225 4.78

S. quinquecuspis 45 Argentina, Santa Fe Roldan 27 0.590 1.152 1.065 1.006 0.962 0.856 0.544 0.919 0.712 0.610 0.948 0.750 0.874 0.970 0.716 1.075 1.131 0.930 1.599 1.586 0.554 0.959 0.692 0.798 0.386 1.039 1.155 4.58

S. quinquecuspis 45 Argentina, Santa Fe Roldan 28 0.370 0.981 0.930 0.870 0.87 0.764 0.514 0.824 0.594 0.636 0.836 0.683 0.825 0.812 0.647 0.972 1.004 0.837 1.448 1.403 0.516 0.878 0.581 0.780 0.351 0.979 1.000 4.14

S. quinquecuspis 45 Argentina, Santa Fe Roldan 29 0.350 1.028 0.954 0.900 0.861 0.679 0.458 0.818 0.646 0.563 0.840 0.616 0.817 0.892 0.669 0.988 1.086 0.839 1.470 1.382 0.457 0.845 0.577 0.753 0.366 0.925 1.043 4.09

S. quinquecuspis 45 Argentina, Santa Fe Roldan 30 0.240 0.939 0.802 0.774 0.745 0.622 0.454 0.802 0.565 0.567 0.777 0.604 0.741 0.770 0.580 0.913 0.932 0.744 1.291 1.282 0.460 0.805 0.566 0.616 0.316 0.737 0.866 3.57

S. quinquecuspis 45 Argentina, Santa Fe Roldan 31 0.120 0.742 0.661 0.618 0.623 0.516 0.317 0.643 0.468 0.490 0.624 0.495 0.604 0.606 0.485 0.714 0.774 0.616 1.089 1.038 0.333 0.685 0.427 0.574 0.253 0.624 0.667 2.98

S. quinquecuspis 45 Argentina, Santa Fe Roldan 32 0.180 0.780 0.710 0.667 0.649 0.565 0.332 0.672 0.480 0.533 0.672 0.514 0.666 0.624 0.529 0.778 0.843 0.632 1.191 1.149 0.385 0.721 0.481 0.630 0.274 0.721 0.721 3.28

S. quinquecuspis 45 Argentina, Santa Fe Roldan 33 0.200 0.808 0.737 0.694 0.687 0.554 0.345 0.704 0.514 0.534 0.699 0.575 0.678 0.662 0.520 0.805 0.844 0.683 1.201 1.150 0.374 0.743 0.488 0.634 0.290 0.702 0.756 3.29

S. quinquecuspis 45 Argentina, Santa Fe Roldan 34 0.210 0.841 0.836 0.722 0.767 0.696 0.434 0.730 0.541 0.513 0.719 0.559 0.700 0.713 0.551 0.823 0.872 0.685 1.239 1.206 0.395 0.728 0.515 0.608 0.301 0.769 0.817 3.42

S. quinquecuspis 45 Argentina, Santa Fe Roldan 35 0.160 0.850 0.774 0.704 0.719 0.592 0.381 0.729 0.551 0.524 0.715 0.593 0.723 0.706 0.549 0.888 0.880 0.699 1.243 1.164 0.419 0.732 0.527 0.594 0.292 0.683 0.790 3.29

S. quinquecuspis 45 Argentina, Santa Fe Roldan 36 0.200 0.840 0.743 0.694 0.688 0.581 0.408 0.707 0.496 0.534 0.706 0.603 0.656 0.698 0.569 0.830 0.850 0.669 1.254 1.176 0.465 0.735 0.515 0.600 0.285 0.691 0.767 3.31

S. quinquecuspis 45 Argentina, Santa Fe Roldan 37 0.130 0.731 0.651 0.613 0.601 0.505 0.340 0.642 0.449 0.445 0.656 0.466 0.569 0.591 0.445 0.720 0.737 0.585 1.065 1.049 0.330 0.637 0.453 0.565 0.259 0.652 0.756 3.00

S. quinquecuspis 45 Argentina, Santa Fe Roldan 38 0.150 0.800 0.692 0.648 0.652 0.565 0.350 0.676 0.500 0.505 0.699 0.530 0.660 0.690 0.503 0.822 0.836 0.670 1.181 1.158 0.394 0.734 0.473 0.608 0.267 0.728 0.728 3.29

S. quinquecuspis 45 Argentina, Santa Fe Roldan 39 0.160 0.774 0.692 0.643 0.629 0.533 0.305 0.668 0.515 0.488 0.671 0.551 0.649 0.653 0.495 0.769 0.843 0.631 1.187 1.110 0.372 0.691 0.495 0.565 0.274 0.667 0.678 3.12

S. quinquecuspis 45 Argentina, Santa Fe Roldan 40 0.180 0.806 0.732 0.684 0.673 0.560 0.344 0.702 0.522 0.515 0.686 0.544 0.657 0.672 0.532 0.778 0.841 0.651 1.187 1.144 0.387 0.706 0.515 0.598 0.281 0.734 0.740 3.28

S. saevissima 46 Brazil, Sergipe, Rt. 101, Propria 1 0.400 0.869 0.740 0.690 0.703 0.622 0.414 0.772 0.565 0.533 0.776 0.654 0.662 0.754 0.609 0.912 0.977 0.781 1.378 1.134 0.368 0.721 0.478 0.666 0.282 0.733 0.707 3.40

S. saevissima 46 Brazil, Sergipe, Rt. 101, Propria 2 0.320 0.820 0.694 0.654 0.674 0.554 0.314 0.759 0.533 0.524 0.776 0.600 0.731 0.768 0.628 0.865 0.916 0.752 1.332 1.142 0.351 0.707 0.495 0.618 0.280 0.708 0.765 3.29

S. saevissima 46 Brazil, Sergipe, Rt. 101, Propria 3 0.300 0.791 0.703 0.652 0.668 0.552 0.310 0.741 0.515 0.530 0.761 0.555 0.691 0.760 0.576 0.872 0.921 0.751 1.289 1.128 0.330 0.676 0.461 0.603 0.258 0.667 0.765 3.19

S. saevissima 46 Brazil, Sergipe, Rt. 101, Propria 4 1.180 1.101 1.015 0.938 0.954 0.813 0.549 0.977 0.700 0.642 1.009 0.769 0.943 0.988 0.826 1.172 1.212 1.000 1.705 1.459 0.526 0.934 0.618 0.751 0.386 0.959 1.191 4.27

S. saevissima 46 Brazil, Sergipe, Rt. 101, Propria 5 0.240 0.749 0.615 0.582 0.582 0.542 0.312 0.684 0.487 0.504 0.631 0.484 0.647 0.641 0.528 0.753 0.783 0.676 1.119 0.981 0.329 0.605 0.409 0.556 0.239 0.630 0.642 2.92

S. saevissima 46 Brazil, Sergipe, Rt. 101, Propria 6 0.220 0.720 0.843 0.675 0.68 0.553 0.330 0.788 0.554 0.529 0.743 0.632 0.743 0.756 0.590 0.921 0.937 0.753 1.329 1.170 0.408 0.713 0.508 0.599 0.282 0.703 0.749 3.19

S. saevissima 46 Brazil, Sergipe, Rt. 101, Propria 7 0.210 0.828 0.708 0.641 0.669 0.528 0.361 0.738 0.522 0.541 0.732 0.566 0.718 0.724 0.590 0.860 0.888 0.742 1.312 1.133 0.354 0.712 0.434 0.630 0.278 0.669 0.752 3.26

S. saevissima 46 Brazil, Sergipe, Rt. 101, Propria 8 0.420 0.885 0.759 0.703 0.712 0.609 0.454 0.800 0.568 0.591 0.821 0.642 0.753 0.808 0.618 0.953 0.970 0.825 1.361 1.210 0.408 0.730 0.518 0.657 0.282 0.747 0.892 3.50

S. saevissima 46 Brazil, Sergipe, Rt. 101, Propria 9 0.980 1.187 1.083 1.032 1.01 0.827 0.616 1.019 0.733 0.655 1.064 0.858 0.956 1.097 0.817 1.256 1.366 1.043 1.812 1.561 0.536 0.964 0.663 0.791 0.407 1.075 1.177 4.61

S. saevissima 46 Brazil, Sergipe, Rt. 101, Propria 10 0.270 0.780 0.663 0.613 0.624 0.563 0.410 0.728 0.498 0.530 0.702 0.604 0.665 0.670 0.558 0.847 0.872 0.728 1.224 1.068 0.356 0.669 0.434 0.612 0.263 0.676 0.736 3.14

S. saevissima 46 Brazil, Sergipe, Rt. 101, Propria 11 0.210 0.809 0.679 0.623 0.637 0.539 0.394 0.728 0.503 0.516 0.720 0.531 0.644 0.699 0.549 0.812 0.867 0.713 1.208 1.072 0.346 0.661 0.474 0.577 0.268 0.681 0.702 3.14

S. saevissima 46 Brazil, Sergipe, Rt. 101, Propria 12 0.190 0.781 0.663 0.607 0.629 0.513 0.368 0.715 0.521 0.506 0.692 0.564 0.674 0.723 0.521 0.802 0.863 0.687 1.218 1.049 0.355 0.654 0.439 0.618 0.252 0.626 0.715 3.07

S. saevissima 46 Brazil, Sergipe, Rt. 101, Propria 13 0.610 0.987 0.885 0.838 0.826 0.700 0.508 0.902 0.615 0.613 0.911 0.697 0.835 0.962 0.719 1.068 1.114 0.859 1.621 1.331 0.524 0.835 0.569 0.716 0.337 0.722 0.952 3.76

S. saevissima 46 Brazil, Sergipe, Rt. 101, Propria 14 0.290 0.813 0.669 0.616 0.641 0.580 0.395 0.728 0.499 0.500 0.685 0.616 0.684 0.693 0.533 0.857 0.881 0.688 1.257 1.099 0.341 0.649 0.468 0.599 0.269 0.617 0.730 3.13

S. saevissima 46 Brazil, Sergipe, Rt. 101, Propria 15 0.210 0.875 0.752 0.680 0.708 0.563 0.409 0.801 0.554 0.557 0.786 0.632 0.745 0.780 0.603 0.942 0.980 0.774 1.356 1.196 0.423 0.726 0.518 0.647 0.292 0.706 0.814 3.42

S. saevissima 46 Brazil, Sergipe, Rt. 101, Propria 16 1.340 1.296 1.244 1.215 1.141 0.929 0.681 1.123 0.793 0.710 1.153 0.916 1.087 1.240 0.955 1.337 1.451 1.196 1.966 1.798 0.754 1.136 0.789 0.984 0.489 1.247 1.374 5.33

S. saevissima 46 Brazil, Sergipe, Rt. 101, Propria 17 0.180 0.782 0.650 0.599 0.609 0.509 0.382 0.705 0.495 0.522 0.659 0.569 0.625 0.672 0.526 0.786 0.815 0.674 1.172 1.058 0.335 0.655 0.436 0.572 0.262 0.582 0.665 2.99

S. saevissima 46 Brazil, Sergipe, Rt. 101, Propria 18 0.530 1.110 1.041 0.988 0.951 0.806 0.572 0.989 0.748 0.641 1.034 0.809 0.955 1.056 0.844 1.176 1.262 1.027 1.788 1.545 0.563 0.969 0.651 0.793 0.388 0.920 1.143 4.37

S. saevissima 46 Brazil, Sergipe, Rt. 101, Propria 19 0.150 0.777 0.653 0.602 0.63 0.559 0.385 0.709 0.528 0.501 0.721 0.589 0.652 0.703 0.547 0.826 0.893 0.703 1.249 1.093 0.424 0.668 0.486 0.553 0.258 0.608 0.715 3.03

S. saevissima 46 Brazil, Sergipe, Rt. 101, Propria 20 0.920 1.133 1.006 0.962 0.939 0.808 0.532 0.972 0.691 0.651 1.010 0.798 0.934 1.032 0.823 1.135 1.257 1.020 1.702 1.536 0.552 0.915 0.649 0.787 0.392 0.998 1.120 4.45

S. saevissima 46 Brazil, Sergipe, Rt. 101, Propria 21 1.320 1.169 1.068 1.022 1.023 0.785 0.597 1.018 0.736 0.658 1.054 0.847 0.925 1.084 0.836 1.218 1.309 1.040 1.885 1.558 0.594 0.979 0.669 0.813 0.385 1.068 1.187 4.61

S. saevissima 46 Brazil, Sergipe, Rt. 101, Propria 22 0.910 1.144 1.094 1.036 1.011 0.827 0.560 1.024 0.753 0.652 1.064 0.807 0.987 1.067 0.869 1.264 1.302 1.069 1.832 1.589 0.583 0.992 0.682 0.858 0.401 0.989 1.155 4.58

S. saevissima 46 Brazil, Sergipe, Rt. 101, Propria 23 0.560 1.168 1.137 1.091 1.055 0.903 0.512 1.027 0.773 0.659 1.099 0.867 0.957 1.064 0.864 1.249 1.279 1.051 1.828 1.598 0.610 0.994 0.671 0.836 0.396 1.132 1.232 4.73

S. saevissima 46 Brazil, Sergipe, Rt. 101, Propria 24 1.460 1.202 1.094 1.069 1.033 0.874 0.479 1.034 0.764 0.664 1.017 0.832 0.945 1.089 0.875 1.305 1.258 1.060 1.775 1.556 0.521 0.940 0.675 0.819 0.406 1.112 1.291 4.69

S. saevissima 46 Brazil, Sergipe, Rt. 101, Propria 25 0.770 1.064 0.964 0.925 0.906 0.770 0.482 0.953 0.688 0.637 0.958 0.742 0.891 0.964 0.790 1.103 1.201 0.977 1.656 1.422 0.479 0.873 0.620 0.745 0.355 0.871 1.138 4.10

S. saevissima 46 Brazil, Sergipe, Rt. 101, Propria 26 0.900 1.085 1.002 0.940 0.925 0.801 0.474 0.967 0.685 0.632 1.017 0.817 0.888 1.013 0.836 1.164 1.168 0.988 1.690 1.464 0.526 0.893 0.623 0.783 0.374 0.981 1.066 4.31

S. saevissima 46 Brazil, Sergipe, Rt. 101, Propria 27 0.370 1.059 0.938 0.881 0.869 0.758 0.531 0.931 0.669 0.593 0.965 0.691 0.923 0.964 0.789 1.143 1.175 0.950 1.677 1.426 0.497 0.858 0.631 0.756 0.366 0.875 0.977 4.12

S. saevissima 46 Brazil, Sergipe, Rt. 101, Propria 28 1.480 1.165 1.087 1.027 1.005 0.825 0.556 1.012 0.753 0.642 1.053 0.806 0.990 1.098 0.827 1.218 1.297 1.003 1.841 1.563 0.558 0.956 0.703 0.786 0.390 1.078 1.228 4.59

S. saevissima 46 Brazil, Sergipe, Rt. 101, Propria 29 0.350 1.018 0.920 0.879 0.871 0.744 0.514 0.903 0.708 0.614 0.962 0.771 0.869 0.948 0.754 1.019 1.162 0.925 1.614 1.363 0.514 0.821 0.596 0.716 0.342 0.893 0.999 3.99

S. saevissima 46 Brazil, Sergipe, Rt. 101, Propria 30 0.470 1.003 0.886 0.825 0.753 0.669 0.492 0.880 0.657 0.593 0.915 0.784 0.863 0.928 0.721 1.056 1.075 0.919 1.576 1.356 0.469 0.807 0.601 0.683 0.323 0.831 0.968 3.87

S. saevissima 46 Brazil, Sergipe, Rt. 101, Propria 31 0.180 0.769 0.624 0.607 0.594 0.518 0.354 0.680 0.473 0.519 0.678 0.536 0.658 0.672 0.524 0.832 0.843 0.664 1.187 1.061 0.340 0.643 0.458 0.569 0.256 0.579 0.697 2.98

S. saevissima 46 Brazil, Sergipe, Rt. 101, Propria 32 0.150 0.783 0.641 0.592 0.616 0.570 0.348 0.708 0.491 0.502 0.698 0.559 0.668 0.674 0.544 0.789 0.860 0.705 1.196 1.066 0.347 0.630 0.464 0.564 0.251 0.656 0.727 3.07

S. saevissima 46 Brazil, Sergipe, Rt. 101, Propria 33 0.140 0.770 0.635 0.585 0.606 0.507 0.348 0.685 0.467 0.514 0.684 0.518 0.649 0.656 0.517 0.797 0.841 0.641 1.172 1.071 0.329 0.631 0.460 0.526 0.248 0.641 0.696 3.01

S. saevissima 46 Brazil, Sergipe, Rt. 101, Propria 34 0.140 0.777 0.635 0.596 0.613 0.502 0.354 0.704 0.480 0.525 0.669 0.529 0.656 0.693 0.524 0.808 0.855 0.677 1.197 1.073 0.352 0.648 0.459 0.561 0.245 0.680 0.675 3.09

S. saevissima 46 Brazil, Sergipe, Rt. 101, Propria 35 0.150 0.781 0.654 0.588 0.615 0.545 0.343 0.696 0.519 0.493 0.666 0.563 0.658 0.669 0.537 0.796 0.815 0.663 1.204 1.033 0.373 0.625 0.460 0.603 0.228 0.699 0.676 3.12

S. saevissima 46 Brazil, Sergipe, Rt. 101, Propria 36 0.340 0.758 0.687 0.637 0.644 0.576 0.368 0.719 0.532 0.518 0.714 0.555 0.747 0.712 0.571 0.853 0.916 0.692 1.250 1.142 0.336 0.651 0.508 0.562 0.282 0.671 0.726 3.13

S. saevissima 46 Brazil, Sergipe, Rt. 101, Propria 37 0.120 0.708 0.613 0.586 0.572 0.487 0.349 0.663 0.462 0.502 0.647 0.503 0.614 0.652 0.490 0.773 0.790 0.640 1.097 0.985 0.319 0.607 0.399 0.621 0.242 0.620 0.637 2.93

S. saevissima 46 Brazil, Sergipe, Rt. 101, Propria 38 0.100 0.771 0.645 0.582 0.604 0.539 0.364 0.688 0.520 0.471 0.677 0.503 0.667 0.642 0.525 0.803 0.827 0.671 1.195 1.018 0.346 0.626 0.444 0.584 0.241 0.613 0.686 2.99

S. saevissima 46 Brazil, Sergipe, Rt. 101, Propria 39 0.110 0.772 0.663 0.600 0.642 0.559 0.351 0.720 0.511 0.485 0.695 0.554 0.669 0.685 0.543 0.834 0.858 0.674 1.231 1.064 0.368 0.659 0.451 0.617 0.257 0.630 0.681 3.08

S. saevissima 46 Brazil, Sergipe, Rt. 101, Propria 40 0.110 0.769 0.624 0.585 0.612 0.524 0.359 0.678 0.476 0.516 0.654 0.540 0.657 0.680 0.519 0.800 0.804 0.647 1.207 0.988 0.330 0.617 0.398 0.592 0.250 0.633 0.680 2.98

S. amblychila 47 AZ, Cochise Co., 1 mi. NW Portal 1 0.250 0.546 0.605 0.551 0.508 0.418 0.279 0.482 0.335 0.381 0.481 0.386 0.492 0.473 0.346 0.519 0.575 0.440 0.819 0.826 0.257 0.517 0.356 0.467 0.220 0.567 0.562 2.41

S. amblychila 47 AZ, Cochise Co., 1 mi. NW Portal 2 0.120 0.652 0.562 0.562 0.535 0.471 0.275 0.493 0.386 0.376 0.575 0.399 0.460 0.469 0.330 0.551 0.588 0.439 0.836 0.846 0.262 0.519 0.350 0.455 0.219 0.537 0.567 2.49

S. amblychila 47 AZ, Cochise Co., 1 mi. NW Portal 3 0.570 1.193 1.230 1.246 1.102 0.888 0.565 0.814 0.599 0.545 0.921 0.675 0.866 0.857 0.672 0.958 0.993 0.812 1.443 1.541 0.566 0.991 0.698 0.780 0.457 0.967 1.188 4.48

S. amblychila 47 AZ, Cochise Co., 1 mi. NW Portal 4 0.360 1.081 1.080 1.097 0.953 0.775 0.518 0.740 0.585 0.494 0.802 0.641 0.717 0.788 0.605 0.881 0.932 0.748 1.378 1.342 0.535 0.867 0.567 0.775 0.428 0.839 1.094 4.04

S. amblychila 47 AZ, Cochise Co., 1 mi. NW Portal 5 0.500 1.087 1.129 1.124 1.012 0.814 0.560 0.777 0.592 0.507 0.891 0.627 0.752 0.794 0.616 0.885 0.949 0.791 1.360 1.436 0.509 0.928 0.604 0.852 0.446 0.839 1.094 4.21

S. amblychila 47 AZ, Cochise Co., 1 mi. NW Portal 6 0.070 0.695 0.615 0.599 0.585 0.498 0.346 0.545 0.375 0.429 0.563 0.466 0.534 0.497 0.414 0.598 0.628 0.526 0.927 0.943 0.316 0.589 0.390 0.505 0.257 0.638 0.647 2.78

S. amblychila 47 AZ, Cochise Co., 1 mi. NW Portal 7 0.140 0.804 0.739 0.733 0.691 0.540 0.387 0.612 0.465 0.440 0.645 0.462 0.648 0.616 0.459 0.647 0.717 0.564 1.036 1.043 0.348 0.654 0.462 0.589 0.291 0.709 0.722 3.15

S. amblychila 47 AZ, Cochise Co., 1 mi. NW Portal 8 0.050 0.626 0.562 0.545 0.524 0.449 0.311 0.518 0.364 0.381 0.520 0.412 0.500 0.482 0.360 0.542 0.549 0.473 0.846 0.860 0.290 0.532 0.388 0.438 0.225 0.568 0.599 2.49

S. amblychila 47 AZ, Cochise Co., 1 mi. NW Portal 9 0.210 0.909 0.840 0.808 0.765 0.594 0.435 0.684 0.499 0.469 0.726 0.531 0.658 0.647 0.526 0.768 0.761 0.672 1.168 1.141 0.418 0.710 0.505 0.594 0.316 0.729 0.814 3.37

S. amblychila 47 AZ, Cochise Co., 1 mi. NW Portal 10 0.430 1.139 1.129 1.123 0.985 0.787 0.539 0.769 0.581 0.510 0.870 0.631 0.725 0.787 0.648 0.888 0.973 0.775 1.369 1.384 0.483 0.889 0.600 0.750 0.396 1.006 1.063 4.28

S. amblychila 47 AZ, Cochise Co., 1 mi. NW Portal 11 0.520 1.139 1.162 1.179 1.049 0.818 0.487 0.767 0.574 0.526 0.892 0.651 0.791 0.831 0.643 0.914 0.997 0.789 1.422 1.467 0.541 0.944 0.641 0.797 0.428 1.003 1.173 4.41

S. amblychila 47 AZ, Cochise Co., 1 mi. NW Portal 12 0.110 0.623 0.551 0.553 0.492 0.444 0.296 0.487 0.354 0.381 0.489 0.378 0.505 0.474 0.341 0.539 0.564 0.456 0.829 0.880 0.306 0.570 0.359 0.435 0.218 0.483 0.574 2.42

S. amblychila 47 AZ, Cochise Co., 1 mi. NW Portal 13 0.540 1.145 1.198 1.209 1.048 0.877 0.507 0.785 0.586 0.522 0.845 0.665 0.811 0.795 0.620 0.928 0.943 0.795 1.388 1.460 0.573 0.945 0.670 0.817 0.424 0.989 1.187 4.41

S. amblychila 47 AZ, Cochise Co., 1 mi. NW Portal 14 0.090 0.608 0.568 0.536 0.523 0.434 0.305 0.503 0.358 0.376 0.495 0.385 0.495 0.447 0.360 0.540 0.559 0.449 0.856 0.855 0.257 0.526 0.355 0.469 0.214 0.508 0.578 2.44

S. amblychila 47 AZ, Cochise Co., 1 mi. NW Portal 15 0.100 0.706 0.637 0.610 0.578 0.481 0.311 0.550 0.383 0.417 0.542 0.416 0.558 0.482 0.390 0.621 0.617 0.516 0.938 0.932 0.316 0.564 0.418 0.516 0.196 0.638 0.652 2.79

S. amblychila 47 AZ, Cochise Co., 1 mi. NW Portal 16 0.130 0.738 0.685 0.658 0.635 0.525 0.364 0.575 0.392 0.438 0.598 0.410 0.567 0.567 0.431 0.639 0.676 0.531 0.962 1.012 0.364 0.632 0.452 0.558 0.264 0.624 0.724 2.93

S. amblychila 47 AZ, Cochise Co., 1 mi. NW Portal 17 0.210 0.733 0.652 0.620 0.625 0.481 0.258 0.542 0.375 0.444 0.578 0.439 0.563 0.522 0.418 0.647 0.650 0.528 0.964 0.973 0.374 0.584 0.421 0.514 0.261 0.647 0.674 2.87

S. amblychila 47 AZ, Cochise Co., 1 mi. NW Portal 18 0.110 0.776 0.685 0.645 0.658 0.546 0.387 0.581 0.441 0.434 0.603 0.469 0.571 0.552 0.412 0.655 0.683 0.565 0.995 0.973 0.353 0.624 0.432 0.484 0.285 0.585 0.661 2.82

S. amblychila 47 AZ, Cochise Co., 1 mi. NW Portal 19 0.090 0.704 0.660 0.620 0.599 0.470 0.325 0.547 0.400 0.420 0.567 0.390 0.538 0.487 0.395 0.632 0.644 0.478 0.970 0.950 0.331 0.573 0.417 0.457 0.236 0.700 0.783 2.81

S. amblychila 47 AZ, Cochise Co., 1 mi. NW Portal 20 0.170 0.866 0.802 0.775 0.759 0.588 0.431 0.638 0.461 0.458 0.622 0.508 0.643 0.632 0.486 0.738 0.773 0.646 1.113 1.161 0.401 0.720 0.503 0.584 0.299 0.715 0.793 3.33

S. amblychila 47 AZ, Cochise Co., 1 mi. NW Portal 21 0.610 1.166 1.289 1.321 1.127 0.968 0.490 0.781 0.615 0.545 0.909 0.657 0.816 0.840 0.638 0.940 1.007 0.803 1.439 1.471 0.567 1.002 0.648 0.821 0.432 0.954 1.254 4.41

S. amblychila 47 AZ, Cochise Co., 1 mi. NW Portal 22 0.630 1.203 1.289 1.305 1.127 0.920 0.565 0.718 0.638 0.534 0.912 0.658 0.852 0.884 0.642 0.945 1.033 0.800 1.468 1.605 0.696 1.049 0.681 0.924 0.448 1.067 1.233 4.80

S. amblychila 47 AZ, Cochise Co., 1 mi. NW Portal 23 0.600 1.220 1.257 1.295 1.098 0.840 0.575 0.820 0.632 0.522 0.929 0.678 0.799 0.881 0.673 0.948 1.019 0.786 1.440 1.614 0.660 1.105 0.666 0.926 0.482 0.997 1.243 4.76

S. amblychila 47 AZ, Cochise Co., 1 mi. NW Portal 24 0.630 1.210 1.311 1.360 1.123 0.964 0.536 0.787 0.653 0.517 0.937 0.687 0.800 0.869 0.669 0.936 1.036 0.827 1.448 1.591 0.642 1.045 0.679 0.831 0.477 0.918 1.294 4.55

S. amblychila 47 AZ, Cochise Co., 1 mi. NW Portal 25 0.570 1.209 1.294 1.305 1.141 0.984 0.579 0.806 0.637 0.540 0.952 0.719 0.811 0.866 0.676 1.032 1.012 0.823 1.471 1.615 0.647 1.055 0.722 0.871 0.493 1.018 1.347 4.71

S. amblychila 47 AZ, Cochise Co., 1 mi. NW Portal 26 0.690 1.244 1.326 1.326 1.134 0.917 0.534 0.842 0.634 0.541 0.894 0.689 0.859 0.900 0.663 0.976 1.062 0.789 1.481 1.593 0.652 1.090 0.715 0.841 0.487 1.033 1.316 4.71

S. amblychila 47 AZ, Cochise Co., 1 mi. NW Portal 27 0.730 1.246 1.310 1.321 1.134 0.888 0.572 0.797 0.630 0.585 0.899 0.703 0.821 0.886 0.672 0.990 1.042 0.834 1.502 1.557 0.645 1.045 0.708 0.799 0.455 1.006 1.364 4.61

S. amblychila 47 AZ, Cochise Co., 1 mi. NW Portal 28 0.540 1.123 1.171 1.176 1.027 0.791 0.551 0.772 0.593 0.536 0.884 0.690 0.786 0.825 0.660 0.924 0.968 0.820 1.414 1.495 0.616 0.985 0.625 0.773 0.444 0.942 1.214 4.33

S. amblychila 47 AZ, Cochise Co., 1 mi. NW Portal 29 0.590 1.155 1.235 1.251 1.064 0.802 0.510 0.768 0.653 0.516 0.798 0.687 0.771 0.857 0.632 0.914 1.007 0.794 1.424 1.502 0.637 1.002 0.677 0.767 0.476 1.123 1.253 4.55

S. amblychila 47 AZ, Cochise Co., 1 mi. NW Portal 30 0.430 1.043 1.091 1.096 0.963 0.749 0.514 0.751 0.585 0.526 0.720 0.608 0.749 0.783 0.617 0.836 0.838 0.773 1.303 1.393 0.539 0.895 0.581 0.660 0.414 0.904 1.160 4.00

S. amblychila 47 AZ, Cochise Co., 1 mi. NW Portal 31 0.060 0.645 0.563 0.552 0.519 0.423 0.281 0.490 0.368 0.387 0.499 0.367 0.481 0.429 0.357 0.545 0.556 0.446 0.829 0.844 0.290 0.526 0.357 0.481 0.220 0.548 0.525 2.52

S. amblychila 47 AZ, Cochise Co., 1 mi. NW Portal 32 0.120 0.658 0.572 0.567 0.535 0.433 0.320 0.505 0.366 0.371 0.525 0.374 0.504 0.499 0.364 0.569 0.574 0.485 0.873 0.888 0.262 0.533 0.375 0.470 0.237 0.551 0.672 2.57

S. amblychila 47 AZ, Cochise Co., 1 mi. NW Portal 33 0.090 0.615 0.540 0.535 0.51 0.444 0.271 0.504 0.371 0.369 0.481 0.394 0.481 0.455 0.342 0.528 0.533 0.423 0.836 0.825 0.305 0.512 0.352 0.499 0.219 0.491 0.612 2.43

S. amblychila 47 AZ, Cochise Co., 1 mi. NW Portal 34 0.100 0.663 0.583 0.562 0.54 0.449 0.325 0.514 0.372 0.400 0.535 0.407 0.515 0.498 0.371 0.524 0.569 0.502 0.876 0.893 0.277 0.551 0.378 0.471 0.230 0.516 0.617 2.54

S. amblychila 47 AZ, Cochise Co., 1 mi. NW Portal 35 0.130 0.659 0.584 0.557 0.545 0.498 0.304 0.555 0.381 0.394 0.520 0.446 0.515 0.523 0.406 0.548 0.613 0.492 0.937 0.884 0.285 0.569 0.381 0.455 0.243 0.596 0.612 2.59

S. amblychila 47 AZ, Cochise Co., 1 mi. NW Portal 36 0.070 0.674 0.594 0.561 0.542 0.460 0.326 0.524 0.386 0.389 0.521 0.390 0.503 0.473 0.371 0.594 0.605 0.468 0.900 0.877 0.294 0.555 0.395 0.436 0.231 0.572 0.650 2.56

S. amblychila 47 AZ, Cochise Co., 1 mi. NW Portal 37 0.050 0.621 0.524 0.503 0.476 0.412 0.301 0.477 0.339 0.375 0.466 0.363 0.446 0.417 0.345 0.521 0.508 0.462 0.794 0.789 0.263 0.507 0.343 0.462 0.216 0.505 0.545 2.38

S. amblychila 47 AZ, Cochise Co., 1 mi. NW Portal 38 0.060 0.642 0.545 0.519 0.508 0.465 0.296 0.484 0.371 0.365 0.483 0.348 0.481 0.425 0.357 0.528 0.561 0.445 0.865 0.809 0.262 0.512 0.359 0.525 0.229 0.487 0.648 2.46

S. amblychila 47 AZ, Cochise Co., 1 mi. NW Portal 39 0.080 0.714 0.630 0.614 0.59 0.528 0.346 0.545 0.404 0.412 0.563 0.471 0.539 0.519 0.393 0.601 0.649 0.514 0.964 0.925 0.313 0.609 0.404 0.531 0.247 0.503 0.615 2.67

S. amblychila 47 AZ, Cochise Co., 1 mi. NW Portal 40 0.110 0.616 0.540 0.530 0.492 0.402 0.280 0.477 0.354 0.353 0.478 0.362 0.445 0.383 0.333 0.506 0.511 0.409 0.804 0.803 0.230 0.506 0.357 0.419 0.215 0.510 0.566 2.35

S. geminata 49 Florida, Columbia Co. 1 0.250 0.706 0.667 0.617 0.623 0.534 0.358 0.608 0.455 0.444 0.638 0.487 0.632 0.626 0.483 0.695 0.788 0.617 1.037 1.016 0.365 0.595 0.440 0.560 0.249 0.604 0.643 2.89

S. geminata 49 Florida, Columbia Co. 2 0.790 1.040 0.994 0.933 0.928 0.824 0.486 0.761 0.563 0.504 0.802 0.627 0.699 0.767 0.581 0.883 0.887 0.744 1.241 1.252 0.472 0.756 0.536 0.668 0.367 0.810 0.845 3.77

S. geminata 49 Florida, Columbia Co. 3 0.280 0.818 0.734 0.678 0.678 0.621 0.369 0.656 0.474 0.482 0.680 0.525 0.619 0.646 0.519 0.716 0.825 0.649 1.101 1.078 0.386 0.648 0.465 0.610 0.265 0.654 0.676 3.16

S. geminata 49 Florida, Columbia Co. 4 0.710 1.181 1.157 1.091 1.096 0.957 0.573 0.893 0.646 0.575 0.901 0.740 0.834 0.912 0.725 0.972 1.094 0.870 1.487 1.466 0.566 0.875 0.645 0.756 0.380 1.019 1.027 4.42

S. geminata 49 Florida, Columbia Co. 5 0.220 0.777 0.704 0.654 0.655 0.582 0.389 0.642 0.465 0.463 0.637 0.540 0.605 0.604 0.498 0.733 0.804 0.617 1.060 1.023 0.369 0.630 0.470 0.568 0.269 0.649 0.677 3.02

S. geminata 49 Florida, Columbia Co. 6 0.190 0.744 0.670 0.615 0.641 0.554 0.315 0.641 0.447 0.455 0.657 0.535 0.618 0.634 0.493 0.693 0.794 0.594 1.083 1.021 0.371 0.615 0.440 0.569 0.257 0.640 0.653 2.97

S. geminata 49 Florida, Columbia Co. 7 0.250 0.759 0.676 0.629 0.63 0.550 0.381 0.620 0.435 0.465 0.593 0.492 0.570 0.603 0.460 0.689 0.731 0.588 1.057 0.988 0.332 0.603 0.442 0.542 0.249 0.620 0.627 2.91

S. geminata 49 Florida, Columbia Co. 8 0.240 0.764 0.693 0.640 0.669 0.600 0.376 0.645 0.451 0.465 0.647 0.522 0.621 0.633 0.525 0.716 0.793 0.640 1.074 1.050 0.370 0.626 0.476 0.569 0.260 0.626 0.670 3.01

S. geminata 49 Florida, Columbia Co. 9 0.210 0.799 0.716 0.665 0.667 0.632 0.410 0.676 0.494 0.447 0.660 0.507 0.646 0.652 0.525 0.714 0.822 0.649 1.107 0.995 0.369 0.632 0.442 0.523 0.278 0.675 0.663 2.99

S. geminata 49 Florida, Columbia Co. 10 0.190 0.752 0.691 0.630 0.644 0.581 0.344 0.642 0.461 0.429 0.634 0.490 0.601 0.617 0.521 0.671 0.772 0.611 1.052 1.041 0.380 0.609 0.455 0.559 0.250 0.633 0.650 2.98

S. geminata 49 Florida, Columbia Co. 11 0.340 0.799 0.693 0.637 0.663 0.587 0.365 0.648 0.466 0.436 0.622 0.528 0.575 0.627 0.505 0.695 0.633 0.622 1.046 1.034 0.344 0.633 0.434 0.572 0.268 0.670 0.710 3.08

S. geminata 49 Florida, Columbia Co. 12 0.220 0.774 0.691 0.645 0.658 0.584 0.336 0.660 0.470 0.444 0.652 0.517 0.621 0.615 0.509 0.716 0.781 0.654 1.078 1.044 0.366 0.631 0.448 0.612 0.271 0.679 0.672 3.11

S. geminata 49 Florida, Columbia Co. 13 0.300 0.817 0.739 0.682 0.704 0.615 0.369 0.685 0.477 0.471 0.659 0.475 0.681 0.668 0.524 0.723 0.785 0.643 1.112 1.102 0.405 0.665 0.472 0.609 0.274 0.744 0.715 3.27

S. geminata 49 Florida, Columbia Co. 14 0.200 0.768 0.690 0.625 0.66 0.610 0.353 0.626 0.462 0.442 0.631 0.511 0.611 0.606 0.495 0.712 0.766 0.618 1.077 0.991 0.370 0.617 0.434 0.533 0.252 0.649 0.637 2.94

S. geminata 49 Florida, Columbia Co. 15 0.250 0.767 0.668 0.609 0.63 0.566 0.361 0.631 0.492 0.391 0.404 0.485 0.634 0.596 0.484 0.693 0.765 0.599 1.051 0.977 0.340 0.598 0.439 0.528 0.262 0.657 0.640 2.93

S. geminata 49 Florida, Columbia Co. 16 0.200 0.755 0.671 0.626 0.633 0.571 0.355 0.634 0.459 0.432 0.567 0.518 0.582 0.617 0.490 0.701 0.737 0.590 1.046 1.001 0.371 0.601 0.450 0.555 0.251 0.630 0.631 2.94

S. geminata 49 Florida, Columbia Co. 17 0.230 0.811 0.710 0.659 0.693 0.615 0.319 0.680 0.481 0.425 0.685 0.515 0.620 0.641 0.520 0.721 0.823 0.648 1.108 1.065 0.366 0.654 0.474 0.593 0.277 0.645 0.658 3.11

S. geminata 49 Florida, Columbia Co. 18 0.200 0.760 0.670 0.615 0.641 0.564 0.345 0.632 0.439 0.446 0.628 0.472 0.606 0.596 0.497 0.687 0.789 0.593 1.030 0.998 0.355 0.624 0.427 0.563 0.260 0.649 0.615 2.97

S. geminata 49 Florida, Columbia Co. 19 0.220 0.766 0.682 0.632 0.641 0.583 0.391 0.649 0.444 0.453 0.630 0.526 0.610 0.611 0.494 0.705 0.805 0.615 1.092 1.019 0.376 0.624 0.435 0.572 0.253 0.610 0.627 2.97

S. geminata 49 Florida, Columbia Co. 20 0.230 0.801 0.712 0.667 0.675 0.600 0.382 0.670 0.511 0.437 0.661 0.535 0.654 0.632 0.521 0.738 0.762 0.669 1.116 1.049 0.364 0.648 0.462 0.600 0.274 0.660 0.688 3.11

S. geminata 49 Florida, Columbia Co. 21 2.980 1.773 2.088 2.030 2.091 1.713 0.809 1.083 0.817 0.617 1.156 0.898 0.996 1.031 0.875 1.153 1.317 1.074 1.714 1.908 0.827 1.278 0.838 0.982 0.584 1.226 1.410 5.89

S. geminata 49 Florida, Columbia Co. 22 2.010 1.615 1.894 1.827 1.901 1.525 0.767 1.037 0.762 0.565 1.094 0.896 0.954 1.098 0.852 1.136 1.223 0.987 1.655 1.844 0.783 1.168 0.789 0.910 0.522 1.048 1.323 5.42

S. geminata 49 Florida, Columbia Co. 23 1.650 1.549 1.703 1.659 1.669 1.371 0.719 0.999 0.735 0.590 1.045 0.850 0.986 1.031 0.868 1.102 1.223 0.979 1.693 1.699 0.658 1.067 0.739 0.932 0.492 1.140 1.257 5.32

S. geminata 49 Florida, Columbia Co. 24 1.810 1.593 1.816 1.726 1.788 1.419 0.664 1.025 0.737 0.578 1.074 0.878 0.919 1.053 0.831 1.125 1.252 0.989 1.654 1.777 0.684 1.127 0.742 0.946 0.519 1.226 1.288 5.54

S. geminata 49 Florida, Columbia Co. 25 1.700 1.514 1.743 1.682 1.732 1.291 0.688 1.020 0.799 0.601 1.082 0.896 0.969 1.048 0.815 1.131 1.214 0.997 1.631 1.722 0.654 1.087 0.741 0.919 0.497 1.136 1.302 5.29

S. geminata 49 Florida, Columbia Co. 26 1.300 1.441 1.628 1.563 1.56 1.273 0.710 0.997 0.707 0.593 0.937 0.853 0.921 1.028 0.813 1.061 1.163 0.952 1.610 1.665 0.607 1.001 0.730 0.902 0.482 1.078 1.213 5.09

S. geminata 49 Florida, Columbia Co. 27 1.370 1.453 1.571 1.504 1.518 1.274 0.721 0.946 0.708 0.591 1.000 0.867 0.916 0.991 0.805 1.053 1.163 0.930 1.597 1.620 0.626 0.976 0.709 0.857 0.447 1.084 1.185 5.01

S. geminata 49 Florida, Columbia Co. 28 1.500 1.392 1.637 1.581 1.565 1.299 0.665 0.953 0.735 0.602 1.032 0.789 0.927 1.032 0.783 1.088 1.157 0.931 1.589 1.661 0.637 1.008 0.742 0.896 0.495 1.147 1.220 5.10

S. geminata 49 Florida, Columbia Co. 29 1.090 1.355 1.445 1.396 1.379 1.154 0.678 0.938 0.684 0.578 0.967 0.809 0.893 0.984 0.763 1.049 1.232 0.953 1.556 1.579 0.561 0.959 0.717 0.794 0.446 1.040 1.184 4.77

S. geminata 49 Florida, Columbia Co. 30 1.000 1.324 1.464 1.402 1.386 1.184 0.660 0.951 0.713 0.600 0.890 0.789 0.913 0.897 0.768 1.060 1.162 0.933 1.590 1.538 0.608 0.945 0.694 0.838 0.442 0.999 1.111 4.70

S. geminata 49 Florida, Columbia Co. 31 0.230 0.738 0.649 0.610 0.615 0.504 0.366 0.601 0.431 0.450 0.628 0.497 0.632 0.584 0.480 0.695 0.756 0.600 1.039 0.976 0.332 0.597 0.420 0.587 0.247 0.702 0.615 3.00

S. geminata 49 Florida, Columbia Co. 32 0.270 0.766 0.667 0.615 0.639 0.570 0.346 0.635 0.457 0.465 0.525 0.522 0.591 0.535 0.492 0.698 0.721 0.640 1.022 0.960 0.355 0.583 0.419 0.579 0.257 0.669 0.630 2.97

S. geminata 49 Florida, Columbia Co. 33 0.290 0.752 0.679 0.629 0.635 0.569 0.362 0.648 0.439 0.448 0.643 0.518 0.593 0.620 0.470 0.690 0.748 0.596 1.025 0.962 0.330 0.610 0.445 0.560 0.263 0.669 0.647 2.94

S. geminata 49 Florida, Columbia Co. 34 0.270 0.738 0.661 0.610 0.624 0.549 0.317 0.609 0.423 0.423 0.611 0.502 0.605 0.596 0.456 0.704 0.731 0.574 1.036 0.984 0.341 0.587 0.435 0.537 0.243 0.592 0.587 2.85

S. geminata 49 Florida, Columbia Co. 35 0.200 0.726 0.648 0.587 0.603 0.559 0.377 0.603 0.401 0.468 0.572 0.479 0.590 0.561 0.453 0.671 0.732 0.596 1.005 0.985 0.316 0.606 0.428 0.609 0.250 0.641 0.607 2.96

S. geminata 49 Florida, Columbia Co. 36 0.190 0.745 0.663 0.606 0.629 0.573 0.379 0.627 0.468 0.440 0.636 0.528 0.586 0.609 0.498 0.720 0.796 0.628 1.057 1.014 0.349 0.606 0.447 0.593 0.263 0.621 0.640 2.97

S. geminata 49 Florida, Columbia Co. 37 0.240 0.761 0.682 0.637 0.645 0.559 0.384 0.649 0.454 0.459 0.577 0.503 0.623 0.622 0.515 0.728 0.773 0.632 1.095 1.022 0.375 0.622 0.459 0.558 0.242 0.654 0.670 3.00

S. geminata 49 Florida, Columbia Co. 38 0.330 0.785 0.707 0.644 0.662 0.576 0.347 0.655 0.442 0.465 0.667 0.533 0.615 0.638 0.510 0.717 0.833 0.636 1.090 1.041 0.391 0.617 0.451 0.606 0.275 0.715 0.665 3.15

S. geminata 49 Florida, Columbia Co. 39 0.180 0.738 0.660 0.598 0.607 0.531 0.368 0.605 0.454 0.421 0.593 0.501 0.588 0.578 0.445 0.682 0.745 0.581 0.999 0.996 0.376 0.605 0.440 0.526 0.257 0.637 0.615 2.90

S. geminata 49 Florida, Columbia Co. 40 0.223 0.743 0.637 0.603 0.603 0.531 0.361 0.631 0.437 0.439 0.606 0.480 0.602 0.585 0.447 0.672 0.771 0.575 1.031 0.969 0.346 0.582 0.422 0.572 0.253 0.688 0.613 2.97

S. geminata 50 Florida, Columbia Co. 1 0.220 0.791 0.704 0.658 0.65 0.541 0.375 0.666 0.477 0.478 0.667 0.564 0.593 0.652 0.499 0.752 0.811 0.622 1.109 1.100 0.404 0.649 0.491 0.581 0.274 0.727 0.719 3.20

S. geminata 50 Florida, Columbia Co. 2 0.210 0.726 0.632 0.589 0.563 0.491 0.351 0.605 0.383 0.447 0.584 0.466 0.590 0.568 0.447 0.660 0.711 0.570 0.974 0.945 0.326 0.587 0.407 0.541 0.240 0.626 0.647 2.84

S. geminata 50 Florida, Columbia Co. 3 0.770 1.227 1.262 1.209 1.196 0.971 0.635 0.893 0.703 0.567 0.959 0.756 0.863 0.937 0.736 0.869 1.158 0.906 1.507 1.515 0.566 0.930 0.628 0.780 0.414 1.055 1.073 4.58

S. geminata 50 Florida, Columbia Co. 4 0.180 0.844 0.750 0.708 0.697 0.586 0.418 0.682 0.527 0.451 0.699 0.539 0.674 0.667 0.545 0.767 0.816 0.687 1.128 1.131 0.414 0.691 0.494 0.593 0.308 0.670 0.710 3.24

S. geminata 50 Florida, Columbia Co. 5 1.130 1.423 1.480 1.449 1.434 1.144 0.688 0.971 0.707 0.640 0.952 0.793 0.923 1.026 0.788 1.062 1.150 0.962 1.567 1.628 0.576 0.997 0.742 0.746 0.459 1.042 1.194 4.84

S. geminata 50 Florida, Columbia Co. 6 0.370 0.875 0.783 0.742 0.731 0.602 0.387 0.706 0.535 0.461 0.724 0.578 0.667 0.695 0.556 0.773 0.893 0.673 1.165 1.177 0.401 0.729 0.504 0.693 0.306 0.669 0.768 3.41

S. geminata 50 Florida, Columbia Co. 7 0.200 0.727 0.646 0.605 0.589 0.489 0.338 0.588 0.438 0.434 0.589 0.475 0.573 0.572 0.434 0.692 0.686 0.563 0.966 0.943 0.349 0.576 0.381 0.552 0.240 0.576 0.628 2.80

S. geminata 50 Florida, Columbia Co. 8 0.240 0.775 0.689 0.654 0.655 0.579 0.376 0.673 0.484 0.469 0.693 0.529 0.629 0.646 0.499 0.774 0.809 0.645 1.083 1.093 0.392 0.649 0.475 0.571 0.281 0.675 0.686 3.11

S. geminata 50 Florida, Columbia Co. 9 0.480 1.070 0.995 0.968 0.946 0.784 0.505 0.815 0.600 0.515 0.831 0.660 0.756 0.847 0.631 0.917 1.026 0.787 1.330 1.338 0.512 0.848 0.601 0.661 0.364 0.855 0.901 3.92

S. geminata 50 Florida, Columbia Co. 10 0.220 0.808 0.727 0.686 0.693 0.593 0.364 0.681 0.505 0.471 0.699 0.564 0.645 0.666 0.553 0.734 0.840 0.676 1.094 1.116 0.390 0.671 0.493 0.605 0.297 0.722 0.705 3.25

S. geminata 50 Florida, Columbia Co. 11 0.350 0.965 0.895 0.849 0.858 0.709 0.460 0.681 0.505 0.471 0.791 0.628 0.676 0.791 0.599 0.874 0.961 0.742 1.259 1.262 0.450 0.778 0.539 0.703 0.335 0.826 0.823 3.76

S. geminata 50 Florida, Columbia Co. 12 0.230 0.669 0.750 0.635 0.621 0.518 0.351 0.636 0.484 0.424 0.650 0.476 0.598 0.614 0.461 0.706 0.730 0.593 1.044 1.047 0.346 0.624 0.455 0.526 0.266 0.659 0.630 2.90

S. geminata 50 Florida, Columbia Co. 13 0.230 0.764 0.660 0.615 0.616 0.570 0.392 0.623 0.450 0.460 0.628 0.510 0.567 0.629 0.476 0.741 0.760 0.616 1.059 1.021 0.403 0.634 0.436 0.620 0.252 0.635 0.635 3.04

S. geminata 50 Florida, Columbia Co. 14 0.210 0.769 0.687 0.653 0.641 0.553 0.359 0.637 0.466 0.437 0.664 0.542 0.583 0.647 0.502 0.715 0.794 0.621 1.066 1.055 0.384 0.648 0.447 0.574 0.278 0.727 0.686 3.12

S. geminata 50 Florida, Columbia Co. 15 0.630 1.233 1.198 1.145 1.144 0.948 0.624 0.862 0.662 0.564 0.928 0.724 0.838 0.914 0.693 0.974 1.096 0.851 1.448 1.448 0.512 0.900 0.635 0.712 0.397 1.000 1.023 4.39

S. geminata 50 Florida, Columbia Co. 16 1.380 1.460 1.587 1.605 1.565 1.308 0.668 0.971 0.710 0.585 1.096 0.826 0.914 1.046 0.779 1.036 1.224 0.952 1.536 1.694 0.645 1.039 0.744 0.910 0.473 1.123 1.286 5.19

S. geminata 50 Florida, Columbia Co. 17 0.190 0.798 0.709 0.656 0.679 0.564 0.409 0.662 0.500 0.437 0.679 0.500 0.618 0.641 0.507 0.718 0.799 0.619 1.062 1.094 0.366 0.649 0.478 0.550 0.280 0.679 0.656 3.12

S. geminata 50 Florida, Columbia Co. 18 0.210 0.820 0.721 0.686 0.694 0.587 0.429 0.691 0.457 0.492 0.701 0.534 0.631 0.663 0.504 0.742 0.852 0.645 1.100 1.105 0.397 0.656 0.500 0.576 0.279 0.686 0.738 3.19

S. geminata 50 Florida, Columbia Co. 19 1.010 1.320 1.419 1.386 1.398 1.102 0.712 0.936 0.707 0.594 0.988 0.804 0.894 1.015 0.791 1.042 1.209 0.921 1.516 1.603 0.624 1.002 0.690 0.775 0.444 1.030 1.123 4.73

S. geminata 50 Florida, Columbia Co. 20 0.230 0.808 0.721 0.669 0.69 0.587 0.379 0.686 0.507 0.456 0.691 0.526 0.631 0.675 0.517 0.715 0.836 0.663 1.078 1.120 0.390 0.680 0.484 0.582 0.287 0.707 0.697 3.22

S. geminata 50 Florida, Columbia Co. 21 5.510 1.931 2.332 2.303 2.313 1.884 1.058 1.155 0.849 0.665 1.202 0.936 1.042 1.110 0.932 1.212 1.347 1.109 1.751 2.187 1.012 1.520 0.942 1.057 0.686 1.413 1.669 6.59

S. geminata 50 Florida, Columbia Co. 22 2.260 1.797 2.030 2.036 2.099 1.605 0.658 1.055 0.785 0.619 1.160 0.904 0.967 1.054 0.822 1.152 1.266 1.032 1.704 2.035 0.779 1.317 0.863 0.986 0.579 1.295 1.502 6.11

S. geminata 50 Florida, Columbia Co. 23 2.250 1.756 2.041 1.995 2.064 1.628 0.716 1.067 0.804 0.604 1.138 0.849 0.989 1.099 0.896 1.117 1.282 1.038 1.628 1.901 0.778 1.258 0.789 0.884 0.539 1.208 1.355 5.75

S. geminata 50 Florida, Columbia Co. 24 1.590 1.576 1.791 1.721 1.762 1.471 0.767 1.010 0.754 0.606 1.088 0.816 0.983 1.060 0.880 1.100 1.250 0.965 1.669 1.797 0.664 1.117 0.787 0.904 0.534 1.228 1.380 5.50

S. geminata 50 Florida, Columbia Co. 25 1.290 1.453 1.566 1.515 1.493 1.128 0.619 0.966 0.725 0.611 1.075 0.802 0.965 1.041 0.821 1.134 1.174 0.974 1.612 1.691 0.669 1.030 0.741 0.816 0.471 1.070 1.199 5.03

S. geminata 50 Florida, Columbia Co. 26 1.820 1.698 1.901 1.831 1.885 1.489 0.757 1.027 0.767 0.601 1.147 0.863 0.959 1.124 0.826 1.117 1.298 0.997 1.658 1.860 0.725 1.210 0.796 0.890 0.546 1.224 1.412 5.67

S. geminata 50 Florida, Columbia Co. 27 1.680 1.533 1.734 1.662 1.701 1.346 0.702 0.980 0.749 0.640 1.083 0.884 0.977 1.069 0.843 1.174 1.253 1.004 1.690 1.758 0.657 1.079 0.757 0.862 0.495 1.094 1.420 5.25

S. geminata 50 Florida, Columbia Co. 28 1.670 1.489 1.778 1.677 1.716 1.309 0.674 1.017 0.778 0.601 1.056 0.844 1.030 1.088 0.835 1.105 1.299 0.986 1.629 1.700 0.660 1.098 0.767 0.821 0.532 1.059 1.345 5.07

S. geminata 50 Florida, Columbia Co. 29 1.570 1.585 1.765 1.733 1.754 1.295 0.617 1.019 0.777 0.584 1.129 0.838 0.955 1.061 0.824 1.108 1.211 0.980 1.605 1.739 0.640 1.118 0.718 0.857 0.512 1.035 1.221 5.22

S. geminata 50 Florida, Columbia Co. 30 1.490 1.532 1.694 1.657 1.67 1.303 0.650 1.021 0.735 0.622 1.045 0.899 0.980 0.848 1.155 1.273 1.002 1.685 1.701 1.688 0.663 1.118 0.705 0.858 0.511 1.087 1.256 5.16

S. geminata 50 Florida, Columbia Co. 31 0.200 0.730 0.644 0.609 0.595 0.504 0.356 0.603 0.438 0.431 0.654 0.468 0.574 0.605 0.463 0.661 0.761 0.564 1.019 1.029 0.365 0.606 0.469 0.517 0.264 0.587 0.610 2.86

S. geminata 50 Florida, Columbia Co. 32 0.190 0.764 0.654 0.619 0.62 0.518 0.368 0.625 0.453 0.458 0.651 0.507 0.635 0.618 0.474 0.713 0.780 0.593 1.073 1.043 0.386 0.646 0.466 0.534 0.268 0.669 0.628 3.01

S. geminata 50 Florida, Columbia Co. 33 0.180 0.768 0.686 0.628 0.653 0.564 0.342 0.629 0.432 0.448 0.650 0.471 0.590 0.606 0.447 0.756 0.768 0.577 1.062 1.033 0.352 0.625 0.448 0.536 0.252 0.634 0.628 2.97

S. geminata 50 Florida, Columbia Co. 34 0.200 0.681 0.595 0.561 0.543 0.462 0.324 0.555 0.375 0.444 0.582 0.445 0.532 0.532 0.442 0.628 0.697 0.526 0.943 0.930 0.308 0.580 0.386 0.514 0.230 0.562 0.557 2.69

S. geminata 50 Florida, Columbia Co. 35 0.180 0.769 0.693 0.635 0.648 0.524 0.305 0.651 0.474 0.456 0.655 0.522 0.582 0.617 0.490 0.724 0.779 0.620 1.044 1.023 0.351 0.634 0.452 0.544 0.284 0.647 0.640 2.98

S. geminata 50 Florida, Columbia Co. 36 0.190 0.777 0.685 0.629 0.63 0.529 0.364 0.634 0.455 0.451 0.653 0.512 0.615 0.625 0.516 0.721 0.780 0.599 1.081 1.059 0.362 0.629 0.481 0.502 0.268 0.642 0.660 2.98

S. geminata 50 Florida, Columbia Co. 37 0.250 0.698 0.628 0.594 0.589 0.506 0.326 0.598 0.409 0.440 0.613 0.455 0.564 0.565 0.440 0.677 0.734 0.557 0.977 0.998 0.357 0.641 0.403 0.520 0.242 0.621 0.597 2.84

S. geminata 50 Florida, Columbia Co. 38 0.170 0.744 0.663 0.623 0.632 0.535 0.393 0.632 0.474 0.435 0.646 0.504 0.598 0.589 0.477 0.688 0.772 0.585 1.036 1.029 0.382 0.634 0.455 0.520 0.266 0.651 0.667 2.94

S. geminata 50 Florida, Columbia Co. 39 0.190 0.773 0.657 0.610 0.63 0.541 0.347 0.636 0.449 0.435 0.641 0.491 0.570 0.609 0.476 0.712 0.750 0.591 1.081 1.052 0.345 0.629 0.460 0.551 0.274 0.614 0.626 2.99

S. geminata 50 Florida, Columbia Co. 40 0.170 0.760 0.675 0.626 0.641 0.546 0.320 0.632 0.444 0.451 0.600 0.536 0.579 0.622 0.477 0.713 0.788 0.602 1.071 1.013 0.384 0.629 0.436 0.550 0.278 0.686 0.651 3.01

S. geminata 51 Florida, Columbia Co. 1 0.120 0.721 0.618 0.575 0.603 0.548 0.327 0.593 0.410 0.422 0.594 0.447 0.553 0.565 0.456 0.615 0.685 0.549 0.957 0.951 0.345 0.578 0.421 0.504 0.228 0.654 0.595 2.83

S. geminata 51 Florida, Columbia Co. 2 0.120 0.776 0.636 0.576 0.624 0.575 0.385 0.614 0.433 0.446 0.648 0.476 0.584 0.604 0.466 0.692 0.788 0.573 1.029 0.990 0.340 0.617 0.428 0.516 0.265 0.675 0.629 2.96

S. geminata 51 Florida, Columbia Co. 3 0.130 0.742 0.630 0.579 0.596 0.539 0.315 0.597 0.443 0.408 0.600 0.428 0.589 0.583 0.433 0.660 0.746 0.564 0.995 0.973 0.324 0.595 0.442 0.497 0.260 0.643 0.595 2.85

S. geminata 51 Florida, Columbia Co. 4 0.740 1.108 1.016 0.968 0.963 0.796 0.527 0.801 0.546 0.543 0.840 0.685 0.735 0.827 0.640 0.876 1.014 0.789 1.298 1.323 0.463 0.811 0.570 0.720 0.360 0.801 0.849 3.95

S. geminata 51 Florida, Columbia Co. 5 0.470 1.057 0.988 0.911 0.948 0.808 0.467 0.792 0.576 0.541 0.820 0.645 0.743 0.794 0.616 0.886 0.991 0.765 1.303 1.322 0.471 0.784 0.601 0.654 0.362 0.875 0.872 3.91

S. geminata 51 Florida, Columbia Co. 6 0.110 0.721 0.619 0.592 0.596 0.490 0.296 0.581 0.442 0.410 0.603 0.459 0.539 0.576 0.455 0.630 0.713 0.563 0.931 0.963 0.324 0.590 0.399 0.537 0.239 0.650 0.595 2.87

S. geminata 51 Florida, Columbia Co. 7 0.680 1.260 1.273 1.216 1.231 1.031 0.527 0.863 0.642 0.534 0.943 0.699 0.871 0.905 0.718 0.968 1.137 0.861 1.446 1.494 0.541 0.924 0.622 0.750 0.405 0.956 1.004 4.46

S. geminata 51 Florida, Columbia Co. 8 0.170 0.761 0.649 0.626 0.633 0.520 0.280 0.613 0.452 0.437 0.652 0.476 0.598 0.619 0.467 0.689 0.764 0.588 1.027 1.034 0.335 0.629 0.454 0.537 0.267 0.573 0.622 2.90

S. geminata 51 Florida, Columbia Co. 9 0.150 0.728 0.635 0.582 0.6 0.517 0.297 0.575 0.406 0.426 0.595 0.448 0.542 0.567 0.423 0.652 0.714 0.568 0.945 0.943 0.335 0.584 0.405 0.533 0.251 0.574 0.576 2.78

S. geminata 51 Florida, Columbia Co. 10 0.410 1.038 0.957 0.914 0.93 0.758 0.422 0.784 0.556 0.498 0.834 0.591 0.746 0.793 0.595 0.894 0.981 0.750 1.255 1.284 0.479 0.762 0.576 0.653 0.349 0.823 0.868 3.80

S. geminata 51 Florida, Columbia Co. 11 0.850 1.248 1.216 1.167 1.165 0.966 0.630 0.869 0.624 0.536 0.890 0.737 0.803 0.886 0.701 0.947 1.038 0.840 1.393 1.491 0.518 0.921 0.637 0.697 0.373 0.965 0.991 4.40

S. geminata 51 Florida, Columbia Co. 12 0.130 0.720 0.593 0.573 0.591 0.497 0.274 0.577 0.423 0.421 0.585 0.454 0.551 0.549 0.431 0.646 0.708 0.540 0.950 0.948 0.327 0.558 0.407 0.538 0.241 0.605 0.571 2.81

S. geminata 51 Florida, Columbia Co. 13 0.550 1.171 1.147 1.111 1.097 0.916 0.574 0.855 0.604 0.545 0.921 0.697 0.843 0.883 0.677 0.996 1.121 0.846 1.415 1.399 0.551 0.852 0.638 0.671 0.378 0.984 1.008 4.23

S. geminata 51 Florida, Columbia Co. 14 0.170 0.742 0.629 0.602 0.618 0.522 0.331 0.605 0.461 0.445 0.634 0.497 0.590 0.605 0.483 0.691 0.779 0.578 1.052 1.033 0.379 0.627 0.434 0.547 0.255 0.654 0.627 2.98

S. geminata 51 Florida, Columbia Co. 15 0.180 0.732 0.629 0.581 0.6 0.522 0.320 0.590 0.448 0.431 0.604 0.460 0.583 0.577 0.452 0.671 0.736 0.567 0.986 0.958 0.343 0.589 0.422 0.518 0.258 0.634 0.598 2.84

S. geminata 51 Florida, Columbia Co. 16 0.550 0.965 0.889 0.845 0.863 0.760 0.469 0.755 0.551 0.487 0.766 0.615 0.723 0.758 0.579 0.841 0.931 0.742 1.254 1.250 0.439 0.738 0.564 0.617 0.332 0.899 0.812 3.73

S. geminata 51 Florida, Columbia Co. 17 0.140 0.737 0.646 0.592 0.621 0.523 0.334 0.613 0.447 0.440 0.638 0.504 0.574 0.636 0.477 0.671 0.753 0.610 1.007 1.005 0.341 0.598 0.441 0.499 0.255 0.627 0.605 2.87

S. geminata 51 Florida, Columbia Co. 18 0.720 1.188 1.146 1.090 1.109 0.968 0.582 0.830 0.615 0.533 0.899 0.715 0.808 0.829 0.683 0.926 1.051 0.850 1.385 1.395 0.535 0.865 0.621 0.690 0.385 0.936 0.933 4.21

S. geminata 51 Florida, Columbia Co. 19 0.150 0.705 0.586 0.565 0.573 0.489 0.312 0.570 0.415 0.407 0.595 0.432 0.573 0.581 0.427 0.657 0.714 0.529 0.962 0.933 0.326 0.579 0.410 0.478 0.246 0.622 0.639 2.74

S. geminata 51 Florida, Columbia Co. 20 0.300 0.774 0.683 0.630 0.656 0.548 0.304 0.616 0.425 0.452 0.635 0.452 0.621 0.639 0.467 0.697 0.749 0.580 1.048 0.990 0.350 0.616 0.433 0.559 0.279 0.720 0.650 3.04

S. geminata 51 Florida, Columbia Co. 21 2.870 1.828 2.129 2.027 2.189 1.737 0.719 1.086 0.773 0.659 1.153 0.930 1.025 1.199 0.906 1.166 1.247 1.071 1.647 2.048 0.826 1.339 0.869 0.974 0.595 1.294 1.503 6.14

S. geminata 51 Florida, Columbia Co. 22 2.080 1.699 1.946 1.930 2.016 1.560 0.688 1.042 0.744 0.605 1.116 0.850 0.988 1.069 0.833 1.099 1.242 1.006 1.622 2.039 0.850 1.295 0.837 0.895 0.577 1.321 1.415 5.95

S. geminata 51 Florida, Columbia Co. 23 1.870 1.727 1.916 1.862 1.925 1.500 0.663 1.065 0.744 0.618 1.103 0.888 0.966 1.099 0.842 1.104 1.283 1.000 1.620 1.837 0.695 1.152 0.730 0.961 0.535 1.234 1.406 5.76

S. geminata 51 Florida, Columbia Co. 24 2.150 1.688 1.904 1.836 1.909 1.538 0.678 1.029 0.746 0.618 1.121 0.861 0.986 1.112 0.825 1.128 1.266 0.989 1.668 1.884 0.763 1.217 0.809 0.928 0.541 1.223 1.433 5.72

S. geminata 51 Florida, Columbia Co. 25 1.490 1.510 1.657 1.591 1.641 1.335 0.772 0.962 0.715 0.600 1.082 0.811 0.973 1.037 0.826 1.086 1.233 0.989 1.584 1.735 0.698 1.118 0.748 0.844 0.498 1.134 1.273 5.22

S. geminata 51 Florida, Columbia Co. 26 1.460 1.489 1.541 1.476 1.521 1.262 0.701 0.961 0.711 0.605 1.057 0.807 0.961 1.077 0.814 1.102 1.240 1.006 1.581 1.688 0.611 1.038 0.753 0.823 0.483 1.074 1.175 5.07

S. geminata 51 Florida, Columbia Co. 27 1.260 1.457 1.506 1.452 1.443 1.199 0.708 0.947 0.677 0.601 1.006 0.796 0.926 0.967 0.785 1.041 1.157 0.916 1.555 1.579 0.586 0.974 0.687 0.829 0.442 1.049 1.189 4.91

S. geminata 51 Florida, Columbia Co. 28 1.410 1.457 1.511 1.473 1.47 1.194 0.729 0.939 0.709 0.588 0.975 0.815 0.898 0.932 0.759 1.041 1.169 0.920 1.507 1.619 0.593 1.014 0.711 0.774 0.436 1.006 1.114 4.86

S. geminata 51 Florida, Columbia Co. 29 1.160 1.479 1.559 1.495 1.551 1.339 0.622 0.976 0.689 0.595 1.054 0.789 0.982 1.016 0.783 1.076 1.210 0.975 1.588 1.687 0.730 1.059 0.761 0.828 0.463 1.083 1.195 5.08

S. geminata 51 Florida, Columbia Co. 30 0.850 1.312 1.382 1.323 1.352 1.076 0.609 0.921 0.666 0.576 0.958 0.746 0.867 0.933 0.735 0.994 1.149 0.915 1.455 1.572 0.553 0.951 0.682 0.776 0.421 1.054 1.130 4.71

S. geminata 51 Florida, Columbia Co. 31 0.160 0.698 0.557 0.543 0.571 0.498 0.300 0.570 0.423 0.421 0.587 0.417 0.556 0.551 0.414 0.650 0.688 0.523 0.964 0.931 0.316 0.573 0.403 0.491 0.230 0.582 0.553 2.70

S. geminata 51 Florida, Columbia Co. 32 0.120 0.684 0.597 0.552 0.57 0.490 0.306 0.565 0.401 0.418 0.568 0.440 0.542 0.594 0.455 0.631 0.684 0.562 0.951 0.947 0.353 0.554 0.426 0.469 0.236 0.616 0.578 2.72

S. geminata 51 Florida, Columbia Co. 33 0.240 0.715 0.618 0.575 0.6 0.478 0.325 0.586 0.412 0.438 0.581 0.448 0.553 0.550 0.424 0.709 0.730 0.569 0.958 0.971 0.352 0.579 0.433 0.511 0.240 0.685 0.593 2.88

S. geminata 51 Florida, Columbia Co. 34 0.150 0.683 0.586 0.570 0.566 0.495 0.340 0.571 0.396 0.406 0.539 0.402 0.587 0.511 0.409 0.620 0.665 0.526 0.904 0.882 0.292 0.541 0.379 0.512 0.222 0.624 0.553 2.70

S. geminata 51 Florida, Columbia Co. 35 0.110 0.713 0.601 0.550 0.572 0.511 0.327 0.576 0.407 0.418 0.608 0.429 0.556 0.569 0.423 0.630 0.702 0.540 0.936 0.931 0.345 0.569 0.413 0.492 0.241 0.594 0.567 2.73

S. geminata 51 Florida, Columbia Co. 36 0.200 0.716 0.619 0.581 0.6 0.549 0.325 0.605 0.428 0.442 0.619 0.457 0.552 0.591 0.428 0.672 0.745 0.556 0.998 0.973 0.346 0.572 0.432 0.506 0.237 0.645 0.605 2.84

S. geminata 51 Florida, Columbia Co. 37 0.130 0.704 0.618 0.570 0.58 0.501 0.323 0.585 0.359 0.482 0.601 0.442 0.575 0.587 0.444 0.681 0.721 0.563 0.988 0.963 0.335 0.582 0.405 0.527 0.254 0.629 0.589 2.82

S. geminata 51 Florida, Columbia Co. 38 0.120 0.704 0.602 0.565 0.578 0.500 0.306 0.584 0.398 0.420 0.578 0.425 0.553 0.555 0.421 0.628 0.716 0.519 0.819 0.935 0.319 0.570 0.416 0.488 0.223 0.541 0.573 2.67

S. geminata 51 Florida, Columbia Co. 39 0.130 0.699 0.586 0.549 0.571 0.484 0.291 0.565 0.400 0.423 0.543 0.419 0.544 0.525 0.427 0.641 0.687 0.522 0.905 0.906 0.319 0.564 0.395 0.519 0.241 0.589 0.609 2.71

S. geminata 51 Florida, Columbia Co. 40 0.140 0.673 0.555 0.511 0.544 0.442 0.211 0.634 0.409 0.408 0.547 0.433 0.585 0.561 0.414 0.570 0.687 0.515 0.865 0.877 0.341 0.528 0.392 0.435 0.206 0.589 0.510 2.57

S. pythia 52 Argentina, Missiones Prov., S. of Posadas 1 0.420 1.079 1.017 1.014 0.962 0.814 0.445 0.815 0.603 0.568 0.907 0.682 0.816 0.886 0.695 0.982 1.122 0.846 1.487 1.399 0.509 0.857 0.667 0.714 0.370 0.952 1.045 4.14

S. pythia 52 Argentina, Missiones Prov., S. of Posadas 2 0.370 1.011 0.929 0.907 0.874 0.714 0.508 0.777 0.535 0.570 0.852 0.646 0.755 0.841 0.651 0.877 1.023 0.776 1.339 1.345 0.523 0.821 0.589 0.679 0.339 0.923 0.978 3.96

S. pythia 52 Argentina, Missiones Prov., S. of Posadas 3 0.330 1.022 0.981 0.947 0.883 0.785 0.505 0.801 0.614 0.535 0.874 0.654 0.777 0.882 0.629 0.946 1.071 0.821 1.379 1.377 0.517 0.843 0.587 0.662 0.362 0.907 1.006 3.97

S. pythia 52 Argentina, Missiones Prov., S. of Posadas 4 0.350 1.078 0.968 0.973 0.892 0.742 0.527 0.816 0.582 0.568 0.868 0.654 0.777 0.888 0.673 0.896 1.094 0.817 1.407 1.361 0.533 0.841 0.602 0.719 0.365 0.904 1.018 4.06

S. pythia 52 Argentina, Missiones Prov., S. of Posadas 5 0.360 1.045 0.996 0.963 0.901 0.758 0.526 0.801 0.575 0.534 0.862 0.674 0.739 0.870 0.675 0.897 1.080 0.798 1.361 1.347 0.495 0.828 0.602 0.659 0.341 0.857 0.962 3.91

S. pythia 52 Argentina, Missiones Prov., S. of Posadas 6 0.320 1.017 0.945 0.923 0.868 0.736 0.480 0.762 0.536 0.553 0.858 0.650 0.717 0.762 0.613 0.895 0.997 0.782 1.350 1.378 0.529 0.822 0.618 0.680 0.352 0.831 1.014 3.91

S. pythia 52 Argentina, Missiones Prov., S. of Posadas 7 0.270 0.995 0.918 0.896 0.838 0.726 0.520 0.773 0.531 0.549 0.831 0.622 0.740 0.848 0.617 0.880 1.039 0.771 1.310 1.286 0.412 0.821 0.587 0.668 0.343 0.813 0.996 3.76

S. pythia 52 Argentina, Missiones Prov., S. of Posadas 8 0.310 1.012 0.937 0.914 0.857 0.704 0.491 0.792 0.566 0.545 0.873 0.656 0.763 0.874 0.665 0.933 1.057 0.794 1.374 1.345 0.567 0.822 0.606 0.671 0.330 0.813 0.929 3.84

S. pythia 52 Argentina, Missiones Prov., S. of Posadas 9 0.300 0.954 0.872 0.860 0.793 0.699 0.424 0.732 0.528 0.548 0.842 0.610 0.751 0.813 0.583 0.902 1.031 0.750 1.337 1.250 0.440 0.800 0.558 0.666 0.341 0.861 0.982 3.73

S. pythia 52 Argentina, Missiones Prov., S. of Posadas 10 0.270 0.918 0.819 0.786 0.755 0.654 0.375 0.719 0.501 0.499 0.769 0.561 0.715 0.778 0.552 0.838 0.986 0.705 1.223 1.212 0.352 0.749 0.533 0.628 0.306 0.787 0.827 3.54

S. pythia 52 Argentina, Missiones Prov., S. of Posadas 11 0.250 0.963 0.877 0.844 0.816 0.697 0.473 0.750 0.537 0.566 0.820 0.631 0.758 0.850 0.600 0.939 0.988 0.770 1.380 1.263 0.447 0.790 0.553 0.664 0.333 0.731 0.868 3.62

S. pythia 52 Argentina, Missiones Prov., S. of Posadas 12 0.220 0.962 0.853 0.830 0.769 0.676 0.479 0.757 0.525 0.507 0.800 0.597 0.710 0.784 0.590 0.835 0.940 0.735 1.274 1.257 0.458 0.764 0.551 0.650 0.330 0.778 0.851 3.65

S. pythia 52 Argentina, Missiones Prov., S. of Posadas 13 0.180 0.939 0.827 0.778 0.769 0.628 0.406 0.716 0.524 0.537 0.767 0.611 0.728 0.739 0.586 0.855 0.909 0.734 1.254 1.244 0.467 0.791 0.528 0.594 0.297 0.767 0.805 3.54

S. pythia 52 Argentina, Missiones Prov., S. of Posadas 14 0.180 0.759 0.655 0.639 0.624 0.524 0.313 0.603 0.462 0.421 0.643 0.487 0.590 0.610 0.447 0.703 0.781 0.578 1.047 1.026 0.371 0.639 0.425 0.581 0.253 0.657 0.708 3.02

S. pythia 52 Argentina, Missiones Prov., S. of Posadas 15 0.170 0.824 0.714 0.687 0.661 0.571 0.358 0.653 0.468 0.462 0.684 0.523 0.615 0.671 0.506 0.725 0.835 0.629 1.098 1.094 0.368 0.666 0.486 0.573 0.276 0.687 0.703 3.18

S. pythia 52 Argentina, Missiones Prov., S. of Posadas 16 0.240 0.803 0.665 0.626 0.621 0.539 0.389 0.639 0.437 0.479 0.654 0.498 0.603 0.660 0.480 0.719 0.795 0.608 1.075 1.050 0.369 0.621 0.452 0.561 0.247 0.605 0.698 3.02

S. pythia 52 Argentina, Missiones Prov., S. of Posadas 17 0.150 0.820 0.689 0.677 0.654 0.547 0.400 0.653 0.446 0.479 0.667 0.511 0.611 0.659 0.478 0.713 0.840 0.614 1.103 1.089 0.359 0.669 0.487 0.657 0.273 0.643 0.709 3.21

S. pythia 52 Argentina, Missiones Prov., S. of Posadas 18 0.220 0.799 0.684 0.673 0.641 0.529 0.408 0.632 0.467 0.480 0.701 0.501 0.614 0.682 0.472 0.743 0.843 0.613 1.127 1.065 0.393 0.667 0.447 0.571 0.254 0.614 0.707 3.05

S. pythia 52 Argentina, Missiones Prov., S. of Posadas 19 0.170 0.780 0.643 0.621 0.605 0.500 0.348 0.605 0.416 0.440 0.622 0.486 0.564 0.602 0.455 0.680 0.779 0.571 1.013 1.023 0.368 0.636 0.440 0.538 0.249 0.555 0.623 2.90

S. pythia 52 Argentina, Missiones Prov., S. of Posadas 20 0.220 0.769 0.653 0.627 0.618 0.503 0.332 0.590 0.411 0.472 0.635 0.466 0.586 0.627 0.462 0.700 0.760 0.605 1.066 1.007 0.363 0.625 0.440 0.524 0.255 0.669 0.647 2.97

S. pythia 52 Argentina, Missiones Prov., S. of Posadas 21 0.170 0.638 0.564 0.552 0.521 0.443 0.293 0.515 0.358 0.424 0.531 0.436 0.521 0.533 0.397 0.609 0.682 0.500 0.927 0.925 0.332 0.567 0.400 0.484 0.215 0.560 0.553 2.61

S. pythia 52 Argentina, Missiones Prov., S. of Posadas 22 0.160 0.770 0.672 0.628 0.626 0.539 0.382 0.617 0.443 0.454 0.661 0.455 0.636 0.637 0.477 0.723 0.805 0.588 1.034 1.035 0.376 0.641 0.448 0.603 0.263 0.621 0.643 3.03

S. pythia 52 Argentina, Missiones Prov., S. of Posadas 23 0.110 0.665 0.577 0.555 0.528 0.440 0.335 0.533 0.360 0.431 0.535 0.422 0.518 0.532 0.401 0.584 0.680 0.500 0.906 0.874 0.330 0.547 0.361 0.503 0.206 0.540 0.589 2.58

S. pythia 52 Argentina, Missiones Prov., S. of Posadas 24 0.140 0.756 0.637 0.597 0.596 0.505 0.370 0.585 0.381 0.469 0.621 0.480 0.553 0.626 0.448 0.662 0.767 0.580 1.025 1.018 0.336 0.601 0.482 0.469 0.243 0.599 0.670 2.84

S. pythia 52 Argentina, Missiones Prov., S. of Posadas 25 0.170 0.699 0.594 0.572 0.554 0.474 0.328 0.540 0.385 0.414 0.566 0.424 0.538 0.562 0.432 0.634 0.719 0.558 0.929 0.944 0.334 0.585 0.393 0.490 0.242 0.585 0.559 2.72

S. pythia 52 Argentina, Missiones Prov., S. of Posadas 26 0.120 0.687 0.577 0.549 0.533 0.456 0.313 0.538 0.374 0.398 0.547 0.450 0.509 0.536 0.408 0.644 0.678 0.509 0.905 0.914 0.319 0.558 0.387 0.489 0.221 0.577 0.571 2.67

S. pythia 52 Argentina, Missiones Prov., S. of Posadas 27 0.110 0.738 0.650 0.628 0.61 0.529 0.384 0.619 0.423 0.460 0.649 0.503 0.564 0.621 0.471 0.686 0.780 0.595 1.011 0.973 0.357 0.628 0.408 0.568 0.254 0.670 0.643 2.95

S. pythia 52 Argentina, Missiones Prov., S. of Posadas 28 0.150 0.703 0.604 0.572 0.559 0.495 0.353 0.566 0.405 0.432 0.588 0.475 0.537 0.601 0.435 0.631 0.734 0.573 0.962 0.954 0.346 0.600 0.403 0.547 0.246 0.621 0.616 2.83

S. pythia 52 Argentina, Missiones Prov., S. of Posadas 29 0.120 0.667 0.560 0.533 0.518 0.431 0.292 0.537 0.360 0.404 0.596 0.416 0.498 0.522 0.397 0.590 0.659 0.507 0.875 0.893 0.321 0.564 0.376 0.528 0.216 0.507 0.608 2.60

S. pythia 52 Argentina, Missiones Prov., S. of Posadas 30 0.100 0.682 0.555 0.533 0.515 0.456 0.322 0.507 0.338 0.400 0.503 0.375 0.511 0.507 0.355 0.596 0.602 0.493 0.838 0.866 0.310 0.577 0.360 0.502 0.221 0.495 0.545 2.54

S. pythia 52 Argentina, Missiones Prov., S. of Posadas 31 0.110 0.666 0.555 0.533 0.517 0.457 0.320 0.512 0.372 0.403 0.536 0.410 0.514 0.527 0.396 0.600 0.663 0.505 0.898 0.900 0.348 0.539 0.425 0.438 0.221 0.522 0.544 2.53

S. pythia 52 Argentina, Missiones Prov., S. of Posadas 32 0.120 0.700 0.590 0.570 0.569 0.492 0.361 0.553 0.367 0.441 0.575 0.455 0.532 0.567 0.418 0.618 0.659 0.523 0.945 0.909 0.298 0.564 0.404 0.516 0.233 0.561 0.590 2.69

S. pythia 52 Argentina, Missiones Prov., S. of Posadas 33 0.110 0.683 0.587 0.557 0.549 0.468 0.329 0.573 0.389 0.422 0.591 0.455 0.541 0.570 0.416 0.646 0.720 0.558 0.952 0.925 0.313 0.577 0.419 0.528 0.235 0.589 0.605 2.73

S. pythia 52 Argentina, Missiones Prov., S. of Posadas 34 0.100 0.712 0.573 0.556 0.545 0.456 0.341 0.538 0.336 0.445 0.578 0.442 0.518 0.556 0.409 0.624 0.718 0.534 0.934 0.915 0.347 0.547 0.397 0.548 0.223 0.550 0.599 2.72

S. pythia 52 Argentina, Missiones Prov., S. of Posadas 35 0.100 0.687 0.572 0.544 0.542 0.478 0.355 0.547 0.384 0.429 0.525 0.407 0.544 0.552 0.411 0.605 0.710 0.510 0.914 0.907 0.325 0.573 0.407 0.490 0.242 0.567 0.584 2.65

S. pythia 52 Argentina, Missiones Prov., S. of Posadas 36 0.090 0.682 0.578 0.550 0.529 0.467 0.344 0.543 0.353 0.412 0.559 0.402 0.528 0.559 0.415 0.595 0.698 0.507 0.912 0.929 0.308 0.579 0.393 0.492 0.232 0.546 0.583 2.65

S. pythia 52 Argentina, Missiones Prov., S. of Posadas 37 0.100 0.698 0.583 0.550 0.555 0.473 0.356 0.527 0.352 0.440 0.575 0.417 0.546 0.558 0.425 0.603 0.705 0.497 0.922 0.940 0.376 0.588 0.402 0.489 0.220 0.537 0.560 2.66

S. pythia 52 Argentina, Missiones Prov., S. of Posadas 38 0.110 0.704 0.612 0.579 0.563 0.493 0.356 0.552 0.390 0.428 0.592 0.417 0.541 0.573 0.400 0.646 0.722 0.514 0.933 0.972 0.358 0.620 0.400 0.549 0.247 0.627 0.616 2.85

S. pythia 52 Argentina, Missiones Prov., S. of Posadas 39 0.090 0.650 0.551 0.529 0.52 0.462 0.320 0.528 0.359 0.416 0.529 0.403 0.507 0.527 0.391 0.600 0.653 0.504 0.883 0.901 0.303 0.553 0.389 0.499 0.216 0.524 0.541 2.57

S. pythia 52 Argentina, Missiones Prov., S. of Posadas 40 0.090 0.650 0.569 0.547 0.523 0.478 0.339 0.533 0.374 0.407 0.551 0.406 0.518 0.554 0.418 0.594 0.697 0.512 0.911 0.868 0.313 0.531 0.393 0.511 0.220 0.533 0.588 2.56

S. geminata 53 Texas, Lampasas Co. 1 0.480 1.275 1.207 1.174 1.137 0.930 0.613 0.947 0.709 0.585 0.997 0.820 0.890 0.986 0.735 1.101 1.188 0.941 1.588 1.537 0.514 0.970 0.661 0.743 0.419 0.932 1.012 4.49

S. geminata 53 Texas, Lampasas Co. 2 0.220 0.963 0.850 0.794 0.802 0.655 0.447 0.793 0.616 0.551 0.794 0.681 0.741 0.823 0.611 0.947 1.006 0.796 1.367 1.252 0.447 0.786 0.555 0.678 0.314 0.793 0.793 3.69

S. geminata 53 Texas, Lampasas Co. 3 0.190 0.911 0.805 0.744 0.741 0.623 0.418 0.726 0.576 0.538 0.767 0.561 0.759 0.757 0.584 0.855 0.959 0.747 1.318 1.201 0.414 0.776 0.510 0.621 0.297 0.760 0.754 3.49

S. geminata 53 Texas, Lampasas Co. 4 0.180 0.933 0.810 0.760 0.758 0.654 0.439 0.758 0.602 0.522 0.837 0.555 0.753 0.743 0.613 0.890 0.955 0.739 1.336 1.240 0.442 0.733 0.550 0.637 0.285 0.738 0.765 3.55

S. geminata 53 Texas, Lampasas Co. 5 0.620 1.396 1.345 1.334 1.31 1.046 0.593 0.978 0.732 0.613 1.057 0.828 0.930 1.072 0.814 1.145 1.352 0.930 1.693 1.634 0.582 1.017 0.740 0.826 0.460 0.984 1.096 4.84

S. geminata 53 Texas, Lampasas Co. 6 0.160 0.866 0.739 0.677 0.69 0.621 0.379 0.716 0.528 0.502 0.706 0.595 0.665 0.680 0.545 0.828 0.886 0.700 1.240 1.138 0.391 0.698 0.497 0.569 0.281 0.715 0.726 3.29

S. geminata 53 Texas, Lampasas Co. 7 0.170 0.912 0.793 0.743 0.728 0.598 0.404 0.733 0.565 0.533 0.731 0.626 0.666 0.735 0.578 0.848 0.945 0.749 1.310 1.183 0.400 0.731 0.510 0.617 0.300 0.699 0.749 3.41

S. geminata 53 Texas, Lampasas Co. 8 0.230 1.012 0.930 0.867 0.864 0.761 0.478 0.815 0.582 0.581 0.858 0.656 0.769 0.838 0.648 0.968 1.039 0.818 1.445 1.325 0.459 0.829 0.577 0.693 0.339 0.827 0.844 3.86

S. geminata 53 Texas, Lampasas Co. 9 0.250 1.028 0.939 0.872 0.86 0.771 0.473 0.795 0.597 0.566 0.834 0.732 0.770 0.792 0.675 0.964 1.074 0.810 1.455 1.336 0.441 0.833 0.570 0.679 0.338 0.900 0.844 3.94

S. geminata 53 Texas, Lampasas Co. 10 0.210 0.921 0.813 0.756 0.769 0.662 0.422 0.757 0.571 0.542 0.788 0.623 0.713 0.754 0.606 0.869 0.972 0.713 1.371 1.190 0.442 0.728 0.522 0.618 0.293 0.804 0.773 3.53

S. geminata 53 Texas, Lampasas Co. 11 0.200 0.951 0.839 0.777 0.791 0.676 0.356 0.780 0.562 0.542 0.786 0.643 0.762 0.777 0.635 0.893 0.990 0.771 1.353 1.260 0.472 0.748 0.522 0.640 0.315 0.763 0.773 3.61

S. geminata 53 Texas, Lampasas Co. 12 0.190 0.901 0.799 0.732 0.733 0.615 0.397 0.740 0.564 0.533 0.814 0.595 0.710 0.779 0.586 0.860 0.956 0.738 1.299 1.201 0.438 0.722 0.523 0.633 0.297 0.743 0.765 3.48

S. geminata 53 Texas, Lampasas Co. 13 1.390 1.654 1.879 1.850 1.87 1.531 0.807 1.092 0.787 0.724 1.249 0.966 1.056 1.253 0.927 1.337 1.432 1.127 1.853 1.944 0.660 1.283 0.852 0.977 0.579 1.230 1.464 5.80

S. geminata 53 Texas, Lampasas Co. 14 0.200 0.951 0.833 0.771 0.792 0.693 0.407 0.796 0.558 0.537 0.805 0.620 0.767 0.811 0.599 0.909 0.988 0.760 1.348 1.232 0.453 0.742 0.517 0.715 0.310 0.830 0.848 3.73

S. geminata 53 Texas, Lampasas Co. 15 0.220 0.956 0.844 0.788 0.795 0.710 0.427 0.794 0.583 0.551 0.774 0.657 0.750 0.759 0.628 0.920 1.000 0.782 1.392 1.214 0.405 0.763 0.513 0.717 0.310 0.707 0.839 3.59

S. geminata 53 Texas, Lampasas Co. 16 0.160 0.901 0.797 0.735 0.738 0.623 0.399 0.720 0.543 0.543 0.749 0.610 0.707 0.733 0.585 0.893 0.907 0.729 1.312 1.175 0.438 0.714 0.520 0.618 0.291 0.760 0.754 3.45

S. geminata 53 Texas, Lampasas Co. 17 0.260 0.978 0.883 0.827 0.826 0.693 0.393 0.806 0.590 0.562 0.833 0.680 0.744 0.827 0.642 0.929 1.007 0.812 1.377 1.281 0.484 0.782 0.567 0.667 0.330 0.788 0.816 3.71

S. geminata 53 Texas, Lampasas Co. 18 0.230 0.924 0.795 0.734 0.746 0.672 0.368 0.773 0.544 0.565 0.732 0.621 0.707 0.736 0.559 0.889 0.847 0.746 1.308 1.205 0.438 0.733 0.524 0.596 0.287 0.764 0.817 3.49

S. geminata 53 Texas, Lampasas Co. 19 0.210 0.916 0.816 0.743 0.761 0.637 0.428 0.763 0.557 0.526 0.727 0.608 0.715 0.760 0.569 0.913 0.961 0.763 1.301 1.198 0.411 0.715 0.512 0.626 0.301 0.744 0.721 3.48

S. geminata 53 Texas, Lampasas Co. 20 0.170 0.922 0.805 0.743 0.753 0.638 0.357 0.721 0.560 0.535 0.742 0.618 0.729 0.746 0.609 0.884 0.971 0.692 1.409 1.192 0.403 0.716 0.517 0.646 0.309 0.726 0.743 3.49

S. geminata 53 Texas, Lampasas Co. 21 1.900 1.693 2.091 2.079 2.082 1.676 0.884 1.109 0.824 0.695 1.235 0.993 1.089 1.227 0.962 1.315 1.436 1.096 1.955 2.089 0.795 1.366 0.925 1.027 0.614 1.255 1.495 6.06

S. geminata 53 Texas, Lampasas Co. 22 1.750 1.711 1.961 1.956 1.925 1.514 0.690 1.097 0.835 0.694 1.210 0.970 1.106 1.188 0.949 1.317 1.380 1.113 1.939 2.121 0.794 1.391 0.891 1.042 0.594 1.258 1.482 6.13

S. geminata 53 Texas, Lampasas Co. 23 1.960 1.861 2.090 2.073 2.03 1.643 0.857 1.114 0.809 0.671 1.217 0.973 1.102 1.216 0.952 1.292 1.436 1.084 1.884 2.088 0.860 1.414 0.878 1.080 0.616 1.218 1.408 6.25

S. geminata 53 Texas, Lampasas Co. 24 2.020 1.889 2.085 2.019 2.086 1.565 0.767 1.165 0.875 0.696 1.297 0.967 1.126 1.241 1.011 1.352 1.518 1.176 1.998 2.071 0.813 1.414 0.865 1.023 0.623

S. geminata 53 Texas, Lampasas Co. 25 1.380 1.654 1.899 1.872 1.851 1.441 0.730 1.112 0.803 0.697 1.161 0.940 1.053 1.166 0.905 1.306 1.414 1.096 1.878 1.992 0.756 1.290 0.844 0.920 0.570 1.218 1.419 5.78

S. geminata 53 Texas, Lampasas Co. 26 1.880 1.767 2.073 2.057 2.02 1.663 0.842 1.154 0.848 0.672 1.164 0.975 1.099 1.200 0.939 1.331 1.437 1.112 1.969 2.042 0.749 1.329 0.881 0.951 0.605 1.264 1.509 6.02

S. geminata 53 Texas, Lampasas Co. 27 1.400 1.659 1.872 1.872 1.79 1.464 0.788 1.116 0.805 0.652 1.159 0.913 1.012 1.148 0.904 1.214 1.270 1.052 1.810 1.955 0.715 1.266 0.846 0.927 0.559 1.256 1.382 5.80

S. geminata 53 Texas, Lampasas Co. 28 1.330 1.766 2.029 2.000 2.009 1.631 0.852 1.125 0.832 0.701 1.203 0.935 1.087 1.193 0.934 1.328 1.397 1.092 1.967 2.008 0.827 1.322 0.863 0.987 0.582 1.171 1.458 5.93

S. geminata 53 Texas, Lampasas Co. 29 1.280 1.534 1.769 1.725 1.698 1.437 0.820 1.087 0.778 0.678 1.104 0.916 1.070 1.157 0.911 1.281 1.384 1.048 1.884 1.922 0.732 1.239 0.847 0.944 0.548 1.207 1.369 5.61

S. geminata 53 Texas, Lampasas Co. 30 1.260 1.603 1.760 1.721 1.684 1.369 0.700 1.045 0.748 0.661 1.023 0.921 0.995 1.123 0.901 1.202 1.339 1.056 1.789 1.826 0.682 1.200 0.746 0.895 0.525 1.139 1.254 5.46

S. geminata 53 Texas, Lampasas Co. 31 0.100 0.843 0.688 0.618 0.656 0.587 0.432 0.594 0.470 0.484 0.665 0.442 0.667 0.680 0.510 0.785 0.760 0.645 1.145 1.046 0.388 0.648 0.450 0.567 0.245 0.655 0.640 3.11

S. geminata 53 Texas, Lampasas Co. 32 0.130 0.849 0.732 0.682 0.689 0.592 0.396 0.690 0.505 0.505 0.636 0.542 0.652 0.660 0.523 0.804 0.804 0.655 1.183 1.106 0.330 0.678 0.484 0.642 0.276 0.666 0.677 3.26

S. geminata 53 Texas, Lampasas Co. 33 0.100 0.886 0.787 0.736 0.747 0.633 0.417 0.723 0.548 0.514 0.725 0.570 0.720 0.689 0.567 0.863 0.913 0.723 1.257 1.179 0.453 0.716 0.498 0.625 0.293 0.672 0.714 3.36

S. geminata 53 Texas, Lampasas Co. 34 0.120 0.883 0.743 0.687 0.686 0.593 0.412 0.695 0.516 0.500 0.744 0.518 0.687 0.694 0.546 0.824 0.893 0.690 1.257 1.090 0.397 0.675 0.491 0.597 0.274 0.688 0.721 3.26

S. geminata 53 Texas, Lampasas Co. 35 0.100 0.838 0.705 0.649 0.675 0.581 0.368 0.691 0.494 0.506 0.674 0.514 0.644 0.627 0.494 0.798 0.760 0.641 1.152 1.095 0.363 0.644 0.469 0.568 0.258 0.651 0.651 3.15

S. geminata 53 Texas, Lampasas Co. 36 0.100 0.869 0.772 0.698 0.705 0.612 0.389 0.710 0.533 0.504 0.653 0.510 0.632 0.649 0.494 0.787 0.777 0.643 1.156 1.179 0.366 0.698 0.512 0.592 0.286 0.687 0.705 3.33

S. geminata 53 Texas, Lampasas Co. 37 0.120 0.878 0.783 0.721 0.724 0.631 0.407 0.735 0.544 0.522 0.718 0.553 0.680 0.670 0.560 0.833 0.874 0.705 1.247 1.174 0.386 0.710 0.536 0.615 0.296 0.698 0.760 3.36

S. geminata 53 Texas, Lampasas Co. 38 0.130 0.889 0.755 0.687 0.707 0.631 0.431 0.729 0.531 0.509 0.753 0.581 0.687 0.749 0.574 0.866 0.923 0.721 1.312 1.126 0.408 0.722 0.499 0.590 0.295 0.676 0.698 3.28

S. geminata 53 Texas, Lampasas Co. 39 0.100 0.871 0.748 0.706 0.711 0.615 0.357 0.712 0.530 0.514 0.728 0.572 0.690 0.704 0.556 0.839 0.873 0.687 1.274 1.135 0.391 0.698 0.498 0.612 0.271 0.738 0.710 3.36

S. geminata 53 Texas, Lampasas Co. 40 0.100 0.967 0.833 0.771 0.767 0.706 0.430 0.759 0.557 0.538 0.749 0.593 0.754 0.750 0.597 0.883 0.955 0.754 1.331 1.218 0.436 0.763 0.530 0.628 0.314 0.805 0.773 3.62

S. geminata 54 Texas, Lampasas Co. 1 0.490 1.236 1.200 1.134 1.174 1.063 0.568 0.928 0.529 0.756 1.007 0.776 0.905 0.972 0.764 1.152 1.174 0.926 1.685 1.572 0.614 0.995 0.646 0.771 0.409 1.012 0.981 4.59

S. geminata 54 Texas, Lampasas Co. 2 0.570 1.286 1.208 1.162 1.191 1.070 0.645 0.936 0.596 0.673 1.015 0.719 0.918 1.004 0.753 1.071 1.223 0.892 1.587 1.548 0.502 0.963 0.665 0.764 0.412 0.980 1.020 4.58

S. geminata 54 Texas, Lampasas Co. 3 0.500 1.313 1.325 1.236 1.238 1.146 0.606 0.906 0.695 0.600 0.999 0.776 0.912 0.985 0.774 1.062 1.206 0.922 1.613 1.592 0.570 0.993 0.686 0.748 0.430 0.988 1.076 4.64

S. geminata 54 Texas, Lampasas Co. 4 0.370 1.158 1.104 1.015 1.048 0.937 0.530 0.901 0.659 0.578 0.933 0.742 0.844 0.933 0.695 1.039 1.116 0.878 1.518 1.448 0.526 0.880 0.607 0.741 0.371 0.951 0.909 4.30

S. geminata 54 Texas, Lampasas Co. 5 0.550 1.272 1.248 1.182 1.221 1.073 0.578 0.899 0.652 0.593 0.983 0.758 0.892 0.952 0.765 1.056 1.209 0.876 1.606 1.535 0.574 0.911 0.668 0.723 0.404 0.895 1.011 4.43

S. geminata 54 Texas, Lampasas Co. 6 0.330 1.117 1.075 1.029 1.042 0.902 0.520 0.868 0.625 0.600 0.900 0.765 0.825 0.902 0.701 0.991 1.034 0.823 1.477 1.397 0.468 0.838 0.614 0.670 0.345 0.899 0.911 4.08

S. geminata 54 Texas, Lampasas Co. 7 0.340 1.099 1.045 0.979 0.985 0.943 0.646 0.879 0.639 0.561 0.917 0.687 0.873 0.892 0.670 1.070 1.084 0.875 1.519 1.423 0.479 0.876 0.617 0.714 0.377 0.887 0.940 4.12

S. geminata 54 Texas, Lampasas Co. 8 0.270 1.073 0.950 0.878 0.889 0.836 0.439 0.842 0.600 0.519 0.871 0.684 0.771 0.836 0.669 0.933 1.045 0.818 1.412 1.323 0.461 0.812 0.559 0.703 0.329 0.875 0.874 3.97

S. geminata 54 Texas, Lampasas Co. 9 0.210 0.979 0.872 0.788 0.807 0.764 0.376 0.799 0.593 0.561 0.800 0.603 0.742 0.775 0.588 0.908 0.996 0.721 1.333 1.265 0.427 0.772 0.571 0.749 0.292 0.783 0.782 3.78

S. geminata 54 Texas, Lampasas Co. 10 0.320 1.051 1.033 0.931 0.935 0.842 0.510 0.852 0.633 0.579 0.860 0.682 0.851 0.840 0.671 1.014 1.063 0.841 1.460 1.351 0.509 0.857 0.608 0.656 0.355 0.891 0.858 3.95

S. geminata 54 Texas, Lampasas Co. 11 0.210 0.847 0.755 0.695 0.705 0.677 0.385 0.718 0.549 0.513 0.730 0.584 0.669 0.693 0.555 0.816 0.891 0.688 1.232 1.102 0.402 0.690 0.472 0.563 0.270 0.728 0.656 3.24

S. geminata 54 Texas, Lampasas Co. 12 0.200 0.832 0.723 0.669 0.686 0.639 0.370 0.708 0.551 0.494 0.707 0.595 0.597 0.665 0.526 0.793 0.859 0.663 1.189 1.099 0.357 0.655 0.471 0.625 0.266 0.718 0.659 3.27

S. geminata 54 Texas, Lampasas Co. 13 0.250 0.849 0.754 0.669 0.704 0.586 0.349 0.727 0.536 0.513 0.841 0.557 0.705 0.695 0.589 0.877 0.861 0.717 1.289 1.144 0.409 0.682 0.507 0.629 0.281 0.806 0.741 3.43

S. geminata 54 Texas, Lampasas Co. 14 0.230 0.896 0.764 0.675 0.707 0.663 0.368 0.719 0.523 0.529 0.734 0.604 0.662 0.705 0.580 0.822 0.914 0.694 1.248 1.139 0.389 0.683 0.489 0.571 0.257 0.782 0.696 3.39

S. geminata 54 Texas, Lampasas Co. 15 0.130 0.816 0.724 0.640 0.657 0.608 0.376 0.645 0.508 0.474 0.675 0.530 0.650 0.682 0.497 0.786 0.842 0.656 1.147 1.052 0.391 0.648 0.470 0.531 0.257 0.717 0.663 3.12

S. geminata 54 Texas, Lampasas Co. 16 0.140 0.842 0.722 0.657 0.683 0.621 0.397 0.686 0.494 0.489 0.725 0.516 0.664 0.677 0.520 0.793 0.872 0.666 1.142 1.082 0.346 0.688 0.446 0.582 0.263 0.724 0.675 3.23

S. geminata 54 Texas, Lampasas Co. 17 0.160 0.866 0.776 0.693 0.731 0.699 0.421 0.734 0.520 0.511 0.728 0.565 0.687 0.717 0.573 0.827 0.893 0.685 1.238 1.126 0.388 0.709 0.508 0.563 0.279 0.896 0.711 3.45

S. geminata 54 Texas, Lampasas Co. 18 0.140 0.842 0.734 0.675 0.689 0.633 0.427 0.719 0.516 0.493 0.710 0.561 0.684 0.700 0.539 0.831 0.904 0.686 1.220 1.152 0.406 0.677 0.509 0.578 0.270 0.769 0.689 3.34

S. geminata 54 Texas, Lampasas Co. 19 0.160 0.866 0.758 0.699 0.715 0.651 0.393 0.711 0.532 0.517 0.722 0.575 0.610 0.712 0.575 0.850 0.926 0.714 1.231 1.146 0.406 0.695 0.505 0.603 0.294 0.728 0.728 3.34

S. geminata 54 Texas, Lampasas Co. 20 0.170 0.884 0.794 0.758 0.762 0.722 0.401 0.736 0.509 0.516 0.777 0.578 0.708 0.742 0.553 0.878 0.929 0.692 1.269 1.170 0.406 0.714 0.518 0.614 0.282 0.768 0.756 3.44

S. geminata 54 Texas, Lampasas Co. 21 0.220 0.903 0.794 0.728 0.75 0.687 0.453 0.710 0.535 0.548 0.746 0.556 0.714 0.716 0.585 0.824 0.914 0.710 1.253 1.214 0.442 0.728 0.509 0.606 0.296 0.854 0.735 3.58

S. geminata 54 Texas, Lampasas Co. 22 0.180 0.854 0.740 0.675 0.709 0.663 0.391 0.699 0.560 0.503 0.719 0.525 0.696 0.689 0.544 0.793 0.870 0.681 1.159 1.131 0.374 0.677 0.503 0.548 0.270 0.753 0.681 3.29

S. geminata 54 Texas, Lampasas Co. 23 0.170 0.848 0.776 0.716 0.711 0.627 0.363 0.733 0.537 0.510 0.745 0.609 0.723 0.713 0.611 0.856 0.891 0.713 1.272 1.140 0.388 0.710 0.491 0.594 0.286 0.747 0.688 3.33

S. geminata 54 Texas, Lampasas Co. 24 0.210 0.892 0.788 0.740 0.771 0.687 0.414 0.720 0.559 0.523 0.788 0.639 0.710 0.779 0.572 0.879 0.957 0.734 1.303 1.234 0.410 0.750 0.517 0.563 0.282 0.777 0.874 3.47

S. geminata 54 Texas, Lampasas Co. 25 0.200 0.825 0.722 0.681 0.675 0.597 0.384 0.727 0.521 0.503 0.711 0.582 0.670 0.675 0.550 0.802 0.883 0.697 1.185 1.103 0.401 0.672 0.470 0.565 0.275 0.693 0.675 3.19

S. geminata 54 Texas, Lampasas Co. 26 0.180 0.885 0.788 0.722 0.748 0.687 0.383 0.744 0.540 0.509 0.733 0.573 0.705 0.710 0.550 0.853 0.900 0.711 1.248 1.147 0.388 0.705 0.489 0.638 0.279 0.716 0.722 3.39

S. geminata 54 Texas, Lampasas Co. 27 0.180 0.857 0.759 0.681 0.697 0.616 0.392 0.693 0.502 0.505 0.713 0.559 0.649 0.705 0.542 0.802 0.872 0.672 1.177 1.087 0.371 0.655 0.510 0.579 0.262 0.718 0.705 3.24

S. geminata 54 Texas, Lampasas Co. 28 0.130 0.872 0.740 0.704 0.698 0.645 0.410 0.715 0.535 0.494 0.725 0.591 0.710 0.700 0.558 0.869 0.892 0.693 1.243 1.137 0.390 0.685 0.489 0.560 0.290 0.705 0.718 3.27

S. geminata 54 Texas, Lampasas Co. 29 0.200 0.890 0.783 0.752 0.765 0.693 0.446 0.761 0.540 0.512 0.772 0.618 0.708 0.758 0.564 0.866 0.879 0.732 1.263 1.188 0.431 0.723 0.511 0.588 0.277 0.711 0.710 3.38

S. geminata 54 Texas, Lampasas Co. 30 0.180 0.804 0.706 0.633 0.663 0.627 0.343 0.655 0.479 0.472 0.657 0.542 0.640 0.658 0.506 0.785 0.857 0.628 1.140 1.054 0.359 0.652 0.465 0.525 0.263 0.675 0.658 3.06

S. geminata 54 Texas, Lampasas Co. 31 0.180 0.836 0.746 0.657 0.693 0.669 0.367 0.688 0.500 0.519 0.715 0.565 0.682 0.693 0.513 0.804 0.845 0.673 1.190 1.096 0.377 0.683 0.433 0.655 0.259 0.712 0.663 3.30

S. geminata 54 Texas, Lampasas Co. 32 0.160 0.836 0.728 0.651 0.676 0.627 0.368 0.723 0.533 0.490 0.700 0.546 0.681 0.674 0.527 0.832 0.864 0.678 1.205 1.092 0.362 0.674 0.467 0.633 0.239 0.672 0.666 3.23

S. geminata 54 Texas, Lampasas Co. 33 0.170 0.807 0.685 0.616 0.666 0.604 0.394 0.688 0.504 0.497 0.697 0.545 0.677 0.661 0.528 0.784 0.834 0.670 1.152 1.054 0.346 0.636 0.481 0.551 0.264 0.747 0.678 3.16

S. geminata 54 Texas, Lampasas Co. 34 0.160 0.896 0.710 0.675 0.661 0.557 0.409 0.697 0.507 0.492 0.708 0.548 0.682 0.676 0.531 0.801 0.858 0.684 1.182 1.087 0.358 0.666 0.453 0.558 0.258 0.784 0.657 3.33

S. geminata 54 Texas, Lampasas Co. 35 0.130 0.844 0.724 0.663 0.69 0.628 0.372 0.670 0.515 0.479 0.709 0.531 0.656 0.658 0.499 0.780 0.830 0.651 1.200 1.068 0.383 0.674 0.447 0.581 0.251 0.651 0.651 3.14

S. geminata 54 Texas, Lampasas Co. 36 0.842 0.776 0.705 0.711 0.645 0.379 0.698 0.563 0.492 0.718 0.598 0.633 0.714 0.538 0.818 0.910 0.693 1.235 1.130 0.364 0.694 0.459 0.589 0.282 0.751 0.678 3.31

S. geminata 54 Texas, Lampasas Co. 37 0.160 0.878 0.729 0.651 0.691 0.635 0.367 0.720 0.522 0.514 0.724 0.578 0.696 0.703 0.550 0.835 0.895 0.617 1.268 1.095 0.359 0.665 0.478 0.566 0.265 0.733 0.678 3.27

S. geminata 54 Texas, Lampasas Co. 38 0.120 0.814 0.724 0.645 0.666 0.618 0.365 0.662 0.494 0.504 0.702 0.531 0.651 0.669 0.509 0.795 0.865 0.643 1.170 1.059 0.374 0.659 0.455 0.540 0.259 0.681 0.657 3.09

S. geminata 54 Texas, Lampasas Co. 39 0.130 0.794 0.716 0.603 0.661 0.627 0.351 0.650 0.505 0.487 0.669 0.555 0.642 0.644 0.517 0.781 0.836 0.639 1.189 1.081 0.342 0.663 0.463 0.561 0.271 0.744 0.676 3.18

S. geminata 54 Texas, Lampasas Co. 40 0.140 0.827 0.730 0.653 0.692 0.597 0.381 0.717 0.501 0.495 0.735 0.567 0.655 0.689 0.599 0.770 0.876 0.679 1.183 1.084 0.383 0.682 0.459 0.556 0.273 0.804 0.678 3.27

S. geminata 55 Texas, Lampasas Co. 1 1.550 1.822 2.040 2.034 2.009 1.543 0.740 1.144 0.850 0.631 1.203 0.973 1.071 1.236 0.909 1.314 1.399 1.090 1.981 2.041 0.865 1.343 0.894 0.955 0.552 1.212 1.378 6.03

S. geminata 55 Texas, Lampasas Co. 2 1.400 1.759 1.925 1.905 1.877 1.606 0.777 1.110 0.871 0.677 1.202 0.924 1.066 1.203 0.931 1.323 1.407 1.069 1.835 1.989 0.756 1.320 0.859 0.937 0.559 1.315 1.452 6.00

S. geminata 55 Texas, Lampasas Co. 3 1.700 1.873 2.121 2.081 2.083 1.605 0.797 1.158 0.829 0.695 1.282 0.974 1.114 1.233 0.953 1.345 1.496 1.116 1.993 2.100 0.908 1.418 0.913 0.995 0.622 1.343 1.559 6.31

S. geminata 55 Texas, Lampasas Co. 4 1.910 1.889 2.178 2.096 2.124 1.638 0.801 1.151 0.845 0.638 1.291 1.023 1.091 1.249 0.931 1.341 1.402 1.143 1.914 2.154 0.865 1.476 0.909 1.006 0.627 1.330 1.535 6.38

S. geminata 55 Texas, Lampasas Co. 5 1.750 1.854 2.087 2.093 2.032 1.676 0.809 1.148 0.804 0.647 1.230 0.935 1.118 1.219 0.931 1.396 1.418 1.125 1.975 2.098 0.795 1.425 0.893 0.990 0.589 1.302 1.552 6.24

S. geminata 55 Texas, Lampasas Co. 6 1.700 1.882 2.050 2.006 2.022 1.747 0.800 1.130 0.884 0.698 1.249 0.993 1.075 1.239 0.952 1.329 1.396 1.128 1.928 2.081 0.935 1.355 0.874 1.002 0.599 1.378 1.492 6.34

S. geminata 55 Texas, Lampasas Co. 7 1.200 1.688 1.824 1.780 1.82 1.580 0.741 1.100 0.829 0.657 1.204 0.911 1.039 1.162 0.916 1.248 1.320 1.090 1.825 1.879 0.757 1.226 0.807 0.921 0.509 1.210 1.392 5.70

S. geminata 55 Texas, Lampasas Co. 8 0.670 1.470 1.507 1.449 1.455 1.242 0.604 1.023 0.768 0.641 1.124 0.860 0.968 1.076 0.854 1.170 1.294 1.029 1.760 1.712 0.590 1.147 0.674 0.883 0.465 1.119 1.303 5.18

S. geminata 55 Texas, Lampasas Co. 9 1.750 1.831 2.069 2.046 2.037 1.640 0.807 1.149 0.808 0.653 1.209 1.001 1.082 1.272 0.931 1.286 1.460 1.038 1.879 2.028 0.837 1.315 0.883 0.944 0.595 1.270 1.505 6.07

S. geminata 55 Texas, Lampasas Co. 10 1.270 1.645 1.759 1.753 1.688 1.389 0.681 1.108 0.799 0.660 1.118 0.934 1.013 1.168 0.925 1.251 1.385 1.098 1.803 1.846 0.771 1.183 0.788 0.881 0.508 1.150 1.445 5.52

S. geminata 55 Texas, Lampasas Co. 11 1.360 1.713 1.872 1.840 1.797 1.519 0.777 1.105 0.819 0.671 1.166 0.952 1.038 1.126 0.895 1.261 1.384 1.081 1.872 1.972 0.757 1.250 0.841 0.952 0.551 1.146 1.384 5.78

S. geminata 55 Texas, Lampasas Co. 12 0.210 0.931 0.817 0.737 0.767 0.677 0.419 0.774 0.515 0.452 0.708 0.617 0.750 0.765 0.596 0.875 0.968 0.740 1.358 1.201 0.400 0.729 0.554 0.561 0.292 0.719 0.751 3.41

S. geminata 55 Texas, Lampasas Co. 13 0.390 1.162 1.119 1.067 1.044 0.894 0.409 0.936 0.668 0.593 0.968 0.755 0.851 0.944 0.736 1.029 1.143 0.886 1.624 1.443 0.460 0.896 0.625 0.808 0.388 0.899 0.909 4.31

S. geminata 55 Texas, Lampasas Co. 14 0.180 0.865 0.757 0.699 0.694 0.611 0.360 0.718 0.555 0.497 0.702 0.572 0.679 0.686 0.554 0.833 0.854 0.714 1.249 1.122 0.373 0.680 0.494 0.599 0.269 0.731 0.743 3.32

S. geminata 55 Texas, Lampasas Co. 15 0.160 0.821 0.693 0.654 0.658 0.584 0.356 0.682 0.509 0.478 0.670 0.593 0.631 0.644 0.499 0.767 0.842 0.651 0.952 1.071 0.389 0.646 0.458 0.581 0.233 0.596 0.681 3.07

S. geminata 55 Texas, Lampasas Co. 16 0.140 0.835 0.737 0.698 0.698 0.600 0.397 0.719 0.543 0.471 0.691 0.591 0.670 0.674 0.549 0.812 0.869 0.698 1.210 1.114 0.391 0.690 0.438 0.597 0.252 0.605 0.770 3.15

S. geminata 55 Texas, Lampasas Co. 17 0.190 0.892 0.762 0.730 0.74 0.654 0.377 0.768 0.542 0.508 0.753 0.533 0.755 0.716 0.593 0.807 0.946 0.720 1.229 1.178 0.417 0.723 0.507 0.589 0.297 0.756 0.744 3.42

S. geminata 55 Texas, Lampasas Co. 18 0.200 0.974 0.847 0.791 0.804 0.711 0.454 0.793 0.585 0.520 0.775 0.604 0.739 0.762 0.627 0.896 0.989 0.753 1.346 1.233 0.438 0.755 0.530 0.615 0.280 0.821 0.793 3.64

S. geminata 55 Texas, Lampasas Co. 19 0.210 0.925 0.814 0.753 0.757 0.645 0.417 0.777 0.571 0.534 0.780 0.622 0.692 0.760 0.593 0.882 0.958 0.740 1.322 1.200 0.401 0.732 0.525 0.595 0.303 0.830 0.770 3.55

S. geminata 55 Texas, Lampasas Co. 20 0.170 0.840 0.732 0.691 0.683 0.592 0.309 0.706 0.532 0.468 0.702 0.549 0.636 0.634 0.512 0.819 0.832 0.638 1.170 1.076 0.379 0.682 0.497 0.571 0.277 0.670 0.784 3.16

S. geminata 55 Texas, Lampasas Co. 21 0.160 0.822 0.692 0.638 0.648 0.573 0.319 0.673 0.490 0.479 0.658 0.515 0.626 0.627 0.524 0.716 0.821 0.647 1.112 1.052 0.386 0.631 0.466 0.550 0.253 0.617 0.628 3.04

S. geminata 55 Texas, Lampasas Co. 22 0.120 0.818 0.698 0.658 0.667 0.612 0.355 0.704 0.506 0.488 0.691 0.504 0.663 0.644 0.542 0.774 0.823 0.676 1.154 1.117 0.372 0.675 0.504 0.555 0.268 0.649 0.665 3.14

S. geminata 55 Texas, Lampasas Co. 23 0.140 0.816 0.687 0.649 0.661 0.590 0.348 0.696 0.495 0.457 0.660 0.505 0.624 0.648 0.508 0.775 0.854 0.627 1.148 1.012 0.352 0.633 0.458 0.587 0.252 0.694 0.700 3.11

S. geminata 55 Texas, Lampasas Co. 24 0.120 0.827 0.714 0.649 0.669 0.589 0.313 0.686 0.498 0.480 0.674 0.541 0.660 0.673 0.498 0.775 0.808 0.653 1.171 1.066 0.395 0.667 0.470 0.582 0.270 0.709 0.697 3.18

S. geminata 55 Texas, Lampasas Co. 25 0.150 0.844 0.720 0.682 0.694 0.623 0.344 0.711 0.516 0.515 0.711 0.568 0.670 0.699 0.548 0.832 0.858 0.750 1.243 1.114 0.440 0.697 0.492 0.668 0.260 0.676 0.692 3.30

S. geminata 55 Texas, Lampasas Co. 26 0.140 0.838 0.719 0.673 0.69 0.613 0.357 0.731 0.531 0.480 0.702 0.563 0.670 0.683 0.540 0.807 0.861 0.698 1.233 1.108 0.379 0.682 0.490 0.559 0.278 0.697 0.710 3.20

S. geminata 55 Texas, Lampasas Co. 27 0.130 0.751 0.665 0.612 0.625 0.552 0.346 0.644 0.459 0.493 0.629 0.497 0.613 0.633 0.478 0.728 0.773 0.616 1.111 1.006 0.330 0.619 0.452 0.557 0.254 0.613 0.596 2.93

S. geminata 55 Texas, Lampasas Co. 28 0.130 0.814 0.705 0.655 0.66 0.613 0.310 0.702 0.494 0.500 0.671 0.538 0.653 0.669 0.533 0.773 0.830 0.679 1.151 1.088 0.384 0.677 0.447 0.575 0.264 0.727 0.699 3.20

S. geminata 55 Texas, Lampasas Co. 29 0.110 0.774 0.642 0.596 0.604 0.517 0.286 0.660 0.463 0.453 0.619 0.511 0.589 0.600 0.480 0.733 0.756 0.604 1.084 0.990 0.351 0.633 0.409 0.528 0.239 0.643 0.595 2.93

S. geminata 55 Texas, Lampasas Co. 30 0.120 0.827 0.703 0.638 0.656 0.589 0.361 0.704 0.491 0.523 0.686 0.551 0.628 0.661 0.528 0.749 0.830 0.680 1.161 1.060 0.400 0.658 0.461 0.574 0.254 0.649 0.682 3.11

S. geminata 55 Texas, Lampasas Co. 31 0.140 0.833 0.692 0.665 0.67 0.604 0.383 0.705 0.493 0.485 0.711 0.543 0.638 0.676 0.529 0.778 0.853 0.682 1.183 1.101 0.378 0.654 0.464 0.584 0.259 0.721 0.694 3.24

S. geminata 55 Texas, Lampasas Co. 32 0.160 0.871 0.752 0.693 0.703 0.629 0.354 0.731 0.509 0.524 0.722 0.558 0.643 0.687 0.570 0.776 0.877 0.698 1.194 1.109 0.405 0.698 0.467 0.565 0.268 0.651 0.674 3.20

S. geminata 55 Texas, Lampasas Co. 33 0.170 0.839 0.709 0.673 0.692 0.609 0.332 0.702 0.479 0.485 0.689 0.517 0.649 0.651 0.525 0.770 0.849 0.676 1.149 1.082 0.356 0.660 0.467 0.567 0.266 0.649 0.649 3.14

S. geminata 55 Texas, Lampasas Co. 34 0.170 0.822 0.710 0.660 0.662 0.548 0.303 0.689 0.498 0.485 0.694 0.541 0.646 0.646 0.526 0.774 0.841 0.676 1.162 1.044 0.376 0.654 0.457 0.611 0.272 0.736 0.651 3.21

S. geminata 55 Texas, Lampasas Co. 35 0.130 0.795 0.665 0.622 0.613 0.530 0.381 0.663 0.508 0.458 0.654 0.518 0.621 0.643 0.507 0.757 0.840 0.652 1.161 1.035 0.333 0.626 0.445 0.510 0.256 0.568 0.622 2.91

S. geminata 55 Texas, Lampasas Co. 36 0.110 0.872 0.748 0.699 0.695 0.617 0.409 0.703 0.554 0.477 0.719 0.562 0.671 0.678 0.559 0.788 0.845 0.692 1.216 1.088 0.396 0.677 0.496 0.536 0.269 0.743 0.774 3.24

S. geminata 55 Texas, Lampasas Co. 37 0.140 0.849 0.725 0.672 0.689 0.650 0.382 0.715 0.524 0.479 0.703 0.581 0.641 0.681 0.537 0.826 0.908 0.658 1.102 0.387 0.691 0.491 0.576 0.275 0.694 0.695 3.22

S. geminata 55 Texas, Lampasas Co. 38 0.150 0.852 0.733 0.684 0.696 0.627 0.368 0.731 0.494 0.510 0.708 0.555 0.677 0.646 0.536 0.800 0.872 0.677 1.185 1.096 0.392 0.667 0.475 0.576 0.281 0.680 0.647 3.20

S. geminata 55 Texas, Lampasas Co. 39 0.150 0.790 0.669 0.616 0.638 0.594 0.352 0.683 0.484 0.476 0.645 0.519 0.622 0.624 0.508 0.752 0.800 0.636 1.107 1.033 0.330 0.634 0.451 0.562 0.260 0.628 0.599 3.01

S. geminata 55 Texas, Lampasas Co. 40 0.100 0.813 0.679 0.652 0.661 0.614 0.350 0.681 0.492 0.487 0.663 0.530 0.636 0.656 0.508 0.768 0.818 0.650 1.145 1.044 0.363 0.626 0.475 0.528 0.255 0.697 0.627 3.08

S. xyloni 56 OK, Caddo 1 1.020 1.353 1.451 1.428 1.278 1.116 0.693 0.910 0.733 0.651 0.995 0.726 0.642 1.141 0.761 0.834 1.200 0.908 1.682 1.827 0.696 1.204 0.811 1.052 0.510 1.503 1.484 5.73

S. xyloni 56 OK, Caddo 2 0.880 1.372 1.387 1.393 1.274 1.157 0.694 0.862 0.704 0.616 1.046 0.797 0.889 1.012 0.733 0.591 1.330 0.913 0.923 1.990 0.726 1.428 0.909 1.076 0.504 1.742 1.670 6.18

S. xyloni 56 OK, Caddo 3 0.970 1.393 1.474 1.514 1.328 1.169 0.662 0.915 0.698 0.600 1.042 0.890 0.738 1.134 0.809 1.119 1.317 0.860 1.473 1.838 0.687 1.215 0.756 1.025 0.544 1.515 1.454 5.77

S. xyloni 56 OK, Caddo 4 1.210 1.331 1.412 1.395 1.235 1.182 0.656 0.882 0.686 0.595 0.945 0.785 0.919 1.144 0.789 1.110 1.153 0.929 1.638 1.757 0.597 1.130 0.760 0.985 0.521 1.850 1.551 5.92

S. xyloni 56 OK, Caddo 5 0.740 1.326 1.336 1.345 1.218 1.202 0.662 0.887 0.725 0.625 1.021 0.756 0.961 1.120 0.762 1.078 1.110 0.914 1.627 1.720 0.704 1.136 0.770 0.977 0.487 1.675 1.517 5.70

S. xyloni 56 OK, Caddo 6 0.860 1.320 1.395 1.401 1.261 1.164 0.597 0.861 0.651 0.634 0.996 0.755 0.909 0.943 0.727 1.107 1.130 0.876 1.661 1.757 0.662 1.172 0.717 0.972 0.476 1.364 1.370 5.41

S. xyloni 56 OK, Caddo 7 0.890 1.356 1.423 1.389 1.291 1.203 0.591 0.917 0.706 0.604 0.990 0.819 0.739 1.004 0.766 1.068 1.194 0.895 1.635 1.759 0.659 1.127 0.740 0.948 0.499 1.448 1.306 5.51

S. xyloni 56 OK, Caddo 8 0.910 1.332 1.441 1.379 1.296 1.220 0.691 0.839 0.717 0.590 1.020 0.805 0.942 1.011 0.822 1.110 1.177 0.948 1.618 1.740 0.699 1.133 0.779 0.989 0.454 1.430 1.445 5.49

S. xyloni 56 OK, Caddo 9 0.670 1.280 1.291 1.274 1.187 1.124 0.563 0.803 0.641 0.568 0.957 0.775 0.865 0.911 0.730 1.036 1.108 0.863 1.558 1.633 0.607 1.009 0.720 0.880 0.438 1.434 1.244 5.23

S. xyloni 56 OK, Caddo 10 0.500 1.138 1.194 1.179 1.118 1.101 0.511 0.800 0.643 0.575 0.895 0.733 0.784 0.857 0.677 0.950 1.011 0.867 1.300 1.587 0.567 1.011 0.607 0.867 0.428 1.267 1.210 4.86

S. xyloni 56 OK, Caddo 11 0.280 0.934 0.876 0.807 0.835 0.801 0.401 0.757 0.443 0.502 0.730 0.591 0.644 0.735 0.542 0.697 0.851 0.663 1.058 1.217 0.428 0.756 0.568 0.574 0.310 0.863 0.870 3.59

S. xyloni 56 OK, Caddo 12 0.180 0.782 0.727 0.676 0.698 0.634 0.359 0.642 0.432 0.447 0.642 0.521 0.600 0.589 0.463 0.653 0.856 0.589 1.104 1.108 0.365 0.703 0.414 0.545 0.241 0.815 0.717 3.25

S. xyloni 56 OK, Caddo 13 0.340 0.997 0.948 0.925 0.904 0.882 0.403 0.757 0.549 0.506 0.767 0.618 0.735 0.759 0.572 0.843 0.842 0.709 1.292 1.320 0.486 0.762 0.627 0.710 0.303 1.069 0.889 4.10

S. xyloni 56 OK, Caddo 14 0.190 0.841 0.751 0.680 0.719 0.698 0.388 0.665 0.424 0.469 0.628 0.456 0.737 0.653 0.532 0.610 0.746 0.629 1.091 1.106 0.340 0.675 0.461 0.631 0.269 1.017 0.809 3.60

S. xyloni 56 OK, Caddo 15 0.230 0.813 0.773 0.732 0.747 0.732 0.310 0.655 0.511 0.436 0.653 0.523 0.607 0.649 0.480 0.737 0.738 0.660 1.085 1.133 0.432 0.689 0.477 0.679 0.248 0.860 0.846 3.48

S. xyloni 56 OK, Caddo 16 0.280 0.906 0.870 0.824 0.834 0.820 0.425 0.735 0.510 0.501 0.768 0.530 0.706 0.694 0.543 0.772 0.854 0.693 1.215 1.244 0.428 0.763 0.525 0.631 0.315 1.052 0.920 3.83

S. xyloni 56 OK, Caddo 17 0.190 0.836 0.761 0.692 0.724 0.709 0.419 0.615 0.466 0.476 0.624 0.495 0.626 0.609 0.474 0.751 0.739 0.599 1.167 1.162 0.382 0.689 0.540 0.602 0.266 1.000 0.816 3.60

S. xyloni 56 OK, Caddo 18 0.240 0.934 0.870 0.784 0.813 0.784 0.399 0.713 0.427 0.480 0.716 0.528 0.676 0.726 0.625 0.709 0.815 0.592 1.016 1.306 0.430 0.845 0.476 0.703 0.283 0.963 0.867 3.91

S. xyloni 56 OK, Caddo 19 0.180 0.854 0.784 0.738 0.737 0.680 0.394 0.640 0.480 0.470 0.719 0.473 0.544 0.667 0.454 0.735 0.797 0.601 1.107 1.156 0.313 0.690 0.508 0.588 0.276 0.850 0.752 3.45

S. xyloni 56 OK, Caddo 20 0.240 0.865 0.807 0.755 0.759 0.744 0.357 0.663 0.500 0.465 0.692 0.599 0.656 0.648 0.494 0.786 0.755 0.657 1.187 1.151 0.415 0.689 0.528 0.668 0.260 0.908 0.752 3.59

S. xyloni 56 OK, Caddo 21 0.210 0.847 0.778 0.732 0.75 0.726 0.415 0.673 0.478 0.465 0.651 0.553 0.561 0.663 0.479 0.789 0.794 0.643 1.060 1.122 0.381 0.700 0.525 0.624 0.283 0.902 0.794 3.50

S. xyloni 56 OK, Caddo 22 0.170 0.767 0.721 0.700 0.714 0.706 0.347 0.641 0.483 0.455 0.652 0.495 0.600 0.548 0.497 0.698 0.724 0.578 1.090 1.078 0.332 0.645 0.494 0.569 0.256 0.925 0.751 3.34

S. xyloni 56 OK, Caddo 23 0.210 0.840 0.798 0.722 0.763 0.760 0.374 0.662 0.452 0.496 0.696 0.582 0.498 0.607 0.461 0.800 0.795 0.652 1.033 1.099 0.370 0.706 0.484 0.571 0.275 0.811 0.786 3.32

S. xyloni 56 OK, Caddo 24 0.220 0.886 0.808 0.745 0.775 0.739 0.399 0.653 0.491 0.413 0.699 0.538 0.605 0.594 0.506 0.766 0.679 0.625 1.143 1.081 0.344 0.704 0.520 0.626 0.297 1.000 0.769 3.59

S. xyloni 56 OK, Caddo 25 0.240 0.830 0.755 0.709 0.741 0.709 0.356 0.663 0.360 0.448 0.631 0.472 0.632 0.616 0.481 0.715 0.738 0.624 1.110 1.053 0.373 0.631 0.471 0.553 0.259 1.050 0.774 3.49

S. xyloni 56 OK, Caddo 26 0.190 0.866 0.808 0.756 0.79 0.763 0.450 0.663 0.465 0.493 0.669 0.515 0.640 0.660 0.505 0.781 0.808 0.621 0.946 1.133 0.382 0.705 0.476 0.601 0.256 1.012 0.822 3.61

S. xyloni 56 OK, Caddo 27 0.280 0.849 0.778 0.732 0.742 0.709 0.400 0.651 0.466 0.464 0.642 0.515 0.588 0.628 0.505 0.760 0.807 0.630 1.119 1.150 0.421 0.681 0.459 0.605 0.265 0.804 0.662 3.41

S. xyloni 56 OK, Caddo 28 0.210 0.885 0.802 0.745 0.768 0.729 0.365 0.697 0.426 0.491 0.675 0.561 0.587 0.640 0.513 0.716 0.739 0.571 1.154 1.125 0.404 0.674 0.474 0.616 0.277 0.875 0.791 3.50

S. xyloni 56 OK, Caddo 29 0.190 0.778 0.715 0.663 0.678 0.622 0.333 0.628 0.432 0.500 0.641 0.489 0.556 0.612 0.480 0.686 0.758 0.602 1.034 1.123 0.366 0.664 0.466 0.600 0.243 0.843 0.766 3.34

S. xyloni 56 OK, Caddo 30 0.160 0.842 0.751 0.707 0.717 0.667 0.353 0.607 0.411 0.513 0.621 0.484 0.675 0.580 0.471 0.722 0.740 0.594 1.119 1.092 0.347 0.654 0.473 0.545 0.260 0.775 0.679 3.25

S. xyloni 56 OK, Caddo 31 0.170 0.773 0.703 0.663 0.678 0.605 0.343 0.640 0.459 0.446 0.610 0.477 0.556 0.606 0.447 0.691 0.808 0.558 1.080 1.055 0.311 0.600 0.505 0.533 0.262 0.815 0.665 3.18

S. xyloni 56 OK, Caddo 32 0.170 0.851 0.737 0.666 0.707 0.665 0.365 0.627 0.387 0.463 0.651 0.502 0.588 0.556 0.509 0.748 0.750 0.611 1.118 1.102 0.379 0.649 0.481 0.574 0.261 0.900 0.775 3.43

S. xyloni 56 OK, Caddo 33 0.190 0.796 0.709 0.663 0.69 0.663 0.361 0.644 0.356 0.312 0.690 0.468 0.572 0.671 0.439 0.686 0.681 0.576 1.043 1.044 0.328 0.612 0.479 0.458 0.236 0.749 0.671 3.05

S. xyloni 56 OK, Caddo 34 0.180 0.779 0.692 0.674 0.678 0.617 0.350 0.618 0.431 0.425 0.627 0.489 0.572 0.616 0.432 0.698 0.747 0.509 1.007 1.058 0.372 0.640 0.465 0.514 0.226 0.857 0.676 3.21

S. xyloni 56 OK, Caddo 35 0.170 0.772 0.704 0.651 0.675 0.640 0.338 0.660 0.456 0.443 0.629 0.466 0.450 0.573 0.465 0.667 0.786 0.619 1.025 1.067 0.337 0.666 0.461 0.572 0.228 0.810 0.700 3.22

S. xyloni 56 OK, Caddo 36 0.160 0.750 0.662 0.609 0.64 0.615 0.351 0.597 0.395 0.434 0.554 0.470 0.555 0.534 0.439 0.653 0.722 0.560 1.025 0.985 0.376 0.578 0.386 0.545 0.203 0.757 0.711 3.04

S. xyloni 56 OK, Caddo 37 0.180 0.774 0.706 0.656 0.689 0.663 0.354 0.622 0.432 0.470 0.528 0.487 0.590 0.646 0.430 0.698 0.657 0.569 1.022 1.094 0.267 0.630 0.450 0.686 0.163 0.780 0.688 3.33

S. xyloni 56 OK, Caddo 38 0.150 0.768 0.695 0.679 0.674 0.637 0.344 0.614 0.398 0.466 0.622 0.468 0.588 0.575 0.440 0.672 0.699 0.527 1.065 1.001 0.312 0.643 0.439 0.218

S. xyloni 56 OK, Caddo 39 0.150 0.767 0.692 0.651 0.672 0.640 0.361 0.611 0.463 0.456 0.618 0.495 0.594 0.611 0.425 0.731 0.675 0.579 1.049 1.069 0.331 0.607 0.429 0.551 0.231 0.705 0.700 3.09

S. xyloni 56 OK, Caddo 40 0.120 0.805 0.695 0.647 0.661 0.642 0.301 0.614 0.423 0.437 0.537 0.451 0.569 0.513 0.418 0.669 0.612 0.533 1.049 1.006 0.309 0.620 0.460 0.459 0.224 0.786 0.665 3.06

S. xyloni 57 OK, Caddo 1 0.960 1.341 1.419 1.363 1.259 1.034 0.726 0.841 0.646 0.608 0.995 0.856 0.873 1.144 0.750 1.022 1.315 0.918 1.678 1.824 0.674 1.221 0.758 0.990 0.483 1.545 1.619 5.70

S. xyloni 57 OK, Caddo 2 0.920 1.368 1.451 1.410 1.272 1.028 0.736 0.793 0.671 0.604 0.969 0.785 0.868 0.961 0.728 1.039 1.111 0.866 1.447 1.776 0.676 1.137 0.768 0.957 0.485 1.321 1.511 5.42

S. xyloni 57 OK, Caddo 3 0.950 1.369 1.447 1.374 1.304 1.151 0.751 0.901 0.635 0.611 0.988 0.847 0.589 1.041 0.738 1.106 1.114 0.869 1.656 1.798 0.683 1.195 0.749 0.913 0.474 1.865 1.450 5.95

S. xyloni 57 OK, Caddo 4 0.970 1.372 1.457 1.436 1.312 1.196 0.834 0.820 0.667 0.613 1.023 0.797 0.872 1.007 0.687 1.027 1.091 0.877 1.646 1.753 0.664 1.199 0.730 1.045 0.480 1.674 1.539 5.84

S. xyloni 57 OK, Caddo 5 0.950 1.375 1.430 1.418 1.313 1.091 0.715 0.885 0.642 0.588 1.026 0.809 0.873 1.022 0.747 1.114 1.177 0.955 1.674 1.770 0.651 1.202 0.765 0.929 0.490 2.056 1.427 6.13

S. xyloni 57 OK, Caddo 6 0.920 1.346 1.405 1.351 1.259 1.042 0.665 0.850 0.665 0.601 1.026 0.772 0.928 0.972 0.785 1.008 1.128 0.905 1.687 1.782 0.529 1.194 0.807 0.969 0.461 1.310 1.499 5.41

S. xyloni 57 OK, Caddo 7 0.910 1.365 1.409 1.364 1.271 1.029 0.691 0.875 0.630 0.558 0.967 0.788 0.892 1.079 0.739 1.120 1.064 0.893 1.708 1.798 0.575 1.137 0.836 0.939 0.488 1.719 1.450 5.82

S. xyloni 57 OK, Caddo 8 0.940 1.322 1.368 1.290 1.225 1.024 0.688 0.857 0.652 0.601 0.894 0.812 0.829 1.046 0.736 1.072 1.107 0.919 1.640 1.689 0.642 1.104 0.655 0.878 0.432 1.780 1.428 5.67

S. xyloni 57 OK, Caddo 9 0.890 1.319 1.400 1.391 1.241 1.024 0.693 0.817 0.644 0.599 0.939 0.760 0.896 0.938 0.751 0.979 1.141 0.939 1.665 1.769 0.676 1.142 0.764 0.918 0.473 1.607 1.449 5.61

S. xyloni 57 OK, Caddo 10 0.800 1.280 1.328 1.301 1.213 1.048 0.688 0.855 0.666 0.604 0.966 0.781 0.776 1.054 0.715 1.018 1.173 0.852 1.610 1.670 0.721 1.068 0.733 0.915 0.450 1.348 1.364 5.21

S. xyloni 57 OK, Caddo 11 0.700 1.214 1.278 1.227 1.163 0.934 0.626 0.842 0.510 0.577 0.903 0.718 0.817 0.925 0.688 0.971 1.060 0.846 1.508 1.579 0.547 1.001 0.684 0.810 0.428 1.686 1.341 5.29

S. xyloni 57 OK, Caddo 12 0.680 1.198 1.226 1.136 1.145 0.990 0.717 0.822 0.596 0.575 0.953 0.705 0.805 0.896 0.716 0.935 1.067 0.771 1.560 1.635 0.526 0.977 0.729 0.869 0.382 1.307 1.243 5.01

S. xyloni 57 OK, Caddo 13 0.300 0.972 0.944 0.872 0.907 0.755 0.535 0.726 0.528 0.511 0.886 0.651 0.547 0.772 0.557 0.804 0.907 0.733 1.278 1.281 0.371 0.843 0.513 0.709 0.305 1.045 0.974 4.01

S. xyloni 57 OK, Caddo 14 0.480 1.123 1.101 1.017 1.023 0.911 0.599 0.784 0.537 0.560 0.852 0.715 0.732 0.917 0.651 0.807 1.018 0.759 1.430 1.420 0.480 0.868 0.623 0.755 0.360 1.433 1.096 4.73

S. xyloni 57 OK, Caddo 15 0.220 0.844 0.806 0.721 0.741 0.659 0.527 0.558 0.463 0.475 0.649 0.515 0.590 0.594 0.459 0.705 0.711 0.539 1.052 1.124 0.361 0.689 0.458 0.519 0.283 1.057 0.882 3.54

S. xyloni 57 OK, Caddo 16 0.190 0.847 0.755 0.700 0.752 0.654 0.453 0.621 0.451 0.475 0.597 0.487 0.640 0.583 0.469 0.695 0.753 0.510 1.109 1.141 0.390 0.673 0.483 0.596 0.247 1.056 0.798 3.64

S. xyloni 57 OK, Caddo 17 0.170 0.790 0.740 0.681 0.731 0.599 0.412 0.573 0.425 0.605 0.478 0.588 0.523 0.492 0.684 0.684 0.586 1.027 1.082 0.377 0.641 0.478 0.562 0.259 1.092 0.822 3.53

S. xyloni 57 OK, Caddo 18 0.200 0.834 0.783 0.721 0.756 0.655 0.463 0.631 0.425 0.442 0.610 0.487 0.570 0.596 0.705 0.805 0.610 0.621 1.164 0.382 0.682 0.535 0.572 0.273 0.867 0.734 3.44

S. xyloni 57 OK, Caddo 19 0.180 0.833 0.760 0.672 0.73 0.641 0.431 0.629 0.453 0.468 0.662 0.544 0.586 0.606 0.460 0.683 0.749 0.586 1.087 1.172 0.395 0.653 0.552 0.679 0.238 0.809 0.855 3.49

S. xyloni 57 OK, Caddo 20 0.170 0.850 0.785 0.700 0.735 0.638 0.419 0.590 0.481 0.469 0.626 0.525 0.544 0.577 0.461 0.650 0.753 0.584 1.096 1.130 0.424 0.686 0.484 0.590 0.260 0.880 0.750 3.45

S. xyloni 57 OK, Caddo 21 0.170 0.788 0.758 0.680 0.722 0.649 0.437 0.623 0.437 0.466 0.744 0.508 0.568 0.587 0.501 0.715 0.760 0.606 1.109 1.107 0.340 0.665 0.469 0.567 0.236 0.768 0.811 3.23

S. xyloni 57 OK, Caddo 22 0.230 0.832 0.777 0.726 0.745 0.654 0.464 0.603 0.418 0.469 0.601 0.494 0.616 0.569 0.478 0.715 0.706 0.604 1.113 1.099 0.327 0.664 0.496 0.596 0.276 0.861 0.706 3.39

S. xyloni 57 OK, Caddo 23 0.210 0.860 0.782 0.721 0.756 0.682 0.459 0.634 0.470 0.464 0.650 0.513 0.650 0.639 0.481 0.734 0.838 0.623 1.125 1.102 0.350 0.689 0.485 0.629 0.260 0.953 0.792 3.54

S. xyloni 57 OK, Caddo 24 0.220 0.838 0.754 0.670 0.723 0.632 0.436 0.578 0.438 0.445 0.658 0.511 0.559 0.611 0.436 0.728 0.740 0.583 1.079 1.096 0.353 0.663 0.504 0.597 0.263 1.070 0.794 3.60

S. xyloni 57 OK, Caddo 25 0.210 0.849 0.762 0.702 0.723 0.626 0.440 0.588 0.419 0.488 0.614 0.488 0.600 0.548 0.410 0.689 0.718 0.585 1.003 1.096 0.323 0.684 0.474 0.562 0.250 1.099 0.759 3.61

S. xyloni 57 OK, Caddo 26 0.160 0.816 0.765 0.693 0.725 0.648 0.469 0.593 0.449 0.457 0.568 0.520 0.592 0.597 0.461 0.682 0.783 0.542 1.070 1.075 0.311 0.700 0.435 0.556 0.263 0.854 0.770 3.30

S. xyloni 57 OK, Caddo 27 0.190 0.838 0.788 0.704 0.745 0.637 0.456 0.634 0.453 0.479 0.588 0.521 0.486 0.617 0.507 0.684 0.811 0.611 1.159 1.126 0.356 0.729 0.520 0.573 0.264 0.874 0.783 3.41

S. xyloni 57 OK, Caddo 28 0.210 0.755 0.773 0.702 0.741 0.641 0.496 0.627 0.392 0.467 0.637 0.539 0.622 0.594 0.530 0.524 0.764 0.590 1.096 1.114 0.327 0.668 0.506 0.596 0.262 1.099 0.861 3.56

S. xyloni 57 OK, Caddo 29 0.170 0.845 0.755 0.690 0.717 0.656 0.460 0.629 0.465 0.459 0.636 0.569 0.525 0.556 0.507 0.626 0.711 0.577 1.035 1.093 0.314 0.645 0.496 0.565 0.287 0.942 0.794 3.45

S. xyloni 57 OK, Caddo 30 0.180 0.828 0.766 0.693 0.727 0.631 0.450 0.615 0.446 0.466 0.583 0.495 0.583 0.592 0.463 0.710 0.759 0.527 1.171 1.074 0.319 0.670 0.455 0.561 0.249 0.851 0.770 3.31

S. xyloni 57 OK, Caddo 31 0.190 0.850 0.767 0.705 0.746 0.684 0.500 0.610 0.442 0.492 0.713 0.541 0.608 0.697 0.498 0.744 0.760 0.528 1.152 1.092 0.363 0.671 0.552 0.476 0.245 0.914 0.803 3.33

S. xyloni 57 OK, Caddo 32 0.160 0.827 0.740 0.692 0.716 0.643 0.455 0.615 0.455 0.474 0.590 0.511 0.581 0.578 0.494 0.686 0.687 0.560 1.148 1.070 0.344 0.677 0.470 0.549 0.247 1.084 0.743 3.53

S. xyloni 57 OK, Caddo 33 0.150 0.812 0.769 0.714 0.732 0.666 0.421 0.629 0.431 0.448 0.606 0.533 0.600 0.627 0.482 0.652 0.703 0.606 1.091 1.064 0.330 0.656 0.494 0.570 0.268 0.793 0.776 3.24

S. xyloni 57 OK, Caddo 34 0.160 0.824 0.741 0.683 0.732 0.622 0.390 0.604 0.450 0.470 0.648 0.536 0.567 0.559 0.508 0.695 0.771 0.615 1.092 1.102 0.351 0.658 0.465 0.589 0.251 0.902 0.769 3.42

S. xyloni 57 OK, Caddo 35 0.190 0.897 0.774 0.705 0.744 0.612 0.417 0.629 0.430 0.469 0.629 0.520 0.613 0.603 0.480 0.676 0.747 0.605 1.100 1.116 0.334 0.694 0.470 0.680 0.262 0.837 0.670 3.53

S. xyloni 57 OK, Caddo 36 0.150 0.782 0.727 0.676 0.686 0.575 0.366 0.581 0.413 0.444 0.703 0.503 0.591 0.632 0.446 0.676 0.751 0.582 1.029 1.047 0.334 0.644 0.463 0.555 0.250 0.788 0.688 3.17

S. xyloni 57 OK, Caddo 37 0.170 0.838 0.777 0.704 0.737 0.661 0.432 0.626 0.508 0.481 0.604 0.505 0.412 0.682 0.474 0.586 0.738 0.619 1.096 1.125 0.360 0.656 0.492 0.591 0.257 0.850 0.755 3.40

S. xyloni 57 OK, Caddo 38 0.160 0.844 0.782 0.710 0.734 0.659 0.448 0.620 0.602 0.488 0.592 0.627 0.487 0.675 0.708 0.625 1.117 1.112 0.387 0.668 0.511 0.627 0.259 0.849 0.736 3.43

S. xyloni 57 OK, Caddo 39 0.140 0.782 0.723 0.644 0.684 0.585 0.399 0.572 0.428 0.436 0.566 0.470 0.588 0.636 0.425 0.673 0.667 0.541 1.057 0.988 0.237 0.646 0.431 0.604 0.259 0.709 0.674 3.08

S. xyloni 57 OK, Caddo 40 0.130 0.749 0.721 0.661 0.693 0.633 0.453 0.521 0.387 0.468 0.565 0.494 0.542 0.548 0.449 0.662 0.642 0.577 0.954 1.128 0.295 0.642 0.552 0.578 0.193 0.686 0.691 3.14

S. xyloni 58 OK, Caddo 1 0.700 1.223 1.265 1.233 1.183 1.060 0.581 0.801 0.670 0.572 0.924 0.800 0.826 0.888 0.690 1.007 1.065 0.851 1.505 1.564 0.637 1.006 0.676 0.853 0.390 1.241 1.263 4.88

S. xyloni 58 OK, Caddo 2 0.640 1.234 1.258 1.214 1.151 1.016 0.617 0.803 0.681 0.588 0.938 0.703 0.906 0.908 0.730 1.015 1.085 0.866 1.530 1.587 0.633 0.967 0.663 0.863 0.410 1.295 1.274 4.98

S. xyloni 58 OK, Caddo 3 0.520 1.171 1.102 1.053 1.038 0.898 0.514 0.798 0.604 0.548 0.873 0.701 0.758 0.868 0.658 0.929 1.036 0.778 1.400 1.427 0.509 0.907 0.626 0.745 0.371 1.094 1.180 4.44

S. xyloni 58 OK, Caddo 4 0.590 1.216 1.220 1.214 1.119 0.983 0.539 0.798 0.599 0.564 0.941 0.714 0.638 0.852 0.694 0.951 1.077 0.867 1.206 1.483 0.561 0.933 0.661 0.772 0.380 1.324 1.180 4.79

S. xyloni 58 OK, Caddo 5 0.550 1.233 1.184 1.152 1.075 0.989 0.561 0.806 0.625 0.575 0.892 0.736 0.772 0.874 0.723 0.941 1.079 0.892 1.432 1.522 0.504 0.919 0.653 0.841 0.399 1.194 1.235 4.79

S. xyloni 58 OK, Caddo 6 0.570 1.174 1.196 1.159 1.102 0.963 0.578 0.810 0.617 0.574 0.897 0.692 0.878 0.853 0.752 1.003 1.100 0.835 1.620 1.531 0.584 0.968 0.623 0.813 0.378 1.201 1.187 4.72

S. xyloni 58 OK, Caddo 7 0.520 1.188 1.181 1.127 1.094 0.949 0.539 0.821 0.647 0.606 0.948 0.788 0.817 0.975 0.730 0.993 1.092 0.868 1.530 1.524 0.598 1.009 0.645 0.806 0.410 1.281 1.202 4.80

S. xyloni 58 OK, Caddo 8 0.550 1.185 1.186 1.157 1.065 0.849 0.506 0.818 0.577 0.588 0.890 0.740 0.795 0.863 0.661 1.004 1.038 0.853 1.449 1.461 0.515 0.922 0.678 0.724 0.375 1.647 1.101 5.02

S. xyloni 58 OK, Caddo 9 0.460 1.163 1.125 1.097 1.033 0.941 0.552 0.793 0.605 0.535 0.841 0.722 0.844 0.809 0.663 0.962 1.018 0.798 1.510 1.431 0.533 0.880 0.600 0.730 0.351 1.101 1.158 4.43

S. xyloni 58 OK, Caddo 10 0.580 1.178 1.168 1.151 1.079 0.865 0.517 0.830 0.650 0.580 0.931 0.735 0.800 0.842 0.738 0.998 1.037 0.897 1.456 1.502 0.509 0.985 0.585 0.792 0.383 1.203 1.123 4.67

S. xyloni 58 OK, Caddo 11 0.510 1.169 1.124 1.077 1.011 0.877 0.564 0.806 0.586 0.583 0.829 0.685 0.821 0.836 0.674 0.951 1.039 0.833 1.410 1.427 0.434 0.867 0.585 0.734 0.386 1.209 1.144 4.54

S. xyloni 58 OK, Caddo 12 0.500 1.157 1.141 1.130 1.04 0.811 0.544 0.796 0.597 0.558 0.793 0.698 0.495 0.804 0.616 1.018 1.032 0.822 1.462 1.447 0.550 0.887 0.628 0.717 0.372 1.181 1.108 4.50

S. xyloni 58 OK, Caddo 13 0.470 1.170 1.186 1.130 1.061 0.880 0.535 0.783 0.624 0.532 0.890 0.700 0.766 0.790 0.661 0.949 1.028 0.840 1.436 1.416 0.435 0.888 0.669 0.737 0.372 1.108 1.122 4.43

S. xyloni 58 OK, Caddo 14 0.100 0.731 0.646 0.584 0.602 0.564 0.330 0.539 0.412 0.455 0.557 0.489 0.526 0.513 0.375 0.643 0.566 0.546 0.907 0.929 0.295 0.555 0.360 0.471 0.209 0.762 0.653 2.89

S. xyloni 58 OK, Caddo 15 0.200 0.888 0.818 0.745 0.774 0.657 0.397 0.628 0.496 0.507 0.694 0.542 0.612 0.648 0.529 0.725 0.787 0.648 1.178 1.094 0.345 0.660 0.496 0.567 0.259 0.986 0.741 3.54

S. xyloni 58 OK, Caddo 16 0.170 0.817 0.672 0.646 0.664 0.584 0.358 0.608 0.446 0.469 0.572 0.501 0.555 0.578 0.420 0.673 0.714 0.591 1.004 0.963 0.324 0.590 0.431 0.515 0.218 0.694 0.653 2.99

S. xyloni 58 OK, Caddo 17 0.220 0.919 0.822 0.773 0.784 0.660 0.394 0.661 0.523 0.507 0.717 0.554 0.632 0.690 0.551 0.820 0.870 0.655 1.225 1.138 0.323 0.686 0.469 0.619 0.260 0.796 0.826 3.47

S. xyloni 58 OK, Caddo 18 0.340 1.032 0.927 0.884 0.861 0.701 0.404 0.606 0.522 0.514 0.768 0.627 0.702 0.744 0.584 0.734 0.922 0.712 1.032 1.264 0.416 0.738 0.541 0.749 0.331 0.827 0.912 3.87

S. xyloni 58 OK, Caddo 19 0.150 0.722 0.655 0.619 0.606 0.540 0.321 0.539 0.437 0.448 0.574 0.448 0.581 0.685 0.528 0.789 0.684 0.572 1.007 0.944 0.315 0.562 0.396 0.524 0.214 0.891 0.626 3.08

S. xyloni 58 OK, Caddo 20 0.180 0.804 0.696 0.642 0.656 0.572 0.325 0.597 0.409 0.472 0.568 0.454 0.593 0.656 0.433 0.691 0.644 0.595 1.030 0.995 0.329 0.604 0.417 0.535 0.232 0.752 0.655 3.09

S. xyloni 58 OK, Caddo 21 0.520 1.151 1.149 1.139 1.052 0.909 0.557 0.771 0.584 0.550 0.884 0.691 0.796 0.849 0.645 0.730 1.084 0.801 1.426 1.459 0.536 0.896 0.663 0.746 0.376 1.137 1.173 4.49

S. xyloni 58 OK, Caddo 22 0.260 0.934 0.810 0.772 0.766 0.653 0.412 0.695 0.506 0.491 0.733 0.546 0.673 0.693 0.537 0.802 0.827 0.690 1.152 1.146 0.325 0.688 0.505 0.637 0.278 0.858 0.831 3.58

S. xyloni 58 OK, Caddo 23 0.210 0.868 0.787 0.729 0.728 0.619 0.412 0.668 0.496 0.475 0.640 0.546 0.639 0.616 0.514 0.763 0.802 0.631 0.959 1.118 0.411 0.702 0.475 0.614 0.233 0.793 0.755 3.39

S. xyloni 58 OK, Caddo 24 0.150 0.780 0.674 0.642 0.629 0.548 0.352 0.601 0.411 0.453 0.583 0.497 0.539 0.525 0.410 0.687 0.672 0.567 1.040 0.946 0.285 0.581 0.409 0.536 0.223 0.700 0.621 2.96

S. xyloni 58 OK, Caddo 25 0.160 0.815 0.728 0.674 0.7 0.623 0.379 0.622 0.460 0.477 0.627 0.489 0.382 0.611 0.489 0.578 0.749 0.626 0.942 1.065 0.333 0.631 0.433 0.533 0.235 0.842 0.719 3.25

S. xyloni 58 OK, Caddo 26 0.140 0.742 0.658 0.615 0.632 0.564 0.404 0.605 0.385 0.453 0.560 0.446 0.565 0.535 0.435 0.635 0.685 0.540 1.046 0.924 0.345 0.586 0.389 0.537 0.216 0.691 0.658 2.89

S. xyloni 58 OK, Caddo 27 0.120 0.761 0.658 0.599 0.608 0.491 0.268 0.579 0.448 0.449 0.568 0.518 0.510 0.546 0.425 0.643 0.648 0.555 0.857 0.953 0.309 0.586 0.416 0.543 0.209 0.712 0.626 2.97

S. xyloni 58 OK, Caddo 28 0.130 0.741 0.631 0.594 0.637 0.557 0.350 0.560 0.417 0.447 0.589 0.499 0.547 0.534 0.429 0.640 0.772 0.526 0.883 0.956 0.296 0.566 0.441 0.488 0.225 0.791 0.612 2.98

S. xyloni 58 OK, Caddo 29 0.150 0.756 0.681 0.627 0.628 0.562 0.323 0.482 0.453 0.455 0.581 0.424 0.612 0.584 0.437 0.672 0.691 0.530 1.085 1.016 0.372 0.611 0.455 0.498 0.230 0.914 0.676 3.18

S. xyloni 58 OK, Caddo 30 0.170 0.747 0.701 0.653 0.635 0.583 0.375 0.571 0.435 0.467 0.585 0.472 0.556 0.504 0.448 0.664 0.654 0.554 1.025 1.001 0.297 0.577 0.437 0.458 0.224 0.806 0.604 3.01

S. xyloni 58 OK, Caddo 31 0.100 0.751 0.666 0.617 0.622 0.553 0.332 0.583 0.383 0.448 0.583 0.441 0.555 0.554 0.398 0.664 0.651 0.568 0.940 0.981 0.331 0.585 0.407 0.558 0.248 0.799 0.691 3.09

S. xyloni 58 OK, Caddo 32 0.110 0.726 0.656 0.599 0.618 0.567 0.357 0.587 0.392 0.436 0.554 0.432 0.516 0.536 0.425 0.622 0.637 0.518 0.963 0.921 0.290 0.549 0.406 0.521 0.220 0.821 0.658 2.99

S. xyloni 58 OK, Caddo 33 0.100 0.734 0.635 0.571 0.584 0.530 0.338 0.560 0.413 0.431 0.553 0.453 0.541 0.514 0.421 0.590 0.647 0.546 0.963 0.917 0.317 0.550 0.397 0.460 0.208 0.594 0.561 2.71

S. xyloni 58 OK, Caddo 34 0.110 0.716 0.618 0.572 0.576 0.504 0.331 0.548 0.410 0.422 0.546 0.426 0.551 0.522 0.370 0.645 0.622 0.536 0.914 0.910 0.312 0.554 0.357 0.501 0.200 0.680 0.594 2.81

S. xyloni 58 OK, Caddo 35 0.080 0.718 0.639 0.600 0.621 0.534 0.314 0.550 0.421 0.443 0.556 0.446 0.541 0.504 0.410 0.633 0.700 0.554 0.946 0.921 0.342 0.578 0.437 0.506 0.213 0.665 0.594 2.81

S. xyloni 58 OK, Caddo 36 0.090 0.775 0.681 0.628 0.644 0.564 0.357 0.585 0.399 0.378 0.539 0.484 0.554 0.516 0.439 0.662 0.707 0.575 1.036 0.971 0.332 0.607 0.422 0.521 0.237 0.809 0.658 3.08

S. xyloni 58 OK, Caddo 37 0.080 0.669 0.604 0.561 0.561 0.508 0.274 0.540 0.382 0.432 0.554 0.431 0.507 0.523 0.385 0.623 0.583 0.450 0.980 0.843 0.298 0.535 0.330 0.519 0.212 0.648 0.550 2.68

S. xyloni 58 OK, Caddo 38 0.070 0.681 0.572 0.518 0.546 0.464 0.303 0.485 0.353 0.399 0.503 0.360 0.526 0.467 0.370 0.592 0.591 0.478 0.870 0.834 0.270 0.511 0.350 0.494 0.205 0.784 0.547 2.79

S. xyloni 58 OK, Caddo 39 0.080 0.752 0.661 0.601 0.616 0.530 0.299 0.577 0.426 0.450 0.499 0.446 0.575 0.540 0.438 0.698 0.727 0.561 1.004 0.957 0.331 0.586 0.396 0.514 0.220 0.809 0.594 3.03

S. xyloni 58 OK, Caddo 40 0.070 0.683 0.604 0.560 0.558 0.457 0.296 0.530 0.405 0.410 0.518 0.419 0.527 0.493 0.412 0.611 0.612 0.496 0.918 0.896 0.291 0.585 0.381 0.482 0.221 0.897 0.572 2.96

S. invicta 60 Argentina, Santa Fe Prov., San Justo 1 1.750 1.411 1.407 1.391 1.248 1.064 0.725 1.004 0.821 0.654 1.169 0.908 0.951 1.149 0.914 0.781 1.35 1.045 1.836 1.899 0.756 1.244 0.783 1.023 0.531 1.282 1.523 5.62

S. invicta 60 Argentina, Santa Fe Prov., San Justo 2 1.370 1.393 1.387 1.348 1.227 1.032 0.613 1.005 0.77 0.672 1.139 0.879 0.967 1.233 0.833 1.169 1.361 1.063 1.61 1.954 0.72 1.208 0.852 0.924 0.515 1.122 1.457 5.39

S. invicta 60 Argentina, Santa Fe Prov., San Justo 3 1.790 1.434 1.392 1.384 1.289 0.972 0.701 1.033 0.819 0.671 1.192 0.954 1.08 1.176 0.914 1.247 1.422 1.083 1.938 1.847 0.688 1.238 0.803 0.965 0.511 1.175 1.713 5.42

S. invicta 60 Argentina, Santa Fe Prov., San Justo 4 0.840 1.427 1.413 1.36 1.233 1.019 0.697 0.998 0.765 0.636 1.107 0.901 0.992 1.111 0.834 1.167 1.354 1.079 1.691 1.888 0.831 1.241 0.81 0.859 0.49 1.102 1.475 5.28

S. invicta 60 Argentina, Santa Fe Prov., San Justo 5 1.880 1.408 1.403 1.358 1.247 0.99 0.665 1.01 0.769 0.604 1.103 0.907 1.008 1.266 0.829 1.166 1.501 1.073 1.473 1.883 0.807 0.776 1.236 0.94 0.502 1.116 1.491 5.35

S. invicta 60 Argentina, Santa Fe Prov., San Justo 6 1.140 1.43 1.397 1.388 1.293 0.986 0.651 1.036 0.832 0.63 1.172 0.922 0.973 1.297 0.916 1.289 1.557 1.104 1.87 1.885 0.777 0.81 1.241 0.961 0.518 1.2 1.54 5.48

S. invicta 60 Argentina, Santa Fe Prov., San Justo 7 1.120 1.32 1.263 1.195 1.132 0.929 0.688 1.025 0.834 0.615 1.079 0.827 1.035 1.147 0.867 1.225 1.426 1.03 1.695 1.73 0.611 0.728 1.114 0.883 0.469 1.088 1.399 5.02

S. invicta 60 Argentina, Santa Fe Prov., San Justo 8 1.090 1.379 1.356 1.323 1.209 0.964 0.685 0.98 0.759 0.611 1.154 0.922 1.027 1.173 0.919 0.839 1.503 1.086 1.838 1.828 0.72 1.181 0.78 0.858 0.513 1.263 1.422 5.33

S. invicta 60 Argentina, Santa Fe Prov., San Justo 9 1.570 1.292 1.261 1.21 1.14 0.968 0.645 0.943 0.726 0.61 1.085 0.869 0.901 1.186 0.843 1.151 1.311 1.018 1.253 1.726 0.698 1.081 0.703 0.804 0.452 0.895 1.207 4.72

S. invicta 60 Argentina, Santa Fe Prov., San Justo 10 0.820 1.327 1.348 1.311 1.251 1.051 0.613 0.962 0.814 0.638 1.159 0.871 0.79 1.292 0.837 1.185 1.371 1.031 1.791 1.851 0.673 1.273 0.759 0.989 0.518 1.218 1.509 5.38

S. invicta 60 Argentina, Santa Fe Prov., San Justo 11 0.910 1.413 1.381 1.328 1.229 1.01 0.75 0.997 0.821 0.533 1.173 0.918 1.026 1.264 0.902 1.252 1.492 0.998 1.884 1.865 0.766 0.753 0.811 0.946 0.508 1.129 1.514 5.35

S. invicta 60 Argentina, Santa Fe Prov., San Justo 12 1.020 1.258 1.235 1.2 1.132 0.931 0.616 0.943 0.764 0.629 0.832 0.832 0.865 1.132 0.859 1.15 1.278 1.04 1.73 1.687 0.638 1.059 0.737 0.803 0.457 0.928 1.231 4.68

S. invicta 60 Argentina, Santa Fe Prov., San Justo 13 0.150 0.853 0.755 0.682 0.7 0.592 0.411 0.68 0.497 0.486 0.747 0.616 0.823 0.584 0.795 0.991 0.727 1.227 1.116 0.384 0.702 0.456 0.354 0.286 0.645 0.737 2.97

S. invicta 60 Argentina, Santa Fe Prov., San Justo 14 0.340 0.898 0.867 0.815 0.794 0.661 0.431 0.737 0.54 0.485 0.949 0.632 0.744 0.884 0.622 0.915 1.092 0.77 1.321 1.26 0.458 0.792 0.526 0.606 0.331 0.667 0.885 3.43

S. invicta 60 Argentina, Santa Fe Prov., San Justo 15 0.120 0.787 0.706 0.656 0.662 0.534 0.405 0.631 0.431 0.432 0.755 0.522 0.628 0.696 0.517 0.733 0.882 0.62 1.126 1.056 0.339 0.665 0.436 0.518 0.253 0.563 0.693 2.92

S. invicta 60 Argentina, Santa Fe Prov., San Justo 16 0.200 0.822 0.77 0.69 0.696 0.636 0.38 0.686 0.541 0.415 0.774 0.578 0.632 0.771 0.556 0.846 0.973 0.69 1.31 1.157 0.38 0.725 0.471 0.597 0.292 0.615 0.732 3.19

S. invicta 60 Argentina, Santa Fe Prov., San Justo 17 0.250 0.741 0.68 0.609 0.647 0.521 0.332 0.597 0.445 0.465 0.751 0.537 0.648 0.718 0.501 0.707 0.876 0.635 1.113 1.046 0.381 0.638 0.464 0.542 0.244 0.645 0.74 2.97

S. invicta 60 Argentina, Santa Fe Prov., San Justo 18 0.260 0.919 0.932 0.876 0.866 0.7 0.466 0.808 0.62 0.484 0.917 0.727 0.721 1.107 0.794 0.936 0.941 0.657 0.912 1.316 0.462 0.816 0.584 0.642 0.338 0.861 0.941 3.74

S. invicta 60 Argentina, Santa Fe Prov., San Justo 19 0.230 0.753 0.701 0.616 0.648 0.548 0.26 0.649 0.435 0.427 0.706 0.537 0.646 0.712 0.466 0.776 0.907 0.63 1.113 1.024 0.348 0.66 0.435 0.557 0.271 0.562 0.665 2.90

S. invicta 60 Argentina, Santa Fe Prov., San Justo 20 0.190 0.769 0.709 0.651 0.654 0.563 0.337 0.679 0.492 0.473 0.74 0.52 0.665 0.72 0.532 0.778 0.723 0.506 0.773 1.073 0.366 0.671 0.425 0.546 0.286 0.569 0.689 2.96

S. invicta 60 Argentina, Santa Fe Prov., San Justo 21 0.160 0.795 0.712 0.644 0.667 0.586 0.287 0.648 0.379 0.353 0.693 0.534 0.683 0.745 0.537 0.738 0.888 0.658 1.181 1.052 0.362 0.674 0.421 0.509 0.267 0.652 0.693 3.01

S. invicta 60 Argentina, Santa Fe Prov., San Justo 22 0.450 1.124 1.097 1.032 0.983 0.82 0.547 0.861 0.749 0.536 1.04 0.782 0.872 1.038 0.747 1.086 1.261 0.992 1.625 1.513 0.517 0.953 0.623 0.725 0.416 0.896 1.082 4.26

S. invicta 60 Argentina, Santa Fe Prov., San Justo 23 0.160 0.741 0.741 0.685 0.655 0.529 0.323 0.658 0.5 0.476 0.744 0.527 0.655 0.726 0.509 0.783 0.881 0.636 0.832 1.059 0.375 0.677 0.423 0.615 0.298 0.608 0.73 3.02

S. invicta 60 Argentina, Santa Fe Prov., San Justo 24 0.210 0.735 0.734 0.677 0.658 0.561 0.32 0.641 0.502 0.472 0.742 0.535 0.676 0.726 0.521 0.766 0.865 0.649 0.806 1.045 0.368 0.666 0.434 0.608 0.301 0.599 0.73 2.99

S. invicta 60 Argentina, Santa Fe Prov., San Justo 25 0.880 1.319 1.278 1.259 1.163 0.938 0.611 0.983 0.758 0.634 1.244 0.851 0.995 1.263 0.891 1.241 1.491 1.082 1.704 1.712 0.671 1.123 0.684 0.754 0.469 1.103 1.492 4.89

S. invicta 60 Argentina, Santa Fe Prov., San Justo 26 0.170 0.694 0.637 0.6 0.591 0.52 0.287 0.619 0.436 0.413 0.717 0.456 0.573 0.608 0.429 0.658 0.798 0.546 0.98 0.957 0.362 0.606 0.41 0.502 0.233 0.542 2.70

S. invicta 60 Argentina, Santa Fe Prov., San Justo 27 0.230 0.843 0.736 0.605 0.7 0.564 0.373 0.667 0.499 0.471 0.778 0.554 0.621 0.759 0.506 0.811 0.908 0.66 1.117 1.139 0.404 0.728 0.463 0.521 0.298 0.725 0.747 3.23

S. invicta 60 Argentina, Santa Fe Prov., San Justo 28 0.150 0.767 0.736 0.659 0.687 0.555 0.344 0.657 0.482 0.421 0.776 0.575 0.647 0.769 0.514 0.773 0.872 0.654 1.224 1.131 0.384 0.717 0.462 0.558 0.282 0.784 0.826 3.24

S. invicta 60 Argentina, Santa Fe Prov., San Justo 29 0.130 0.689 0.645 0.577 0.612 0.527 0.354 0.592 0.452 0.438 0.673 0.434 0.565 0.655 0.453 0.651 0.751 0.558 1.006 1.008 0.358 0.63 0.427 0.526 0.256 0.514 0.668 2.74

S. invicta 60 Argentina, Santa Fe Prov., San Justo 30 0.120 0.73 0.645 0.576 0.601 0.546 0.337 0.6 0.441 0.431 0.711 0.481 0.618 0.656 0.437 0.748 0.797 0.597 1.027 0.979 0.344 0.627 0.401 0.514 0.243 0.562 0.632 2.79

S. invicta 60 Argentina, Santa Fe Prov., San Justo 31 0.190 0.769 0.71 0.651 0.661 0.587 0.38 0.636 0.499 0.438 0.759 0.521 0.674 0.733 0.515 0.779 0.883 0.672 1.087 1.069 0.355 0.704 0.437 0.525 0.284 0.618 0.664 2.98

S. invicta 60 Argentina, Santa Fe Prov., San Justo 32 0.160 0.73 0.667 0.612 0.601 0.519 0.358 0.639 0.444 0.469 0.724 0.433 0.625 0.673 0.445 0.739 0.884 0.615 1.091 1.068 0.397 0.672 0.433 0.547 0.244 0.564 0.63 2.91

S. invicta 60 Argentina, Santa Fe Prov., San Justo 33 0.170 0.778 0.702 0.635 0.651 0.562 0.352 0.657 0.494 0.437 0.77 0.562 0.641 0.746 0.535 0.745 0.847 0.663 1.141 1.06 0.435 0.686 0.435 0.502 0.27 0.634 0.669 2.97

S. invicta 60 Argentina, Santa Fe Prov., San Justo 34 0.130 0.729 0.686 0.63 0.628 0.551 0.295 0.646 0.435 0.52 0.779 0.505 0.473 0.727 0.469 0.77 0.903 0.601 1.099 1.038 0.343 0.646 0.435 0.544 0.264 0.693 0.671 3.00

S. invicta 60 Argentina, Santa Fe Prov., San Justo 35 0.120 0.728 0.645 0.589 0.596 0.465 0.339 0.602 0.447 0.432 0.661 0.471 0.587 0.647 0.427 0.716 0.809 0.555 1.034 1.023 0.396 0.612 0.459 0.47 0.253 0.616 0.624 2.84

S. invicta 60 Argentina, Santa Fe Prov., San Justo 36 0.120 0.686 0.654 0.613 0.598 0.522 0.309 0.599 0.394 0.454 0.712 0.473 0.617 0.682 0.444 0.716 0.834 0.6 0.881 0.977 0.328 0.614 0.406 0.549 0.24 0.568 0.613 2.78

S. invicta 60 Argentina, Santa Fe Prov., San Justo 37 0.120 0.681 0.634 0.581 0.587 0.469 0.319 0.566 0.453 0.433 0.686 0.444 0.588 0.649 0.442 0.595 0.798 0.562 0.999 0.95 0.313 0.604 0.378 0.481 0.237 0.537 0.607 2.65

S. invicta 60 Argentina, Santa Fe Prov., San Justo 38 0.130 0.724 0.646 0.583 0.621 0.547 0.337 0.53 0.431 0.424 0.703 0.485 0.581 0.642 0.428 0.675 0.825 0.573 1.032 1.01 0.359 0.652 0.429 0.519 0.254 0.55 0.659 2.80

S. invicta 60 Argentina, Santa Fe Prov., San Justo 39 0.070 0.616 0.591 0.535 0.507 0.427 0.33 0.537 0.39 0.372 0.616 0.408 0.528 0.566 0.411 0.635 0.702 0.505 0.936 0.927 0.311 0.574 0.398 0.453 0.232 0.539 0.559 2.54

S. invicta 60 Argentina, Santa Fe Prov., San Justo 40 0.080 0.641 0.571 0.55 0.533 0.444 0.3 0.577 0.42 0.375 0.593 0.391 0.523 0.585 0.387 0.58 0.631 0.5 0.917 0.905 0.314 0.546 0.388 0.443 0.23 0.512 0.617 2.50

S. invicta 61 Argentina, Santa Fe Prov., San Justo 1 1.017 1.475 1.342 1.274 1.176 1 0.678 0.991 0.773 0.565 1.279 0.896 0.957 1.272 0.882 1.247 1.53 1.06 1.867 1.872 0.755 1.202 0.824 0.905 0.521 1.203 1.733 5.46

S. invicta 61 Argentina, Santa Fe Prov., San Justo 2 1.430 1.38 1.315 1.29 1.224 1.026 0.715 0.956 0.793 0.598 1.251 0.849 1.02 1.283 0.818 1.324 1.504 1.091 1.849 1.899 0.76 1.206 0.797 0.984 0.517 1.252 1.557 5.51

S. invicta 61 Argentina, Santa Fe Prov., San Justo 3 1.251 1.426 1.451 1.405 1.275 1.087 0.705 1.025 0.826 0.659 1.349 0.936 1.087 1.332 0.901 1.24 1.524 1.11 1.905 2.079 0.86 1.4 0.98 0.974 0.589 1.191 1.912 5.67

S. invicta 61 Argentina, Santa Fe Prov., San Justo 4 1.960 1.359 1.295 1.292 1.218 0.897 0.735 0.969 0.74 0.525 1.256 0.864 0.971 1.247 0.846 1.209 1.447 1.032 1.47 1.848 0.705 1.137 0.768 0.868 0.47 1.164 1.719 5.24

S. invicta 61 Argentina, Santa Fe Prov., San Justo 5 0.645 1.252 1.187 1.16 1.082 0.902 0.609 0.915 0.734 0.559 1.156 0.828 0.947 1.177 0.771 1.048 1.419 1.008 1.779 1.638 0.589 1.013 0.751 0.833 0.451 0.941 1.426 4.66

S. invicta 61 Argentina, Santa Fe Prov., San Justo 6 0.642 1.235 1.099 1.043 0.988 0.851 0.506 0.888 0.684 0.579 1.098 0.787 0.838 1.084 0.751 1.091 1.342 0.937 1.653 1.485 0.557 0.901 0.667 0.787 0.412 1.113 1.186 4.62

S. invicta 61 Argentina, Santa Fe Prov., San Justo 7 0.899 1.339 1.24 1.188 1.101 0.938 0.636 0.973 0.662 0.517 1.215 0.839 0.98 1.268 0.848 1.199 1.449 1.022 1.777 1.714 0.599 1.053 0.731 0.867 0.489 1.149 1.468 5.07

S. invicta 61 Argentina, Santa Fe Prov., San Justo 8 0.884 1.319 1.314 1.261 1.152 0.968 0.66 1.034 0.803 0.611 1.226 0.894 0.926 1.313 0.905 1.218 1.494 1.075 1.835 1.806 0.66 1.157 0.76 0.877 0.516 1.282 1.563 5.28

S. invicta 61 Argentina, Santa Fe Prov., San Justo 9 1.127 1.39 1.4 1.321 1.236 1.006 0.712 1.001 0.749 0.563 1.27 0.884 0.944 1.247 0.867 1.075 1.487 1.069 1.874 1.794 0.7 1.106 0.779 0.858 0.509 1.322 1.583 5.36

S. invicta 61 Argentina, Santa Fe Prov., San Justo 10 1.030 1.34 1.352 1.289 1.164 1.026 0.711 0.961 0.76 0.611 1.247 0.901 0.974 1.255 0.908 1.196 1.484 1.058 1.81 0.617 1.149 0.75 0.75 0.5 1.198 1.599 5.10

S. invicta 61 Argentina, Santa Fe Prov., San Justo 11 1.046 1.368 1.316 1.261 1.185 0.996 0.734 1.072 0.766 0.617 1.205 0.905 0.98 1.256 0.884 1.488 1.036 1.829 1.837 0.624 1.148 0.83 0.874 0.539 1.236 1.689 5.32

S. invicta 61 Argentina, Santa Fe Prov., San Justo 12 0.155 0.762 0.604 0.57 0.549 0.44 0.372 0.598 0.488 0.469 0.733 0.505 0.51 0.681 0.476 0.524 0.845 0.628 1.025 1.036 0.344 0.637 0.44 0.56 0.28 0.677 0.716 3.04

S. invicta 61 Argentina, Santa Fe Prov., San Justo 13 0.518 1.151 1.041 0.943 0.954 0.769 0.576 0.874 0.594 0.548 1.028 0.679 0.865 1.025 0.68 1.147 1.269 0.88 1.567 1.382 0.518 0.889 0.626 0.759 0.378 1.005 1.002 4.30

S. invicta 61 Argentina, Santa Fe Prov., San Justo 14 0.633 1.215 1.148 1.089 1.005 0.886 0.58 0.915 0.688 0.598 1.127 0.772 0.748 1.128 0.736 1.123 1.283 0.944 1.778 1.587 0.535 0.968 0.712 0.747 0.409 1.092 1.347 4.64

S. invicta 61 Argentina, Santa Fe Prov., San Justo 15 0.476 1.034 0.987 0.907 0.884 0.734 0.544 0.832 0.622 0.561 0.965 0.686 0.838 0.986 0.677 1.056 1.222 0.86 1.541 1.357 0.479 0.852 0.585 0.644 0.375 0.942 1.034 3.98

S. invicta 61 Argentina, Santa Fe Prov., San Justo 16 0.593 1.201 1.115 1.073 1.017 0.801 0.595 0.911 0.684 0.604 1.104 0.755 0.767 1.064 0.755 1.137 1.353 0.978 1.582 1.562 0.49 0.957 0.674 0.79 0.437 1.019 1.192 4.57

S. invicta 61 Argentina, Santa Fe Prov., San Justo 17 0.454 0.996 0.91 0.85 0.808 0.682 0.448 0.575 0.585 0.547 0.984 0.635 0.796 0.907 0.675 0.964 1.132 0.79 1.452 1.321 0.447 0.799 0.528 0.666 0.342 0.862 0.999 3.85

S. invicta 61 Argentina, Santa Fe Prov., San Justo 18 0.190 0.842 0.732 0.658 0.664 0.574 0.336 0.657 0.506 0.448 0.763 0.49 0.652 0.743 0.523 0.809 0.948 0.674 1.179 1.12 0.342 0.656 0.483 0.593 0.287 0.727 0.767 3.28

S. invicta 61 Argentina, Santa Fe Prov., San Justo 19 0.276 0.915 0.832 0.737 0.753 0.647 0.445 0.706 0.506 0.5 0.834 0.588 0.836 0.561 0.902 1.011 0.692 1.243 1.194 0.377 0.725 0.524 0.608 0.304 0.864 0.905 3.58

S. invicta 61 Argentina, Santa Fe Prov., San Justo 20 0.159 0.779 0.706 0.608 0.634 0.527 0.342 0.613 0.449 0.454 0.694 0.495 0.57 0.714 0.466 0.765 0.901 0.607 0 1.04 0.329 0.642 0.454 0.574 0.268 0.645 0.759 3.04

S. invicta 61 Argentina, Santa Fe Prov., San Justo 21 0.257 0.939 0.794 0.728 0.721 0.609 0.441 0.702 0.499 0.508 0.807 0.566 0.517 0.827 0.528 0.866 1.005 0.702 1.315 1.211 0.394 0.742 0.527 0.608 0.304 0.719 0.894 3.48

S. invicta 61 Argentina, Santa Fe Prov., San Justo 22 0.186 0.793 0.704 0.623 0.626 0.522 0.409 0.654 0.472 0.474 0.738 0.503 0.649 0.74 0.481 0.815 0.909 0.606 0 1.083 0.354 0.655 0.465 0.534 0.266 0.658 0.815 3.07

S. invicta 61 Argentina, Santa Fe Prov., San Justo 23 0.200 0.767 0.685 0.619 0.623 0.519 0.345 0.551 0.424 0.446 0.719 0.544 0.618 0.722 0.502 0.759 0.862 0.572 1.109 1.011 0.343 0.599 0.478 0.488 0.257 0.708 0.725 2.97

S. invicta 61 Argentina, Santa Fe Prov., San Justo 24 0.167 0.823 0.736 0.665 0.657 0.573 0.343 0.674 0.465 0.491 0.805 0.558 0.659 0.748 0.528 0.799 0.948 0.678 1.076 1.086 0.333 0.653 0.47 0.319 0.265 0.717 0.786 2.95

S. invicta 61 Argentina, Santa Fe Prov., San Justo 25 0.133 0.737 0.643 0.598 0.583 0.471 0.347 0.591 0.381 0.36 0.698 0.476 0.58 0.616 0.471 0.705 0.709 0.496 1.064 0.965 0.276 0.597 0.4 0.479 0.253 0.642 0.717 2.82

S. invicta 61 Argentina, Santa Fe Prov., San Justo 26 0.230 0.842 0.658 0.626 0.588 0.499 0.325 0.676 0.477 0.479 0.768 0.521 0.651 0.74 0.501 0.806 0.935 0.66 1.18 1.069 0.403 0.643 0.47 0.511 0.274 0.733 0.802 3.16

S. invicta 61 Argentina, Santa Fe Prov., San Justo 27 0.681 0.997 0.909 0.861 0.846 0.699 0.472 0.818 0.59 0.479 0.945 0.666 0.762 0.963 0.637 1.001 1.144 0.859 1.429 1.345 0.444 0.806 0.586 0.629 0.357 0.936 1.061 3.91

S. invicta 61 Argentina, Santa Fe Prov., San Justo 28 0.164 0.746 0.662 0.589 0.615 0.488 0.321 0.586 0.407 0.421 0.693 0.479 0.619 0.663 0.454 0.659 0.816 0.57 1.045 0.988 0.335 0.624 0.424 0.532 0.251 0.658 0.727 2.92

S. invicta 61 Argentina, Santa Fe Prov., San Justo 29 0.190 0.797 0.693 0.62 0.625 0.509 0.364 0.614 0.453 0.461 0.742 0.528 0.613 0.727 0.46 0.737 0.89 0.628 1.138 1.017 0.306 0.62 0.419 0.526 0.26 0.675 0.764 3.02

S. invicta 61 Argentina, Santa Fe Prov., San Justo 30 0.243 0.928 0.82 0.744 0.764 0.629 0.409 0.764 0.53 0.498 0.894 0.6 0.621 0.846 0.558 0.854 1.029 0.728 1.318 1.239 0.385 0.726 0.559 0.576 0.319 0.775 0.955 3.52

S. invicta 61 Argentina, Santa Fe Prov., San Justo 31 0.355 1.029 0.887 0.822 0.796 0.654 0.5 0.776 0.465 0.5 0.902 0.643 0.741 0.865 0.621 0.89 1.077 0.732 1.118 1.228 0.457 0.786 0.535 0.704 0.343 0.814 0.899 3.77

S. invicta 61 Argentina, Santa Fe Prov., San Justo 32 0.240 0.846 0.749 0.674 0.672 0.569 0.39 0.651 0.479 0.484 0.804 0.538 0.625 0.786 0.535 0.787 0.936 0.615 1.247 1.11 0.347 0.673 0.484 0.555 0.281 0.645 0.804 3.16

S. invicta 61 Argentina, Santa Fe Prov., San Justo 33 0.174 0.846 0.728 0.665 0.649 0.552 0.398 0.657 0.473 0.504 0.735 0.519 0.631 0.764 0.519 0.75 0.894 0.63 1.107 1.081 0.35 0.668 0.462 0.619 0.269 0.719 0.788 3.27

S. invicta 61 Argentina, Santa Fe Prov., San Justo 34 0.149 0.757 0.697 0.622 0.636 0.549 0.382 0.648 0.419 0.421 0.746 0.531 0.645 0.716 0.466 0.79 0.875 0.596 1.163 1.01 0.368 0.619 0.46 0.543 0.251 0.742 0.738 3.05

S. invicta 61 Argentina, Santa Fe Prov., San Justo 35 0.167 0.771 0.682 0.606 0.629 0.546 0.396 0.629 0.421 0.451 0.737 0.529 0.509 0.717 0.505 0.772 0.893 0.635 1.076 1.046 0.339 0.64 0.453 0.564 0.268 0.717 0.752 3.10

S. invicta 61 Argentina, Santa Fe Prov., San Justo 36

S. invicta 61 Argentina, Santa Fe Prov., San Justo 37

S. invicta 61 Argentina, Santa Fe Prov., San Justo 38

S. invicta 61 Argentina, Santa Fe Prov., San Justo 39

S. invicta 61 Argentina, Santa Fe Prov., San Justo 40

S. invicta 62 Argentina, Santa Fe Prov., San Justo 1 0.977 1.37 1.36 1.33 1.24 1.053 0.67 1.00 0.76 0.64 1.32 0.95 1.03 1.33 0.87 1.28 1.58 1.09 1.82 1.89 0.70 1.22 0.83 0.92 0.51 1.21 1.64 5.39

S. invicta 62 Argentina, Santa Fe Prov., San Justo 2 0.695 1.27 1.23 1.18 1.12 0.923 0.58 0.92 0.64 0.62 1.20 0.82 0.95 1.22 0.81 1.19 1.43 1.03 1.70 1.67 0.60 1.05 0.70 0.78 0.47 1.08 1.50 4.80

S. invicta 62 Argentina, Santa Fe Prov., San Justo 3 0.759 1.29 1.25 1.24 1.13 0.943 0.65 0.97 0.74 0.60 1.24 0.85 0.99 1.21 0.85 1.20 1.42 1.04 1.75 1.72 0.68 1.11 0.79 0.81 0.48 1.08 1.59 4.91

S. invicta 62 Argentina, Santa Fe Prov., San Justo 4 0.121 0.73 0.67 0.63 0.61 0.51 0.34 0.60 0.44 0.46 0.79 0.48 0.63 0.68 0.47 0.73 0.85 0.60 0.98 0.97 0.33 0.62 0.42 0.52 0.25 0.54 0.70 2.75

S. invicta 62 Argentina, Santa Fe Prov., San Justo 5 0.828 1.33 1.28 1.25 1.11 0.952 0.58 0.67 0.64 0.38 1.22 0.88 0.93 1.24 0.86 1.21 1.51 1.03 1.78 1.79 0.69 1.15 0.79 0.93 0.51 1.04 1.49 5.09

S. invicta 62 Argentina, Santa Fe Prov., San Justo 6 0.951 1.37 1.33 1.30 1.22 1.051 0.68 0.97 0.73 0.61 1.25 0.86 0.90 1.26 0.85 1.18 1.48 1.08 1.79 1.82 0.70 1.18 0.75 0.87 0.52 1.04 1.66 5.10

S. invicta 62 Argentina, Santa Fe Prov., San Justo 7 0.770 1.30 1.25 1.22 1.13 0.923 0.66 0.94 0.77 0.62 1.15 0.85 0.94 1.16 0.87 1.17 1.38 1.05 1.75 1.70 0.63 1.07 0.74 0.80 0.50 1.16 1.40 4.96

S. invicta 62 Argentina, Santa Fe Prov., San Justo 8 0.881 1.35 1.37 1.29 1.22 1.01 0.72 0.98 0.75 0.61 1.25 0.89 0.95 1.26 0.88 1.17 1.54 1.07 1.89 1.86 0.69 1.15 0.79 0.89 0.51 1.21 1.54 5.31

S. invicta 62 Argentina, Santa Fe Prov., San Justo 9 0.172 0.84 0.73 0.66 0.67 0.586 0.42 0.66 0.46 0.46 0.77 0.55 0.59 0.75 0.51 0.79 0.95 0.67 1.10 1.14 0.38 0.67 0.51 0.55 0.28 0.62 0.82 3.15

S. invicta 62 Argentina, Santa Fe Prov., San Justo 10 0.946 1.37 1.35 1.35 1.21 1.051 0.60 0.99 0.75 0.63 1.29 0.94 1.02 1.28 0.90 1.13 1.46 1.06 1.85 1.91 0.71 1.25 0.83 0.86 0.53 1.20 1.59 5.34

S. invicta 62 Argentina, Santa Fe Prov., San Justo 11 0.617 1.31 1.22 1.22 1.09 0.895 0.57 0.94 0.73 0.60 1.17 0.83 0.77 1.21 0.79 1.13 1.40 1.00 1.73 1.75 0.64 1.07 0.76 0.82 0.47 1.04 1.34 4.93

S. invicta 62 Argentina, Santa Fe Prov., San Justo 12 0.472 1.13 1.09 1.03 1.00 0.816 0.54 0.86 0.66 0.60 1.07 0.76 0.81 1.10 0.73 1.05 1.33 0.94 1.57 1.56 0.53 0.97 0.65 0.71 0.43 0.80 1.25 4.20

S. invicta 62 Argentina, Santa Fe Prov., San Justo 13 0.454 1.10 1.05 0.99 0.94 0.815 0.52 0.84 0.65 0.57 1.08 0.70 0.83 1.06 0.72 0.99 1.29 0.88 1.53 1.39 0.51 0.91 0.63 0.71 0.39 0.98 1.16 4.18

S. invicta 62 Argentina, Santa Fe Prov., San Justo 14 0.278 1.00 0.90 0.83 0.81 0.64 0.42 0.79 0.53 0.45 0.89 0.72 0.76 0.94 0.65 0.89 1.13 0.80 1.41 1.24 0.43 0.81 0.54 0.68 0.32 0.87 0.92 3.79

S. invicta 62 Argentina, Santa Fe Prov., San Justo 15 0.236 0.80 0.70 0.72 0.72 0.597 0.47 0.71 0.56 0.43 0.88 0.63 0.71 0.84 0.55 0.79 1.03 0.73 1.25 1.18 0.41 0.73 0.51 0.60 0.24 0.69 0.88 3.27

S. invicta 62 Argentina, Santa Fe Prov., San Justo 16 0.236 0.95 0.84 0.75 0.76 0.627 0.46 0.72 0.54 0.49 0.87 0.63 0.70 0.88 0.59 0.78 1.08 0.69 1.34 1.25 0.40 0.79 0.53 0.61 0.32 0.83 0.92 3.64

S. invicta 62 Argentina, Santa Fe Prov., San Justo 17 0.912 1.12 1.05 0.98 0.95 0.76 0.57 0.86 0.62 0.59 1.00 0.73 0.81 1.04 0.72 0.87 1.17 0.94 1.58 1.45 0.51 0.91 0.63 0.72 0.39 0.89 1.09 4.17

S. invicta 62 Argentina, Santa Fe Prov., San Justo 18 0.166 0.84 0.72 0.65 0.65 0.551 0.39 0.65 0.50 0.45 0.74 0.53 0.65 0.72 0.47 0.76 0.91 0.57 1.13 1.08 0.35 0.70 0.42 0.53 0.27 0.69 0.70 3.14

S. invicta 62 Argentina, Santa Fe Prov., San Justo 19 0.146 0.79 0.71 0.61 0.64 0.566 0.36 0.63 0.49 0.49 0.72 0.52 0.59 0.78 0.51 0.76 0.93 0.65 1.11 1.08 0.33 0.67 0.47 0.57 0.27 0.71 0.70 3.15

S. invicta 62 Argentina, Santa Fe Prov., San Justo 20 0.272 0.94 0.85 0.75 0.79 0.624 0.51 0.76 0.58 0.52 0.89 0.62 0.65 0.90 0.60 0.86 1.09 0.77 1.36 1.27 0.44 0.79 0.55 0.63 0.33 0.76 0.91 3.59

S. invicta 62 Argentina, Santa Fe Prov., San Justo 21 0.973 1.38 1.40 1.39 1.21 1.026 0.69 0.98 0.80 0.63 1.28 0.92 0.96 1.28 0.89 1.25 1.52 1.08 1.85 1.85 0.67 1.18 0.82 0.80 0.53 1.17 1.66 5.20

S. invicta 62 Argentina, Santa Fe Prov., San Justo 22 0.196 0.89 0.78 0.72 0.72 0.613 0.43 0.70 0.46 0.46 0.83 0.59 0.61 0.58 0.50 0.77 0.87 0.56 0.95 1.15 0.40 0.73 0.47 0.63 0.29 0.74 0.77 3.40

S. invicta 62 Argentina, Santa Fe Prov., San Justo 23 0.117 0.80 0.67 0.61 0.61 0.474 0.31 0.58 0.43 0.43 0.65 0.48 0.60 0.69 0.46 0.73 0.83 0.57 0.91 1.00 0.36 0.63 0.43 0.52 0.25 0.58 0.66 2.89

S. invicta 62 Argentina, Santa Fe Prov., San Justo 24 0.126 0.74 0.66 0.59 0.61 0.491 0.28 0.59 0.47 0.41 0.74 0.49 0.62 0.69 0.47 0.73 0.88 0.59 1.07 1.00 0.30 0.62 0.44 0.53 0.26 0.63 0.63 2.90

S. invicta 62 Argentina, Santa Fe Prov., San Justo 25 0.118 0.76 0.65 0.59 0.58 0.536 0.34 0.59 0.40 0.40 0.72 0.48 0.59 0.68 0.46 0.73 0.82 0.60 1.06 0.99 0.35 0.62 0.41 0.51 0.25 0.66 0.65 2.93

S. invicta 62 Argentina, Santa Fe Prov., San Justo 26 0.250 0.83 0.70 0.67 0.67 0.535 0.33 0.65 0.48 0.47 0.75 0.54 0.63 0.72 0.50 0.70 0.92 0.65 1.11 1.09 0.33 0.68 0.47 0.54 0.28 0.73 0.73 3.19

S. invicta 62 Argentina, Santa Fe Prov., San Justo 27 0.137 0.79 0.69 0.62 0.63 0.507 0.32 0.61 0.48 0.42 0.75 0.51 0.62 0.74 0.49 0.76 0.88 0.64 1.10 1.06 0.34 0.66 0.46 0.50 0.27 0.65 0.70 3.00

S. invicta 62 Argentina, Santa Fe Prov., San Justo 28 0.146 0.81 0.71 0.65 0.64 0.611 0.33 0.64 0.48 0.45 0.74 0.51 0.61 0.72 0.47 0.76 0.89 0.60 1.17 1.06 0.36 0.65 0.47 0.53 0.26 0.62 0.81 3.02

S. invicta 62 Argentina, Santa Fe Prov., San Justo 29 0.182 0.79 0.68 0.60 0.63 0.515 0.35 0.54 0.47 0.44 0.69 0.51 0.61 0.68 0.47 0.74 0.87 0.61 1.10 1.00 0.33 0.62 0.41 0.53 0.26 0.75 0.69 3.08

S. invicta 62 Argentina, Santa Fe Prov., San Justo 30 0.276 0.83 0.73 0.65 0.68 0.609 0.40 0.66 0.50 0.48 0.77 0.53 0.54 0.74 0.54 0.92 0.62 1.14 1.08 0.38 0.68 0.44 0.57 0.27 0.75 0.75 3.23

S. invicta 62 Argentina, Santa Fe Prov., San Justo 31 0.82 0.72 0.66 0.66 0.586 0.38 0.63 0.45 0.53 0.62 0.54 1.05 0.45 0.64 0.54 0.00 0.76 0.73 2.63

S. invicta 62 Argentina, Santa Fe Prov., San Justo 32 0.111 0.76 0.67 0.60 0.61 0.512 0.33 0.62 0.37 0.41 0.72 0.49 0.60 0.70 0.49 0.74 0.86 0.61 0.00 1.04 0.31 0.65 0.44 0.55 0.25 0.66 0.66 3.00

S. invicta 62 Argentina, Santa Fe Prov., San Justo 33 0.115 0.75 0.67 0.60 0.60 0.505 0.34 0.60 0.45 0.45 0.69 0.49 0.46 0.67 0.49 0.58 0.83 0.62 1.03 1.06 0.34 0.69 0.44 0.51 0.25 0.59 0.67 2.91

S. invicta 62 Argentina, Santa Fe Prov., San Justo 34 0.186

S. invicta 62 Argentina, Santa Fe Prov., San Justo 35 0.215 0.89 0.81 0.74 0.75 0.629 0.38 0.69 0.55 0.48 0.88 0.59 0.70 0.84 0.60 0.73 1.07 0.74 1.30 1.19 0.40 0.74 0.51 0.55 0.31 0.80 0.84 3.44

S. invicta 62 Argentina, Santa Fe Prov., San Justo 36 0.110 0.72 0.64 0.58 0.55 0.485 0.27 0.56 0.43 0.42 0.65 0.46 0.58 0.63 0.43 0.68 0.79 0.51 1.06 1.00 0.35 0.60 0.43 0.52 0.25 0.61 0.68 2.84

S. invicta 62 Argentina, Santa Fe Prov., San Justo 37 0.811 1.36 1.32 1.29 1.17 0.974 0.65 0.97 0.71 0.61 1.24 0.90 0.94 1.25 0.88 1.19 1.50 1.02 1.87 1.83 0.69 1.17 0.81 0.83 0.48 1.25 1.50 5.27

S. invicta 62 Argentina, Santa Fe Prov., San Justo 38

S. invicta 62 Argentina, Santa Fe Prov., San Justo 39

S. invicta 62 Argentina, Santa Fe Prov., San Justo 40

S. amblychila 65 AZ, Santa Cruz, Yanks Canyon 1 0.750 1.212 1.257 1.252 1.118 0.855 0.544 0.738 0.635 0.554 0.915 0.660 0.820 0.860 0.656 0.988 1.008 0.776 1.499 1.533 0.615 1.030 0.620 0.873 0.458 1.408 1.196 5.03

S. amblychila 65 AZ, Santa Cruz, Yanks Canyon 2 0.820 1.185 1.270 1.298 1.141 0.916 0.644 0.751 0.602 0.524 0.883 0.716 0.814 0.855 0.637 0.965 1.019 0.792 1.461 1.692 0.693 1.093 0.726 0.895 0.461 1.274 1.240 5.05

S. amblychila 65 AZ, Santa Cruz, Yanks Canyon 3 0.790 1.214 1.282 1.287 1.146 0.964 0.649 0.799 0.643 0.537 0.907 0.716 0.852 0.883 0.671 0.942 1.042 0.849 1.511 1.576 0.585 1.030 0.745 0.849 0.436 1.272 1.197 4.91

S. amblychila 65 AZ, Santa Cruz, Yanks Canyon 4 0.800 1.253 1.313 1.308 1.163 0.944 0.693 0.776 0.635 0.573 0.940 0.719 0.873 0.858 0.671 0.996 1.041 0.842 1.553 1.626 0.615 1.041 0.742 0.898 0.494 1.352 1.207 5.13

S. amblychila 65 AZ, Santa Cruz, Yanks Canyon 5 0.750 1.232 1.281 1.258 1.126 0.906 0.686 0.775 0.626 0.548 0.884 0.719 0.821 0.912 0.686 0.962 1.009 0.803 1.493 1.707 0.617 1.127 0.661 0.886 0.480 1.338 1.164 5.16

S. amblychila 65 AZ, Santa Cruz, Yanks Canyon 6 0.790 1.179 1.269 1.253 1.119 0.885 0.661 0.776 0.615 0.555 0.883 0.715 0.685 0.884 0.662 0.845 0.987 0.823 1.469 1.604 0.618 1.031 0.661 0.830 0.461 1.263 1.151 4.88

S. amblychila 65 AZ, Santa Cruz, Yanks Canyon 7 0.600 1.164 1.196 1.151 1.096 0.875 0.635 0.738 0.548 0.496 0.943 0.662 0.760 0.834 0.664 0.883 0.943 0.751 1.385 1.524 0.559 0.965 0.640 0.800 0.437 1.363 1.140 4.85

S. amblychila 65 AZ, Santa Cruz, Yanks Canyon 8 0.740 1.090 1.170 1.118 1.062 0.831 0.599 0.693 0.593 0.543 0.838 0.673 0.839 0.799 0.694 0.885 0.973 0.822 1.383 1.524 0.583 0.997 0.633 0.815 0.425 1.185 1.096 4.61

S. amblychila 65 AZ, Santa Cruz, Yanks Canyon 9 0.610 1.162 1.229 1.196 1.095 0.889 0.633 0.773 0.603 0.543 0.903 0.750 0.800 0.856 0.648 0.938 1.030 0.822 1.448 1.576 0.616 0.979 0.683 0.873 0.419 1.151 1.113 4.76

S. amblychila 65 AZ, Santa Cruz, Yanks Canyon 10 0.850 1.172 1.257 1.222 1.119 0.903 0.646 0.772 0.533 0.531 0.878 0.730 0.805 0.866 0.631 0.982 1.031 0.765 1.459 1.583 0.603 1.020 0.665 0.859 0.450 1.274 1.174 4.89

S. amblychila 65 AZ, Santa Cruz, Yanks Canyon 11 0.480 1.023 1.050 1.006 0.983 0.822 0.518 0.714 0.489 0.514 0.792 0.616 0.762 0.859 0.555 0.856 0.906 0.738 1.349 1.334 0.525 0.894 0.477 0.775 0.357 0.933 0.924 4.07

S. amblychila 65 AZ, Santa Cruz, Yanks Canyon 12 0.210 0.788 0.755 0.704 0.687 0.587 0.421 0.591 0.398 0.466 0.664 0.488 0.579 0.647 0.441 0.674 0.694 0.534 1.026 0.976 0.369 0.610 0.426 0.545 0.262 0.866 0.683 3.18

S. amblychila 65 AZ, Santa Cruz, Yanks Canyon 13 0.360 0.963 0.929 0.904 0.859 0.700 0.503 0.641 0.468 0.465 0.717 0.539 0.659 0.697 0.535 0.763 0.854 0.652 1.200 1.207 0.440 0.747 0.527 0.681 0.340 1.251 0.813 4.10

S. amblychila 65 AZ, Santa Cruz, Yanks Canyon 14 0.400 1.168 1.017 0.990 0.926 0.714 0.501 0.685 0.468 0.502 0.749 0.608 0.717 0.820 0.550 0.843 0.896 0.678 1.278 1.342 0.517 0.816 0.550 0.691 0.350 0.970 0.914 4.17

S. amblychila 65 AZ, Santa Cruz, Yanks Canyon 15 0.170 0.703 0.644 0.588 0.601 0.514 0.359 0.530 0.317 0.459 0.540 0.407 0.594 0.520 0.386 0.614 0.628 0.516 0.919 0.932 0.353 0.585 0.379 0.502 0.229 0.656 0.652 2.79

S. amblychila 65 AZ, Santa Cruz, Yanks Canyon 16 0.120 0.708 0.646 0.607 0.615 0.524 0.377 0.538 0.368 0.402 0.563 0.422 0.541 0.490 0.396 0.578 0.614 0.485 0.914 0.921 0.329 0.576 0.404 0.501 0.245 0.752 0.591 2.88

S. amblychila 65 AZ, Santa Cruz, Yanks Canyon 17 0.250 0.864 0.819 0.786 0.764 0.597 0.421 0.633 0.441 0.482 0.667 0.540 0.652 0.632 0.510 0.724 0.760 0.649 1.120 1.153 0.418 0.719 0.477 0.602 0.290 1.115 0.753 3.73

S. amblychila 65 AZ, Santa Cruz, Yanks Canyon 18 0.130 0.696 0.619 0.574 0.576 0.468 0.306 0.507 0.359 0.416 0.506 0.416 0.509 0.469 0.368 0.579 0.581 0.452 0.894 0.876 0.340 0.562 0.352 0.488 0.224 0.702 0.680 2.76

S. amblychila 65 AZ, Santa Cruz, Yanks Canyon 19 0.130 0.758 0.696 0.652 0.65 0.586 0.418 0.552 0.362 0.468 0.685 0.490 0.523 0.625 0.435 0.619 0.732 0.530 1.010 1.015 0.346 0.677 0.421 0.557 0.268 1.003 0.702 3.33

S. amblychila 65 AZ, Santa Cruz, Yanks Canyon 20 0.170 0.742 0.665 0.627 0.609 0.507 0.347 0.532 0.393 0.418 0.559 0.440 0.540 0.591 0.382 0.626 0.649 0.485 0.938 0.958 0.379 0.596 0.398 0.507 0.234 0.669 0.597 2.88

S. amblychila 65 AZ, Santa Cruz, Yanks Canyon 21 0.140 0.704 0.642 0.592 0.594 0.535 0.379 0.518 0.363 0.413 0.534 0.422 0.547 0.530 0.378 0.613 0.625 0.489 0.902 0.931 0.334 0.576 0.393 0.574 0.223 0.719 0.663 2.93

S. amblychila 65 AZ, Santa Cruz, Yanks Canyon 22 0.560 1.094 1.100 1.096 0.989 0.782 0.514 0.723 0.466 0.587 0.837 0.616 0.778 0.796 0.613 0.868 0.938 0.698 1.307 1.445 0.624 0.883 0.638 0.781 0.404 1.237 1.036 4.56

S. amblychila 65 AZ, Santa Cruz, Yanks Canyon 23 0.220 0.808 0.730 0.657 0.696 0.552 0.359 0.571 0.418 0.458 0.684 0.474 0.587 0.569 0.429 0.712 0.784 0.560 1.042 1.031 0.402 0.659 0.446 0.585 0.289 0.805 0.718 3.23

S. amblychila 65 AZ, Santa Cruz, Yanks Canyon 24 0.230 0.761 0.690 0.627 0.645 0.537 0.394 0.568 0.396 0.427 0.571 0.468 0.578 0.485 0.446 0.619 0.658 0.524 0.984 0.964 0.385 0.610 0.374 0.505 0.247 0.764 0.635 2.99

S. amblychila 65 AZ, Santa Cruz, Yanks Canyon 25 0.370 1.003 1.026 0.982 0.929 0.736 0.489 0.710 0.527 0.482 0.778 0.608 0.717 0.719 0.553 0.775 0.883 0.708 1.270 1.281 0.490 0.796 0.564 0.669 0.359 1.059 0.869 4.01

S. amblychila 65 AZ, Santa Cruz, Yanks Canyon 26 0.240 0.826 0.776 0.720 0.715 0.620 0.425 0.609 0.418 0.474 0.630 0.502 0.586 0.596 0.460 0.669 0.729 0.565 1.051 1.051 0.430 0.652 0.430 0.632 0.282 0.876 0.775 3.39

S. amblychila 65 AZ, Santa Cruz, Yanks Canyon 27 0.170 0.698 0.624 0.579 0.604 0.475 0.288 0.564 0.385 0.388 0.527 0.400 0.541 0.584 0.356 0.596 0.696 0.485 0.897 0.876 0.313 0.564 0.359 0.496 0.228 0.647 0.574 2.72

S. amblychila 65 AZ, Santa Cruz, Yanks Canyon 28 0.120 0.747 0.669 0.624 0.624 0.507 0.322 0.560 0.388 0.424 0.586 0.441 0.569 0.592 0.423 0.620 0.700 0.534 0.958 0.938 0.329 0.590 0.410 0.540 0.240 0.899 0.635 3.12

S. amblychila 65 AZ, Santa Cruz, Yanks Canyon 29 0.140 0.754 0.620 0.565 0.574 0.493 0.342 0.557 0.375 0.390 0.589 0.422 0.503 0.557 0.374 0.591 0.604 0.485 0.920 0.865 0.318 0.561 0.358 0.482 0.233 0.695 0.582 2.80

S. amblychila 65 AZ, Santa Cruz, Yanks Canyon 30 0.160 0.721 0.631 0.620 0.608 0.521 0.350 0.526 0.356 0.448 0.551 0.451 0.520 0.540 0.391 0.647 0.625 0.497 0.942 0.930 0.335 0.577 0.382 0.521 0.247 0.698 0.603 2.87

S. amblychila 65 AZ, Santa Cruz, Yanks Canyon 31 0.150 0.686 0.607 0.563 0.571 0.485 0.311 0.508 0.369 0.423 0.607 0.409 0.486 0.561 0.351 0.546 0.582 0.485 0.804 0.886 0.359 0.575 0.350 0.507 0.229 0.682 0.620 2.76

S. amblychila 65 AZ, Santa Cruz, Yanks Canyon 32 0.080 0.652 0.541 0.513 0.529 0.440 0.300 0.457 0.296 0.393 0.468 0.393 0.470 0.414 0.329 0.519 0.525 0.411 0.803 0.803 0.323 0.505 0.335 0.463 0.213 0.524 0.535 2.44

S. amblychila 65 AZ, Santa Cruz, Yanks Canyon 33 0.070 0.624 0.597 0.568 0.54 0.446 0.295 0.475 0.318 0.396 0.479 0.400 0.461 0.454 0.332 0.532 0.535 0.436 0.813 0.819 0.329 0.528 0.349 0.485 0.229 0.591 0.557 2.52

S. amblychila 65 AZ, Santa Cruz, Yanks Canyon 34 0.090 0.709 0.608 0.561 0.579 0.442 0.320 0.508 0.368 0.402 0.588 0.425 0.484 0.483 0.374 0.556 0.602 0.477 0.875 0.897 0.330 0.556 0.398 0.528 0.233 0.635 0.597 2.77

S. amblychila 65 AZ, Santa Cruz, Yanks Canyon 35 0.100 0.719 0.574 0.518 0.529 0.435 0.312 0.470 0.334 0.374 0.481 0.390 0.448 0.448 0.346 0.541 0.630 0.422 0.841 0.824 0.306 0.520 0.340 0.449 0.218 0.637 0.570 2.63

S. amblychila 65 AZ, Santa Cruz, Yanks Canyon 36 0.080 0.676 0.586 0.552 0.568 0.463 0.323 0.497 0.357 0.396 0.568 0.400 0.500 0.538 0.371 0.538 0.608 0.442 0.889 0.865 0.312 0.536 0.363 0.440 0.218 0.653 0.552 2.63

S. amblychila 65 AZ, Santa Cruz, Yanks Canyon 37 0.090 0.664 0.603 0.564 0.561 0.470 0.321 0.473 0.352 0.403 0.569 0.408 0.483 0.513 0.362 0.552 0.660 0.459 0.858 0.839 0.314 0.547 0.331 0.522 0.225 0.652 0.607 2.68

S. amblychila 65 AZ, Santa Cruz, Yanks Canyon 38 0.070 0.612 0.551 0.494 0.507 0.410 0.271 0.511 0.314 0.373 0.540 0.376 0.463 0.411 0.347 0.520 0.535 0.453 0.797 0.785 0.269 0.502 0.337 0.430 0.212 0.574 0.518 2.40

S. amblychila 65 AZ, Santa Cruz, Yanks Canyon 39 0.060 0.657 0.574 0.513 0.532 0.462 0.333 0.533 0.344 0.380 0.591 0.396 0.492 0.563 0.357 0.556 0.579 0.423 0.896 0.842 0.324 0.519 0.338 0.442 0.224 0.680 0.568 2.62

S. amblychila 65 AZ, Santa Cruz, Yanks Canyon 40 0.050 0.635 0.540 0.496 0.515 0.435 0.298 0.462 0.307 0.388 0.468 0.383 0.467 0.480 0.340 0.511 0.624 0.418 0.812 0.842 0.294 0.530 0.354 0.495 0.204 0.597 0.535 2.57

S. amblychila 66 AZ, Santa Cruz, Yanks Canyon 1 0.800 1.278 1.317 1.317 1.171 1.014 0.636 0.773 0.636 0.539 0.948 0.730 0.808 0.891 0.674 0.970 1.080 0.796 1.510 1.664 0.623 1.032 0.754 0.849 0.463 1.284 1.300 5.08

S. amblychila 66 AZ, Santa Cruz, Yanks Canyon 2 0.840 1.267 1.339 1.377 1.165 0.949 0.566 0.811 0.616 0.553 1.670 0.610 1.072 0.716 0.893 0.493 0.918 0.695 0.837 0.660 0.997 1.072 0.834 1.538 1.675 1.366 4.67

S. amblychila 66 AZ, Santa Cruz, Yanks Canyon 3 0.960 1.289 1.344 1.388 1.171 0.948 0.567 0.805 0.628 0.571 0.965 0.750 0.897 0.876 0.735 1.030 1.083 0.910 1.589 1.752 0.704 1.085 0.769 0.923 0.495 1.378 1.344 5.34

S. amblychila 66 AZ, Santa Cruz, Yanks Canyon 4 0.860 1.283 1.337 1.347 1.187 0.958 0.614 0.816 0.623 0.540 0.963 0.700 0.879 0.920 0.675 1.059 1.107 0.864 1.542 1.686 0.687 1.069 0.725 0.894 0.497 1.377 1.311 5.24

S. amblychila 66 AZ, Santa Cruz, Yanks Canyon 5 0.930 1.280 1.269 1.273 1.133 0.906 0.563 0.775 0.617 0.562 0.957 0.733 0.807 0.937 0.692 1.004 1.098 0.848 1.537 1.647 0.687 1.035 0.739 0.877 0.444 1.267 1.256 5.07

S. amblychila 66 AZ, Santa Cruz, Yanks Canyon 6 0.910 1.303 1.352 1.394 1.201 1.000 0.587 0.816 0.584 0.546 0.952 0.722 0.871 0.904 0.698 0.834 1.105 0.856 1.510 1.664 0.694 1.094 0.734 0.871 0.468 1.245 1.289 5.08

S. amblychila 66 AZ, Santa Cruz, Yanks Canyon 7 0.830 1.256 1.234 1.223 1.159 0.938 0.556 0.742 0.612 0.542 0.966 0.750 0.854 0.917 0.670 0.982 1.085 0.848 1.477 1.612 0.616 1.005 0.741 0.805 0.454 1.333 1.278 5.01

S. amblychila 66 AZ, Santa Cruz, Yanks Canyon 8 0.740 1.245 1.267 1.290 1.116 0.893 0.567 0.768 0.620 0.536 0.913 0.749 0.791 0.887 0.697 0.982 1.080 0.830 1.468 1.566 0.660 0.981 0.710 0.853 0.439 1.146 1.267 4.81

S. amblychila 66 AZ, Santa Cruz, Yanks Canyon 9 0.760 1.192 1.185 1.197 1.096 0.939 0.570 0.748 0.595 0.526 0.915 0.692 0.829 0.849 0.720 0.937 1.058 0.843 1.488 1.557 0.657 0.953 0.690 0.828 0.432 1.257 1.223 4.83

S. amblychila 66 AZ, Santa Cruz, Yanks Canyon 10 0.810 1.257 1.284 1.296 1.183 0.860 0.560 0.801 0.651 0.542 0.931 0.772 0.834 0.926 0.706 1.060 1.080 0.883 1.524 1.647 0.720 1.048 0.745 0.888 0.477 1.289 1.289 5.08

S. amblychila 66 AZ, Santa Cruz, Yanks Canyon 11 0.740 1.246 1.236 1.259 1.13 0.915 0.581 0.761 0.587 0.522 0.885 0.725 0.800 0.882 0.662 0.926 1.037 0.832 1.423 1.610 0.555 0.967 0.706 0.839 0.499 1.313 1.168 5.01

S. amblychila 66 AZ, Santa Cruz, Yanks Canyon 12 0.370 0.959 0.895 0.841 0.843 0.739 0.431 0.670 0.459 0.496 0.742 0.567 0.645 0.706 0.518 0.776 0.843 0.653 1.181 1.221 0.415 0.774 0.512 0.641 0.326 0.926 0.826 3.75

S. amblychila 66 AZ, Santa Cruz, Yanks Canyon 13 0.550 1.135 1.121 1.116 1.015 0.805 0.546 0.740 0.525 0.560 0.871 0.648 0.755 0.841 0.618 0.849 0.980 0.770 1.385 1.444 0.444 0.877 0.607 0.786 0.401 1.069 1.124 4.43

S. amblychila 66 AZ, Santa Cruz, Yanks Canyon 14 0.150 0.728 0.672 0.610 0.616 0.534 0.303 0.553 0.415 0.394 0.566 0.444 0.564 0.535 0.402 0.627 0.656 0.507 0.967 0.947 0.298 0.578 0.369 0.540 0.244 0.788 0.623 3.00

S. amblychila 66 AZ, Santa Cruz, Yanks Canyon 15 0.780 1.257 1.257 1.263 1.136 0.973 0.599 0.799 0.617 0.507 0.966 0.739 0.840 0.877 0.683 0.951 1.007 0.787 1.476 1.633 0.666 1.024 0.715 0.871 0.475 1.312 0.970 5.07

S. amblychila 66 AZ, Santa Cruz, Yanks Canyon 16 0.510 1.082 1.059 1.043 0.962 0.832 0.489 0.728 0.552 0.518 0.818 0.627 0.767 0.786 0.560 0.839 1.015 0.738 1.310 1.332 0.507 0.860 0.499 0.758 0.395 1.048 1.003 4.22

S. amblychila 66 AZ, Santa Cruz, Yanks Canyon 17 0.150 0.782 0.689 0.645 0.652 0.569 0.320 0.584 0.410 0.431 0.596 0.430 0.615 0.595 0.428 0.665 0.745 0.535 1.048 0.988 0.371 0.615 0.413 0.525 0.251 0.750 0.639 3.04

S. amblychila 66 AZ, Santa Cruz, Yanks Canyon 18 0.210 0.884 0.790 0.760 0.733 0.661 0.411 0.654 0.447 0.476 0.688 0.490 0.628 0.647 0.476 0.746 0.796 0.621 1.175 1.093 0.419 0.694 0.424 0.618 0.289 0.951 0.750 3.55

S. amblychila 66 AZ, Santa Cruz, Yanks Canyon 19 0.790 1.251 1.301 1.325 1.147 0.976 0.588 0.793 0.607 0.545 0.993 0.689 0.834 0.890 0.675 0.921 1.078 0.823 1.444 1.575 0.621 1.022 0.641 0.869 0.485 1.282 1.264 4.98

S. amblychila 66 AZ, Santa Cruz, Yanks Canyon 20 0.520 1.147 1.102 1.086 0.976 0.804 0.553 0.734 0.576 0.510 0.871 0.646 0.753 0.834 0.611 0.862 0.970 0.756 1.362 1.425 0.524 0.906 0.617 0.773 0.422 1.138 1.029 4.48

S. amblychila 66 AZ, Santa Cruz, Yanks Canyon 21 0.500 1.118 1.086 1.075 0.978 0.832 0.541 0.744 0.551 0.525 0.849 0.620 0.763 0.810 0.575 0.882 0.982 0.742 1.330 1.422 0.566 0.867 0.580 0.786 0.398 1.135 1.047 4.46

S. amblychila 66 AZ, Santa Cruz, Yanks Canyon 22 0.330 0.931 0.882 0.865 0.813 0.684 0.430 0.667 0.507 0.483 0.697 0.540 0.645 0.683 0.507 0.772 0.826 0.631 1.213 1.201 0.437 0.764 0.504 0.684 0.342 0.937 0.860 3.75

S. amblychila 66 AZ, Santa Cruz, Yanks Canyon 23 0.320 0.959 0.920 0.882 0.841 0.667 0.408 0.680 0.464 0.497 0.744 0.563 0.662 0.705 0.506 0.783 0.857 0.688 1.219 1.201 0.444 0.754 0.520 0.669 0.331 1.015 0.811 3.84

S. amblychila 66 AZ, Santa Cruz, Yanks Canyon 24 0.640 1.235 1.179 1.190 1.072 0.937 0.564 0.773 0.590 0.524 0.864 0.667 0.761 0.860 0.652 0.923 1.040 0.789 1.458 1.547 0.543 0.968 0.647 0.821 0.441 1.147 1.146 4.75

S. amblychila 66 AZ, Santa Cruz, Yanks Canyon 25 0.840 1.269 1.270 1.283 1.133 0.950 0.578 0.800 0.546 0.585 0.941 0.701 0.832 0.901 0.659 0.927 1.069 0.806 1.510 1.645 0.663 1.063 0.684 0.904 0.480 1.336 1.290 5.15

S. amblychila 66 AZ, Santa Cruz, Yanks Canyon 26 0.220 0.835 0.779 0.726 0.717 0.613 0.365 0.603 0.431 0.466 0.652 0.494 0.607 0.605 0.454 0.714 0.849 0.580 1.147 1.073 0.375 0.692 0.409 0.603 0.300 1.124 0.837 3.64

S. amblychila 66 AZ, Santa Cruz, Yanks Canyon 27 0.310 0.981 0.904 0.860 0.836 0.737 0.414 0.682 0.518 0.484 0.756 0.561 0.677 0.821 0.507 0.785 0.842 0.662 1.213 1.167 0.386 0.761 0.463 0.675 0.343 1.325 0.926 4.15

S. amblychila 66 AZ, Santa Cruz, Yanks Canyon 28 0.220 0.887 0.817 0.730 0.755 0.685 0.438 0.617 0.422 0.492 0.659 0.507 0.617 0.612 0.470 0.714 0.801 0.595 1.135 1.101 0.364 0.705 0.444 0.610 0.300 0.871 0.760 3.47

S. amblychila 66 AZ, Santa Cruz, Yanks Canyon 29 0.100 0.649 0.561 0.524 0.531 0.445 0.281 0.463 0.342 0.366 0.506 0.347 0.495 0.514 0.347 0.560 0.625 0.421 0.865 0.804 0.259 0.524 0.303 0.478 0.213 0.645 0.568 2.58

S. amblychila 66 AZ, Santa Cruz, Yanks Canyon 30 0.130 0.723 0.630 0.587 0.59 0.506 0.332 0.524 0.386 0.384 0.610 0.397 0.546 0.506 0.376 0.589 0.690 0.496 0.934 0.905 0.332 0.572 0.373 0.513 0.249 0.739 0.639 2.88

S. amblychila 66 AZ, Santa Cruz, Yanks Canyon 31 0.170 0.802 0.696 0.658 0.663 0.553 0.350 0.591 0.408 0.458 0.620 0.458 0.595 0.562 0.391 0.696 0.745 0.519 1.027 1.003 0.376 0.628 0.408 0.581 0.263 1.016 0.723 3.40

S. amblychila 66 AZ, Santa Cruz, Yanks Canyon 32 0.140 0.739 0.651 0.596 0.619 0.574 0.326 0.529 0.376 0.441 0.564 0.404 0.595 0.533 0.414 0.602 0.656 0.513 0.959 0.948 0.324 0.595 0.400 0.549 0.259 0.755 0.683 2.99

S. amblychila 66 AZ, Santa Cruz, Yanks Canyon 33 0.090 0.700 0.617 0.574 0.58 0.508 0.321 0.525 0.360 0.386 0.548 0.417 0.552 0.618 0.401 0.535 0.610 0.469 0.899 0.881 0.336 0.575 0.333 0.524 0.232 0.668 0.590 2.77

S. amblychila 66 AZ, Santa Cruz, Yanks Canyon 34 0.070 0.678 0.579 0.551 0.552 0.441 0.281 0.531 0.354 0.386 0.521 0.404 0.495 0.567 0.362 0.575 0.649 0.457 0.909 0.855 0.292 0.546 0.330 0.495 0.223 0.694 0.621 2.72

S. amblychila 66 AZ, Santa Cruz, Yanks Canyon 35 0.100 0.690 0.413 0.507 0.56 0.507 0.297 0.513 0.349 0.397 0.514 0.351 0.542 0.502 0.368 0.573 0.601 0.474 0.921 0.855 0.325 0.566 0.326 0.525 0.229 0.689 0.551 2.76

S. amblychila 66 AZ, Santa Cruz, Yanks Canyon 36 0.080 0.681 0.574 0.541 0.545 0.450 0.254 0.507 0.358 0.384 0.536 0.386 0.517 0.488 0.351 0.582 0.571 0.483 0.855 0.835 0.326 0.536 0.337 0.530 0.226 0.800 0.568 2.85

S. amblychila 66 AZ, Santa Cruz, Yanks Canyon 37 0.100 0.672 0.590 0.534 0.569 0.516 0.312 0.520 0.332 0.410 0.530 0.404 0.516 0.435 0.369 0.582 0.590 0.475 0.871 0.863 0.320 0.537 0.363 0.518 0.224 0.703 0.542 2.76

S. amblychila 66 AZ, Santa Cruz, Yanks Canyon 38 0.060 0.683 0.595 0.562 0.555 0.483 0.299 0.540 0.365 0.387 0.553 0.390 0.492 0.481 0.371 0.571 0.612 0.452 0.866 0.838 0.298 0.547 0.340 0.492 0.211 0.695 0.551 2.71

S. amblychila 66 AZ, Santa Cruz, Yanks Canyon 39 0.050 0.571 0.495 0.457 0.452 0.395 0.264 0.401 0.276 0.353 0.442 0.301 0.396 0.441 0.278 0.413 0.474 0.336 0.665 0.741 0.160 0.489 0.288 0.412 0.197 0.551 0.452 2.28

S. amblychila 66 AZ, Santa Cruz, Yanks Canyon 40 0.040 0.608 0.503 0.470 0.48 0.352 0.239 0.468 0.305 0.351 0.442 0.342 0.447 0.380 0.298 0.447 0.540 0.363 0.694 0.788 0.259 0.503 0.293 0.450 0.199 0.608 0.485 2.45

S. xyloni 68 AZ, Tempe 1 1.120 1.33 1.341 1.33 1.193 1.015 0.645 0.95 0.711 0.576 1.128 0.797 0.959 1.09 0.755 1.2 1.289 0.953 1.738 1.699 0.615 1.099 0.706 0.867 0.501 1.022 1.294 4.92

S. xyloni 68 AZ, Tempe 2 0.840 1.267 1.24 1.236 1.161 1.009 0.559 0.863 0.667 0.614 1.087 0.759 0.644 1.035 0.736 1.066 1.272 0.933 1.653 1.668 0.636 1.045 0.707 0.821 0.451 1.027 1.346 4.78

S. xyloni 68 AZ, Tempe 3 0.810 1.288 1.245 1.203 1.135 0.955 0.592 0.853 0.676 0.559 1.05 0.716 0.893 1.075 0.689 1.087 1.266 0.877 1.687 1.568 0.581 1.043 0.666 0.785 0.452 1.019 1.219 4.66

S. xyloni 68 AZ, Tempe 4 0.850 1.351 1.314 1.288 1.209 1.062 0.594 0.808 0.704 0.59 1.089 0.796 0.881 1.056 0.749 1.113 1.262 0.904 1.684 1.675 0.622 1.095 0.697 0.839 0.502 1.055 1.273 4.92

S. xyloni 68 AZ, Tempe 5 0.680 1.231 1.199 1.184 1.093 0.873 0.575 0.844 0.66 0.552 1.016 0.74 0.872 1.004 0.717 1.062 1.171 0.869 1.533 1.546 0.582 0.989 0.663 0.751 0.425 0.944 1.157 4.47

S. xyloni 68 AZ, Tempe 6 0.710 1.293 1.256 1.268 1.148 1.019 0.617 0.83 0.668 0.602 1.048 0.763 0.9 1.019 0.738 1.045 1.225 0.891 1.595 1.599 0.666 1.008 0.666 0.833 0.441 1.045 1.163 4.77

S. xyloni 68 AZ, Tempe 7 0.800 1.315 1.251 1.249 1.129 0.976 0.607 0.86 0.642 0.587 1.052 0.752 0.851 1.073 0.708 1.05 1.237 0.908 1.601 1.623 0.65 1.016 0.666 0.769 0.434 0.988 1.168 4.70

S. xyloni 68 AZ, Tempe 8 0.770 1.304 1.289 1.257 1.154 0.972 0.56 0.883 0.71 0.576 1.094 0.71 0.871 1.087 0.726 1.099 1.3 0.936 1.647 1.643 0.661 1.051 0.673 0.827 0.463 1.009 1.216 4.78

S. xyloni 68 AZ, Tempe 9 1.170 1.172 1.284 1.278 1.162 0.936 0.566 0.836 0.708 0.587 1.066 0.752 0.897 1.066 0.733 1.123 1.236 0.897 1.663 1.648 0.639 1.047 0.701 0.788 0.466 1.103 1.242 4.71

S. xyloni 68 AZ, Tempe 10 0.580 1.196 1.145 1.124 1.036 0.845 0.571 0.802 0.644 0.574 1.03 0.737 0.826 0.987 0.734 0.994 1.228 0.855 1.528 1.478 0.541 0.929 0.633 0.754 0.403 0.865 1.04 4.29

S. xyloni 68 AZ, Tempe 11 0.550 1.168 1.148 1.121 1.041 0.866 0.558 0.804 0.638 0.553 1.012 0.712 0.86 1.01 0.685 1.024 1.216 0.869 1.598 1.532 0.6 0.981 0.642 0.786 0.412 0.993 1.077 4.48

S. xyloni 68 AZ, Tempe 12 0.140 0.739 0.67 0.617 0.628 0.574 0.349 0.576 0.396 0.449 0.676 0.424 0.59 0.602 0.433 0.692 0.767 0.539 1.021 0.968 0.332 0.591 0.407 0.507 0.242 0.628 0.612 2.84

S. xyloni 68 AZ, Tempe 13 0.170 0.725 0.657 0.616 0.606 0.556 0.326 0.591 0.434 0.454 0.662 0.479 0.608 0.674 0.427 0.662 0.748 0.53 1.041 0.963 0.37 0.6 0.39 0.525 0.254 0.592 0.612 2.81

S. xyloni 68 AZ, Tempe 14 0.280 0.919 0.855 0.814 0.807 0.71 0.408 0.699 0.519 0.525 0.808 0.582 0.686 0.75 0.549 0.8 0.957 0.664 1.255 1.177 0.419 0.739 0.505 0.637 0.331 0.702 0.713 3.44

S. xyloni 68 AZ, Tempe 15 0.750 1.22 1.185 1.144 1.046 0.899 0.517 0.847 0.659 0.561 1.023 0.718 0.882 1.004 0.72 1.029 1.233 0.902 1.54 1.511 0.575 0.944 0.658 0.741 0.434 0.962 1.098 4.43

S. xyloni 68 AZ, Tempe 16 0.630 1.224 1.161 1.14 1.035 0.868 0.566 0.839 0.584 0.564 0.995 0.74 0.857 0.981 0.684 1.068 1.162 0.849 1.582 1.502 0.54 0.951 0.633 0.747 0.419 0.95 1.14 4.42

S. xyloni 68 AZ, Tempe 17 0.170 0.769 0.743 0.706 0.689 0.602 0.407 0.628 0.469 0.491 0.714 0.476 0.708 0.66 0.482 0.719 0.81 0.613 1.142 1.042 0.355 0.65 0.433 0.593 0.265 0.705 0.689 3.11

S. xyloni 68 AZ, Tempe 18 0.150 0.755 0.681 0.623 0.629 0.559 0.319 0.567 0.419 0.439 0.664 0.432 0.55 0.591 0.418 0.646 0.782 0.544 0.987 0.969 0.348 0.605 0.42 0.503 0.242 0.646 0.593 2.87

S. xyloni 68 AZ, Tempe 19 0.130 0.708 0.665 0.618 0.624 0.532 0.31 0.559 0.409 0.426 0.669 0.442 0.567 0.624 0.415 0.654 0.755 0.548 0.985 0.966 0.322 0.604 0.392 0.544 0.237 0.608 0.56 2.83

S. xyloni 68 AZ, Tempe 20 0.110 0.723 0.67 0.623 0.613 0.549 0.396 0.422 0.449 0.449 0.666 0.433 0.585 0.418 0.418 0.757 0.757 0.554 1.009 0.929 0.308 0.589 0.376 0.502 0.255 0.633 0.586 2.79

S. xyloni 68 AZ, Tempe 21 0.160 0.708 0.676 0.602 0.624 0.546 0.328 0.569 0.428 0.431 0.658 0.432 0.597 0.559 0.422 0.661 0.761 0.54 1.036 0.96 0.308 0.58 0.394 0.465 0.243 0.592 0.575 2.73

S. xyloni 68 AZ, Tempe 22 0.320 0.956 0.893 0.845 0.845 0.704 0.445 0.717 0.578 0.507 0.844 0.572 0.693 0.806 0.524 0.883 0.93 0.707 1.349 1.18 0.454 0.748 0.5 0.615 0.328 0.761 0.826 3.51

S. xyloni 68 AZ, Tempe 23 0.360 0.952 0.967 0.924 0.891 0.775 0.408 0.756 0.528 0.504 0.887 0.629 0.736 0.856 0.555 0.884 0.999 0.733 1.282 1.274 0.47 0.818 0.529 0.648 0.338 0.797 0.955 3.67

S. xyloni 68 AZ, Tempe 24 0.130 0.744 0.66 0.606 0.611 0.582 0.302 0.566 0.396 0.44 0.649 0.448 0.545 0.587 0.402 0.578 0.734 0.52 0.986 0.943 0.328 0.59 0.387 0.504 0.242 0.602 0.607 2.79

S. xyloni 68 AZ, Tempe 25 0.160 0.745 0.682 0.63 0.628 0.531 0.357 0.57 0.412 0.432 0.649 0.479 0.564 0.622 0.419 0.697 0.758 0.541 1.003 0.964 0.359 0.606 0.409 0.5 0.244 0.632 0.584 2.84

S. xyloni 68 AZ, Tempe 26 0.180 0.739 0.681 0.623 0.639 0.543 0.323 0.596 0.406 0.432 0.666 0.454 0.6 0.598 0.42 0.659 0.753 0.571 1.046 0.983 0.302 0.615 0.393 0.521 0.25 0.602 0.644 2.85

S. xyloni 68 AZ, Tempe 27 0.120 0.734 0.679 0.617 0.607 0.545 0.329 0.576 0.405 0.436 0.635 0.436 0.54 0.603 0.406 0.644 0.762 0.528 0.982 0.936 0.317 0.589 0.387 0.487 0.252 0.665 0.612 2.82

S. xyloni 68 AZ, Tempe 28 0.420 1.084 1.031 0.995 0.935 0.825 0.452 0.768 0.566 0.5 0.895 0.681 0.758 0.892 0.607 0.954 1.069 0.808 1.388 1.361 0.491 0.847 0.584 0.671 0.365 0.831 0.938 3.95

S. xyloni 68 AZ, Tempe 29 0.160 0.718 0.665 0.589 0.607 0.546 0.348 0.588 0.401 0.414 0.659 0.425 0.552 0.565 0.362 0.623 0.745 0.505 0.966 0.958 0.343 0.602 0.382 0.518 0.231 0.592 0.582 2.79

S. xyloni 68 AZ, Tempe 30 0.160 0.75 0.689 0.64 0.643 0.589 0.331 0.575 0.429 0.444 0.681 0.447 0.616 0.632 0.424 0.677 0.836 0.565 1.077 0.997 0.345 0.632 0.408 0.547 0.251 0.643 0.647 2.94

S. xyloni 68 AZ, Tempe 31 0.130 0.759 0.662 0.628 0.634 0.578 0.319 0.596 0.444 0.444 0.595 0.476 0.615 0.651 0.424 0.708 0.791 0.554 1.024 0.974 0.308 0.618 0.395 0.513 0.261 0.628 0.575 2.87

S. xyloni 68 AZ, Tempe 32 0.120 0.76 0.681 0.639 0.623 0.55 0.329 0.582 0.449 0.471 0.662 0.444 0.599 0.616 0.454 0.66 0.77 0.555 1.039 0.955 0.343 0.591 0.413 0.518 0.248 0.592 0.607 2.83

S. xyloni 68 AZ, Tempe 33 0.100 0.762 0.662 0.629 0.615 0.511 0.291 0.512 0.422 0.415 0.664 0.469 0.588 0.606 0.443 0.648 0.75 0.55 1.011 0.934 0.328 0.585 0.39 0.495 0.249 0.569 0.542 2.76

S. xyloni 68 AZ, Tempe 34 0.120 0.734 0.639 0.596 0.554 0.516 0.329 0.561 0.393 0.423 0.638 0.439 0.553 0.583 0.401 0.63 0.734 0.491 0.961 0.91 0.327 0.597 0.388 0.482 0.245 0.607 0.612 2.73

S. xyloni 68 AZ, Tempe 35 0.100 0.723 0.628 0.587 0.576 0.56 0.349 0.478 0.417 0.423 0.649 0.454 0.533 0.581 0.423 0.637 0.713 0.535 0.99 0.913 0.344 0.584 0.39 0.484 0.232 0.644 0.549 2.76

S. xyloni 68 AZ, Tempe 36 0.090 0.718 0.612 0.554 0.565 0.52 0.308 0.55 0.407 0.423 0.564 0.448 0.54 0.534 0.386 0.62 0.676 0.507 0.934 0.884 0.301 0.556 0.359 0.48 0.219 0.56 0.518 2.64

S. xyloni 68 AZ, Tempe 37 0.110 0.739 0.637 0.57 0.612 0.539 0.351 0.545 0.415 0.423 0.684 0.43 0.546 0.604 0.406 0.635 0.686 0.49 0.951 0.907 0.309 0.589 0.38 0.484 0.233 0.64 0.56 2.77

S. xyloni 68 AZ, Tempe 38 0.090 0.699 0.628 0.572 0.594 0.555 0.294 0.547 0.402 0.391 0.607 0.386 0.546 0.571 0.358 0.648 0.673 0.493 0.951 0.888 0.303 0.563 0.37 0.445 0.225 0.553 0.542 2.59

S. xyloni 68 AZ, Tempe 39 0.080 0.613 0.573 0.53 0.521 0.488 0.296 0.501 0.366 0.401 0.551 0.391 0.528 0.447 0.35 0.574 0.612 0.445 0.852 0.8 0.271 0.507 0.337 0.413 0.216 0.544 0.569 2.37

S. xyloni 68 AZ, Tempe 40 0.070 0.668 0.598 0.545 0.558 0.519 0.277 0.522 0.371 0.416 0.614 0.431 0.515 0.539 0.4 0.628 0.658 0.49 0.717 0.862 0.25 0.543 0.361 0.498 0.222 0.614 0.51 2.64

S. gayi 69 Peru, Lima 1 0.515 1.141 1.134 1.075 1.072 0.792 0.509 0.843 0.633 0.521 1.105 0.755 0.825 1.006 0.714 0.997 1.262 0.896 1.433 1.458 0.501 0.899 0.592 0.862 0.374 0.960 1.121 4.42

S. gayi 69 Peru, Lima 2 0.618 1.231 1.225 1.187 1.158 0.96 0.657 0.878 0.694 0.584 1.001 0.805 0.737 1.095 0.773 1.040 1.365 1.038 1.599 1.582 0.543 0.981 0.709 0.793 0.429 1.058 1.308 4.66

S. gayi 69 Peru, Lima 3 0.553 1.149 1.149 1.117 1.059 0.817 0.517 0.773 0.603 0.560 1.006 0.771 0.743 0.924 0.695 1.026 1.178 0.720 1.413 1.496 0.497 0.934 0.660 0.742 0.398 1.092 1.155 4.48

S. gayi 69 Peru, Lima 4 0.464 1.142 1.124 1.077 1.057 0.823 0.575 0.844 0.633 0.534 1.038 0.715 0.792 1.052 0.750 0.947 1.261 0.890 1.454 1.478 0.576 0.934 0.655 0.779 0.385 1.066 1.142 4.46

S. gayi 69 Peru, Lima 5 0.454 1.125 1.088 1.013 1.011 0.778 0.505 0.805 0.601 0.505 1.034 0.728 0.786 0.972 0.711 0.935 1.186 0.881 1.438 1.412 0.503 0.869 0.616 0.718 0.377 1.049 1.014 4.30

S. gayi 69 Peru, Lima 6 0.543 1.183 1.160 1.123 1.074 0.811 0.552 0.826 0.626 0.549 1.016 0.756 0.830 0.995 0.754 0.971 1.250 0.884 1.481 1.483 0.534 0.907 0.633 0.741 0.395 1.029 1.125 4.44

S. gayi 69 Peru, Lima 7 0.718 1.161 1.214 1.173 1.110 0.87 0.596 0.905 0.613 0.618 1.074 0.793 0.887 1.095 0.771 0.982 1.299 0.958 1.521 1.560 0.573 0.968 0.680 0.789 0.427 0.993 1.297 4.50

S. gayi 69 Peru, Lima 8 0.570 1.190 1.200 1.153 1.115 0.855 0.553 0.866 0.661 0.571 1.069 0.790 0.839 1.059 0.775 1.012 1.282 0.931 1.559 1.551 0.642 0.960 0.688 0.762 0.407 1.103 1.175 4.61

S. gayi 69 Peru, Lima 9 0.597 1.172 1.193 1.143 1.118 0.906 0.563 0.885 0.655 0.571 1.102 0.786 0.777 1.072 0.731 1.071 1.312 0.977 1.596 1.560 0.552 0.981 0.674 0.832 0.424 1.089 1.235 4.65

S. gayi 69 Peru, Lima 10 0.604 1.195 1.249 1.218 1.165 0.893 0.598 0.891 0.698 0.559 1.064 0.780 0.828 1.078 0.742 0.987 1.295 0.937 1.476 1.581 0.623 0.972 0.708 0.827 0.419 0.977 1.215 4.58

S. gayi 69 Peru, Lima 11 0.385 1.079 1.045 0.990 0.964 0.79 0.469 0.798 0.601 0.546 1.017 0.691 0.793 0.991 0.650 0.983 1.125 0.851 1.455 1.376 0.520 0.854 0.592 0.690 0.360 0.815 1.018 3.96

S. gayi 69 Peru, Lima 12 0.559 1.132 1.174 1.142 1.074 0.861 0.543 0.874 0.662 0.545 1.010 0.767 0.751 1.021 0.743 1.023 1.276 0.958 1.500 1.483 0.553 0.941 0.638 0.770 0.396 0.980 1.195 4.37

S. gayi 69 Peru, Lima 13 0.481 1.123 1.120 1.047 1.046 0.827 0.503 0.839 0.608 0.554 0.980 0.783 0.813 1.026 0.690 0.969 1.255 0.912 1.513 1.446 0.522 0.894 0.664 0.719 0.371 1.010 1.162 4.30

S. gayi 69 Peru, Lima 14 0.490 1.083 1.118 1.054 1.041 0.836 0.530 0.850 0.609 0.520 1.022 0.747 0.823 1.014 0.706 0.932 1.232 0.887 1.476 1.443 0.542 0.920 0.643 0.762 0.393 0.977 1.206 4.26

S. gayi 69 Peru, Lima 15 0.550 1.187 1.169 1.138 1.081 0.879 0.532 0.859 0.627 0.585 1.091 0.796 0.838 1.024 0.775 1.059 1.314 0.936 1.524 1.606 0.629 0.971 0.733 0.744 0.410 1.054 1.258 4.59

S. gayi 69 Peru, Lima 16 0.578 1.177 1.163 1.131 1.099 0.86 0.523 0.866 0.644 0.588 1.119 0.774 0.816 1.035 0.770 1.044 1.300 0.925 1.525 1.578 0.633 0.965 0.717 0.802 0.417 1.016 1.274 4.57

S. gayi 69 Peru, Lima 17 0.593 1.191 1.212 1.164 1.137 0.95 0.610 0.807 0.649 0.573 1.049 0.774 0.767 1.049 0.732 1.007 1.339 0.914 1.559 1.532 0.582 0.981 0.638 0.769 0.409 1.077 1.243 4.57

S. gayi 69 Peru, Lima 18 0.466 1.112 1.102 1.038 1.031 0.801 0.490 0.831 0.616 0.539 1.050 0.717 0.683 1.001 0.711 0.930 1.216 0.858 1.470 1.448 0.558 0.920 0.647 0.782 0.373 0.928 1.142 4.27

S. gayi 69 Peru, Lima 19 0.492 1.114 1.135 1.101 1.066 0.845 0.510 0.848 0.671 0.572 1.050 0.772 0.810 1.022 0.714 1.033 1.283 0.898 1.544 1.489 0.557 0.909 0.671 0.784 0.390 1.072 1.168 4.46

S. gayi 69 Peru, Lima 20 0.522 1.177 1.143 1.107 1.058 0.862 0.531 0.858 0.618 0.543 1.055 0.709 0.799 1.048 0.722 0.999 1.198 0.917 1.541 1.493 0.489 0.940 0.646 0.764 0.389 1.042 1.135 4.48

S. gayi 69 Peru, Lima 21 0.487 1.051 1.025 0.971 0.955 0.77 0.488 0.821 0.569 0.517 0.953 0.677 0.794 0.955 0.657 0.868 1.154 0.838 1.367 1.321 0.500 0.836 0.574 0.727 0.352 0.945 0.988 4.04

S. gayi 69 Peru, Lima 22 0.358 0.995 0.959 0.902 0.898 0.727 0.471 0.754 0.548 0.518 0.947 0.645 0.739 0.901 0.614 0.871 1.081 0.767 1.301 1.314 0.486 0.822 0.577 0.704 0.339 0.889 0.938 3.90

S. gayi 69 Peru, Lima 23 0.233 0.883 0.820 0.754 0.781 0.605 0.386 0.647 0.480 0.419 0.794 0.594 0.607 0.767 0.532 0.760 0.972 0.695 1.171 1.153 0.415 0.698 0.493 0.599 0.291 0.736 0.775 3.37

S. gayi 69 Peru, Lima 24 0.263 0.907 0.868 0.801 0.814 0.66 0.401 0.671 0.514 0.463 0.839 0.595 0.684 0.775 0.537 0.784 0.955 0.691 1.239 1.171 0.398 0.735 0.506 0.640 0.297 0.847 0.854 3.57

S. gayi 69 Peru, Lima 25 0.294 0.940 0.922 0.869 0.866 0.671 0.410 0.620 0.521 0.475 0.870 0.600 0.642 0.818 0.576 0.782 1.043 0.723 1.241 1.283 0.446 0.784 0.553 0.620 0.314 0.866 0.849 3.71

S. gayi 69 Peru, Lima 26 0.306 0.947 0.922 0.871 0.838 0.676 0.409 0.679 0.533 0.518 0.888 0.609 0.614 0.843 0.571 0.834 1.062 0.748 1.285 1.225 0.474 0.766 0.538 0.656 0.320 0.899 0.917 3.73

S. gayi 69 Peru, Lima 27 0.372 1.034 0.995 0.958 0.906 0.724 0.428 0.758 0.566 0.516 0.952 0.672 0.717 0.935 0.656 0.868 1.120 0.817 1.304 1.338 0.495 0.837 0.606 0.698 0.335 1.015 1.019 4.09

S. gayi 69 Peru, Lima 28 0.313 0.983 0.924 0.874 0.872 0.673 0.371 0.733 0.522 0.506 0.803 0.592 0.735 0.821 0.576 0.867 1.067 0.758 1.208 1.260 0.432 0.775 0.513 0.703 0.324 0.874 0.826 3.82

S. gayi 69 Peru, Lima 29 0.223 0.841 0.798 0.751 0.761 0.604 0.377 0.635 0.495 0.471 0.788 0.543 0.613 0.745 0.512 0.741 0.931 0.645 1.153 1.136 0.438 0.700 0.506 0.605 0.289 0.747 0.794 3.33

S. gayi 69 Peru, Lima 30 0.228 0.853 0.806 0.767 0.741 0.613 0.368 0.620 0.486 0.440 0.761 0.544 0.695 0.756 0.524 0.728 0.919 0.644 1.144 1.113 0.401 0.685 0.498 0.579 0.274 0.764 0.804 3.31

S. gayi 69 Peru, Lima 31 0.252 0.915 0.882 0.825 0.826 0.649 0.374 0.696 0.534 0.478 0.852 0.606 0.667 0.803 0.567 0.815 1.017 0.682 1.230 1.210 0.383 0.717 0.520 0.609 0.304 0.780 0.815 3.51

S. gayi 69 Peru, Lima 32 0.319 0.861 0.829 0.782 0.796 0.734 0.411 0.653 0.533 0.473 0.824 0.575 0.622 0.795 0.559 0.749 0.926 0.614 1.211 1.140 0.363 0.706 0.485 0.604 0.296 0.728 0.808 3.33

S. gayi 69 Peru, Lima 33 0.230 0.863 0.810 0.759 0.769 0.636 0.387 0.668 0.500 0.466 0.769 0.538 0.653 0.750 0.530 0.751 0.894 0.658 1.045 1.128 0.378 0.700 0.474 0.642 0.291 0.742 0.860 3.38

S. gayi 69 Peru, Lima 34 0.204 0.851 0.784 0.724 0.744 0.599 0.382 0.640 0.488 0.451 0.801 0.559 0.665 0.743 0.488 0.676 0.962 0.655 1.129 1.104 0.367 0.692 0.470 0.561 0.259 0.683 0.721 3.20

S. gayi 69 Peru, Lima 35 0.350 0.944 0.947 0.900 0.899 0.764 0.473 0.765 0.612 0.487 0.942 0.666 0.742 0.852 0.627 0.848 1.101 0.789 1.281 1.300 0.465 0.801 0.585 0.668 0.319 0.903 0.848 3.82

S. gayi 69 Peru, Lima 36 0.177 0.794 0.770 0.718 0.724 0.583 0.385 0.641 0.463 0.440 0.774 0.514 0.596 0.694 0.480 0.670 0.886 0.617 0.999 1.042 0.371 0.655 0.312 0.602 0.267 0.734 0.738 3.17

S. gayi 69 Peru, Lima 37 0.224 0.867 0.831 0.803 0.803 0.596 0.375 0.623 0.415 0.448 0.783 0.568 0.674 0.748 0.515 0.723 0.919 0.691 1.119 1.137 0.393 0.704 0.488 0.622 0.294 0.624 0.766 3.25

S. gayi 69 Peru, Lima 38 0.203 0.847 0.788 0.744 0.742 0.588 0.379 0.633 0.406 0.418 0.790 0.541 0.663 0.737 0.563 0.741 0.988 0.636 0.932 1.099 0.279 0.677 0.471 0.587 0.284 0.765 0.771 3.30

S. gayi 69 Peru, Lima 39 0.304 0.988 0.938 0.870 0.882 0.709 0.479 0.708 0.544 0.513 0.893 0.610 0.638 0.865 0.607 0.847 1.058 0.740 1.283 1.242 0.438 0.771 0.548 0.667 0.329 0.874 0.936 3.77

S. gayi 69 Peru, Lima 40 0.135 0.712 0.689 0.635 0.646 0.566 0.357 0.557 0.376 0.408 0.382 0.320 0.533 0.640 0.442 0.631 0.765 0.537 0.955 0.984 0.339 0.627 0.395 0.526 0.234 0.657 0.628 2.88

S. interrupta 70 Argentina, Salta, RT 51 1 0.522 1.119 1.075 1.025 0.979 0.8 0.553 0.647 0.654 0.59 1.053 0.741 0.688 1.044 0.719 1.024 1.248 0.921 1.497 1.505 0.54 0.915 0.66 0.814 0.393 1.015 1.164 4.45

S. interrupta 70 Argentina, Salta, RT 51 2 0.593 1.129 1.074 1.032 0.971 0.799 0.576 0.824 0.668 0.584 1.075 0.746 0.857 1.115 0.756 1.267 0.864 1.636 1.564 0.539 0.985 0.687 0.82 0.381 0.926 1.199 4.44

S. interrupta 70 Argentina, Salta, RT 51 3 0.603 1.186 1.16 1.13 1.059 0.811 0.595 0.85 0.656 0.597 1.086 0.777 0.874 1.109 0.725 1.056 1.292 0.935 1.551 0.621 0.978 0.67 0.876 0.337 1.037 1.181 4.65

S. interrupta 70 Argentina, Salta, RT 51 4 0.665 1.247 1.199 1.178 1.102 0.893 0.636 0.949 0.723 0.662 1.205 0.81 0.942 1.134 0.799 1.098 1.357 0.939 1.709 1.653 0.624 1.032 0.728 0.792 0.435 1.155 1.29 4.85

S. interrupta 70 Argentina, Salta, RT 51 5 0.597 1.196 1.148 1.109 1.056 0.873 0.582 0.867 0.656 0.598 1.115 0.755 0.874 1.092 0.708 1.054 1.268 0.889 1.618 1.618 0.602 0.999 0.696 0.748 0.389 0.903 1.194 4.46

S. interrupta 70 Argentina, Salta, RT 51 6 0.627 1.199 1.157 1.093 1.087 0.86 0.616 0.951 0.661 0.611 1.153 0.82 0.695 1.129 0.826 1.16 1.304 0.974 1.72 1.666 0.607 1.069 0.702 0.758 0.407 0.934 1.267 4.56

S. interrupta 70 Argentina, Salta, RT 51 7 0.599 1.215 1.175 1.143 1.074 0.865 0.605 0.786 0.756 0.633 1.065 0.769 0.917 1.309 0.888 1.661 1.325 0.893 1.628 1.637 0.578 0.998 0.727 0.826 0.418 0.922 1.258 4.60

S. interrupta 70 Argentina, Salta, RT 51 8 0.622 1.201 1.141 1.125 1.05 0.862 0.594 0.908 0.699 0.596 1.125 0.826 1.148 0.818 1.342 0.964 1.7 1.565 0.608 1.026 0.699 0.796 0.413 1.041 1.207 4.60

S. interrupta 70 Argentina, Salta, RT 51 9 0.622 1.25 1.186 1.157 1.097 0.867 0.589 0.88 0.703 0.605 1.112 0.769 0.935 1.098 0.734 1.104 1.33 0.924 1.477 1.605 0.643 0.986 0.707 0.754 0.412 1.138 1.308 4.75

S. interrupta 70 Argentina, Salta, RT 51 10 0.547 1.13 1.053 0.991 0.97 0.839 0.507 0.877 0.644 0.589 1.042 0.756 0.754 1.044 0.776 1.269 0.884 1.613 1.566 0.568 0.949 0.669 0.818 0.338 0.986 1.117 4.50

S. interrupta 70 Argentina, Salta, RT 51 11 0.374 1.026 0.961 0.917 0.855 0.706 0.529 0.813 0.569 0.539 0.973 0.703 0.778 0.929 0.67 0.945 1.129 0.815 1.475 1.371 0.49 0.837 0.592 0.762 0.318 0.894 1.018 4.05

S. interrupta 70 Argentina, Salta, RT 51 12 0.476

S. interrupta 70 Argentina, Salta, RT 51 13 0.133 0.743 0.664 0.616 0.608 0.522 0.302 0.608 0.475 0.465 0.679 0.508 0.615 0.642 0.434 0.693 0.804 0.595 0.961 0.984 0.355 0.611 0.421 0.527 0.235 0.612 0.647 2.87

S. interrupta 70 Argentina, Salta, RT 51 14 0.131 0.731 0.679 0.619 0.619 0.543 0.343 0.614 0.444 0.459 0.721 0.492 0.607 0.662 0.471 0.708 0.818 0.608 0 0.976 0.394 0.631 0.443 0.468 0.24 0.547 0.666 2.72

S. interrupta 70 Argentina, Salta, RT 51 15 0.288 0.986 0.943 0.902 0.867 0.723 0.456 0.738 0.551 0.554 0.792 0.699 0.674 0.885 0.635 0.974 1.072 0.775 1.419 1.335 0.506 0.818 0.558 0.636 0.32 0.909 1.029 3.87

S. interrupta 70 Argentina, Salta, RT 51 16 0.501 1.122 1.037 1.01 0.98 0.803 0.545 0.841 0.665 0.606 1.018 0.758 0.852 0.852 0.733 0.995 1.223 0.875 1.564 1.507 0.55 0.901 0.668 0.772 0.403 0.965 1.135 4.37

S. interrupta 70 Argentina, Salta, RT 51 17 0.449 1.098 1.036 0.953 0.929 0.815 0.507 0.606 0.628 0.542 0.981 0.714 0.793 0.952 0.691 1.14 0.861 1.495 1.415 0.538 0.866 0.611 0.688 0.368 0.956 1.141 4.16

S. interrupta 70 Argentina, Salta, RT 51 18 0.418 1.062 0.977 0.952 0.894 0.706 0.547 0.863 0.627 0.584 1.001 0.708 0.852 0.971 0.682 0.835 1.189 0.856 1.566 1.44 0.503 0.897 0.623 0.817 0.364 0.898 1.1 4.22

S. interrupta 70 Argentina, Salta, RT 51 19 0.133 0.737 0.661 0.612 0.61 0.529 0.305 0.569 0.445 0.428 0.693 0.5 0.601 0.679 0.44 0.575 0.794 0.574 1.037 1.048 0.415 0.662 0.423 0.509 0.229 0.612 0.701 2.91

S. interrupta 70 Argentina, Salta, RT 51 20 0.126 0.712 0.635 0.588 0.594 0.504 0.315 0.571 0.469 0.464 0.602 0.457 0.609 0.661 0.431 0.676 0.79 0.562 1.037 0.959 0.345 0.587 0.411 0.463 0.233 0.585 0.721 2.72

S. interrupta 70 Argentina, Salta, RT 51 21 0.185 0.864 0.78 0.731 0.736 0.575 0.38 0.675 0.521 0.477 0.812 0.573 0.624 0.769 0.532 0.781 0.936 0.654 1.278 1.144 0.437 0.702 0.49 0.594 0.297 0.728 0.791 3.33

S. interrupta 70 Argentina, Salta, RT 51 22 0.134 0.738 0.669 0.61 0.637 0.535 0.345 0.63 0.355 0.446 0.698 0.457 0.579 0.644 0.484 0.769 0.771 0.539 1.004 1.029 0.364 0.629 0.445 0.597 0.241 0.702 0.61 3.07

S. interrupta 70 Argentina, Salta, RT 51 23 0.111 0.71 0.633 0.578 0.586 0.512 0.33 0.483 0.428 0.441 0.624 0.434 0.57 0.623 0.434 0.68 0.758 0.505 0.97 0.933 0.321 0.603 0.387 0.528 0.246 0.54 0.669 2.71

S. interrupta 70 Argentina, Salta, RT 51 24 0.106 0.748 0.634 0.582 0.608 0.515 0.358 0.631 0.434 0.432 0.69 0.497 0.588 0.556 0.468 0.822 0.583 1.128 0.953 0.312 0.594 0.42 0.507 0.254 0.475 0.68 2.68

S. interrupta 70 Argentina, Salta, RT 51 25 0.122 0.732 0.615 0.578 0.574 0.47 0.323 0.515 0.427 0.446 0.671 0.465 0.576 0.648 0.436 0.725 0.798 0.551 1.012 0.985 0.293 0.592 0.422 0.501 0.232 0.578 0.649 2.80

S. interrupta 70 Argentina, Salta, RT 51 26 0.089 0.696 0.604 0.589 0.567 0.48 0.281 0.555 0.424 0.419 0.624 0.456 0.493 0.524 0.395 0.656 0.535 0.992 0.901 0.34 0.586 0.374 0.461 0.218 0.562 0.643 2.62

S. interrupta 70 Argentina, Salta, RT 51 27 0.088 0.694 0.606 0.553 0.538 0.461 0.224 0.497 0.392 0.396 0.618 0.42 0.455 0.573 0.398 0.624 0.734 0.5 0 0.903 0.318 0.568 0.38 0.489 0.209 0.48 0.612 2.57

S. interrupta 70 Argentina, Salta, RT 51 28 0.127 0.725 0.635 0.599 0.577 0.498 0.301 0.557 0.422 0.423 0.641 0.47 0.546 0.658 0.455 0.667 0.802 0.571 1.034 0.951 0.312 0.612 0.391 0.512 0.24 0.515 0.658 2.70

S. interrupta 70 Argentina, Salta, RT 51 29 0.586 1.199 1.165 1.158 1.018 0.866 0.584 0.949 0.727 0.624 1.102 0.771 0.909 1.086 0.795 1.183 1.138 0.965 1.749 1.633 0.613 1.001 0.732 0.901 0.425 1.004 1.271 4.74

S. interrupta 70 Argentina, Salta, RT 51 30 0.464 0.965 0.895 0.827 0.834 0.671 0.424 0.757 0.535 0.474 0.911 0.663 0.733 0.881 0.597 0.915 1.093 0.79 1.433 1.308 0.448 0.795 0.577 0.69 0.325 0.946 0.888 3.91

S. interrupta 70 Argentina, Salta, RT 51 31 0.287 1.168 1.154 1.139 1.014 0.807 0.554 0.877 0.686 0.459 1.107 0.799 0.789 1.096 0.789 1.089 1.305 0.955 1.648 1.645 0.575 1.006 0.717 0.856 0.428 1.116 1.281 4.79

S. interrupta 70 Argentina, Salta, RT 51 32 0.615 1.171 1.095 1.039 1.018 0.819 0.558 0.92 0.719 0.55 1.076 0.753 0.946 1.084 0.458 1.316 0.957 1.586 1.567 0.575 0.958 0.676 0.878 0.391 0.846 1.241 4.46

S. interrupta 70 Argentina, Salta, RT 51 33 0.576 1.082 1.033 0.977 0.935 0.762 0.482 0.758 0.607 0.548 1 0.752 0.786 0.977 0.695 1.024 1.155 0.851 1.416 1.413 0.542 0.9 0.629 0.795 0.368 0.931 1.096 4.22

S. interrupta 70 Argentina, Salta, RT 51 34 0.432

S. interrupta 70 Argentina, Salta, RT 51 35 0.118

S. interrupta 70 Argentina, Salta, RT 51 36 0.110 0.522

S. interrupta 70 Argentina, Salta, RT 51 37 0.131

S. interrupta 70 Argentina, Salta, RT 51 38 0.110 0.703 0.644 0.582 0.598 0.522 0.342 0.563 0.413 0.429 0.638 0.439 0.531 0.634 0.417 0.551 0.75 0.521 0.97 0.912 0.327 0.593 0.408 0.492 0.613 0.649 2.72

S. interrupta 70 Argentina, Salta, RT 51 39 0.194

S. interrupta 70 Argentina, Salta, RT 51 40 0.111

S. macdonaghi 71 Argentina, Entre Rios, Jtc. Rts. 40, 130 1 1.526 1.466 1.631 1.573 1.455 1.434 0.779 1.09 0.79 0.68 1.365 0.978 0.932 1.334 0.913 1.294 1.588 1.143 2.013 2.073 0.789 1.288 0.882 0.945 0.595 1.369 1.776 5.85

S. macdonaghi 71 Argentina, Entre Rios, Jtc. Rts. 40, 130 2 1.470 1.462 1.517 1.531 1.317 1.33 0.814 0.999 0.78 0.61 1.307 0.906 1.061 1.334 0.892 1.312 1.58 1.098 1.802 1.981 0.818 1.253 0.866 0.903 0.605 1.262 1.728 5.61

S. macdonaghi 71 Argentina, Entre Rios, Jtc. Rts. 40, 130 3 1.030 1.326 1.324 1.317 1.168 1.186 0.586 0.942 0.672 0.618 1.205 0.871 0.953 1.225 0.837 1.441 1.034 1.827 1.763 0.64 1.108 0.775 0.786 0.506 1.105 1.634 4.98

S. macdonaghi 71 Argentina, Entre Rios, Jtc. Rts. 40, 130 4 1.143 1.353 1.397 1.379 1.216 1.211 0.644 0.988 0.707 0.62 1.268 0.844 0.938 1.23 0.832 1.295 1.425 1.003 1.776 1.883 0.831 1.171 0.843 0.909 0.555 1.243 1.628 5.39

S. macdonaghi 71 Argentina, Entre Rios, Jtc. Rts. 40, 130 5 1.493 1.45 1.54 1.515 1.321 1.354 0.775 0.959 0.778 0.626 1.297 0.945 1.093 1.258 0.877 1.5 1.098 1.87 2.012 0.776 1.277 0.845 0.893 0.571 1.162 1.891 5.52

S. macdonaghi 71 Argentina, Entre Rios, Jtc. Rts. 40, 130 6 1.522 1.48 1.576 1.585 1.337 1.368 0.744 1.03 0.759 0.644 1.434 1.004 1.061 1.387 0.956 1.313 1.58 1.129 1.975 2.03 0.781 1.323 0.897 0.927 0.576 1.236 1.777 5.67

S. macdonaghi 71 Argentina, Entre Rios, Jtc. Rts. 40, 130 7 0.971 1.345 1.365 1.327 1.192 1.201 0.645 0.953 0.763 0.636 1.311 0.926 0.975 1.258 0.882 1.226 1.493 1.074 1.92 1.832 0.75 1.114 0.843 0.779 0.515 0.981 1.527 4.94

S. macdonaghi 71 Argentina, Entre Rios, Jtc. Rts. 40, 130 8 1.463 1.413 1.476 1.482 1.295 1.323 0.691 1.018 0.77 0.616 1.289 0.905 0.975 1.296 0.892 1.305 1.506 1.011 1.921 1.961 0.77 1.24 0.862 0.866 0.603 1.123 1.746 5.36

S. macdonaghi 71 Argentina, Entre Rios, Jtc. Rts. 40, 130 9 0.651 1.21 1.136 1.087 1.015 1.036 0.555 0.895 0.662 0.59 1.056 0.74 0.88 1.081 0.752 1.053 1.28 0.894 1.536 0.548 0.918 0.686 0.774 0.434 1.04 1.289 4.56

S. macdonaghi 71 Argentina, Entre Rios, Jtc. Rts. 40, 130 10 0.770 1.253 1.203 1.17 1.037 1.069 0.594 0.93 0.682 0.598 1.158 0.845 0.922 1.131 0.787 1.096 1.358 0.945 1.659 1.676 0.596 0.998 0.76 0.786 0.48 1.035 1.314 4.75

S. macdonaghi 71 Argentina, Entre Rios, Jtc. Rts. 40, 130 11 1.122 1.117 1.04 0.975 0.978 0.521 0.846 0.594 0.556 1.072 0.737 0.86 1.082 0.697 1.024 1.279 0.918 1.523 1.458 0.601 0.913 0.667 0.697 0.427 0.922 1.192 4.20

S. macdonaghi 71 Argentina, Entre Rios, Jtc. Rts. 40, 130 12 0.844 0.759 0.687 0.671 0.686 0.416 0.647 0.429 0.493 0.808 0.559 0.624 0.76 0.511 0.79 0.966 0.655 1.201 1.122 0.405 0.697 0.507 0.557 0.344 0.743 0.826 3.27

S. macdonaghi 71 Argentina, Entre Rios, Jtc. Rts. 40, 130 13 0.595 1.154 1.097 1.069 0.986 0.958 0.548 0.817 0.627 0.495 0.98 0.713 0.872 1.057 0.708 1.03 1.213 0.857 1.525 1.479 0.53 0.949 0.663 0.722 0.44 1.02 1.185 4.38

S. macdonaghi 71 Argentina, Entre Rios, Jtc. Rts. 40, 130 14 0.474 1.11 1.025 0.942 0.895 0.9 0.49 0.865 0.582 0.57 1.014 0.711 0.84 1.007 0.67 1.026 1.229 0.793 1.62 1.487 0.506 0.893 0.648 0.686 0.398 0.798 1.108 4.08

S. macdonaghi 71 Argentina, Entre Rios, Jtc. Rts. 40, 130 15 0.248 0.808 0.793 0.702 0.704 0.702 0.39 0.677 0.498 0.459 0.795 0.554 0.592 0.779 0.515 0.824 0.976 0.639 1.208 1.198 0.431 0.719 0.533 0.541 0.304 0.802 0.865 3.35

S. macdonaghi 71 Argentina, Entre Rios, Jtc. Rts. 40, 130 16 0.516 1.086 1.05 0.959 0.943 0.926 0.494 0.832 0.556 0.549 0.983 0.708 0.865 0.996 0.735 0.911 1.233 0.842 1.514 1.442 0.501 0.887 0.617 0.693 0.396 0.942 1.084 4.16

S. macdonaghi 71 Argentina, Entre Rios, Jtc. Rts. 40, 130 17 0.473 1.035 1.035 0.967 0.915 0.911 0.491 0.806 0.589 0.542 0.997 0.647 0.788 0.965 0.669 0.65 1.175 0.807 1.204 1.418 0.521 0.887 0.609 0.694 0.386 0.984 1.227 4.13

S. macdonaghi 71 Argentina, Entre Rios, Jtc. Rts. 40, 130 18 0.182 0.775 0.712 0.632 0.645 0.663 0.344 0.577 0.447 0.464 0.743 0.521 0.584 0.712 0.463 0.746 0.91 0.627 1.093 1.081 0.37 0.637 0.476 0.508 0.274 1.201 0.972 3.56

S. macdonaghi 71 Argentina, Entre Rios, Jtc. Rts. 40, 130 19 0.223 0.831 0.764 0.718 0.682 0.692 0.359 0.677 0.474 0.455 0.81 0.546 0.663 0.783 0.528 0.804 0.974 0.663 1.248 1.067 0.405 0.661 0.471 0.609 0.289 0.691 0.785 3.20

S. macdonaghi 71 Argentina, Entre Rios, Jtc. Rts. 40, 130 20 0.330 0.948 0.874 0.799 0.777 0.781 0.402 0.719 0.544 0.507 0.883 0.606 0.715 0.603 0.843 1.014 0.715 1.287 1.305 0.466 0.763 0.585 0.674 0.27 0.844 0.984 3.77

S. macdonaghi 71 Argentina, Entre Rios, Jtc. Rts. 40, 130 21 0.228 0.327 0.808 0.698 0.637 0.637 0.659 0.356 0.626 0.43 0.439 0.717 0.489 0.617 0.703 0.465 0 0.896 0.603 1.116 0.561 0.764 0.826 2.77

S. macdonaghi 71 Argentina, Entre Rios, Jtc. Rts. 40, 130 22 0.244 0.848 0.776 0.69 0.697 0.711 0.387 0.637 0.471 0.476 0.824 0.522 0.664 0.741 0.478 0.761 0.938 0.655 0.902 1.185 0.39 0.707 0.51 0.567 0.296 0.701 0.851 3.30

S. macdonaghi 71 Argentina, Entre Rios, Jtc. Rts. 40, 130 23 0.184 0.785 0.722 0.655 0.63 0.639 0.365 0.596 0.463 0.455 0.733 0.509 0.644 0.709 0.484 0.72 0.88 0.586 1.124 1.081 0.378 0.651 0.464 0.555 0.275 0.555 0.713 2.98

S. macdonaghi 71 Argentina, Entre Rios, Jtc. Rts. 40, 130 24 0.179 0.806 0.714 0.63 0.635 0.623 0.303 0.617 0.456 0.403 0.77 0.505 0.641 0.735 0.472 0.766 0.897 0.623 1.164 1.072 0.364 0.652 0.495 0.474 0.271 0.702 0.733 3.05

S. macdonaghi 71 Argentina, Entre Rios, Jtc. Rts. 40, 130 25 0.168 0.793 0.649 0.592 0.601 0.607 0.362 0.563 0.419 0.442 0.71 0.443 0.558 0.65 0.441 0.697 0.827 0.574 1.047 0.989 0.352 0.632 0.43 0.555 0.241 0.609 0.646 2.95

S. macdonaghi 71 Argentina, Entre Rios, Jtc. Rts. 40, 130 26 0.209 0.787 0.747 0.686 0.678 0.687 0.34 0.672 0.462 0.485 0.777 0.522 0.643 0.749 0.47 0.722 0.915 0.653 1.068 1.144 0.324 0.674 0.483 0.577 0.267 0.748 0.79 3.26

S. macdonaghi 71 Argentina, Entre Rios, Jtc. Rts. 40, 130 27 0.185 0 0.747 0.714 0.647 0.662 0.364 0.658 0.5 0.458 0.78 0.565 0.666 0.765 0.526 0.769 0.94 0.655 1.154 1.12 0.359 0.66 0.492 0.552 0.267 0.702 0.759 2.37

S. macdonaghi 71 Argentina, Entre Rios, Jtc. Rts. 40, 130 28 0.192 0.761 0.691 0.63 0.631 0.623 0.357 0.569 0.424 0.405 0.632 0.482 0.677 0.446 0.838 0.569 1.055 1.013 0.364 0.62 0.453 0.488 0.267 0.645 0.705 2.91

S. macdonaghi 71 Argentina, Entre Rios, Jtc. Rts. 40, 130 29 0.235 0.808 0.714 0.666 0.65 0.626 0.396 0.646 0.46 0.478 0.802 0.496 0.548 0.727 0.511 0.754 0.909 0.632 1.131 1.127 0.392 0.713 0.477 0.585 0.277 0.679 0.824 3.20

S. macdonaghi 71 Argentina, Entre Rios, Jtc. Rts. 40, 130 30 1.338 1.438 1.513 1.492 1.319 1.311 0.76 1.034 0.743 0.648 1.303 0.97 1.018 1.328 0.853 1.274 1.548 1.055 1.827 1.961 0.815 1.226 0.845 0.989 0.594 1.29 1.651 5.68

S. macdonaghi 71 Argentina, Entre Rios, Jtc. Rts. 40, 130 31 0.135 0.789 0.728 0.659 0.648 0.645 0.341 0.599 0.442 0.437 0.749 0.516 0.553 0.67 0.497 0.721 0.876 0.567 1.157 1.082 0.389 0.664 0.5 0.546 0.283 0.698 0.631 3.12

S. macdonaghi 71 Argentina, Entre Rios, Jtc. Rts. 40, 130 32 0.224 0.814 0.753 0.684 0.658 0.672 0.347 0.65 0.4 0.468 0.777 0.516 0.735 0.465 0.78 0.911 0.593 1.012 1.139 0.397 0.681 0.531 0.536 0.307 0.765 0.791 3.25

S. macdonaghi 71 Argentina, Entre Rios, Jtc. Rts. 40, 130 33 1.541 1.476 1.566 1.524 1.359 1.323 0.655 1.027 0.816 0.552 1.377 0.846 1.109 1.302 0.862 1.314 1.563 1.063 1.935 1.99 0.756 1.269 0.888 0.967 0.571 1.325 1.688 5.76

S. macdonaghi 71 Argentina, Entre Rios, Jtc. Rts. 40, 130 34 1.434 1.509 1.525 1.515 1.309 1.339 0.686 1.016 0.752 0.588 1.324 0.916 1.058 1.315 0.916 1.453 1.114 1.909 2.052 0.842 1.336 0.957 0.868 0.607 1.304 1.608 5.73

S. macdonaghi 71 Argentina, Entre Rios, Jtc. Rts. 40, 130 35 0.236 0.844 0.767 0.697 0.692 0.702 0.413 0.54 0.51 0.472 0.77 0.544 0.659 0.732 0.5 0.769 0.973 0.588 1.162 1.164 0.39 0.74 0.497 0.57 0.285 0.78 0.764 3.36

S. macdonaghi 71 Argentina, Entre Rios, Jtc. Rts. 40, 130 36 0.200 0.853 0.749 0.674 0.642 0.657 0.393 0.645 0.493 0.463 0.824 0.526 0.621 0.668 0.513 0.932 0.627 1.19 1.138 0.396 0.681 0.508 0.559 0.283 0.693 0.751 3.24

S. macdonaghi 71 Argentina, Entre Rios, Jtc. Rts. 40, 130 37 0.170 0.757 0.68 0.617 0.612 0.628 0.332 0.592 0.455 0.398 0.71 0.477 0.603 0.697 0.437 0.76 0.804 0.569 0.925 1.048 0.366 0.656 0.453 0.552 0.256 0.616 0.701 2.97

S. macdonaghi 71 Argentina, Entre Rios, Jtc. Rts. 40, 130 38 0.207 0.822 0.741 0.69 0.655 0.652 0.314 0.64 0.445 0.401 0.787 0.449 0.651 0.729 0.527 0.802 0.9 0.644 1.11 1.131 0.392 0.69 0.523 0.592 0.291 0.659 0.775 3.20

S. macdonaghi 71 Argentina, Entre Rios, Jtc. Rts. 40, 130 39 0.173 0.779 0.698 0.67 0.613 0.621 0.323 0.574 0.421 0.39 0.718 0.454 0.603 0.626 0.451 0.702 0.784 0.577 0.97 1.006 0.305 0.628 0.456 0.542 0.275 0.626 0.698 2.95

S. macdonaghi 71 Argentina, Entre Rios, Jtc. Rts. 40, 130 40 0.288 0.894 0.801 0.734 0.73 0.732 0.4 0.676 0.499 0.442 0.842 0.608 0.708 0.847 0.53 0.836 1.009 0.702 1.277 1.251 0.387 0.751 0.561 0.628 0.297 0.758 0.869 3.53

S. richteri 72 Argentina, San Luis, Sierra San Luis 1 1.123 1.359 1.373 1.359 1.197 1.231 0.599 0.913 0.697 0.597 1.153 0.84 0.904 1.198 0.863 1.18 1.343 1.001 1.832 1.791 0.626 1.121 0.805 0.941 0.561 1.307 1.576 5.40

S. richteri 72 Argentina, San Luis, Sierra San Luis 2 1.087 1.352 1.376 1.311 1.223 1.23 0.659 0.972 0.707 0.624 1.176 0.863 0.919 1.221 0.841 1.112 1.41 1.045 1.797 1.768 0.711 1.164 0.797 0.948 0.52 1.236 1.576 5.30

S. richteri 72 Argentina, San Luis, Sierra San Luis 3 1.210 1.413 1.413 1.365 1.245 1.206 0.669 1 0.729 0.57 1.24 0.928 0.952 1.22 0.908 1.51 1.093 2.051 1.928 0.696 1.175 0.873 1.028 0.552 1.405 1.646 5.77

S. richteri 72 Argentina, San Luis, Sierra San Luis 4 1.088 1.369 1.347 1.325 1.219 1.235 0.611 0.983 0.646 0.575 1.229 0.882 0.954 1.231 0.859 1.425 1.034 1.71 1.776 0.616 1.134 0.821 0.925 0.519 1.221 1.515 5.29

S. richteri 72 Argentina, San Luis, Sierra San Luis 5 0.588 1.14 1.099 1.059 1.008 0.973 0.486 0.846 0.636 0.542 1.076 0.756 0.804 1.06 0.725 1.015 1.277 0.902 1.393 1.492 0.529 0.938 0.689 0.729 0.409 1.056 1.107 4.42

S. richteri 72 Argentina, San Luis, Sierra San Luis 6 0.935 1.281 1.265 1.222 1.128 1.103 0.598 0.926 0.648 0.591 1.097 0.829 0.915 1.131 0.802 1.102 1.352 0.975 1.493 1.71 0.657 1.082 0.795 0.863 0.451 1.221 1.463 5.08

S. richteri 72 Argentina, San Luis, Sierra San Luis 7 1.160 1.345 1.42 1.413 1.275 1.233 0.611 1.02 0.673 0.647 1.189 0.803 0.806 1.252 0.87 1.181 1.49 1.032 1.796 1.847 0.7 1.159 0.86 0.905 0.572 1.309 1.656 5.41

S. richteri 72 Argentina, San Luis, Sierra San Luis 8 1.159 1.355 1.398 1.376 1.249 1.204 0.715 1.013 0.708 0.569 1.201 0.92 0.871 1.183 0.852 1.25 1.365 1.053 1.834 1.859 0.702 1.231 0.851 0.907 0.548 1.32 1.561 5.44

S. richteri 72 Argentina, San Luis, Sierra San Luis 9 0.546 1.151 1.102 1.06 1.024 0.984 0.57 0.867 0.642 0.55 1.058 0.731 0.867 0.984 0.738 0.802 1.208 0.838 1.598 1.491 0.541 0.932 0.657 0.74 0.44 0.993 1.2 4.38

S. richteri 72 Argentina, San Luis, Sierra San Luis 10 0.455 1.044 1.005 0.936 0.887 0.894 0.478 0.78 0.588 0.558 0.851 0.701 0.739 0.976 0.701 1.009 1.148 0.852 1.493 1.423 0.433 0.857 0.635 0.776 0.363 0.909 1.07 4.15

S. richteri 72 Argentina, San Luis, Sierra San Luis 11 0.497 1.078 1.01 0.97 0.929 0.894 0.493 0.806 0.614 0.549 0.98 0.683 0.774 0.943 0.679 0.901 1.16 0.834 1.422 1.408 0.494 0.834 0.597 0.702 0.382 1.022 1.084 4.21

S. richteri 72 Argentina, San Luis, Sierra San Luis 12 0.288

S. richteri 72 Argentina, San Luis, Sierra San Luis 13 0.186 0.807 0.733 0.675 0.669 0.661 0.324 0.597 0.474 0.437 0.722 0.504 0.592 0.737 0.5 0.83 0.871 0.653 1.12 1.072 0.338 0.632 0.476 0.571 0.283 0.738 0.769 3.19

S. richteri 72 Argentina, San Luis, Sierra San Luis 14 0.396 0.988 0.956 0.884 0.883 0.836 0.464 0.754 0.586 0.541 0.919 0.621 0.78 0.905 0.614 1.068 0.747 1.378 1.317 0.416 0.789 0.575 0.704 0.353 0.911 0.982 3.92

S. richteri 72 Argentina, San Luis, Sierra San Luis 15 0.280 0.846 0.832 0.776 0.77 0.742 0.374 0.688 0.515 0.478 0.811 0.583 0.738 0.784 0.538 0.86 0.969 0.709 1.209 1.185 0.417 0.723 0.528 0.621 0.318 0.834 0.864 3.49

S. richteri 72 Argentina, San Luis, Sierra San Luis 16 0.184 0.762 0.702 0.657 0.667 0.658 0.31 0.613 0.404 0.412 0.675 0.512 0.654 0.675 0.434 0.838 0.57 1.146 1.041 0.316 0.619 0.452 0.522 0.262 0.736 0.78 3.06

S. richteri 72 Argentina, San Luis, Sierra San Luis 17 0.243 0.837 0.795 0.733 0.748 0.738 0.379 0.683 0.5 0.428 0.789 0.552 0.663 0.783 0.536 0.8 0.937 0.658 1.097 1.149 0.346 0.705 0.511 0.601 0.291 0.708 0.824 3.30

S. richteri 72 Argentina, San Luis, Sierra San Luis 18 0.372 0.98 0.902 0.846 0.854 0.819 0.478 0.698 0.529 0.534 0.928 0.666 0.716 0.928 0.639 0.929 1.097 0.776 1.321 1.321 0.425 0.795 0.574 0.689 0.358 0.911 0.941 3.90

S. richteri 72 Argentina, San Luis, Sierra San Luis 19 0.237 0.843 0.796 0.722 0.717 0.712 0.388 0.586 0.468 0.534 0.802 0.558 0.584 0.776 0.548 0.93 0.639 1.182 1.15 0.364 0.647 0.513 0.614 0.297 0.786 3.39

S. richteri 72 Argentina, San Luis, Sierra San Luis 20 0.163 0.761 0.696 0.639 0.624 0.64 0.317 0.615 0.441 0.469 0.739 0.511 0.593 0.709 0.476 0.844 0.606 1.056 1.052 0.343 0.636 0.442 0.553 0.261 0.708 0.695 3.07

S. richteri 72 Argentina, San Luis, Sierra San Luis 21 0.174 0.741 0.684 0.62 0.623 0.621 0.358 0.594 0.415 0.442 0.716 0.488 0.614 0.642 0.454 0.685 0.799 0.494 1.043 1.027 0.348 0.626 0.459 0.527 0.271 0.649 0.769 2.94

S. richteri 72 Argentina, San Luis, Sierra San Luis 22 0.187 0.748 0.71 0.66 0.659 0.647 0.319 0.629 0.459 0.447 0.757 0.513 0.574 0.711 0.483 0.713 0.851 0.582 1.085 1.06 0.358 0.641 0.454 0.546 0.267 0.712 0.714 3.07

S. richteri 72 Argentina, San Luis, Sierra San Luis 23 0.229 0.863 0.765 0.689 0.713 0.716 0.386 0.662 0.46 0.489 0.769 0.582 0.675 0.754 0.52 0.8 0.953 0.639 1.315 1.135 0.377 0.675 0.514 0.566 0.298 0.728 0.843 3.29

S. richteri 72 Argentina, San Luis, Sierra San Luis 24 0.213 0.894 0.838 0.776 0.781 0.764 0.401 0.71 0.505 0.513 0.839 0.606 0.663 0.819 0.582 0.792 0.992 0.694 1.269 1.2 0.367 0.734 0.52 0.595 0.325 0.762 0.892 3.45

S. richteri 72 Argentina, San Luis, Sierra San Luis 25 0.210 0.836 0.753 0.686 0.689 0.696 0.345 0.643 0.456 0.441 0.787 0.523 0.658 0.653 0.493 0.795 0.887 0.618 1.076 1.083 0.358 0.678 0.49 0.507 0.277 0.706 0.798 3.13

S. richteri 72 Argentina, San Luis, Sierra San Luis 26 0.194

S. richteri 72 Argentina, San Luis, Sierra San Luis 27 0.301 0.897 0.812 0.743 0.756 0.75 0.394 0.688 0.484 0.49 0.848 0.588 0.716 0.834 0.58 0.845 0.983 0.701 0 1.247 0.416 0.755 0.551 0.624 0.299 0.796 0.855 3.56

S. richteri 72 Argentina, San Luis, Sierra San Luis 28 0.211 0.838 0.773 0.678 0.707 0.713 0.404 0.609 0.494 0.476 0.667 0.546 0.636 0.768 0.518 0.762 0.894 0.582 1.154 1.13 0.36 0.698 0.502 0.558 0.288 0.747 0.805 3.27

S. richteri 72 Argentina, San Luis, Sierra San Luis 29 1.234 1.374 1.389 1.358 1.227 1.201 0.644 0.977 0.676 0.644 1.273 0.912 1.004 1.232 0.897 1.256 1.457 1.038 1.867 1.86 0.686 1.158 0.809 0.819 0.536 1.406 1.482 5.46

S. richteri 72 Argentina, San Luis, Sierra San Luis 30 0.302 0.946 0.847 0.78 0.791 0.785 0.386 0.7 0.494 0.419 0.819 0.608 0.744 0.828 0.571 1.024 0.705 1.298 1.244 0.391 0.738 0.539 0.569 0.321 0.769 0.855 3.53

S. richteri 72 Argentina, San Luis, Sierra San Luis 31 0.818 1.266 1.239 1.166 1.129 1.102 0.527 0.921 0.653 0.602 1.141 0.821 1.069 0.812 1.085 1.399 0.988 1.689 0.604 1.009 0.743 0.702 0.471 1.156 1.403 4.81

S. richteri 72 Argentina, San Luis, Sierra San Luis 32 0.269 0.834 0.781 0.715 0.721 0.702 0.356 0.643 0.482 0.424 0.814 0.578 0.706 0.71 0.522 0.835 0.929 0.677 1.194 1.132 0.369 0.736 0.5 0.611 0.305 0.812 0.848 3.39

S. richteri 72 Argentina, San Luis, Sierra San Luis 33 0.263 0.863 0.773 0.713 0.727 0.719 0.363 0.669 0.487 0.481 0.787 0.583 0.697 0.756 0.553 0.779 0.929 0.686 1.143 1.158 0.38 0.697 0.517 0.557 0.295 0.765 0.855 3.34

S. richteri 72 Argentina, San Luis, Sierra San Luis 34 0.764 1.185 1.192 1.143 1.083 1.025 0.581 0.887 0.686 0.604 1.097 0.819 0.927 1.082 0.804 1.043 1.346 0.948 1.609 1.591 0.533 0.994 0.702 0.755 0.452 1.06 1.283 4.59

S. richteri 72 Argentina, San Luis, Sierra San Luis 35 1.095 1.255 1.322 1.306 1.222 1.152 0.673 0.952 0.678 0.613 1.178 0.805 0.872 1.155 0.805 1.181 1.384 1.045 1.741 1.75 0.611 1.078 0.773 0.836 0.49 1.191 1.532 5.03

S. richteri 72 Argentina, San Luis, Sierra San Luis 36 0.199 0.769 0.713 0.651 0.665 0.661 0.369 0.612 0.439 0.426 0.732 0.459 0.622 0.725 0.522 0.714 0.851 0.579 1.104 1.05 0.363 0.634 0.445 0.515 0.281 0.582 0.769 2.92

S. richteri 72 Argentina, San Luis, Sierra San Luis 37 0.195 0.849 0.757 0.66 0.716 0.694 0.379 0.671 0.459 0.463 0.754 0.605 0.543 0.761 0.541 0.909 0.657 1.243 1.139 0.379 0.699 0.517 0.573 0.278 0.716 0.8 3.28

S. richteri 72 Argentina, San Luis, Sierra San Luis 38 0.174 0.727 0.659 0.605 0.611 0.597 0.322 0.577 0.437 0.445 0.685 0.474 0.621 0.683 0.478 0.689 0.816 0.585 1.049 1.049 0.314 0.628 0.453 0.479 0.273 0.631 0.706 2.89

S. richteri 72 Argentina, San Luis, Sierra San Luis 39 0.331 0.945 0.883 0.817 0.811 0.791 0.409 0.733 0.512 0.862 0.602 0.714 0.843 0.588 0.833 1.093 0.729 1.29 1.237 0.4 0.736 0.562 0.567 0.342 0.838 0.935 3.59

S. richteri 72 Argentina, San Luis, Sierra San Luis 40 0.603 1.108 1.052 0.995 0.983 0.942 0.539 0.808 0.546 0.537 0.984 0.712 0.814 0.989 0.678 0.999 1.207 0.856 1.507 1.445 0.507 0.878 0.648 0.713 0.403 0.955 1.117 4.22
